# Fe-LMCT Photodecarboxylation for (Hetero)arene Chloro- and Bromodifluoromethylation: Rapid Access to Aromatic Acyl Fluorides

**DOI:** 10.1021/acs.orglett.6c01214

**Published:** 2026-04-20

**Authors:** Sara Fernández-García, Paula Visiedo-Jiménez, Francisco Juliá-Hernández

**Affiliations:** Facultad de Química, Centro Multidisciplinar Pleiades-Vitalis, 16751 Universidad de Murcia, Campus de Espinardo, 30100 Murcia, Spain

## Abstract

Chloro- and bromodifluoromethyl groups (CF_2_X) offer complementary properties to other fluorinated substituents in modulating biological functionality and provide versatile synthetic handles for postfunctionalization. However, their widespread use remains limited by the scarcity of practical halodifluoromethylating reagents. Here, we report a photoinduced Fe-catalyzed strategy that repurposes readily available chloro- and bromodifluoroacetates as traceless radical precursors for (hetero)­arene C–H halodifluoroalkylations. The resulting products exhibit broad synthetic versatility, enabling streamlined one-pot access to valuable acyl fluoride synthons.

## Graphical abstract

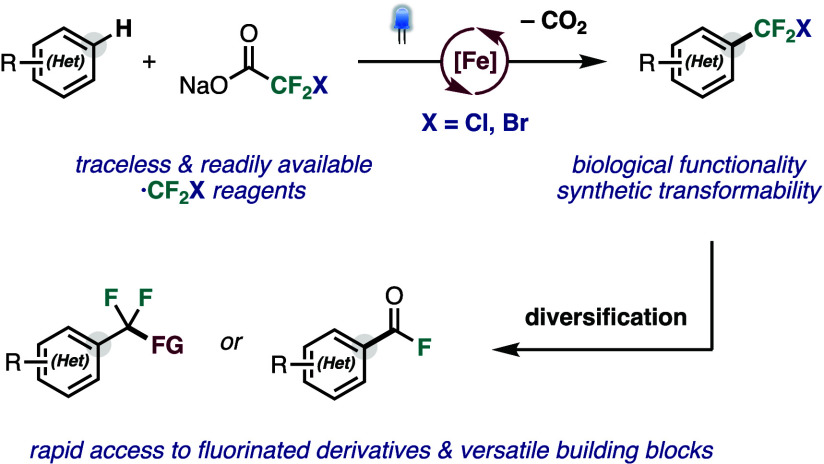

Fluorinated functional groups are widely employed in modern small-molecule drugs, where strategic fluorine incorporation modulates lipophilicity, membrane permeability, bioavailability and overall biological performance.^1,2^ Among these motifs, the trifluoromethyl group (CF_3_) has become a cornerstone substituent, particularly on (hetero)­aromatic scaffolds.^3^ Its strong electron-withdrawing character and low polarizability impart exceptional chemical and metabolic robustness.^4^ However, this same inertness limits opportunities for postinstallation diversification and directional noncovalent interactions. The development of fluoroalkyl groups with complementary properties and reactivity profiles therefore represents an important frontier in expanding the functional landscape of fluorinated molecules.^5^ In this context, chloro- and bromodifluoromethyl substituents (CF_2_X, X = Cl, Br) offer an orthogonal combination of properties (Figure 1A). While maintaining substantial inductive electron withdrawal, these groups contain a polarizable halogen atom capable of engaging in halogen-bonding interactions.^6^ This property enables additional modes of molecular recognition and solid-state organization that are inaccessible to CF_3_ analogues^7,8^ and may be advantageous in various applications.^9^ This effect is exemplified by the enhanced activity of the CF_2_Cl-containing drug asciminib (Scemblix), which was recently approved by the FDA to treat blood cancers.^2^ Moreover, the C–X bond provides a latent synthetic handle for downstream diversification, including reduction,^10^ nucleophilic substitution^11^ and further derivatization processes,^12^ rendering CF_2_X a strong potential for the rapid generation of libraries of fluorinated compounds of interest for medicinal chemistry.^1,5^ Furthermore, CF_2_X-functionalized compounds are valuable precursors for the development of ^18^F-labeled radiopharmaceuticals via X/F exchange reactions.^13^ Despite these attractive features, CF_2_X-containing (hetero)­arenes have not yet been widely adopted in applied settings. A principal obstacle lies in the limited access to general methods for their direct installation, particularly via C–H functionalization, in contrast to well-established trifluoromethylation strategies.^14^ Existing approaches typically rely on prefunctionalized substrates or forcing activation protocols,^15–19^ which can reduce their suitability for late-stage modification of complex molecules, an essential consideration in hit-to-lead optimization during drug discovery.^20^

**Figure 1:**
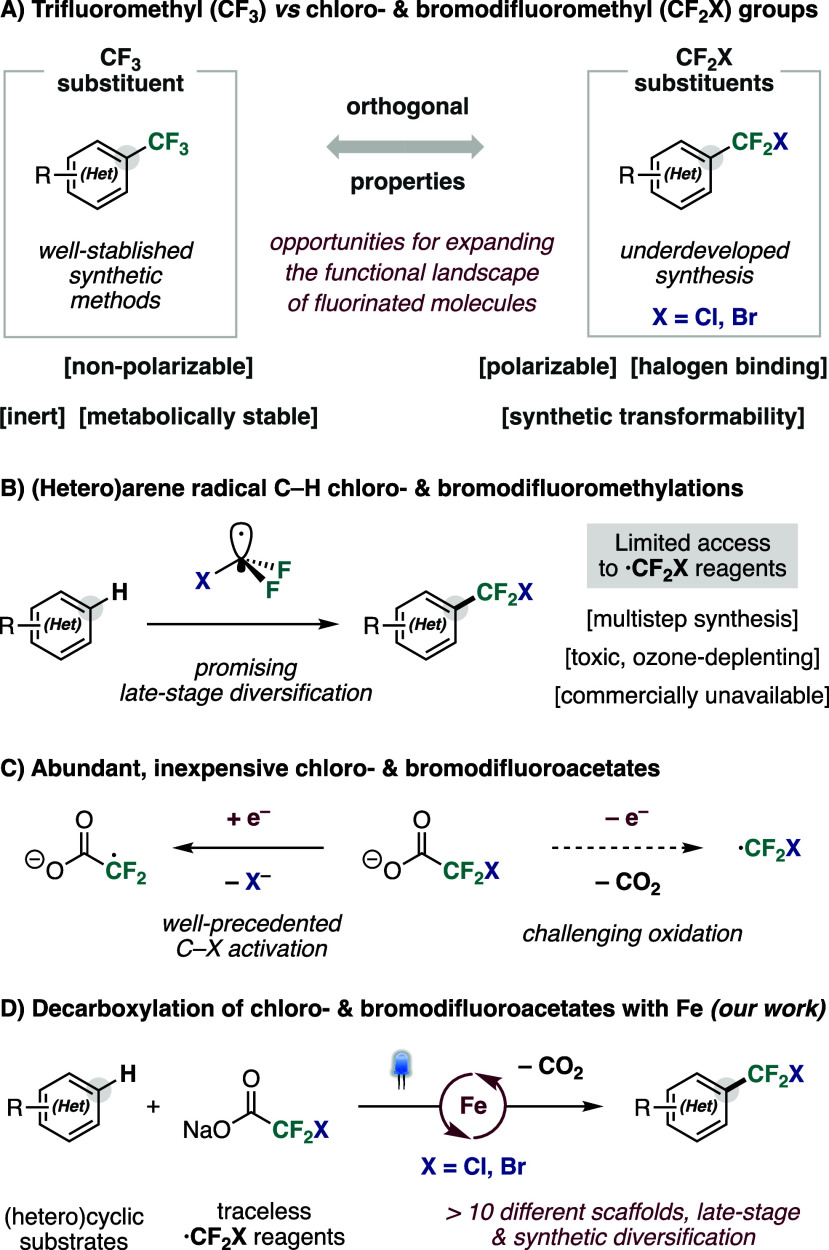
(A) Properties of chloro- and bromodifluoromethyl substituents. (B) Radical C–H chloro- and bromodifluoromethylation of (hetero)­arenes. (C) Activation of chloro- and bromodifluoroacetates via SET processes. (D) (Hetero)­arene C–H fluoroalkylation via direct decarboxylation with Fe photocatalysis (*our work*).

From an electronic perspective, chloro- and bromodifluoromethyl radicals (·CF_2_X) are electrophilic species that can potentially engage in the direct C–H fluoroalkylation of previously unfunctionalized (hetero)­aromatic substrates, similar to established trifluoromethylation methods.^21^ While this could constitute a promising approach for CF_2_X incorporation, this remains underdeveloped mainly because of the limited availability of practical radical chloro- and bromodifluoromethylating reagents (Figure 1B). Although notable advances have been reported,^20,22–25^ the dependence on specialized, noncommercially available reagents that require multistep preparation or originate from toxic and ozone-depleting precursors, remains a substantial barrier to widespread implementation.

Photocatalytic radical decarboxylation of chloro- and bromodifluoroacetates represents an appealing alternative, as these salts are bench-stable, inexpensive, and among the most accessible sources of CF_2_X units, potentially serving as traceless radical precursors under visible-light irradiation. Nevertheless, extending canonical (photo)­redox decarboxylation strategies to such electron-deficient fluorinated carboxylates remains challenging.^26–29^ Their high oxidation potentials render outer-sphere single-electron activation difficult,^21^ while the C–X bond is susceptible to competitive reductive pathways that can divert productive radical generation, giving rise to elegant synthetic protocols (Figure 1C).^30–33^ Although recent approaches have addressed these issues through preactivation with stoichiometric activating groups,^34–39^ the direct catalytic decarboxylation of chloro- and bromodifluoroacetates for the C–H fluoroalkylation of (hetero)­arenes, incorporating electron-rich substrates, remains largely undeveloped.^40^

Building on our pioneering studies on the direct photodecarboxylation of trifluoroacetates for (hetero)­arene trifluoromethylations via iron ligand-to-metal charge transfer (LMCT) catalysis,^41,42^ we hypothesized that chloro- and bromodifluoroacetates could undergo direct decarboxylation via Fe-LMCT catalysis,^43,44^ thereby generating CF_2_X radicals upon visible-light illumination. If successful, this strategy would establish readily available chloro- and bromodifluoroacetate salts as practical and modular CF_2_X radical precursors for (hetero)­arene C–H functionalization, obviating the need for specialized fluoroalkylating reagents. Complementary Fe-LMCT approaches for alkene fluoroalkylation have recently emerged, further underscoring the potential of this activation mode.^45–47^ Very recently, Nocera and co-workers reported the fluoroalkylation of electron-deficient and electron-neutral arenes under Ag photoelectrochemical settings.^40^ Herein, we report the development of a general platform for C­(sp^2^)–H (hetero)­arene chloro- and bromodifluoromethylation that repurposes simple chloro- and bromodifluoroacetate feedstocks using Earth-abundant iron catalysts and visible light as a sustainable energy input (Figure 1D). The protocol operates under mild conditions and accommodates a variety of (hetero)­cyclic scaffolds, including electron-rich substrates that could potentially prove problematic under alternative methodologies.^40^ Moreover, the resulting CF_2_X products serve as versatile intermediates, enabling rapid diversification through exploitation of the labile C–Cl and C–Br bonds. Finally, we demonstrate that this strategy can be extended to a one-pot synthesis of the corresponding aromatic acyl fluorides, collectively constituting a direct C–H fluoroacylation manifold.

At the outset of our investigations, we questioned whether the direct decarboxylation of chloro- and bromodifluoroacetates using Fe-LMCT catalysis would be effective for generating CF_2_X radicals.^42^ In particular, we anticipated potential challenges arising from the reduced stability of ·CF_2_X species relative to CF_3_ radicals, given the inherent lability of the C–X bonds.

To this end, 4-*tert*-butylanisole and sodium chlorodifluoroacetate were subjected to a catalytic system comprising Fe­(OTf)_2_ (OTf, trifluoromethanesulfonate) and 4,4′-dimethoxy-2,2′-bipyridine (**L1**), which, in the presence of K_2_S_2_O_8_ as an inorganic oxidant, generated the photoactive Fe­(III) species in situ. The reaction was conducted in MeCN at 25 °C under irradiation at 405 nm. To our delight, the corresponding chlorodifluoromethylated product **1a** was detected in 78% yield after 24 h (Table 1, entry 1), validating the feasibility of our strategy. Variation of the ligand nature and Fe/ligand ratio did not result in a significant improvement in reactivity.^48^ Control experiments demonstrated the photoinduced activity of the in situ formed Fe­(III) species. No product formation was observed in the absence of iron, oxidant or performing the reaction in the dark (Table 1, entries 2–4). Notably, the photocatalytic platform was also compatible with sodium bromodifluoroacetate, enabling the synthesis of the corresponding bromodifluoromethylated derivative **1b** (Table 1, entry 5).

**Table 1:**
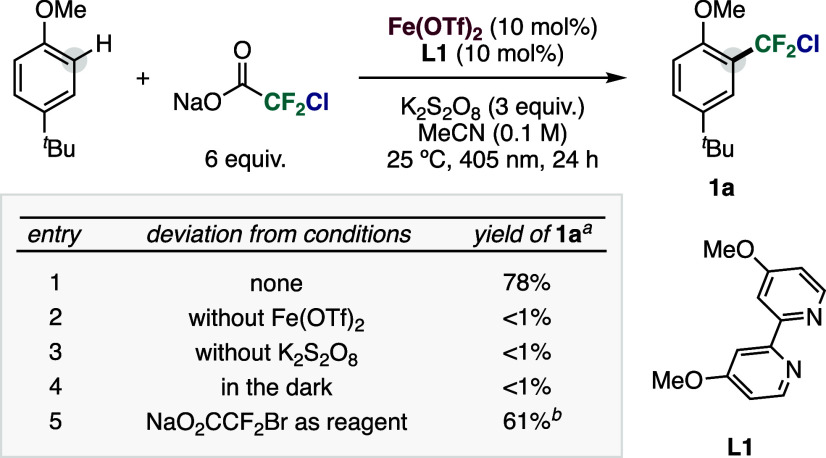
Screening of Reaction Conditions^a^

a Reaction conditions: 4-*tert*-butylanisole (0.3 mmol), NaO_2_CCF_2_Cl (6 equiv), Fe­(OTf)_2_ (10 mol %), **L1** (10 mol %), K_2_S_2_O_8_ (3 equiv), acetonitrile (0.1 M), 405 nm irradiation, 25 °C, 24 h. Yields determined by ^19^F NMR.

b Yield of the corresponding bromodifluoromethylated compound **1b**.

Following optimization, we evaluated the substrate scope of the decarboxylative chloro- and bromodifluoromethylation reaction, demonstrating the installation of CF_2_X groups across diverse (hetero)­cyclic motifs (Figure 2). We first explored the chlorodifluoromethylation reaction. Simple, electron-rich arenes with various substitution patterns underwent selective C–H functionalization to furnish the corresponding chlorodifluoromethylated products in good yields (**1a**–**5a**). Functionalization occurred preferentially at the most electron-rich C­(sp^2^)–H position, consistent with a radical aromatic substitution pathway involving an electrophilic C-centered radical. Mono- and bis-substituted products were often observed when multiple reactive sites were present (**4a**), as commonly encountered in radical fluoroalkylation reactions. The methodology proved compatible with 5-membered heteroarenes, including thiophene (**6a**) and substituted pyrroles (**7a**–**9a**), demonstrating the utility of the synthetic protocol to functionalize electron-rich substrates. Also, 6-membered heterocycles with varying electronic properties, such as 2-pyridone (**10a**), pyrimidine (**11a**) and pyridine (**12a**) were converted into their corresponding chlorodifluoromethylated derivatives. Functionalized indoles (**13a**–**15a**) also underwent smooth chlorodifluoromethylation, yielding both mono- and bis-substituted products depending on the availability of reactive positions within the indole core.

**Figure 2:**
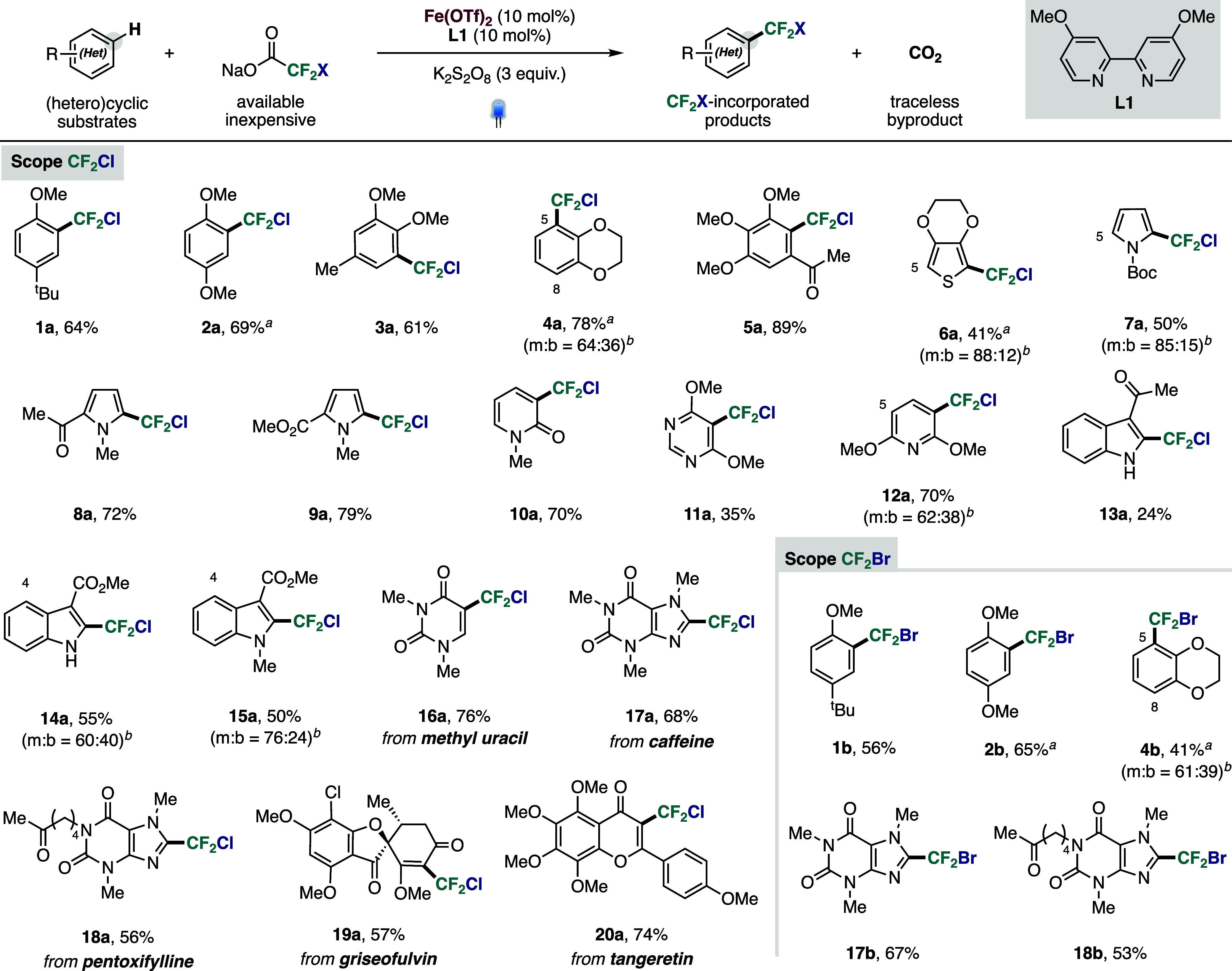
Scope of the chloro- and bromodifluoromethylation reaction. Isolated yields. See Supporting Information (SI) for details. ^*a*^Yield determined by ^19^F NMR due to volatility. ^*b*^Ratio between mono (m) and bis-functionalized (b) products. Minor regioisomeric position labeled with atom number.

To showcase the practicality of the protocol, we applied it to complex molecules, including natural products, pharmaceuticals, and drug-like scaffolds. Methyl uracil (**16a**), caffeine (**17a**) and pentoxifylline (**18a**) were efficiently functionalized, providing the corresponding fluorinated derivatives in good yields. More structurally complex substrates, such as the antifungal agent griseofulvin (Gris-PEG) and the bioactive flavonoid tangeretin, underwent selective functionalization at the olefinic enone moiety, affording previously unreported derivatives (**19a** and **20a**). These examples highlight the potential of this strategy for late-stage diversification, enabling the possibility for rapid construction of libraries of fluorinated compounds. Next, we extended the methodology to bromodifluoromethylation. Using sodium bromodifluoroacetate under otherwise identical conditions, a representative set of arenes (**1b**–**4b**) and heteroarenes (**17b** and **18b**) were successfully converted to the corresponding CF_2_Br derivatives bearing a more labile C–Br bond, demonstrating the generality of the platform for the installation of both CF_2_Cl and CF_2_Br groups. While the chlorodifluoromethylated products are generally bench-stable, some bromodifluoromethylated derivatives show limited stability in solution.

Mechanistically, the reaction is proposed to proceed via radical decarboxylation of the corresponding chloro- and bromodifluoroacetates through inner-sphere electron transfer, affording the corresponding CF_2_X radicals (Figure S8).^48^ The involvement of these radical species was supported by radical trap experiments with the addition of TEMPO and 1,1-diphenylethylene (Figures S9–S11).^48^ Regarding the photodecarboxylation step, UV–Vis spectroscopy of in situ-formed Fe­(III) chlorodifluoroacetate intermediates showed that 405 nm irradiation generated an Fe­(II) band, consistent with simultaneous Fe­(III) photoreduction and fluorinated acetate decarboxylation. (Figure S16).^48^ Fluoroalkylated product was detected in the presence of the substrate (Figure S14).^48^ Although preliminary, these experiments provide evidence for the inner-sphere character of the photodecarboxylation process^49^ and the intermediacy of CF_2_X radicals.

The synthetic utility for downstream diversification of the chloro- and bromodifluoromethylated compounds, featuring labile C–Cl and C–Br bonds, was demonstrated through postfunctionalization reactions. Chlorodifluoromethylated caffeine (**17a**) was converted into new fluorinated derivatives bearing C–O and C–Si bonds (**21** and **22**) via classical nucleophilic displacement or umpolung strategies (Figure 3A).^35,38^ In addition, functional group interconversion of the CF_2_Cl-containing 2-pyridone **10a** afforded methyl ester **23** (Figure 3B).^37^

**Figure 3:**
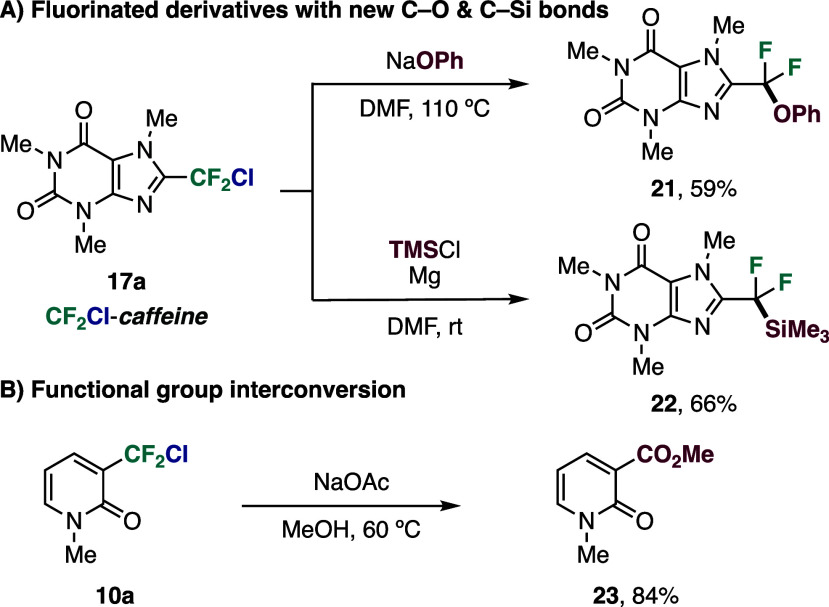
(A) Formation of new C–O and C–Si bonds. (B) Functional group interconversion. See SI for experimental details.

Capitalizing on the synthetic transformability of the chloro- and bromodifluoromethylated compounds, we sought to streamline the synthesis of the corresponding aromatic acyl fluorides. These versatile carboxylic acid derivatives are typically prepared using specialized fluorinating reagents. Acyl fluorides combine high electrophilicity with remarkable stability relative to other acyl halides, providing versatile bench-stable intermediates for the synthesis of esters, amides or ketones.^50–52^ Inspired by the recent work from Zhao and co-workers,^22^ we realized a one-pot conversion of the chloro- and bromodifluoromethylated products into (hetero)­aromatic acyl fluorides by simple addition of DMSO to the crude reaction mixture following fluoroalkylation, constituting a formal C–H fluoroacylation reaction with few precedents (Figure 4A).^22^ Under these conditions, a broad range of (hetero)­cycles could be converted into the corresponding acyl fluoride derivatives, including arenes with diverse substitution patterns (**24**–**27**) as well as heterocycles with varying electronic properties such as thiophene (**28**), pyrrole (**29**), pyridine (**30**) and pyrimidine (**31**), all of which were successfully isolated. To further showcase the synthetic potential of these acyl fluorides, we explored their direct derivatization with structurally complex nucleophiles (Figure 4B). To illustrate this, compound **30** was converted into the corresponding ester with estrone (**32**), and into an amide with Ibrutinib (**33**), a clinically used drug for the treatment of certain lymphomas, demonstrating the utility of these building blocks for late-stage diversification of bioactive molecules.

**Figure 4:**
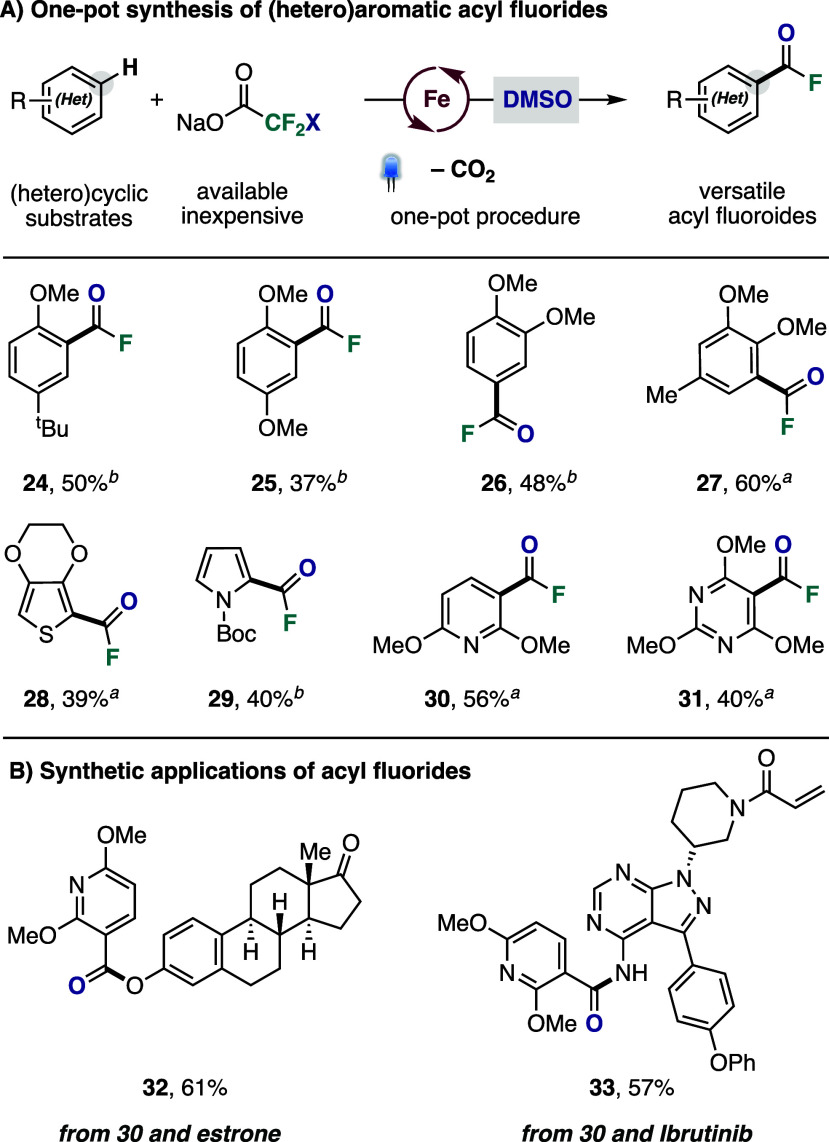
(A) (Hetero)­arene C–H fluoroacylation reactions. Isolated yields. ^*a*^With NaO_2_CCF_2_Cl. ^*b*^With NaO_2_CCF_2_Br. (B) Synthetic applications of acyl fluorides. See SI for details.

In conclusion, we have developed a general strategy for the chloro- and bromodifluoromethylation of diverse (hetero)­cyclic scaffolds, including electron-rich substrates and late-stage functionalization of industrially relevant compounds. The protocol is simple and practical, employing Earth-abundant iron catalysts, visible light and readily available fluorinated acetate salts as traceless radical precursors. The synthetic utility of the CF_2_X products was demonstrated through multiple downstream transformations, providing rapid access to fluorinated derivatives of potential interest in medicinal chemistry. Furthermore, a streamlined one-pot protocol enabled the synthesis of (hetero)­aromatic acyl fluorides, which are bench-stable, activated carboxylic acid synthons. Taken together, the broad substrate scope and versatility of these protocols offer a practical platform for the preparation of chloro- and bromodifluoromethylated (hetero)­cyclic compounds, allowing their implementation in applied and late-stage functionalization settings.

## Supplementary Material



## Data Availability

The data underlying this study are available in the published article, in its Supporting Information, and openly available in *Zenodo* at 10.5281/zenodo.19052940.

## Notes

Experimental procedures, characterization data and NMR spectra (PDF)
