# Supplementary material for: Fe-LMCT Photodecarboxylation for (Hetero)arene Chloro- and Bromodifluoromethylation: Rapid Access to Aromatic Acyl Fluorides
Source: Org Lett. 2026 Apr 20;28(17):5574–9. doi: 10.1021/acs.orglett.6c01214 (PMC13140126; doi:10.1021/acs.orglett.6c01214)

Supplementary Information for

Fe-LMCT Photodecarboxylation for (Hetero)arene Chloro- and Bromodifluoromethylation: Rapid Access to Aromatic Acyl Fluorides

Sara Fernández-García,[‡] Paula Visiedo-Jiménez,[‡] Francisco Juliá-Hernández[*]

Facultad de Química, Centro Multidisciplinar Pleiades-Vitalis, Universidad de Murcia, Campus de Espinardo, 30100 Murcia (Spain).

[‡]S. F.-G. and P. V.-J. contributed equally to this work.

*Corresponding author: francisco.julia@um.es

TABLE OF CONTENTS

General considerations **3**

Photoreactor 4

Light source 5

Optimization of the reaction conditions **6**

General procedure for screening reactions 6

Screening of ligands 7

Screening of ligand loading 8

Loading of sodium chlorodifluoroacetate 9

Control experiments 10

General reaction procedure for chlorodifluoromethylations **11**

Scope of the chlorodifluoromethylation reaction **13**

General reaction procedure for bromodifluoromethylations **29**

Scope of the bromodifluoromethylation reaction **30**

Synthetic applications **36**

General reaction procedure for C–H fluorocarbonylations **39**

Scope of the C–H fluoroacylation reaction **40**

Diversification of acyl fluorides **45**

Mechanistic investigations **47**

Mechanistic hypothesis 47

Radical trapping experiments 48

UV-Vis absorption spectroscopy study 51

References **55**

NMR spectral data **57**

GENERAL CONSIDERATIONS

Material and reagents

Commercially available reagents were purchased from Sigma Aldrich, Acros Organic, Alfa Aesar and/or Fluorochem and used directly without purification. Anhydrous MeCN was purchased from Acros Organics as extra dry and 99.9+% purity. The solvents used in column chromatography were obtained from commercial suppliers and used without further purification.

Chromatography

Thin layer chromatography (TLC) was carried out on 0.25 mm Merck silica plates (60F-254) using UV light (λ = 254, 365 nm) as visualizing agent as well as potassium permanganate stains. Flash column chromatography was performed using Merck flash silica gel (particle size 0.043-0.063 mm). Dry-loading was used to introduce the sample into the column using Celite® as supporting material.

Analytical methods

Gas chromatography was performed on an Agilent 5973 mass spectrometer coupled to an Agilent 6890N gas chromatograph. High-resolution mass spectra (HRMS) were determined using an Agilent 1920 Infinity II HPLC module, an Agilent Q-TOF 7250B, and an Agilent Q-TOF 6550 hybrid mass spectrometer with JetStream electrospray + i-Funnel ionization source. UV-Vis measurements were recorded in a Horiba Duetta fluorescence and absorbance spectrometer. NMR spectra for the characterization of compounds were recorded on a Bruker instrument 400 MHz (^{1}H) and at 101 MHz (^{13}C) and 376 MHz (^{19}F) at 25 ºC. Chemical shifts (δ) are reported in ppm, using the residual solvent peak in $CDCl_3$ (^{1}H = 7.26 and ^{13}C = 77.16 ppm), DMSO-d^6 (^{1}H = 2.50 and ^{13}C = 39.52 ppm), CD_3OD (^{1}H = 3.31 and ^{13}C = 49.00 ppm). Coupling constants, *J*, are reported in hertz. All ^{13}C NMR spectra were obtained with ^{1}H decoupling.

Photoreactor

Photocatalytic reactions were carried out in a photoreactor consisting of 8 reaction-vial slots equipped with 8 independent LEDs with temperature control (Fig. S1). Its design provides a constant magnetic stirring and a strict control of temperature and light intensity, which ensures reproducibility. Reaction vials are placed at 1 cm distance from the light source.

Solvent recirculation system to control the reaction temperature.

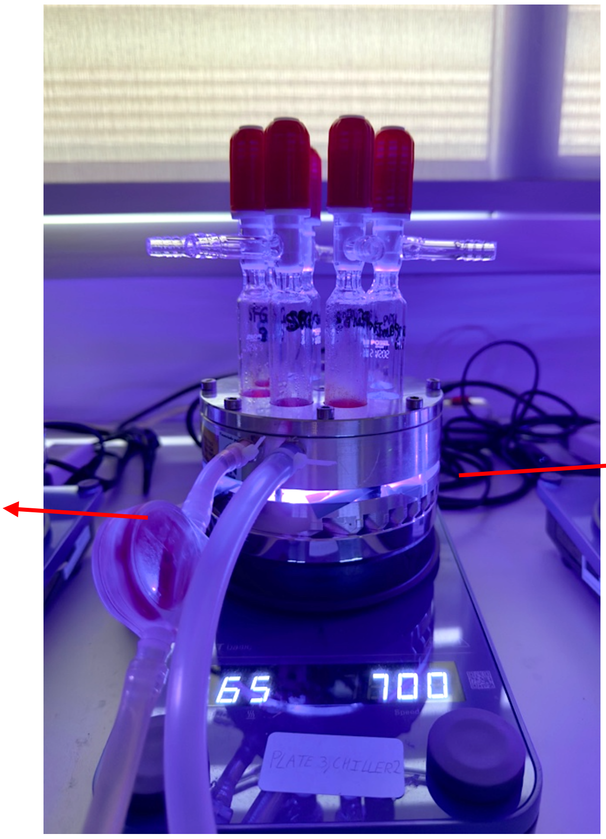

Light is irradiated from the bottom of the tube.

Fig. S1. Reaction setup. Photoreactor consisting of 8 reaction-vial slots equipped with independent 405 nm LEDs.

Light source

Reactions were irradiated with High Efficiency blue LED Emitter LED Engin LZ1-00UB00 on Standard Star MCPCB located in each slot of the photoreactor. Radiant flux of the LED at the working temperature is 1050 mW. The emission spectrum of the light source, which were recorded on a Jobin Yvon Fluorolog 3-22 spectrofluorometer, displays a peak wavelength at 405 nm (Fig. S2).

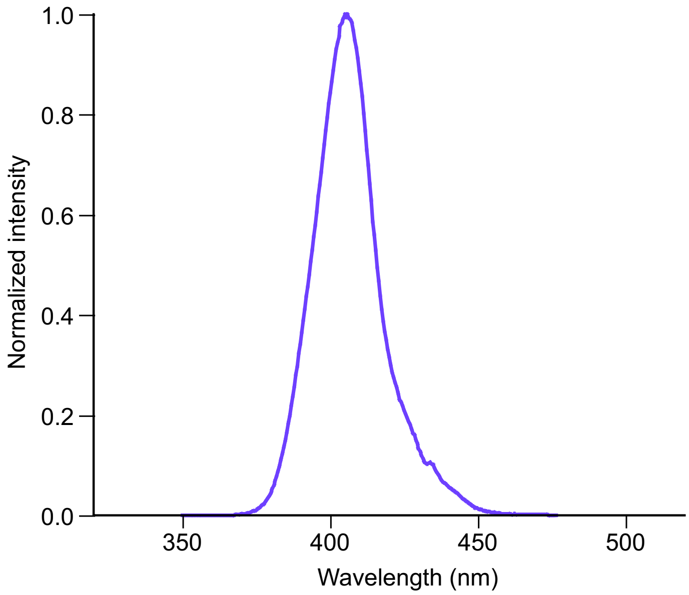

Fig. S2. Emission spectrum of the light source utilized in the photodecarboxylation reactions.

OPTIMIZATION OF THE REACTION CONDITIONS

General procedure for screening and control reactions

An oven-dried 12 mL Schlenk tube with a screw cap containing a stirring bar was charged with NaO_2CCF_2Cl (274.4 mg, 1.8 mmol, 6.0 equiv), $Fe(OTf)_2$ (10.6 mg, 0.03 mmol, 10 mol%), 4,4'-dimethoxy-2,2'-bipyridine **L1** (6.5 mg, 0.03 mmol, 10 mol%), $K_2S_2O_8$ (243.3 mg, 0.9 mmol, 3.0 equiv) and the corresponding (hetero)aromatic substrate (0.30 mmol, 1.0 equiv). The tube was evacuated and back-filled with nitrogen, and this procedure was repeated three times. Against a positive N_2 flow, the degassed MeCN (5 mL) was added via a syringe. The tube was sealed, placed on the photoreactor, and irradiated with 405 nm LEDs at 25 ºC. After 24 hours, hexafluorobenzene (35 μL, 0.30 mmol) was added and the reaction was quenched by the addition of a saturated aqueous solution of $NaHCO_3$ (2 mL) and diluted with EtOAc (10 mL). An aliquot of the organic phase was analyzed by ^{19}F NMR spectroscopy using hexafluorobenzene as an internal standard to determine the yield of **1a**. ^{19}F NMR spectra were recorded with D1 = 7 s to ensure reproducibility in the quantification of crude NMR yields.

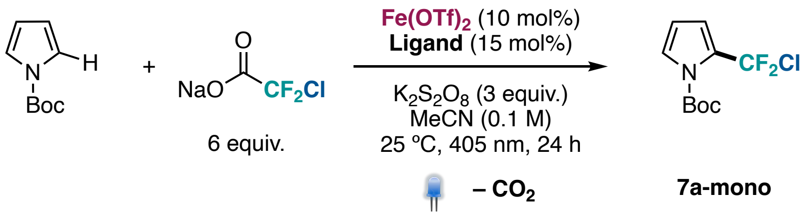

Entry	*Ligand*	*Yield (%)* ***7a-mono***
**1**	**L1**	39
2	**L2**	23
3	**L3**	10
4	**L4**	8
5	**L5**	31

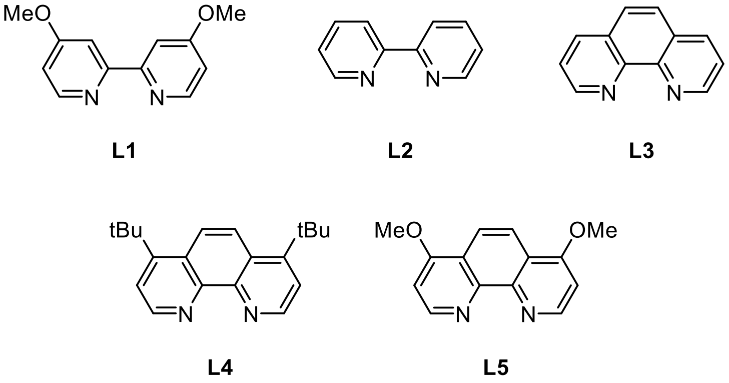

Table S1. Screening of ligands. Reaction conditions: tert-butyl-1*H*-pyrrole-1-carboxylate (0.3 mmol), NaO_2CCF_2Cl (6 equiv.), $Fe(OTf)_2$ (10 mol %), **ligand** (15 mol %), $K_2S_2O_8$ (3 equiv.), acetonitrile (0.1 M), 405 nm irradiation, 25 °C, 24h. Yields determined by ^{19}F NMR using hexafluorobenzene as internal standard.

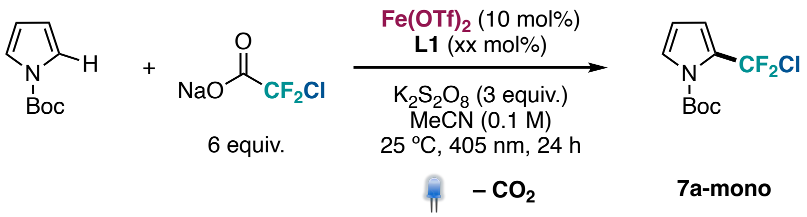

Entry	***L1*** *(mol%)*	*Yield (%)****7a-mono***
1	0	13
2	5	43
3	10	54
4	15	39
5	20	22

Table S2. Loading of L1. Reaction conditions: tert-butyl-1*H*-pyrrole-1-carboxylate (0.3 mmol), NaO_2CCF_2Cl (6 equiv.), $Fe(OTf)_2$ (10 mol %), **L1** (xx mol%), $K_2S_2O_8$ (3 equiv.), acetonitrile (0.1 M), 405 nm irradiation, 25 °C, 24h. Yields determined by ^{19}F NMR using hexafluorobenzene as internal standard.

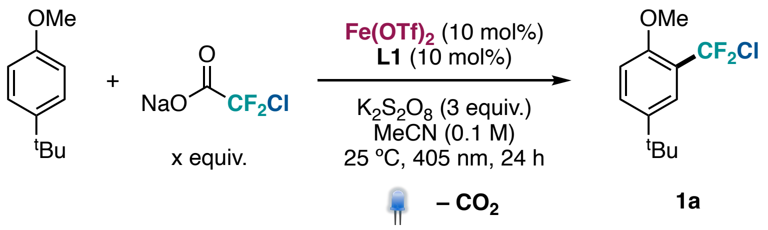

Entry	NaO_2CCF_2Cl *(equiv.)*	*Yield (%) of **1a***
1	1	42
2	2	70
3	4	80
4	6	78

Table S3. Loading of sodium chlorodifluoroacetate. Reaction conditions: 4-tert-butylanisole (0.3 mmol), NaO_2CCF_2Cl (xx equiv.), $Fe(OTf)_2$ (10 mol %), **L1** (10 mol%), $K_2S_2O_8$ (3 equiv.), acetonitrile (0.1 M), 405 nm irradiation, 25 °C, 24h. Yields determined by ^{19}F NMR using hexafluorobenzene as internal standard.

Although the use of 4 equivalents of NaO_2CCF_2Cl afforded a very good yield for the formation of product **1a**, the reaction conditions using 6 equivalents of NaO_2CCF_2Cl are generally more effective and reproducible for the remaining substrates in the reaction scope.

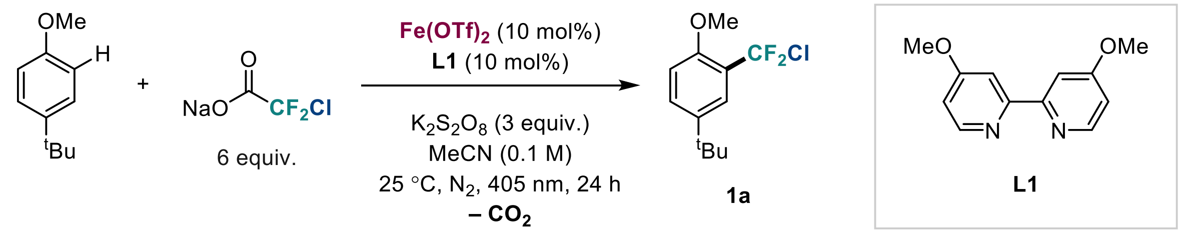

Entry	*Deviation from conditions*	*Yield (%) of **1a***
1	none	78
2	without $Fe(OTf)_2$	<1
3	without $K_2S_2O_8$	<1
4	in the dark	<1
5	under air	<5
6	NaO_2CCF_2Br as reagent	61[b]

Table S4. Control experiments. Reaction conditions: 4-tert-butylanisole (0.3 mmol), NaO_2CCF_2Cl (6 equiv.), $Fe(OTf)_2$ (10 mol %), **L1** (10 mol %), $K_2S_2O_8$ (3 equiv.), acetonitrile (0.1 M), 405 nm irradiation, 25 °C, 24h. Yields determined by ^{19}F NMR using hexafluorobenzene as internal standard. [a]Yield of the corresponding bromodifluoromethylated compound **1b** using NaO_2CCF_2Br instead of NaO_2CCF_2Cl, determined by ^{19}F NMR using hexafluorobenzene as internal standard.

GENERAL REACTION PROCEDURE FOR CHLORODIFLUOROMETHYLATION REACTIONS

General procedure A

An oven-dried 12 mL Schlenk tube with a screw cap containing a stirring bar was charged with NaO_2CCF_2Cl (458 mg, 3.0 mmol, 6.0 equiv), $Fe(OTf)_2$ (17.8 mg, 0.05 mmol, 10 mol%), 4,4'-dimethoxy-2,2'-bipyridine **L1** (10.6 mg, 0.05 mmol, 10 mol%), $K_2S_2O_8$ (406 mg, 1.5 mmol, 3.0 equiv) and the corresponding (hetero)aromatic substrate (0.50 mmol, 1.0 equiv). The tube was evacuated and back-filled with nitrogen, and this procedure was repeated three times. Against a positive N_2 flow, the degassed MeCN (5 mL) was added via a syringe. The tube was sealed, placed on the photoreactor, and irradiated with 405 nm LEDs at 25 ºC. After 24 hours, the reaction was quenched with saturated aqueous $NaHCO_3$ solution (2 mL), diluted with EtOAc (10 mL) and transferred to a separating funnel. The two phases were separated, the organic layer was washed with saturated aqueous $NaHCO_3$ solution (2x15mL), then brine (15 mL) and dried over Na_2SO_4. After removal of the solvent under reduced pressure, the crude mixture was purified by flash column chromatography on a silica gel column to afford the chlorodifluoromethylated product.

General procedure B (for volatile products)

An oven-dried 12 mL Schlenk tube with a screw cap containing a stirring bar was charged with NaO_2CCF_2Cl (458 mg, 3.0 mmol, 6.0 equiv), $Fe(OTf)_2$ (17.8 mg, 0.05 mmol, 10 mol%), 4,4'-dimethoxy-2,2'-bipyridine **L1** (10.6 mg, 0.05 mmol, 10 mol%), $K_2S_2O_8$ (406 mg, 1.5 mmol, 3.0 equiv) and the corresponding (hetero)aromatic substrate (0.50 mmol, 1.0 equiv). The tube was evacuated and back-filled with nitrogen, and this procedure was repeated three times. Against a positive N_2 flow, the degassed MeCN (5 mL) was added via a syringe. The tube was sealed, placed on the photoreactor, and irradiated with 405 nm LEDs at 25 ºC. After 24 hours, the reaction was quenched with saturated aqueous $NaHCO_3$ solution (2 mL) and diluted with Et_2O (10 mL). The characterization of volatile compounds was determined by ^{19}F NMR of the crude reaction mixture using hexafluorobenzene as internal standard. ^{19}F NMR spectra were recorded with D1 = 7 s to ensure reproducibility in the quantification of crude NMR yields. The identity of the products was also confirmed by HRMS analysis.

General procedure C

An oven-dried 12 mL Schlenk tube with a screw cap containing a stirring bar was charged with NaO_2CCF_2Cl (458 mg, 3.0 mmol, 6.0 equiv), $Fe(OTf)_2$ (17.8 mg, 0.05 mmol, 10 mol%), 4,4'-dimethoxy-2,2'-bipyridine **L1** (10.6 mg, 0.05 mmol, 10 mol%), $K_2S_2O_8$ (406 mg, 1.5 mmol, 3.0 equiv) and the corresponding (hetero)aromatic substrate (0.50 mmol, 1.0 equiv). The tube was evacuated and back-filled with nitrogen, and this procedure was repeated three times. Against a positive N_2 flow, the degassed MeCN (5 mL) was added via a syringe. The tube was sealed, placed on the photoreactor, and irradiated with 405 nm LEDs at 25 ºC. After 24 hours, the reaction was quenched with saturated aqueous $NaHCO_3$ solution (2 mL) and diluted with Et_2O (10 mL) and transferred to a separating funnel. The two phases were separated, the organic layer was washed with saturated aqueous $NaHCO_3$ solution (2x15mL), then brine (15 mL) and dried over Na_2SO_4 anhydrous. After removal of the solvent under reduced pressure, the crude mixture was purified by flash column chromatography on basic alumina to afford the chlorodifluoromethylated product.

SUBSTRATE SCOPE OF THE CHLORODIFLUOROMETHYLATION OF (HETERO)AROMATIC C–H BONDS

4-(Tert-butyl)-1-methoxy-2-(chlorodifluoromethyl)benzene (1a)

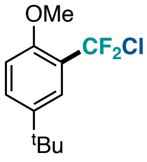

1a

Prepared following the general procedure A and starting from 4-tert-butylanisole (82.2 mg, 0.5 mmol). The crude mixture was purified by flash column chromatography on a silica gel column using a mixture of hexane/EtOAc (9:1) to provide **1a** as a colourless oil (79.9 mg, 64% yield).

Rf = 0.82 (Hex/EtOAc 6:1 (v/v)).

^{1}H NMR (400 MHz, $CDCl_3$): δ 7.56 (d, *J* = 2.5 Hz, 1H), 7.50 (dd, *J* = 8.7,2.5 Hz, 1H), 6.96 (d, *J* = 8.7 Hz, 1H), 3.92 (s, 3H), 1.33 (s, 9H) ppm.

^{13}C NMR (101 MHz, $CDCl_3$): δ 154.6, 143.0, 129.8, 125.5 (t, *J* = 291.0 Hz), 123.7 (t, *J* = 24.6 Hz), 122.6 (t, *J* = 7.0 Hz), 112.2, 56.2, 34.4, 31.5 ppm.

^{19}F NMR (376 MHz, $CDCl_3$): δ –48.83 ppm.

HRMS (ESI, *m/z*) calcd. For $C_{12}H_{15}ClF_2O$ [M+]: 248.0779; found 248.0772.

Spectroscopic data is in agreement with previously reported literature data.[1]

1,4-Dimethoxy-2-(chlorodifluoromethyl)benzene (2a)

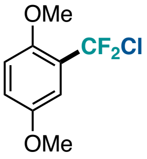

2a

Starting from 1,4-dimethoxybenzene (69.1 mg, 0.5 mmol) and following the general procedure B to provide **2a** (69%, ^{19}F NMR yield). Isolation of the product could not be performed effectively due to volatility.

^{19}F NMR (376 MHz, $CDCl_3$): δ – 49.69 ppm.

HRMS (ESI, *m/z*) calcd. For $C_9H_9ClF_2O_2$ [M+]: 222.0259; found 222.0261.

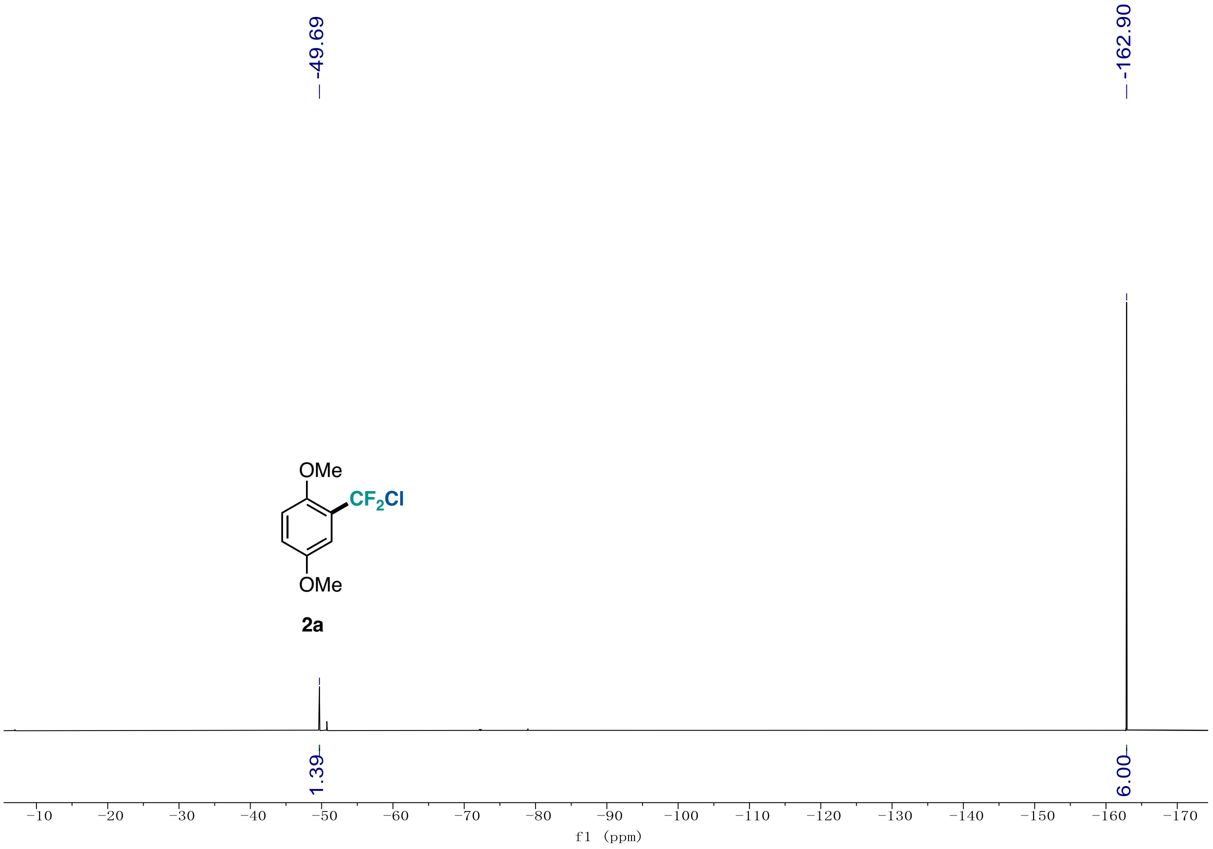

Fig. S3. ^{19}F NMR spectrum of the chlorodifluoromethylation of 1,4-dimethoxybenzene, showing the formation of **2a**. Yield determined using hexafluorobenzene as internal standard.

1-(Chlorodifluoromethyl)-2,3-dimethoxy-5-methylbenzene (3a)

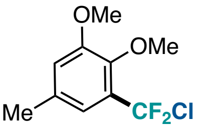

3a

Prepared following the general procedure A and starting from 1,2-dimethoxy-4-methylbenzene (76.1 mg, 0.5 mmol). The crude mixture was purified by flash column chromatography on basic alumina column using a mixture of hexane/Et_2O (6:1) to provide **3a** as a white solid (71 mg, 61% yield).

Rf = 0.57 (Hex/Et_2O 6:1 (v/v)).

^{1}H NMR (400 MHz, $CDCl_3$): δ 7.60 (s, 1H), 6.73 (s, 1H), 3.94 (s, 3H), 3.91 (s, 3H), 2.63 (s, 3H) ppm.

^{19}F NMR (376 MHz, $CDCl_3$): δ – 46.00 ppm.

HRMS (ESI, *m*/*z*) calcd. For $C_{10}H_{11}ClF_2O_2$ [M+]: 236.0416; found 236.0411.
^{13}C NMR spectra of this product could not be recorded due to its instability.

5-(Chlorodifluoromethyl)-2,3-dihydrobenzo[b][1,4]dioxine and 5,8-bis(chlorodifluoromethyl)-2,3-dihydrobenzo[b][1,4]dioxine (4a)

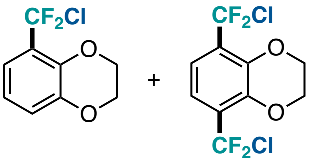

4a-mono **4a-bis**

Starting from 2,3-dihydrobenzo[*b*][1,4]dioxine (60 µL, 0.5 mmol) and following the general procedure B to provide the products (ratio 64:36) **4a-mono** (50%, ^{19}F NMR yield) and **4a-bis** (28%, ^{19}F NMR yield). Isolation of the products could not be performed effectively due to volatility.

Data for the major isomer **4a-mono**:
^{19}F NMR (376 MHz, CDCl$_3$): δ –47.25 ppm.
HRMS (ESI, *m*/*z*) calcd. For $C_9H_7ClF_2O_2$ [M+]: 220.0103; found 220.0098.
Spectroscopic data is in agreement with previously reported literature data.[2]

Data for the minor isomer **4a-bis**:
^{19}F NMR (376 MHz, CDCl$_3$): δ –49.07 ppm.
HRMS (ESI, *m*/*z*) calcd. For $C_{10}H_6Cl_2F_4O_2$ [M+]: 303.9681; found 303.9678.

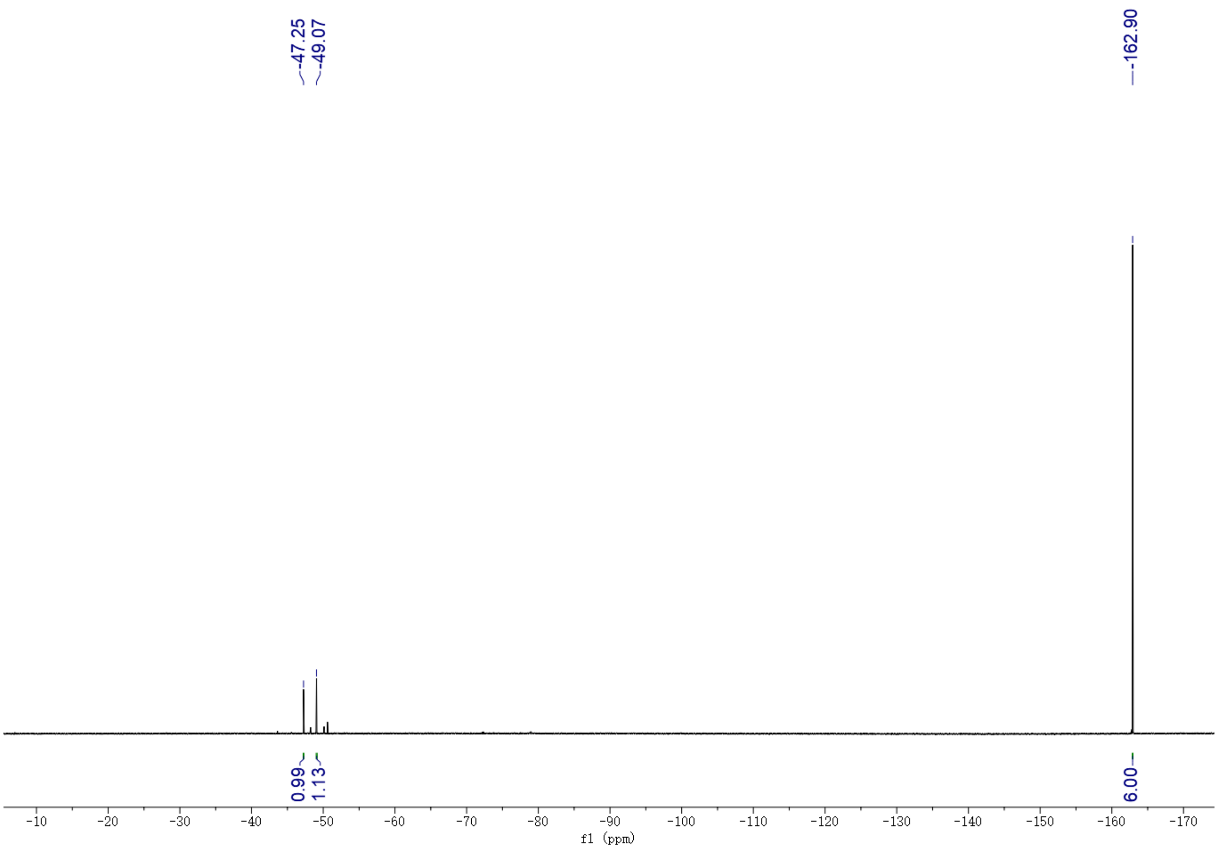

Fig. S4. ^{19}F NMR spectrum of the chlorodifluoromethylation of 2,3-dihydrobenzo[b][1,4]dioxine, showing the formation of **4a-mono** and **4a-bis**. Yields determined using hexafluorobenzene as internal standard.

1-(2-(Chlorodifluoromethyl)-3,4,5-trimethoxyphenyl)ethan-1-one (5a)

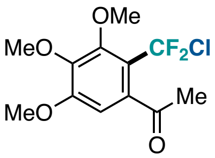

5a

Prepared following the general procedure A and starting from 1-(3,4,5-trimethoxyphenyl)ethan-1-one (105.1 mg, 0.5 mmol). The crude mixture was purified by flash column chromatography on a silica gel column using a mixture of hexane/EtOAc (3:1) to provide **5a** as a white solid (132 mg, 89% yield).

Rf = 0.45 (Hex/EtOAc 3:1 (v/v)).

^{1}H NMR (400 MHz, $CDCl_3$): δ 6.43 (s, 1H), 3.98 (s, 3H), 3.87 (s, 3H), 3.87 (s, 3H), 2.48 (s, 3H) ppm.

^{13}C NMR (101 MHz, $CDCl_3$): δ 202.6, 156.1, 152.0, 143.5, 136.2, 124.7 (t, J = 289.5 Hz), 118.7 (t, J = 25.1 Hz), 104.2, 61.7, 60.9, 56.3, 31.4 ppm.

^{19}F NMR (376 MHz, $CDCl_3$): δ –41.87 ppm.

HRMS (ESI, *m*/*z*) calcd. For $C_{12}H_{13}ClF_2O_4$ [M+]: 294.0470; found 294.0467.

5-(Chlorodifluoromethyl)-2,3-dihydrothieno[3,4-b][1,4]dioxine (6a)

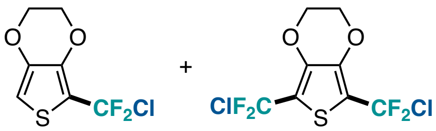

6a-mono **6a-bis**

Prepared following the general procedure B and starting from 3,4-ethylenedioxythiophene (55 μL, 0.5 mmol) to provide the products (ratio 88:12) **6a-mono** (36%, ^{19}F NMR yield) and **6a-bis** (5%, ^{19}F NMR yield). Isolation of the product could not be performed effectively due to its instability.

Data for the major isomer **6a-mono**:

^{19}F NMR (376 MHz, $CDCl_3$): δ –39.88 ppm.

HRMS (ESI, m/z) calcd. For $C_7H_5ClF_2O_2S$ [M+]: 225.9667; found 225.9661.

Data for the minor isomer **6a-bis**:

^{19}F NMR (376 MHz, $CDCl_3$): δ –41.45 ppm.

HRMS (ESI, m/z) calcd. For $C_8H_4Cl_2F_4O_2S$ [M+]: 309,9245; found 309.9270.

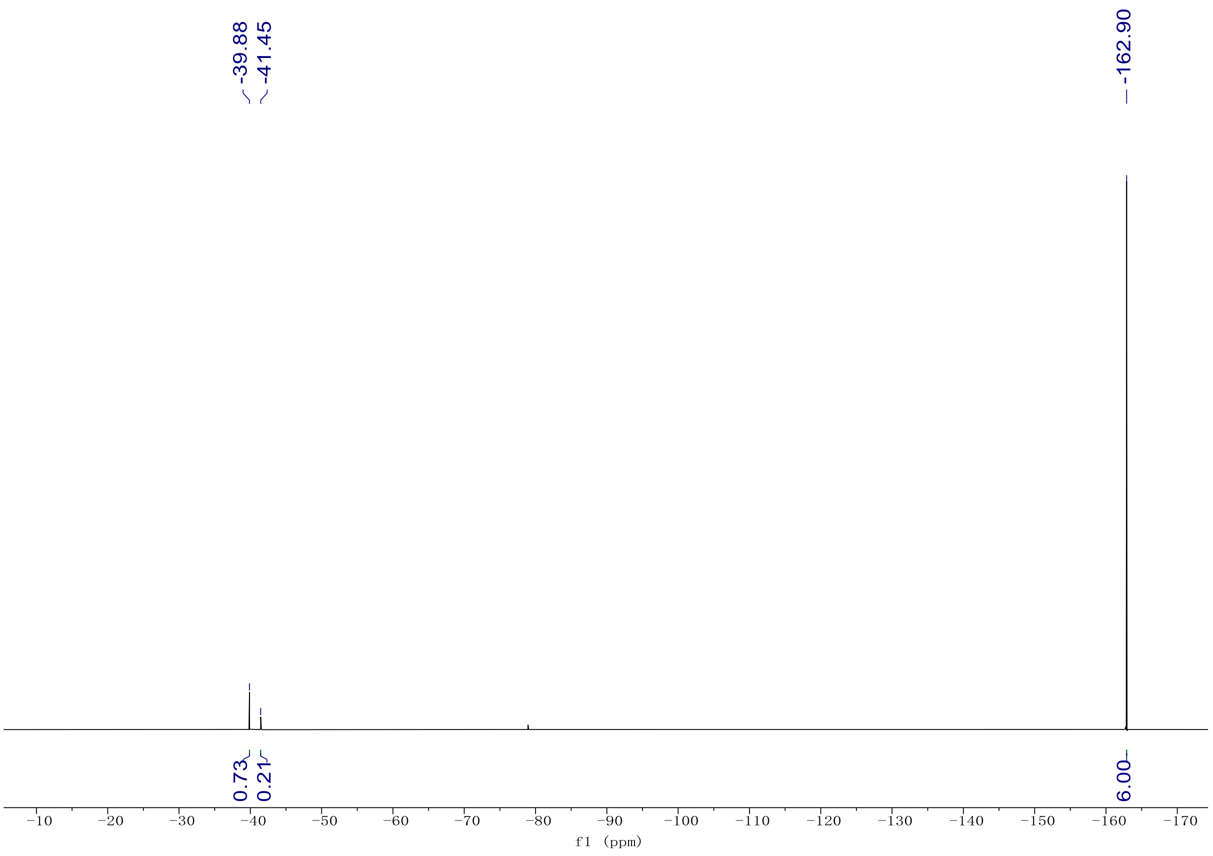

Fig. S5. ^{19}F NMR spectrum of the chlorodifluoromethylation of 3,4-ethylenedioxythiophene, showing the formation of **6a-mono** and **6a-bis**. Yields determined using hexafluorobenzene as internal standard.

Tert-butyl 2-(chlorodifluoromethyl)-1H-pyrrole-1-carboxylate and tert-butyl 2,5-bis(chlorodifluoromethyl)-1H-pyrrole-1-carboxylate) (7a)

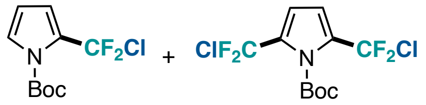

7a-mono **7a-bis**

Prepared following the general procedure C and starting from tert-butyl-1*H*-pyrrole-1-carboxylate (84 µL, 0.5 mmol). The crude mixture was purified by flash column chromatography on a basic alumina column using a mixture of hexane/Et_2O (6:1) to provide a mixture of isomers (ratio 85:15) **7a-mono** and **7a-bis** as a light-orange oil (67 mg, 50%).

Data for the major isomer **7a-mono**:

^{1}H NMR (600 MHz, $CDCl_3$): δ 7.43 (dd, *J* = 3.4, 1.9 Hz, 1H), 6.71 (dd, *J* = 3.6, 2.0 Hz, 1H), 6.16 (t, *J* = 3.4 Hz, 1H), 1.62 (s, 9H) ppm.

^{13}C NMR (151 MHz, CDCl$_3$): δ 147.6, 126.9 (t, J = 34.6 Hz), 126.3, 122.7 (t, J = 284.2 Hz), 117.2 (t, J = 5.4 Hz), 109.5, 85.9, 27.9 ppm.
^{19}F NMR (376 MHz, CDCl$_3$): δ –43.76 ppm.
HRMS (ESI, *m/z*) calcd. For $C_{10}H_{12}ClF_2NO_2$ [M+]: 251.0519; found 251.0513.

Data for the minor isomer **7a-bis**:

^{1}H NMR (600 MHz, CDCl$_3$): δ 6.63 (s, 2H), 1.64 (s, 9H) ppm.
^{13}C NMR (151 MHz, CDCl$_3$): δ 146.7, 130.6 (t, J = 34.5 Hz), 121.06 (t, J = 285.1 Hz), 112.82 (t, J = 4.5 Hz), 88.2, 27.4 ppm.
^{19}F NMR (376 MHz, CDCl$_3$): δ –43.91 ppm.
Spectroscopic data is in agreement with previously reported literature data.[1]

1-(5-(Chlorodifluoromethyl)-1-methyl-1H-pyrrol-2-yl)ethan-1-one (8a)

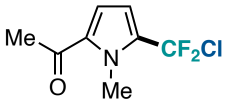

8a

Prepared following the general procedure A and starting from 1-(1-methyl-1*H*-pyrrol-2-yl)ethan-1-one (60 µL, 0.5 mmol). The crude mixture was purified by flash column chromatography on a silica gel column using a mixture of hexane/Et_2O (7:1) to provide **8a** as a colourless oil (75 mg, 72% yield).

Rf = 0.46 (Hex/Et_2O 7:1 (v/v)).
^{1}H NMR (400 MHz, CDCl$_3$): δ 6.87 (d, J = 4.3 Hz, 1H), 6.51 (d, J = 4.4 Hz, 1H), 4.05 (s, 3H), 2.47 (s, 3H) ppm.
^{13}C NMR (101 MHz, CDCl$_3$): δ 189.9, 133.9, 132.7 (t, J = 32.0 Hz), 121.9 (t, J = 285.4 Hz), 117.5, 109.7 (t, J = 4.1 Hz), 34.6, 28.1 ppm.
^{19}F NMR (376 MHz, CDCl$_3$): δ –46.35 ppm.
HRMS (ESI, *m/z*) calcd. For $C_8H_8ClF_2NO$ [M+]: 207.0262; found 207.0276.
Spectroscopic data is in agreement with previously reported literature data.[3]

Methyl 1-methyl-5-(chlorodifluoromethyl)-1H-pyrrole-2-carboxylate (9a)

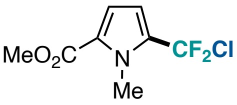

9a

Prepared following the general procedure A and starting from methyl 1-methyl-1*H*-pyrrole-2-carboxylate (60 µL, 0.5 mmol). The crude mixture was purified by flash column chromatography on a silica gel column using a mixture of hexane/Et_2O (7:1) to provide **9a** as a white solid (88 mg, 79% yield).

Rf = 0.51 (Hex/Et_2O 7:1 (v/v)).

^{1}H NMR (400 MHz, $CDCl_3$): δ 6.86 (d, *J* = 4.2 Hz, 1H), 6.50 (d, *J* = 4.2 Hz, 1H), 4.06 (s, 3H), 3.84 (s, 3H) ppm.

^{13}C NMR (101 MHz, $CDCl_3$): δ 161.9, 131.9 (t, *J* = 32.2 Hz), 126.5, 121.9 (t, *J* = 285.1 Hz), 116.0, 109.9 (t, *J* = 4.1 Hz), 51.7, 33.9 ppm.

^{19}F NMR (376 MHz, $CDCl_3$): δ –45.93 ppm.

HRMS (ESI, *m*/*z*) calcd. For $C_8H_8ClF_2NO_2$ [M+]: 223.0212; found 223.0205.

1-Methyl-3-(chlorodifluoromethyl)pyridin-2(1H)-one (10a)

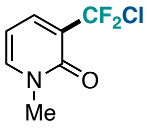

10a

Prepared following the general procedure A and starting from *N*-methyl-2-pyridone (50 µL, 0.5 mmol). The crude mixture was purified by flash column chromatography on a silica gel column eluting with EtOAc to provide **10a** as a yellow solid (67 mg, 70% yield).

Rf = 0.53 (EtOAc).

^{1}H NMR (400 MHz, $CDCl_3$): δ 7.70 (dd, *J* = 7.2, 2.1 Hz, 1H), 7.51 (dd, *J* = 6.7, 2.1 Hz, 1H), 6.23 (t, *J* = 7.0 Hz, 1H), 3.61 (s, 3H) ppm.

^{13}C NMR (101 MHz, $CDCl_3$): δ 158.5, 142.0, 136.9 (t, *J* = 6.7 Hz), 125.4 (t, *J* = 25.2 Hz), 124.3 (t, *J* = 288.5 Hz), 104.0, 37.9 ppm.

^{19}F NMR (376 MHz, $CDCl_3$): δ – 53.36 ppm.

HRMS (ESI, *m/z*) calcd. For $C_7H_6ClF_2NO$ $[M+H]^+$: 194.0179; found 194.0183.
Spectroscopic data is in agreement with previously reported literature data.[1]

5-(Chlorodifluoromethyl)-4,6-dimethoxypyrimidine (11a)

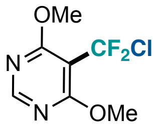

11a

Prepared following the general procedure C and starting from 4,6-dimethoxypyrimidine (70.1 mg, 0.5 mmol). The crude mixture was purified by flash column chromatography on basic alumina column eluting using a mixture of hexane/Et_2O (6:1) to provide **11a** a white solid (40 mg, 35% yield).

^{1}H NMR (400 MHz, $CDCl_3$): δ 8.44 (s, 1H), 4.05 (s, 6H) ppm.
^{13}C NMR (75 MHz, $CDCl_3$): δ 166.7, 158.6, 124.0 (t, J = 292.5 Hz), 101.22 (t, J = 27.5 Hz), 55.2 ppm.
^{19}F NMR (376 MHz, $CDCl_3$): δ – 44.86 ppm.
HRMS (ESI, *m/z*) calcd. For $C_7H_7ClF_2N_2O_2$ [M+]: 224.0164; found 224.0141.
Spectroscopic data is in agreement with previously reported literature data.[4]

3-(Chlorodifluoromethyl)-2,6-dimethoxypyridine and 3,5-bis(chlorodifluoromethyl)-2,6-dimethoxypyridine (12a)

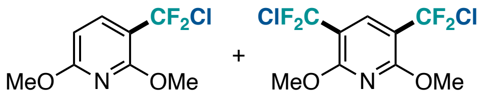

12a-mono **12a-bis**

Prepared following the general procedure C and starting from 2,6-dimethoxypyridine (65 µL, 0.5 mmol). The crude mixture was purified by flash column chromatography on basic alumina column using a mixture of hexane/Et_2O (6:1) to provide a mixture of isomers (ratio 62:38) **12a-mono** and **12a-bis** as a colourless oil (87 mg, 70% yield).

Data for the major isomer **12a-mono**:

^{1}H NMR (600 MHz, $CDCl_3$): δ 7.71 (d, J = 8.3 Hz, 1H), 6.32 (d, J = 8.3Hz, 1H), 4.05 (s, 3H), 3.96 (s, 3H) ppm.

^{13}C NMR (151 MHz, CDCl$_3$): δ 165.0, 159.6, 137.7 (t, J = 5.9 Hz), 125.2 (t, J = 288.2 Hz), 110.4 (t, J = 27.3Hz), 100.9, 54.1, 54.0 ppm.
^{19}F NMR (376 MHz, CDCl$_3$): δ –47.97 ppm.
HRMS (ESI, *m*/*z*) calcd. For $C_8H_8ClF_2NO_2$ [M+]: 223.0212; found 223.0211.

Data for the minor isomer **12a-bis**:

^{1}H NMR (600 MHz, CDCl$_3$): δ 7.96 (s, 1H), 4.11 (s, 6H) ppm.
^{13}C NMR (151 MHz, CDCl$_3$): δ 161.0, 134.8 (t, J = 6.1 Hz), 124.2 (t, J = 288.6 Hz), 109.8 (t, J = 28.6Hz), 54.9 ppm.
^{19}F NMR (376 MHz, CDCl$_3$): δ –48.78 ppm.
HRMS (ESI, *m*/*z*) calcd. For $C_9H_7Cl_2F_4NO_2$ [M+]: 306.9790; found 306.9783.

1-(2-(Chlorodifluoromethyl)-1H-indol-3-yl)ethan-1-one (13a)

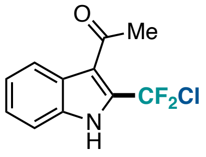

13a

Prepared following the general procedure A and starting from 1-(1*H*-indol-3-yl)ethan-1-one (79.6 mg, 0.5 mmol). The crude mixture was purified by flash column chromatography on a silica gel column using a mixture of hexane/EtOAc (10:1) to provide **13a** as a white solid (28.8 mg, 24% yield).

Rf = 0.36 (Hex/EtOAc 6:1 (v/v)).
^{1}H NMR (400 MHz, CDCl$_3$): δ 9.31 (br s, 1H), 8.15 (d, J = 7.2 Hz, 1H), 7.52 – 7.49 (m, 1H), 7.42 – 7.32 (m, 2H), 2.76 (s, 3H) ppm.
^{13}C NMR (75 MHz, CDCl$_3$): δ 194.4, 133.9, 132.4 (t, J = 32.0 Hz), 126.4, 125.6, 123.6, 122.6, 122.1 (t, J = 287.4 Hz), 116.2, 112.3, 31.6 (t, J = 2.5 Hz) ppm.
^{19}F NMR (376 MHz, CDCl$_3$): δ – 45.91 ppm.
HRMS (ESI, *m*/*z*) calcd. For $C_{11}H_8ClF_2NO$ [M+]: 243.0262; found 243.0259.

Methyl 2-(chlorodifluoromethyl)-1H-indole-3-carboxylate (14a-mono)

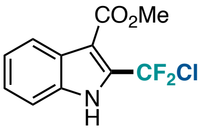

14a-mono

Prepared following the general procedure A and starting from methyl 1*H*-indole-3-carboxylate (87.6 mg, 0.5 mmol). The crude mixture was purified by flash column chromatography on a silica gel column using a mixture of hexane/EtOAc (9:1) to provide **14a-mono** as a white solid (42 mg, 33% yield).

Rf = 0.60 (Hex/EtOAc 6:1 (v/v)).

^{1}H NMR (400 MHz, CDCl$_3$): δ 9.29 (br s, 1H), 8.24 (d, *J* = 7.9 Hz, 1H), 7.48 (d, *J* = 8.0 Hz, 1H), 7.40 – 7.31 (m, 2H), 4.0 (s, 3H) ppm.

^{13}C NMR (75 MHz, CDCl$_3$): δ 163.8, 134.0 (t, *J* = 32.1 Hz), 133.6, 126.7, 125.6, 123.3, 123.0, 121.7 (t, *J* = 287.7 Hz), 112.1, 107.0, 51.8 ppm.

^{19}F NMR (376 MHz, CDCl$_3$): δ – 41.87 ppm.

HRMS (ESI, *m/z*) calcd. For $C_{11}H_8ClF_2NO_2$ [M+]: 259.0212; found 259.0211.

Spectroscopic data is in agreement with previously reported literature data.[5]

Methyl 2,4-bis(chlorodifluoromethyl)-1H-indole-3-carboxylate (14a-bis)

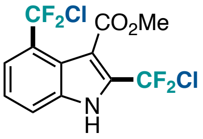

14a-bis

Prepared following the general procedure A and starting from methyl 1*H*-indole-3-carboxylate (87.6 mg, 0.5 mmol). The crude mixture was purified by flash column chromatography on a silica gel column using a mixture of hexane/EtOAc (9:1) to provide **14a-bis** as a white solid (38.7 mg, 22% yield).

Rf = 0.54 (Hex/EtOAc 6:1 (v/v)).

^{1}H NMR (400 MHz, CDCl$_3$): δ 9.63i (br s, 1H), 7.59 (t, *J* = 8.0 Hz, 2H), 7.40 (t, *J* = 7.9 Hz, 1H), 3.97 (s, 3H) ppm.

^{13}C NMR (101 MHz, $CDCl_3$): δ 165.5, 135.5, 131.1 (t, *J* = 32.7 Hz), 129.3 (t, *J* = 28.1 Hz), 126.1 (t, *J* = 289.6 Hz), 124.7, 121.5 (t, *J* = 287.1 Hz), 120.4 (t, *J* = 7.1 Hz), 118.9 (t, *J* = 1.9 Hz), 116.0, 109.5, 52.9 ppm.
^{19}F NMR (376 MHz, $CDCl_3$): δ –46.76, –47.03 ppm.
HRMS (ESI, *m/z*) calcd. For $C_{12}H_7Cl_2F_4NO_2$ [M+]: 342.9790; found: 342.9783.

Methyl 2-(chlorodifluoromethyl)-1-methyl-1H-indole-3-carboxylate (15a-mono)

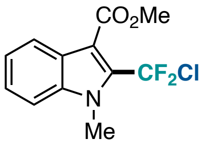

15a-mono

Prepared following the general procedure A and starting from methyl 1-methyl-1*H*-indole-3-carboxylate (94.6 mg, 0.5 mmol). The crude mixture was purified by flash column chromatography on a silica gel column using a mixture of hexane/EtOAc (9:1) to provide **15a-mono** as a yellow solid (51.4 mg, 38% yield).

Rf = 0.34 (Hex/EtOAc 9:1 (v/v)).
^{1}H NMR (300 MHz, $CDCl_3$): δ 8.06 (m, 1H), 7.42 – 7.40 (m, 2H), 7.35 – 7.28 (m, 1H), 3.97 (s, 3H), 3.94 (t, *J* = 2.0 Hz, 3H) ppm.
^{13}C NMR (75 MHz, $CDCl_3$): δ 164.1, 137.0, 133.1 (t, *J* = 30.8 Hz), 125.4, 125.3, 122.8, 122.6, 121.9 (t, *J* = 288.7 Hz), 110.2, 108.1, 52.0, 32.3 (t, *J* = 5.0 Hz) ppm.
^{19}F NMR (282 MHz, $CDCl_3$): δ –44.47 ppm.
HRMS (ESI, *m/z*) calcd. For $C_{12}H_{10}ClF_2NO_2$ [M+]: 273.0368; found 273.0364.
Spectroscopic data is in agreement with previously reported literature data.[6]

Methyl 2,4-bis(chlorodifluoromethyl)-1-methyl-1H-indole-3-carboxylate (15a-bis)

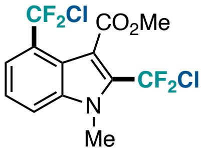

15a-bis

Prepared following the general procedure A and starting from methyl 1-methyl-1*H*-indole-3-carboxylate (94.6 mg, 0.5 mmol). The crude mixture was purified by flash column

chromatography on a silica gel column using a mixture of hexane/EtOAc (9:1) to provide **15a-bis** as a yellow solid (21.3 mg, 12% yield).

Rf = 0.12 (Hex/EtOAc 9:1 (v/v)).

^{1}H NMR (400 MHz, CDCl$_3$): δ 7.59 (d, *J* = 3.1 Hz, 1H), 7.57 (d, *J* = 3.9 Hz, 1H), 7.45 (m, 1H), 3.95 (s, 3H), 3.94 (s, 3H) ppm.

^{13}C NMR (101 MHz, CDCl$_3$): δ 165.6, 138.2, 130.9 (t, *J* = 31.3 Hz), 129.4 (t, *J* = 27.8 Hz), 126.1 (t, *J* = 288.1 Hz), 124.5, 121.6 (t, *J* = 287.8 Hz), 119.9 (t, *J* = 7.1 Hz), 117.6, 113.9, 109.7 (t, *J* = 2.5 Hz), 53.0, 31.7 (t, *J* = 3.0 Hz) ppm.

^{19}F NMR (376 MHz, CDCl$_3$): δ – 46.85, –46.94 ppm.

HRMS (ESI, *m/z*) calcd. For $C_{13}H_9Cl_2F_4NO_2$ [M+]: 356.9946; found 356.9941.

5-(Chlorodifluoromethyl)-1,3-dimethylpyrimidine-2,4(1H,3H)-dione (16a)

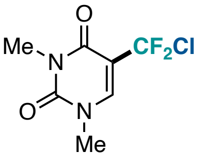

16a

Prepared following the general procedure A and starting from 1,3-dimethyluracil (70.1 mg, 0.5 mmol). The crude mixture was purified by flash column chromatography on a silica gel column using a mixture of hexane:EtOAc (1:1) to provide **16a** as a white solid (86 mg, 76% yield).

Rf = 0.70 (EtOAc).

^{1}H NMR (400 MHz, CDCl$_3$): δ 7.66 (s, 1H), 3.49 (s, 3H), 3.37 (s, 3H) ppm.

^{13}C NMR (151MHz, CDCl$_3$): δ 158.6, 151.0, 142.1 (t, *J* = 7.6 Hz), 123.3 (t, *J* = 287.1 Hz), 109.4 (t, *J* = 26.7 Hz), 37.9, 28.2 ppm.

^{19}F NMR (376 MHz, CDCl$_3$): δ – 51.06 ppm.

HRMS (ESI, *m/z*) calcd. For $C_7H_7ClF_2N_2O_2$ [M+]: 224.0164; found 224.0167.

Spectroscopic data is in agreement with previously reported literature data.[7]

1,3,7-Trimethyl-8-(chlorodifluoromethyl)-3,7-dihydro-1H-purine-2,6-dione (17a)

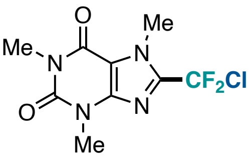

17a

Prepared following the general procedure A and starting from caffeine (97.1 mg, 0.5 mmol). The crude mixture was purified by flash column chromatography on a silica gel column eluting with hexane/EtOAc (1:4) to provide **17a** as a white solid (95 mg, 68% yield).

Rf = 0.69 (Hex/EtOAc 1:1 (v/v)).

^{1}H NMR (400 MHz, CDCl$_3$): δ 4.17 (t, *J* = 1.4 Hz, 3H), 3.58 (s, 3H), 3.41 (s, 3H) ppm.

^{13}C NMR (101 MHz, CDCl$_3$): δ 155.6, 151.5, 146.4, 142.6 (t, *J* = 32.9 Hz), 119.8 (t, *J* = 288.2 Hz), 109.7, 33.6 (t, *J* = 2.8 Hz), 30.0, 28.3 ppm.

^{19}F NMR (376 MHz, CDCl$_3$): δ – 51.01 ppm.

HRMS (ESI, *m/z*) calcd. For $C_9H_9ClF_2N_4O_2$ $[M+H]^+$: 279.0455; found 279.0460.

Spectroscopic data is in agreement with previously reported literature data.[1]

3,7-Dimethyl-1-(3-oxobutyl)-8-(chlorodifluoromethyl)-3,7-dihydro-1H-purine-2,6-dione (18a)

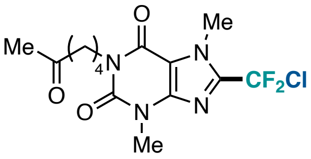

18a

Prepared following the general procedure A and starting from pentoxifylline (139.2 mg, 0.5 mmol). The crude mixture was purified by flash column chromatography on a silica gel column eluting with EtOAc to provide **18a** as a white solid (99 mg, 56% yield).

Rf = 0.72 (EtOAc).

^{1}H NMR (400 MHz, CDCl$_3$): δ 4.12 (t, *J* = 1.2 Hz, 3H), 3.96 (t, *J = 7.0* Hz, 2H), 3.52 (s, 3H), 2.45 (t, *J = 6.9* Hz, 2H), 2.09 (s, 3H), 1.60 (m, 4H) ppm.

^{13}C NMR (101 MHz, CDCl$_3$): δ 208.5, 155.4, 151.1, 146.4, 142.5 (t, *J* = 32.9 Hz), 119.7 (t, *J* = 288.2 Hz), 109.7, 43.1, 41.1, 33.5 (t, *J* = 2.7 Hz), 29.9, 29.8, 27.3, 20.9 ppm.

^{19}F NMR (376 MHz, CDCl$_3$): δ –51.07 ppm.
HRMS (ESI, *m/z*) calcd. For $C_{14}H_{17}ClF_2N_4O_3$ $[M+H]^+$: 362.0957; found 362.0961.
Spectroscopic data is in agreement with previously reported literature data.[1]

(2S,6'R)-7-Chloro-3'-(chlorodifluoromethyl)-2',4,6-trimethoxy-6'-methyl-3H-spiro[benzofuran-2,1'-cyclohexan]-2'-ene-3,4'-dione (19a)

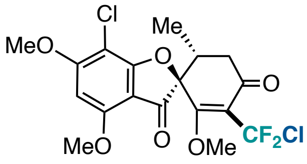

19a

Prepared following the general procedure A and starting from griseofulvin (105.8 mg, 0.3 mmol). The crude mixture was purified by flash column chromatography on a silica gel column using a mixture of hexane:EtOAc (1:1) to provide **19a** as a light-yellow solid (75 mg, 57% yield).

Rf = 0.21 (Hex/EtOAc 1:1 (v/v)).
^{1}H NMR (600 MHz, CDCl$_3$): δ 6.17 (s, 1H), 4.04 (s, 3H), 3.98 (s, 3H), 3.86 (s, 3H), 3.11 (dd, J = 16.8, 13.8 Hz, 1H), 2.86 − 2.79 (m, 1H), 2.52 (dd, J = 16.8, 4.4 Hz, 1H), 0.94 (d, J = 6.7 Hz, 3H) ppm.
^{13}C NMR (151 MHz, CDCl$_3$): δ 191.6, 191.0, 170.4, 169.1, 165.2, 158.1, 123.5 (t, J = 22.6 Hz) 123.0 (t, J = 293.5 Hz), 105.1, 97.6, 92.3, 90.2, 63.8 (t, J = 3.0 Hz), 57.3, 56.6, 40.3, 36.0, 14.2 ppm.
^{19}F NMR (376 MHz, CDCl$_3$): δ – 48.30 (d, J = 169.0 Hz), –49.30 (d, J = 168.9 Hz) ppm.
HRMS (ESI, *m/z*) calcd. For $C_{18}H_{16}Cl_2F_2O_6$ [M+]: 436.0292; found 436.0285.

3-(chlorodifluoromethyl)-5,6,7,8-tetramethoxy-2-(4-methoxyphenyl)-4H-chromen-4-one (20a)

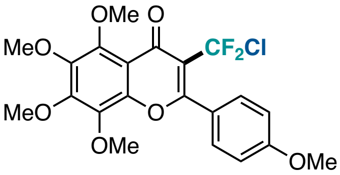

20a

Prepared following the general procedure A and starting from tangeritin (50 μL, 0.5 mmol). The crude mixture was purified by flash column chromatography on a silica gel column using a mixture of hexane:EtOAc (6:1) to provide **20a** as a white solid (98.9 mg, 74% yield).

Rf = 0.34 (Hex/EtOAc 6:1 (v/v)).

^{1}H NMR (600 MHz, CDCl$_3$): δ 7.62 (d, *J* = 8.7 Hz, 2H), 7.03 – 6.99 (m, 2H), 4.07 (s, 3H), 3.97 (s, 3H), 3.93 (s, 3H), 3.89 (s, 3H), 3.88 (s, 3H) ppm.

^{13}C NMR (151 MHz, CDCl$_3$): δ 173.3, 163.0, 162.1, 152.2, 148.6, 146.8, 144.9, 137.7, 130.8, 124.7, 124.2 (t, *J* = 290.9 Hz), 117.6 (t, *J* = 22.8 Hz), 114.3, 113.9, 62.4, 61.9, 61.8, 55.6 ppm.

^{19}F NMR (376 MHz, CDCl$_3$): δ – 45.77 ppm.

HRMS (ESI, *m/z*) calcd. For $C_{21}H_{19}ClF_2O_7$ [M+]: 456.0787; found 456.0791.

BROMODIFLUOROMETHYLATION OF (HETERO)ARENES

General procedure D

An oven-dried 12 mL Schlenk tube with a screw cap containing a stirring bar was charged with NaO_2CCF_2Br (590.7 mg, 3.0 mmol, 6.0 equiv), $Fe(OTf)_2$ (17.8 mg, 0.05 mmol, 10 mol%), 4,4'-dimethoxy-2,2'-bipyridine **L1** (10.6 mg, 0.05 mmol, 10 mol%), $K_2S_2O_8$ (406 mg, 1.5 mmol, 3.0 equiv) and the corresponding (hetero)aromatic substrate (0.50 mmol, 1.0 equiv). The tube was evacuated and back-filled with nitrogen, and this procedure was repeated three times. Against a positive N_2 flow, the degassed MeCN (5 mL) was added via a syringe. The tube was sealed, placed on the photoreactor, and irradiated with 405 nm LEDs at 25 ºC. After 24 hours, the reaction was quenched with saturated aqueous $NaHCO_3$ solution (2 mL), diluted with Et_2O (10 mL) and transferred to a separating funnel. The two phases were separated, the organic layer was washed with saturated aqueous $NaHCO_3$ solution (2x15mL), then brine (15 mL) and dried over Na_2SO_4. After removal of the solvent under reduced pressure, the crude mixture was purified by flash column chromatography on a silica gel column or basic alumina column to afford the bromodifluoromethylated product.

SUBSTRATE SCOPE OF THE BROMODIFLUOROMETHYLATION OF (HETERO)AROMATIC C–H BONDS

4-(Tert-butyl)-1-methoxy-2-(bromodifluoromethyl)benzene (1b)

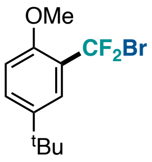

1b

Prepared following the general procedure D and starting from 4-tert-butylanisole (82.2 mg, 0.5 mmol). The crude mixture was purified by flash column chromatography on basic alumina column using a mixture of hexane/EtOAc (9:1) to provide **1b** as a colourless oil (83 mg, 56% yield).

Rf = 0.46 (Hex/EtOAc 9:1 (v/v)).

^{1}H NMR (400 MHz, $CDCl_3$): δ 7.51 – 7.47 (m, 2H), 6.94 (d, *J* = 8.5 Hz, 1H), 3.93 (s, 3H), 1.32 (s, 9H) ppm.

^{19}F NMR (376 MHz, $CDCl_3$): δ – 43.41 ppm.

HRMS (ESI, *m/z*) calcd. For $C_{12}H_{15}BrF_2O$ [M+]: 292.0274; found 292.0270.

^{13}C NMR spectra of this product could not be recorded due to its instability.

1,4-Dimethoxy-2-(bromodifluoromethyl)benzene (2b)

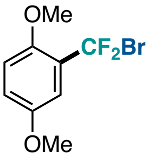

2b

Starting from 1,4-dimethoxybenzene (69.1 mg, 0.5 mmol) and following the general procedure D to provide **2b** (65%, ^{19}F NMR yield). Isolation of the product could not be performed effectively due to volatility.

^{19}F NMR (376 MHz, $CDCl_3$): δ – 44.63 ppm.

HRMS (ESI, *m/z*) calcd. For $C_9H_9F_2O_2Br$ [M+]: 265.9754; found 265.9756.

Spectroscopic data is in agreement with previously reported literature data.[8]

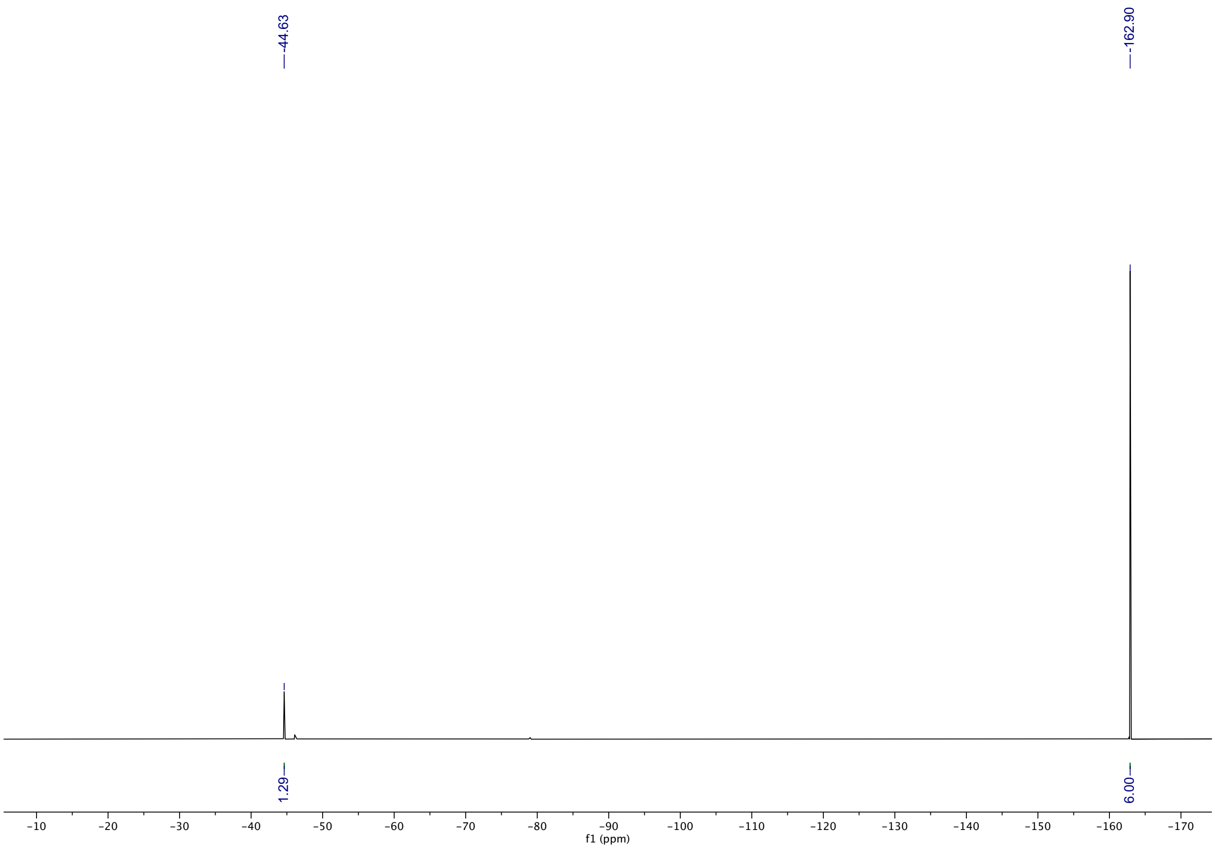

Fig. S6. ^{19}F NMR spectrum of the bromodifluoromethylation of 1,4-dimethoxybenzene, showing the formation of **2b**. Yield determined using hexafluorobenzene as internal standard.

5-(bromodifluoromethyl)-2,3-dihydrobenzo[b][1,4]dioxine and 5,8-bis(bromodifluoromethyl)-2,3-dihydrobenzo[b][1,4]dioxine (4b)

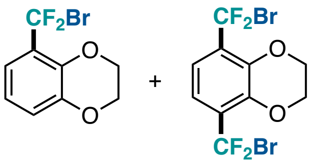

4b-mono **4b-bis**

Starting from 2,3-dihydrobenzo[*b*][1,4]dioxine (60 µL, 0.5 mmol) and following the general procedure D to provide the products (ratio 61:39) **4b-mono** (25%, ^{19}F NMR yield) and **4b-bis** (16%, ^{19}F NMR yield). Isolation of the products could not be performed effectively due to volatility.

Data for the major isomer **4b-mono**:

^{19}F NMR (376 MHz, $CDCl_3$): δ –42.10 ppm.

HRMS (ESI, *m/z*) calcd. For $C_9H_7BrF_2O_2$ [M+]: 263.9597; found 263.9590

Data for the minor isomer **4b-bis**:

^{19}F NMR (376 MHz, $CDCl_3$): δ –44.03 ppm.

HRMS (ESI, *m/z*) calcd. For $C_{10}H_6Br_2F_4O_2$ [M+]: 391.8671; found 391.8658.

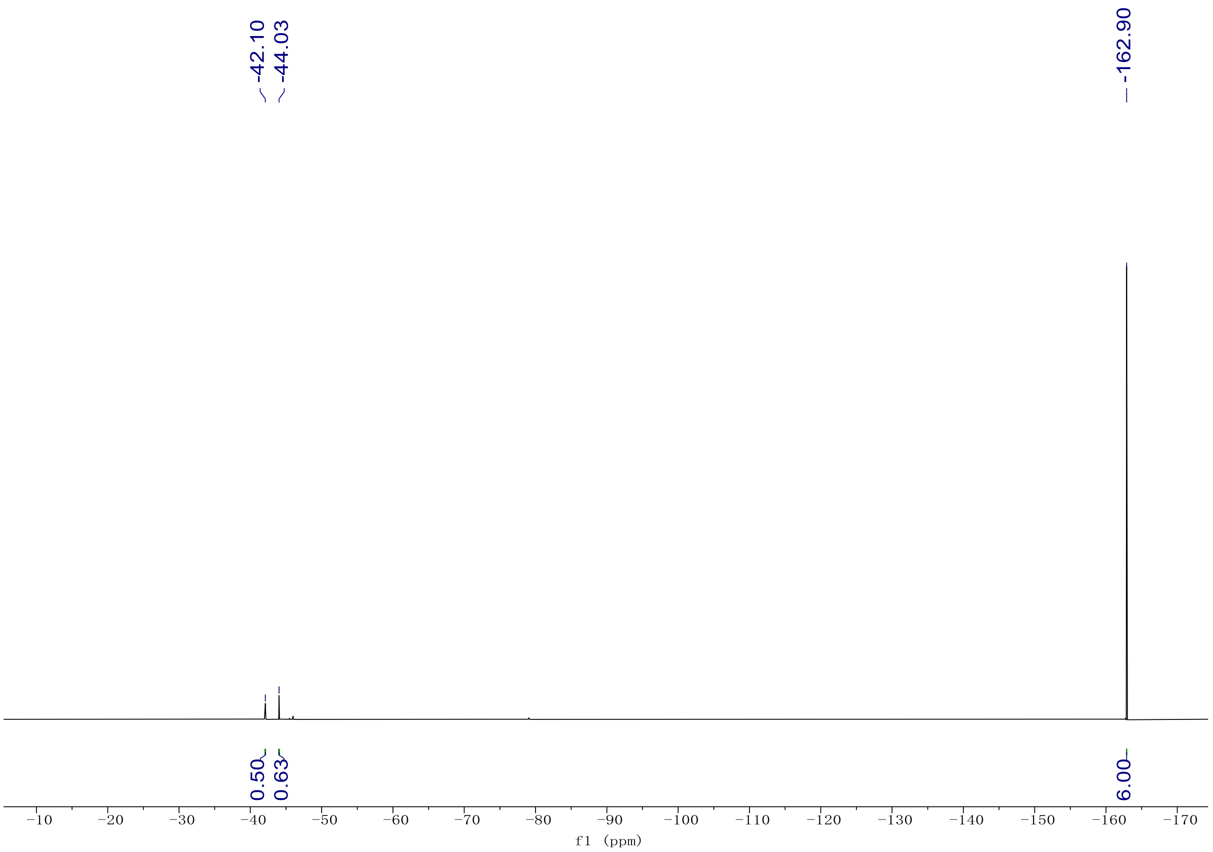

Fig. S7. ^{19}F NMR spectrum of the bromodifluoromethylation of 2,3-dihydrobenzo[*b*][1,4]dioxine, showing the formation of **4b-mono** and **4b-bis**. Yield determined using hexafluorobenzene as internal standard.

1,3,7-Trimethyl-8-(bromodifluoromethyl)-3,7-dihydro-1H-purine-2,6-dione (17b)

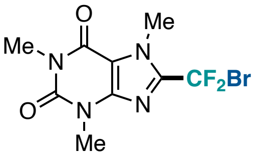

17b

Prepared following the general procedure D and starting from caffeine (97.1 mg, 0.5 mmol). The crude mixture was purified by flash column chromatography on a silica gel column eluting with EtOAc to provide **17b** as a white solid (105 mg, 67% yield).

Rf = 0.69 (Hex/EtOAc 1:1 (v/v)).

^{1}H NMR (400 MHz, $CDCl_3$): δ 4.15 (t, *J* = 1.4 Hz, 3H), 3.58 (s, 3H), 3.41 (s, 3H) ppm.

^{13}C NMR (101 MHz, $CDCl_3$): δ 155.6, 151.5, 146.5, 143.6 (t, *J* = 30.0 Hz), 110.1 (t, *J* = 302.3 Hz), 109.0, 33.8, 30.1, 28.3 ppm.

^{19}F NMR (376 MHz, $CDCl_3$): δ – 47.67 (d, *J* = 1.4 Hz) ppm.

HRMS (ESI, *m/z*) calcd. For $C_9H_9BrF_2N_4O_2$ [M+]: 321.9877; found 321.9879.

Spectroscopic data is in agreement with previously reported literature data.[9]

3,7-Dimethyl-1-(3-oxobutyl)-8-(chlorodifluoromethyl)-3,7-dihydro-1H-purine-2,6-dione (18b)

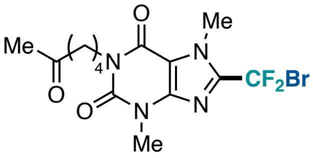

18b

Prepared following the general procedure D and starting from pentoxifylline (139.2 mg, 0.5 mmol). The crude mixture was purified by flash column chromatography on a silica gel column eluting with EtOAc to provide **18b** as a white solid (107 mg, 53% yield).

Rf = 0.55 (EtOAc).

^{1}H NMR (400 MHz, $CDCl_3$): δ 4.13 (t, *J* = 1.4 Hz, 3H), 3.99 (t, *J* = 7.0 Hz, 2H), 3.55 (s, 3H), 2.48 (t, *J* = 6.9 Hz, 2H), 2.12 (s, 3H), 1.66 – 1.60 (m, 4H) ppm.

^{13}C NMR (151 MHz, $CDCl_3$): δ 208.7, 155.5, 151.2, 146.5, 143.6 (t, *J* = 29.9 Hz), 110.1 (t, *J* = 302.1 Hz), 109.6, 43.2, 41.2, 33.7, 30.1, 29.9, 27.5, 21.0 ppm.

^{19}F NMR (376 MHz, $CDCl_3$): δ – 47.69 (d, *J* = 1.5 Hz) ppm.

HRMS (ESI, *m/z*) calcd. For $C_{14}H_{17}BrF_2N_4O_3$ [M+]: 406.0460; found 406.0452.

REPRESENTATIVE SCALED UP REACTION

1,3,7-Trimethyl-8-(chlorodifluoromethyl)-3,7-dihydro-1H-purine-2,6-dione (17a)

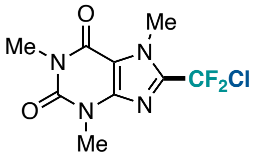

17a

An oven-dried 12 mL Schlenk tube with a screw cap containing a stirring bar was charged with NaO_2CCF_2Cl (916 mg, 6.0 mmol, 6.0 equiv), $Fe(OTf)_2$ (35.6 mg, 0.10 mmol, 10 mol%), 4,4'-dimethoxy-2,2'-bipyridine **L1** (21.2 mg, 0.10 mmol, 10 mol%), $K_2S_2O_8$ (812 mg, 3.0 mmol, 3.0 equiv) and caffeine (194.2 mg, 1.0 mmol, 1.0 equiv). The tube was evacuated and back-filled with nitrogen, and this procedure was repeated three times. Against a positive N_2 flow, the degassed MeCN (10 mL) was added via a syringe. The tube was sealed, placed on the photoreactor, and irradiated with 405 nm LEDs at 25 ºC. After 48 hours, the reaction was quenched with saturated aqueous $NaHCO_3$ solution (4 mL), diluted with EtOAc (20 mL) and transferred to a separating funnel. The two phases were separated, the organic layer was washed with saturated aqueous $NaHCO_3$ solution (2x30mL), then brine (30 mL) and dried over Na_2SO_4. After removal of the solvent under reduced pressure, the crude mixture was purified by flash column chromatography on a silica gel column eluting with EtOAc to provide **17a** as a white solid (207 mg, 74% yield).

Rf = 0.69 (Hex/EtOAc 1:1 (v/v)).
^{1}H NMR (400 MHz, $CDCl_3$): δ 4.17 (t, J = 1.4 Hz, 3H), 3.58 (s, 3H), 3.41 (s, 3H) ppm.
^{13}C NMR (101 MHz, $CDCl_3$): δ 155.6, 151.5, 146.4, 142.6 (t, J = 32.9 Hz), 119.8 (t, J = 288.2 Hz), 109.7, 33.6 (t, J = 2.8 Hz), 30.0, 28.3 ppm.
^{19}F NMR (376 MHz, $CDCl_3$): δ – 51.01 ppm.
HRMS (ESI, *m*/*z*) calcd. For $C_9H_9ClF_2N_4O_2$ $[M+H]^+$: 279.0455; found 279.0460.
Spectroscopic data is in agreement with previously reported literature data.[1]

SYNTHETIC APPLICATIONS

Preparation of 8-(difluoro(phenoxy)methyl)-1,3,7-trimethyl-3,7-dihydro-1H-purine-2,6-dione (21)

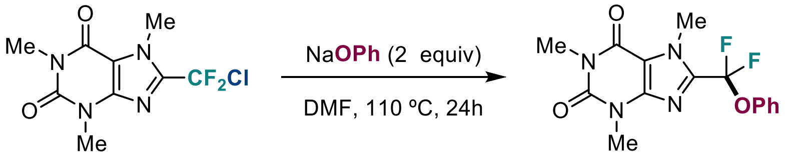

This compound was prepared from a slightly modified reported procedure.[7]

An oven-dried crimp top vial was charged with a stir bar along with the chlorodifluoromethylated product **17a** (27.9 mg, 1.0 equiv, 0.10 mmol) and sodium phenoxide (34 mg, 2.0 equiv, 0.20 mmol). The sealed vial was evacuated and backfilled with nitrogen 3 times, with 5 minutes between each cycle. Dry N,N-dimethylformamide (1 mL, 0.1 M) was added via syringe, and the vial was sealed with parafilm. The reaction mixture was allowed to stir 24 hours at 110°C in an oil bath. After the reaction was done, the reaction was quenched with H2O and transferred to an extraction funnel. After removal of the solvent under reduced pressure, the crude mixture was purified by flash column chromatography on a silica gel column with a mixture of hexane:EtOAc (1:1) to provide **21** as a yellow solid (20 mg, 59% yield).

^{1}H NMR (600 MHz, $CDCl_3$): δ 7.42 – 7.38 (m, 2H), 7.30 – 7.27 (m, 3H), 4.21 (s, 3H), 3.61 (s, 3H), 3.42 (s, 3H) ppm.

^{13}C NMR (151 MHz, $CDCl_3$): δ 155.7, 151.6, 149.4, 146.7, 142.2 (t, J = 39.4 Hz), 129.9, 126.7, 121.9, 116.8 (t, J = 261.3 HZ), 109.4, 33.5, 30.1, 28.3 ppm.

^{19}F NMR (376 MHz, $CDCl_3$): δ – 65.24 ppm.

HRMS (ESI, *m*/*z*) calcd. For $C_{15}H_{14}F_2N_4O_3$ [M+]: 336.1034; found 336.1038.

Spectroscopic data is in agreement with previously reported literature data.[7]

Preparation of 8-(difluoro(trimethylsilyl)methyl)-1,3,7-trimethyl-3,7-dihydro-1*H*-purine-2,6-dione (22)

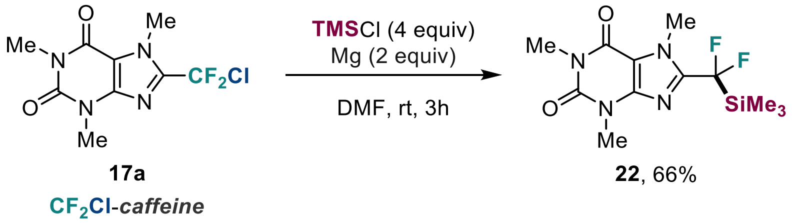

This compound was prepared from a slightly modified reported procedure.[10]

An oven-dried crimp top vial was charged with a stir bar along with the chlorodifluoromethylated product **17a** (27.9 mg, 1.0 equiv, 0.10 mmol), magnesium metal (4.9 mg, 2.0 equiv, 0.20 mmol) and trimethylsilyl chloride (43.5 mg, 4.0 equiv, 0.4 mmol). The sealed vial was evacuated and backfilled with nitrogen 3 times, with 5 minutes between each cycle. Dry N,N-dimethylformamide (1 mL, 0.1 M) was added via syringe, and the vial was sealed with parafilm. After stirring for 3h at room temperature, EtOAc (10 mL) and H_2O (15 mL) were added to the reaction mixture. The phases were separated, and the aqueous layer was extracted with EtOAc (2x10mL). The combined organic layer was dried over anhydrous Na_2SO_4 and after removal of the solvent under reduced pressure, the crude mixture was purified by flash column chromatography on a silica gel column eluting with EtOAc to provide **22** as a white solid (21 mg, 66%).

^{1}H NMR (600 MHz, $CDCl_3$): δ 4.15 (s, 3H), 3.54 (s, 3H), 3.41 (s, 3H), 0.34 (s, 9H) ppm.

^{13}C NMR (151 MHz, $CDCl_3$): δ 155.8, 151.7, 146.7, 146.4 (t, J = 26.4 Hz), 124.3 (t, J = 255.1 Hz), 109.1, 33.3, 29.8, 28.2, -3.82 ppm.

^{19}F NMR (376 MHz, $CDCl_3$): δ – 111.3 ppm.

HRMS (ESI, *m/z*) calcd. For $C_{12}H_{18}F_2N_4O_2Si$ [M+]: 316.1167; found 316.1165.

Spectroscopic data is in agreement with previously reported literature data.[10]

Preparation of methyl 1-methyl-2-oxo-1,2-dihydropyridine-3-carboxylate (23)

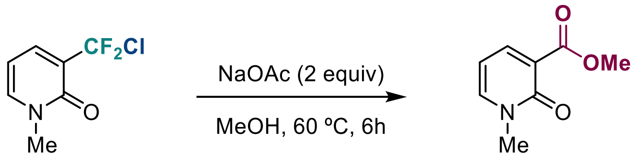

10a **23**, 84%

This compound was prepared from a slightly modified reported procedure.[1]

An oven-dried crimp top vial was charged with a stir bar along with **10a** (19 mg, 0.1 mmol, 1 equiv), sodium acetate (8 mg, 0.1 mmol, 1 equiv) and 1 mL MeOH. After stirring for 6 h at 60 °C in an oil bath, CH_2Cl_2 (4 mL) and H_2O (3 mL) were added to the reaction mixture. The phases were separated, and the aqueous layer was washed with CH_2Cl_2 (3 x 5 mL), and the organic layers combined were washed with Brine (10 mL) and dried over anhydrous Na_2SO_4. After removal of the solvent under reduced pressure, the crude mixture was purified by flash column chromatography on a silica gel column eluting with a gradient of CH_2Cl_2/MeOH (100:0 to 90:10) to provide **23** as a dark brown oil (14 mg, 84%).

^{1}H NMR (600 MHz, $CDCl_3$): δ 8.14 (dd, *J* = 7.2, 2.2 Hz, 1H), 7.54 (dd, *J* = 6.6, 2.2 Hz, 1H), 6.22 (t, *J* = 6.9 Hz, 1H), 3.89 (s, 3H), 3.58 (s, 3H) ppm.

^{13}C NMR (151 MHz, $CDCl_3$): δ 166.0, 159.9, 145.0, 143.2, 120.7, 104.7, 52.5, 38.5 ppm.

HRMS (ESI, *m/z*) calcd. For $C_8H_9NO_3$ [M+]: 167.0582; found 167.0577.

Spectroscopic data is in agreement with previously reported literature data.[1]

C–H FLUOROCARBONYLATION OF (HETERO)ARENES

General procedure E

An oven-dried 12 mL Schlenk tube with a screw cap containing a stirring bar was charged with NaO_2CCF_2Cl (458 mg, 3.0 mmol, 6.0 equiv), $Fe(OTf)_2$ (17.8 mg, 0.05 mmol, 10 mol%), 4,4'-dimethoxy-2,2'-bipyridine **L1** (10.6 mg, 0.05 mmol, 10 mol%), $K_2S_2O_8$ (406 mg, 1.5 mmol, 3.0 equiv) and the corresponding (hetero)aromatic substrate (0.50 mmol, 1.0 equiv). The tube was evacuated and back-filled with nitrogen, and this procedure was repeated three times. Against a positive N_2 flow, the degassed MeCN (5 mL) was added via a syringe. The tube was sealed, placed on the photoreactor, and irradiated with 405 nm LEDs at 25ºC. After 24 hours, 5 mL of DMSO were added in open air into the Schlenk, and the reaction was stirred for another 24 hours without irradiation. Then, the reaction was quenched with saturated aqueous $NaHCO_3$ solution (2 mL), diluted with EtOAc (10 mL) and transferred to a separating funnel. The two phases were separated, the organic layer was washed with saturated aqueous $NaHCO_3$ solution (2x15mL), then brine (15 mL) and dried over Na_2SO_4. After removal of the solvent under reduced pressure, the crude mixture was purified by flash column chromatography on a silica gel column to afford the desired product.

General procedure F

An oven-dried 12 mL Schlenk tube with a screw cap containing a stirring bar was charged with NaO_2CCF_2Br (590.7 mg, 3.0 mmol, 6.0 equiv), $Fe(OTf)_2$ (17.8 mg, 0.05 mmol, 10 mol%), 4,4'-dimethoxy-2,2'-bipyridine **L1** (10.6 mg, 0.05 mmol, 10 mol%), $K_2S_2O_8$ (406 mg, 1.5 mmol, 3.0 equiv) and the corresponding (hetero)aromatic substrate (0.50 mmol, 1.0 equiv). The tube was evacuated and back-filled with nitrogen, and this procedure was repeated three times. Against a positive N_2 flow, the degassed MeCN (5 mL) was added via a syringe. The tube was sealed, placed on the photoreactor, and irradiated with 405 nm LEDs at 25ºC. After 24 hours, 5 mL of DMSO were added in open air into the Schlenk, and the reaction was stirred for another 24 hours without irradiation. Then, the reaction was quenched with saturated aqueous $NaHCO_3$ solution (2 mL), diluted with EtOAc (10 mL) and transferred to a separating funnel. The two phases were separated, the organic layer was washed with saturated aqueous $NaHCO_3$ solution (2x15mL), then brine (15 mL) and dried over Na_2SO_4. After removal of the solvent under reduced pressure, the crude mixture was purified by flash column chromatography on a silica gel column to afford the desired product.

SUBSTRATE SCOPE OF THE C–H FLUOROCARBONYLATION OF (HETERO)ARENES

5-(Tert-butyl)-2-methoxybenzoyl fluoride (24)

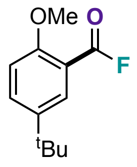

24

Prepared following the general procedure F and starting from 1-(tert-butyl)-4-methoxybenzene (88 µL, 0.5 mmol). The crude mixture was purified by flash column chromatography on a silica gel column using a gradient from hexane to a mixture of hexane/EtOAc (10:1) to provide **24** as a pale yellow solid (53 mg, 50% yield).

Rf = 0.33 (Hex/Et_2O 12:1 (v/v)).
^{1}H NMR (300 MHz, $CDCl_3$): δ 7.91 (d, *J* = 2.6 Hz, 1H), 7.65 (dd, *J* = 8.8, 2.6 Hz, 1H), 6.98 (dd, *J* = 8.8, 1.4 Hz, 1H), 3.94 (s, 3H), 1.32 (s, 9H) ppm.
^{13}C NMR (75 MHz, $CDCl_3$): δ 159.6 (d, *J* = 3.9 Hz), 155.7 (d, *J* = 342.8 Hz), 143.4, 134.1, 130.5 (d, *J* = 3.0 Hz), 112.7 (d, *J* = 57.3 Hz), 112.1 (d, *J* = 3.2 Hz), 56.2, 34.3, 31.4 ppm.
^{19}F NMR (282 MHz, $CDCl_3$): δ 31.35 ppm.
HRMS (ESI, *m/z*) calcd. For $C_{12}H_{15}FO_2$ [M+]: 210.1056; found 210.1053.
Spectroscopic data is in agreement with previously reported literature data.[8]

2,5-Dimethoxybenzoyl fluoride (25)

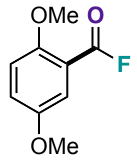

25

Prepared following the general procedure F and starting from 1,4-dimethoxybenzene dimethoxybenzene (69 mg, 0.5 mmol). The crude mixture was purified by flash column

chromatography on a silica gel column using a mixture of hexane/Et_2O (6:1) to provide **25** as a pale yellow solid (34 mg, 37% yield).

Rf = 0.22 (Hex/Et_2O 6:1 (v/v)).

^{1}H NMR (400 MHz, $CDCl_3$): δ 7.40 (d, *J* = 3.2 Hz, 1H), 7.19 (dd, *J* = 9.2, 3.2 Hz, 1H), 6.98 (dd, *J* = 9.2, 1.4 Hz, 1H), 3.91 (s, 3H), 3.81 (s, 3H) ppm.

^{13}C NMR (101 MHz, $CDCl_3$): δ 156.2 (d, J = 3.8 Hz), 155.2 (d, J = 343.0 Hz), 153.1, 123.6, 117.1 (d, J = 2.8 Hz), 113.9 (d, J = 3.3 Hz), 113.5 (d, J = 58.9 Hz), 56.6, 55.0 ppm.

^{19}F NMR (376 MHz, $CDCl_3$): δ 31.71 ppm.

HRMS (ESI, *m/z*) calcd. For $C_9H_9FO_3$ [M+]: 184.0536; found 184.0528.

Spectroscopic data is in agreement with previously reported literature data.[8]

2,3-Dimethoxybenzoyl fluoride (26)

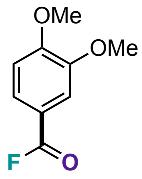

26

Prepared following the general procedure F and starting from 1,2-dimethoxybenzene (64 µL, 0.5 mmol). The crude mixture was purified by flash column chromatography on a silica gel column using a mixture of hexane/Et_2O (6:1) to provide **26** as a white solid (44 mg, 48% yield).

Rf = 0.17 (Hex/Et_2O 6:1 (v/v)).

^{1}H NMR (400 MHz, $CDCl_3$): δ 7.70 (dd, *J* = 8.5, 2.1 Hz, 1H), 7.48 (d, *J* = 1.7 Hz, 1H), 6.94 (dd, *J* = 8.5, 1.1 Hz, 1H), 3.97 (s, 3H), 3.93 (s, 3H) ppm.

^{13}C NMR (101 MHz, $CDCl_3$): δ 157.5 (d, *J* = 340.1 Hz), 155.2, 149.3 (d, *J* = 1.9 Hz), 126.4 (d, *J* = 3.4 Hz), 117.0 (d, *J* = 62.2 Hz), 113.2 (d, *J* = 4.7 Hz), 110.8, 56.4, 56.2 ppm.

^{19}F NMR (376 MHz, $CDCl_3$): δ 15.64 ppm.

HRMS (ESI, *m/z*) calcd. For $C_9H_9FO_3$ [M+]: 184.0536; found 184.0530.

Spectroscopic data is in agreement with previously reported literature data.[8]

2,3-Dimethoxy-6-methylbenzoyl fluoride (27)

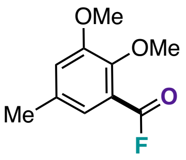

27

Prepared following the general procedure E and starting from 1,2-dimethoxy-4-methylbenzene (72 µL, 0.5 mmol). The crude mixture was purified by flash column chromatography on a silica gel column using a mixture of hexane/Et_2O (6:1) to provide **27** as a yellow solid (59 mg, 60% yield).

Rf = 0.14 (Hex/Et_2O 12:1 (v/v)).
^{1}H NMR (400 MHz, $CDCl_3$): δ 7.43 (s, 1H), 6.76 (s, 1H), 3.95 (s, 3H), 3.90 (s, 3H), 2.60 (d, *J* = 2.0 Hz 3H) ppm.
^{13}C NMR (101 MHz, $CDCl_3$): δ 156.7 (d, *J* = 340.9 Hz), 154.2, 147.0, 139.2 (d, *J* = 6.9 Hz), 114.9 (d, *J* = 57.4 Hz), 114.5 (d, *J* = 7.3 Hz), 114.5 (d, *J* = 5.1 Hz), 56.3, 56.2, 21.9 ppm.
^{19}F NMR (376 MHz, $CDCl_3$): δ 26.81 ppm.
HRMS (ESI, *m/z*) calcd. For $C_{10}H_{11}FO_3$ [M+]: 198.0692; found 198.0688.

2,3-Dihydrothieno[3,4-b][1,4]dioxine-5-carbonyl fluoride (28)

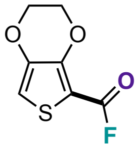

28

Prepared following the general procedure E and starting from 2,3-dihydrothieno[3,4-b][1,4]dioxine (54µL, 0.5 mmol). The crude mixture was purified by flash column chromatography on a silica gel column using a mixture of hexane/Et_2O (6:1) to provide **28** as a yellow solid (37 mg, 39% yield).

Rf = 0.11 (Hex/ Et_2O 6:1 (v/v)).
^{1}H NMR (400 MHz, $CDCl_3$): δ 6.81 (d, *J* = 0.8 Hz, 1H), 4.42 (m, 2H), 4.26 (m, 2H) ppm.
^{13}C NMR (101 MHz, $CDCl_3$): δ 151.5 (d, *J* = 326.5 Hz), 149.7 (d, *J* = 6.0 Hz), 142.0 (d, *J* = 4.6 Hz), 111.4 (d, *J* = 1.3 Hz), 101.6 (d, *J* = 76.7 Hz), 65.7, 64.1 ppm.
^{19}F NMR (376 MHz, $CDCl_3$): δ 27.20 ppm.

HRMS (ESI, *m/z*) calcd. For $C_7H_5FO_3S$ [M+]: 187.9943; found 187.9938.

Tert-butyl 2-(fluorocarbonyl)-1H-pyrrole-1-carboxylate (29)

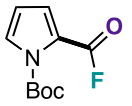

29

Prepared following the general procedure F and starting from tert-butyl 1*H*-pyrrole-1-carboxylate (84 μL, 0.5 mmol). The crude mixture was purified by flash column chromatography on a silica gel column using a mixture of cyclohexane/Et_2O (30:1) to provide **29** as an orange oil (43 mg, 40% yield).

Rf = 0.31 (Cyclohexane/ Et_2O 20:1 (v/v)).

^{1}H NMR (400 MHz, $CDCl_3$): δ 7.55 (m, 1H), 7.20 (m, 1H), 6.28 (dd, J = 3.7, 3.1 Hz, 1H), 1.61 (s, 9H) ppm.

^{13}C NMR (101 MHz, $CDCl_3$): δ 149.4 (d, *J* = 328.5 Hz), 147.8, 130.9 (d, *J* = 3.8 Hz), 127.2, 118.9 (d, *J* = 87.6 Hz), 111.0, 86.4, 27.7 ppm.

^{19}F NMR (376 MHz, $CDCl_3$): δ 25.92 ppm.

HRMS (ESI, *m/z*) calcd. For $C_{10}H_{12}FNO_3$ [M+]: 213.0801; found 213.0794.

2,6-Dimethoxynicotinoyl fluoride (30)

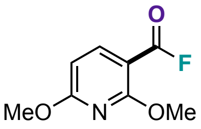

30

Prepared following the general procedure E and starting from 2,6-dimethoxypyridine (66 μL, 0.5 mmol). The crude mixture was purified by flash column chromatography on a silica gel column using a gradient from cyclohexane to a mixture of cyclohexane/EtOAc (20:1) to provide **30** as a white solid (52 mg, 56% yield).

Rf = 0.24 (Cyclohexane/ Et_2O 20:1 (v/v)).

^{1}H NMR (400 MHz, $CDCl_3$): δ 8.09 (d, *J* = 8.6 Hz, 1H), 6.39 (d, *J* = 8.6z Hz, 1H), 4.09 (s, 3H), 4.01 (s, 3H) ppm.

^{13}C NMR (151 MHz, $CDCl_3$): δ 167.5, 165.1 (d, *J* = 6.2 Hz), 154.6 (d, *J* = 335.3 Hz), 145.3, 103.2, 99.02 (d, *J* = 63.8 Hz), 54.6, 54.4 ppm.

^{19}F NMR (376 MHz, $CDCl_3$): δ 26.54 ppm.

HRMS (ESI, *m/z*) calcd. For $C_8H_8FNO_3$ [M+]: 185.0488; found 185.0485.

Spectroscopic data is in agreement with previously reported literature data.[8]

2,4,6-Trimethoxypyrimidine-5-carbonyl fluoride (31)

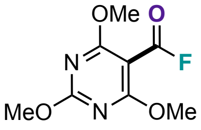

31

Prepared following the general procedure E and starting from 2,4,6-trimethoxypyrimidine (85 mg, 0.5 mmol). The crude mixture was purified by flash column chromatography on a silica gel column using a mixture of hexane/AcOEt (10:1) to provide **31** as a pale yellow solid (43 mg, 40% yield).

Rf = 0.14 (Hex/Et_2O 6:1 (v/v)).

^{1}H NMR (400 MHz, $CDCl_3$): δ 4.07 (s, 6H), 4.04 (m, 3H) ppm.

^{13}C NMR (101 MHz, $CDCl_3$): δ 172.8, 166.1, 153.4 (d, J = 338.0 Hz), 86.7 (d, J = 63.4 Hz), 55.7, 55.6 ppm.

^{19}F NMR (376 MHz, $CDCl_3$): δ 44.08 ppm.

HRMS (ESI, *m/z*) calcd. For $C_8H_9FN_2O_4$ [M+]: 216.0546; found 216.0538.

LATE-STAGE DIVERSIFICATION OF ACYL FLUORIDES

<u>General procedure G</u>

To an oven-dried 12 mL Schlenk tube with a screw cap containing a stirring bar was charged with 2,6-dimethoxynicotinoyl fluoride **30** (105 mg, 0.2 mmol), complex molecule (1.1 equiv) and DIPEA (38 µL, 1.1 equiv). Then DCM (2 mL) was added, purged with nitrogen and the reaction mixture was stirred for 20 hours at 25 ºC.[8] The solvent was removed in vacuo, and the residue was purified by column chromatography.

Preparation of (8*R*,9*S*,13*S*,14*S*)-13-methyl-17-oxo-7,8,9,11,12,13,14,15,16,17-decahydro-6*H*-cyclopenta[*a*]phenanthren-3-yl 2,6-dimethoxynicotinate (32)

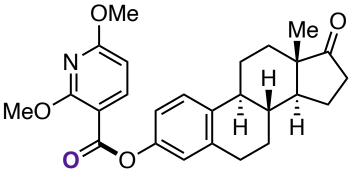

32

Starting from **30** and estrone. The crude reaction was purified by flash column chromatography on basic alumina column using a mixture of cyclohexane/EtOAc (3:1) to provide **32** as a yellow oil (53 mg, 61% yield).

^{1}H NMR (600 MHz, $CDCl_3$): δ 8.30 (d, *J* = 8.4 Hz, 1H), 7.30 (d, *J* = 8.1 Hz, 1H), 6.98 – 6.90 (m, 2H), 6.38 (d, *J* = 8.4 Hz, 1H), 4.06 (s, 3H), 4.00 (s, 3H), 2.96 – 2.88 (m, 2H), 2.57 – 2.46 (m, 1H), 2.46 – 2.39 (m, 1H), 2.31 (td, *J* = 10.7, 4.0 Hz, 1H), 2.22 – 1.90 (m, 5H), 1.71 – 1.39 (m, 8H) ppm.

^{13}C NMR (151 MHz, $CDCl_3$): δ 221.0, 166.1, 163.6, 163.5, 149.0, 144.6, 138.0, 137.2, 126.4, 122.0, 119.2, 104.2, 102.1, 54.3, 54.1, 50.6, 48.1, 44.3, 38.2, 36.0, 31.7, 29.5, 26.5, 25.9, 21.7, 14.0 ppm.

HRMS (ESI, *m*/*z*) calcd. For $C_{26}H_{29}NO_5$ $[M+H]^+$: 436.2124; found 436.2124.

(*R*)-*N*-(1-(1-acryloylpiperidin-3-yl)-3-(4-phenoxyphenyl)-1*H*-pyrazolo[3,4-*d*]pyrimidin-4-yl)-2,6-dimethoxynicotinamide (33)

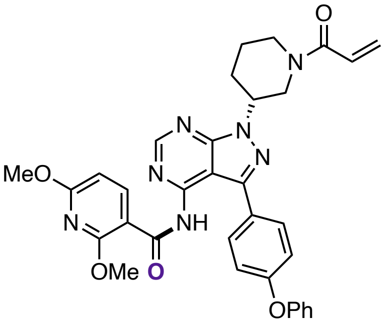

33

Starting from **30** and Ibrutinib. The crude reaction was purified by column chromatography using a mixture of EtOAc/MeOH (20:1) to provide **33** as a yellow oil (69 mg, 57% yield).

^{1}H NMR (600 MHz, $CDCl_3$): δ 10.16 (s, 1H), 8.83 (s, 1H), 8.26 (d, *J* = 8.3 Hz, 1H), 7.69 (d, *J* = 8.5 Hz, 2H), 7.38 – 7.28 (m, 2H), 7.18 – 7.09 (m, 1H), 7.06 – 6.96 (m, 4H), 6.69 – 6.51 (m, 1H), 6.45 (d, *J* = 8.4 Hz, 1H), 6.30 (t, *J* = 13.0 Hz, 1H), 5.69 (dd, *J* = 21.1, 10.5 Hz, 1H), 4.93–4.01 (m, 2H), 3.99 (s, 3H), 3.87 – 3.76 (m, 1H), 3.72 (s, 3H), 3.50 – 2.83 (m, 2H), 2.52 – 2.33 (m, 1H), 2.33 – 2.24 (m, 1H), 2.09 – 1.97 (m, 1H), 1.83 – 1.68 (m, 1H) ppm.
^{13}C NMR (151 MHz, $CDCl_3$): δ 165.9, 165.7, 161.6, 160.1, 158.4, 156.5, 155.3, 152.9, 145.3, 144.9, 130.3, 130.0, 128.2, 127.7, 124.0, 119.4, 118.7, 106.9, 104.4, 104.0, 54.3, 54.1, 53.9, 52.9, 50.1, 46.2, 46.0, 42.3, 30.4, 30.1, 29.8, 25.4, 24.0 ppm.
HRMS (ESI, *m*/*z*) calcd. For $C_{33}H_{31}N_7O_5$ $[M+H]^+$: 606.2465; found 606.2464.

MECHANISTIC INVESTIGATIONS

Mechanistic hypothesis

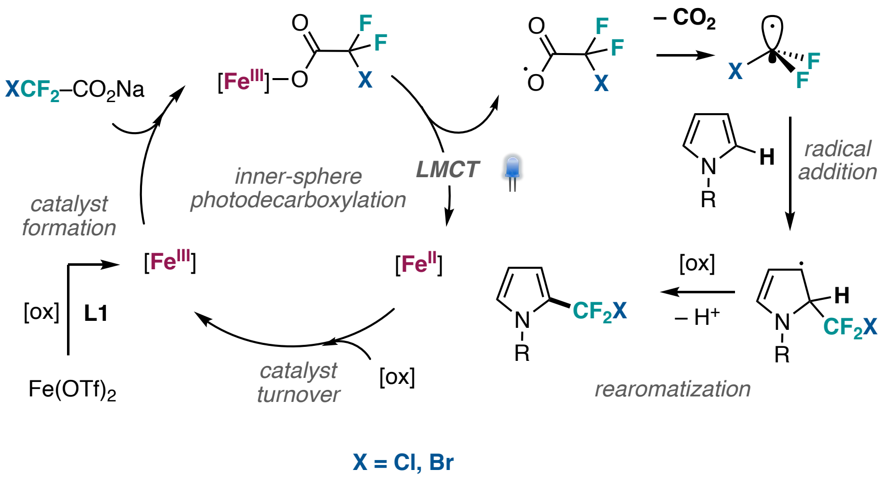

Fig. S8. Mechanistic proposal.

First, the catalytically competent Fe(III) species is generated in situ through coordination of ligand **L1** to Fe(OTf)₂, followed by oxidation with $K_2S_2O_8$. Binding of the corresponding chloro- or bromodifluoroacetates then prepares the system for an inner-sphere photodecarboxylation process under 405 nm irradiation. This step involves homolytic cleavage of the Fe–O bond in the iron-carboxylate intermediate, producing the fluoroalkyl radical. The resulting radical subsequently adds to the substrate, and the desired fluoroalkylated product is obtained after oxidation and restoration of aromaticity. Regeneration of the active catalyst occurs through oxidation of Fe(II) back to Fe(III) by the inorganic oxidant.

Radical trapping experiments

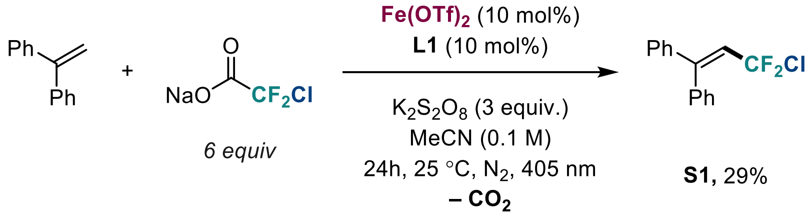

Fig. S9. Chlorodifluoromethylation reaction of 1,1-diphenylethylene.

An oven-dried 12 mL Schlenk tube with a screw cap containing a stirring bar was charged with NaO_2CCF_2Cl (458 mg, 3.0 mmol, 6.0 equiv), $Fe(OTf)_2$ (17.8 mg, 0.05 mmol, 10 mol%), 4,4'-dimethoxy-2,2'-bipyridine **L1** (10.6 mg, 0.05 mmol, 10 mol%), $K_2S_2O_8$ (406 mg, 1.5 mmol, 3.0 equiv). The tube was evacuated and back-filled with nitrogen, and this procedure was repeated three times. Against a positive N_2 flow, diphenylethylene (88 μL, 0.5 mmol) and the degassed MeCN (5 mL) were added via a syringe. The tube was sealed, placed on the photoreactor, and irradiated with 405 nm LEDs at 25 ºC. After 24 hours, hexafluorobenzene (58 μL, 0.5 mmol) was added and the reaction was quenched by the addition of a saturated aqueous solution of $NaHCO_3$ (2 mL) and diluted with EtOAc (10 mL). An aliquot of the organic phase was analyzed by ^{19}F NMR spectroscopy using hexafluorobenzene as an internal standard to determine the yield of **S1** (30%). The presence of diphenylethylene–CF_2Cl adduct was also confirmed by HRMS analysis.

^{19}F NMR (376 MHz, $CDCl_3$): δ –42.89 (d, *J* = 11.7 Hz) ppm.
HRMS (ESI, *m/z*) calcd. For $C_{15}H_{11}ClF_2$ [M+]: 264.0517; found 264.0520.

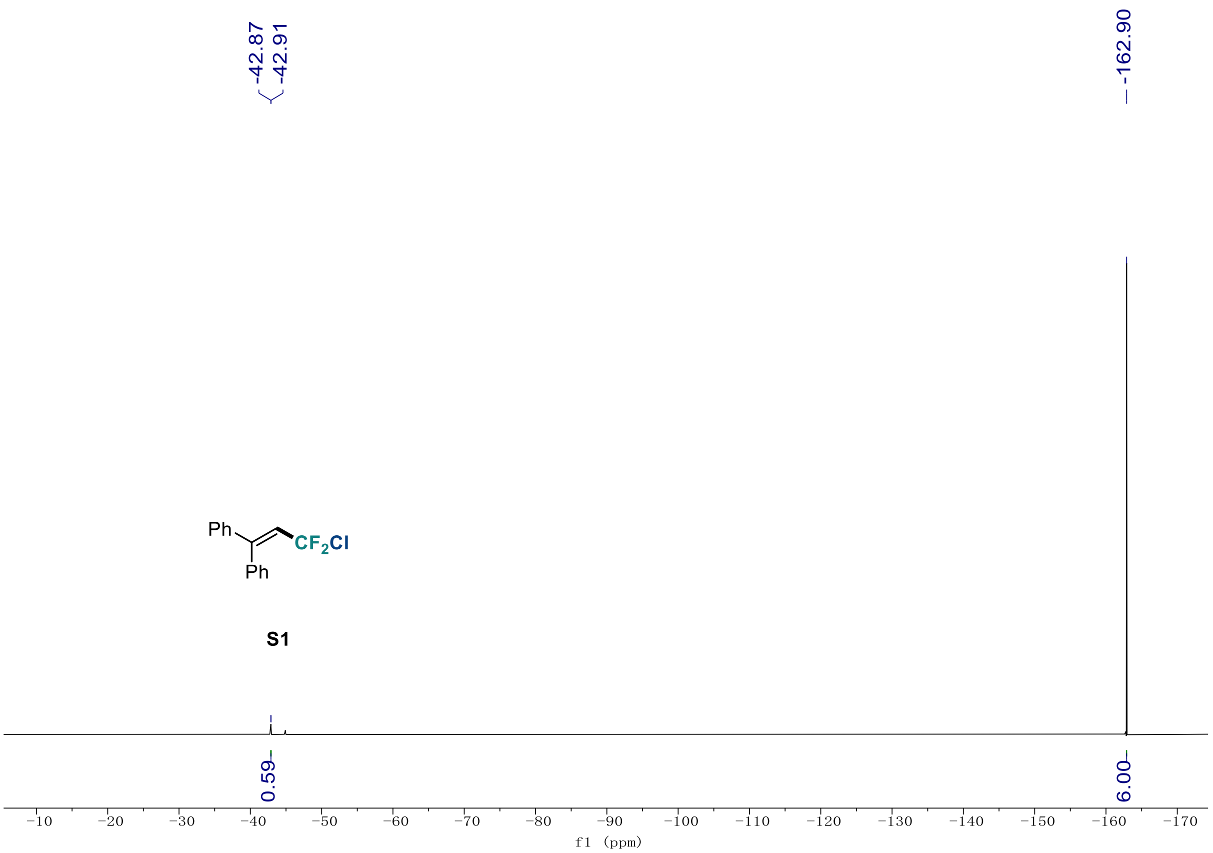

Fig. S10. ^{19}F NMR spectrum of the chlorodifluoromethylation reaction of 1,1-diphenylethylene, showing the formation of S1. Yield determined by ^{19}F NMR using hexafluorobenzene as internal standard.

Radical trapping experiments

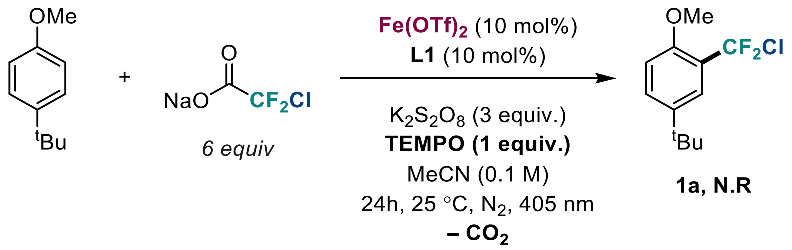

Fig. S11. Radical trapping experiment with the addition of TEMPO

An oven-dried 12 mL Schlenk tube with a screw cap containing a stirring bar was charged with NaO_2CCF_2Cl (458 mg, 3.0 mmol, 6.0 equiv), $Fe(OTf)_2$ (17.8 mg, 0.05 mmol, 10 mol%), 4,4'-dimethoxy-2,2'-bipyridine **L1** (10.6 mg, 0.05 mmol, 10 mol%), $K_2S_2O_8$ (406 mg, 1.5 mmol, 3.0 equiv), 4-tert-butylanisole (82.2 mg, 0.5 mmol) and TEMPO (156 mg, 0.5 mmol, 1 equiv). The tube was evacuated and back-filled with nitrogen, and this procedure was repeated three times. Against a positive N_2 flow, the degassed MeCN (5 mL) were added via a syringe. The tube was sealed, placed on the photoreactor, and irradiated with 405 nm LEDs at 25 ºC. After 24 hours, hexafluorobenzene (35 µL, 0.30 mmol) was added and the reaction was quenched by the addition of a saturated aqueous solution of $NaHCO_3$ (2 mL) and diluted with EtOAc (10 mL). An aliquot of the organic phase was analyzed by ^{19}F NMR spectroscopy using hexafluorobenzene as an internal standard. No product **1a** was detected, indicating that the addition of TEMPO impeded product formation.

UV-Vis absorption spectroscopy study

Samples for UV-Vis monitorization were prepared with the concentration of the optimized reaction conditions and then diluted for UV-Vis spectra acquisition, resulting in a 50μM concentration in iron. Measurements were recorded in a Horiba Duetta fluorescence and absorbance spectrometer.

UV-Vis spectrum of Fe(II) species

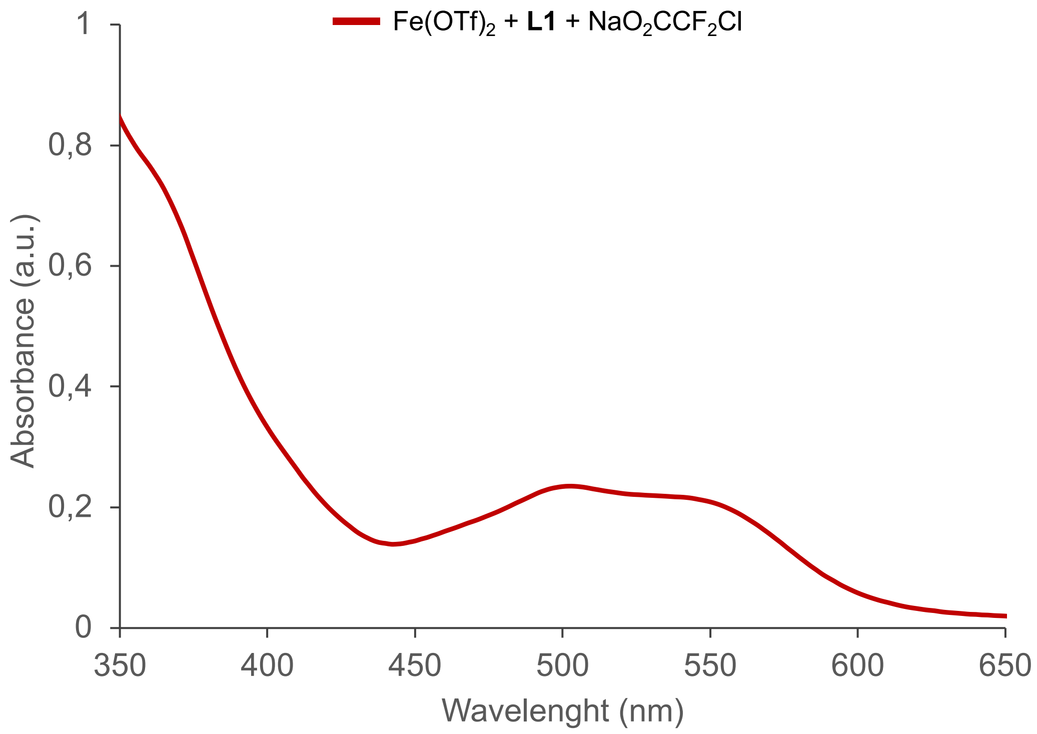

Fig. S12. UV-Vis spectrum of Fe(II) species. Conditions: $Fe(OTf)_2$ (10.6 mg, 0.03 mmol, 1 equiv.), 4,4'-dimethoxy-2,2'-bipyridine (**L1**) (6.5 mg, 0.03 mmol, 1 equiv.) and NaO_2CCF_2Cl (274.4 mg, 1.5 mmol, 60 equiv.) in 3 mL MeCN. The solution was diluted before recording UV-Vis spectrum.

UV-Vis spectrum of Fe(III) species

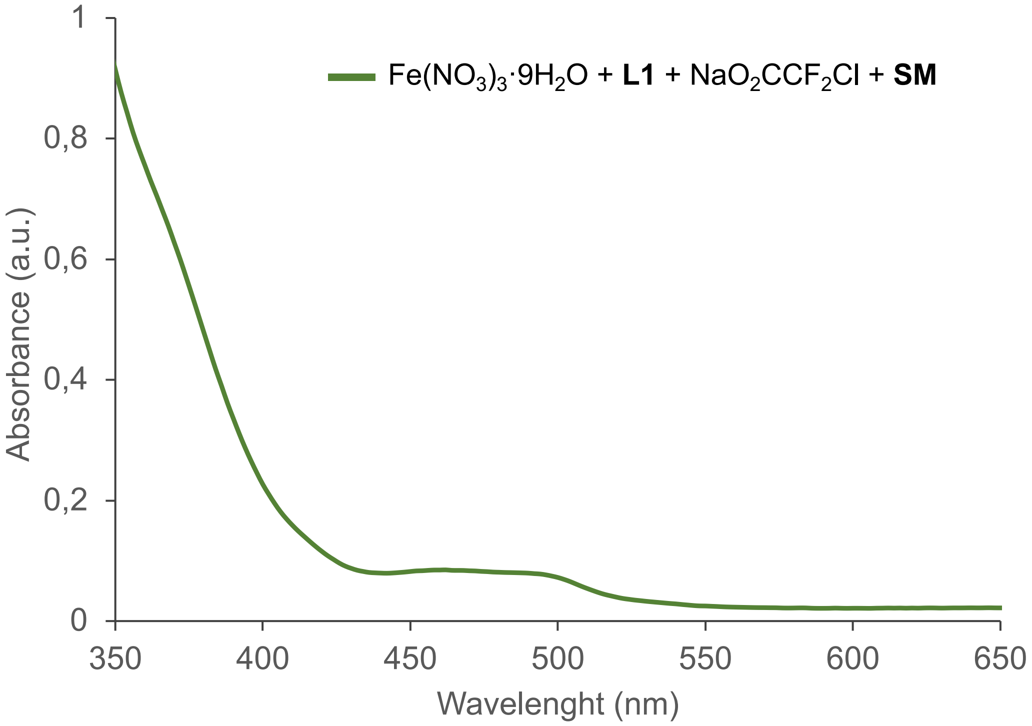

Fig. S13. UV-Vis spectrum of Fe(III) species. Conditions: $Fe(NO_3)_3\cdot 9H_2O$ (12.1 mg, 0.03 mmol, 1 equiv.), 4,4'-dimethoxy-2,2'-bipyridine (**L1**) (6.5 mg, 0.03 mmol, 10 mol%), NaO_2CCF_2Cl (274.4 mg, 1.5 mmol, 60 equiv.) and 4-tert-butylanisole (**SM**, 52.5 µL, 0.30 mmol, 10 equiv.) in 3 mL MeCN. The solution was diluted before recording UV-Vis spectrum.

UV-Vis monitorization of the photodecarboxylation in the absence of oxidant

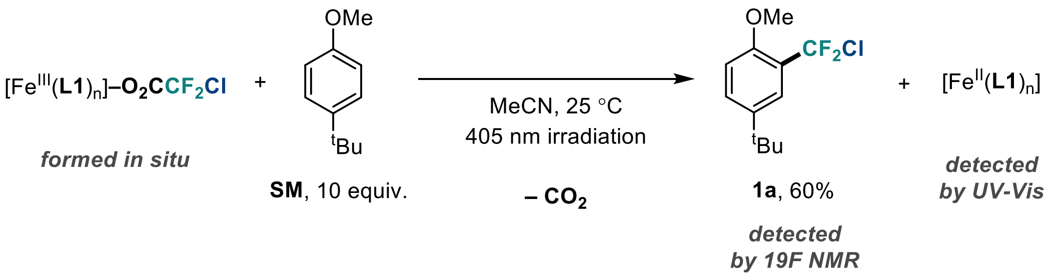

Fig. S14. Photodecarboxylation and chlorodifluoromethylation in the absence of oxidant. Conditions: $Fe(NO_3)_3\cdot 9H_2O$ (12.1 mg, 0.03 mmol, 1 equiv.), 4,4'-dimethoxy-2,2'-bipyridine (**L1**) (6.5 mg, 0.03 mmol, 10 mol%), NaO_2CCF_2Cl (274.4 mg, 1.5 mmol, 60 equiv.) and 4-tert-butylanisole (**SM**, 52.5 µL, 0.30 mmol, 10 equiv.) in 3 mL MeCN, irradiated with 405 nm LEDs

for 24 hours. After reaction, the mixture was analyzed by UV-Vis spectroscopy observing Fe(II) species; and by ^{19}F NMR detecting a 60% of chlorodifluoromethylated product **1a** (calculated respect to Fe), using hexafluorobenzene as internal standard.

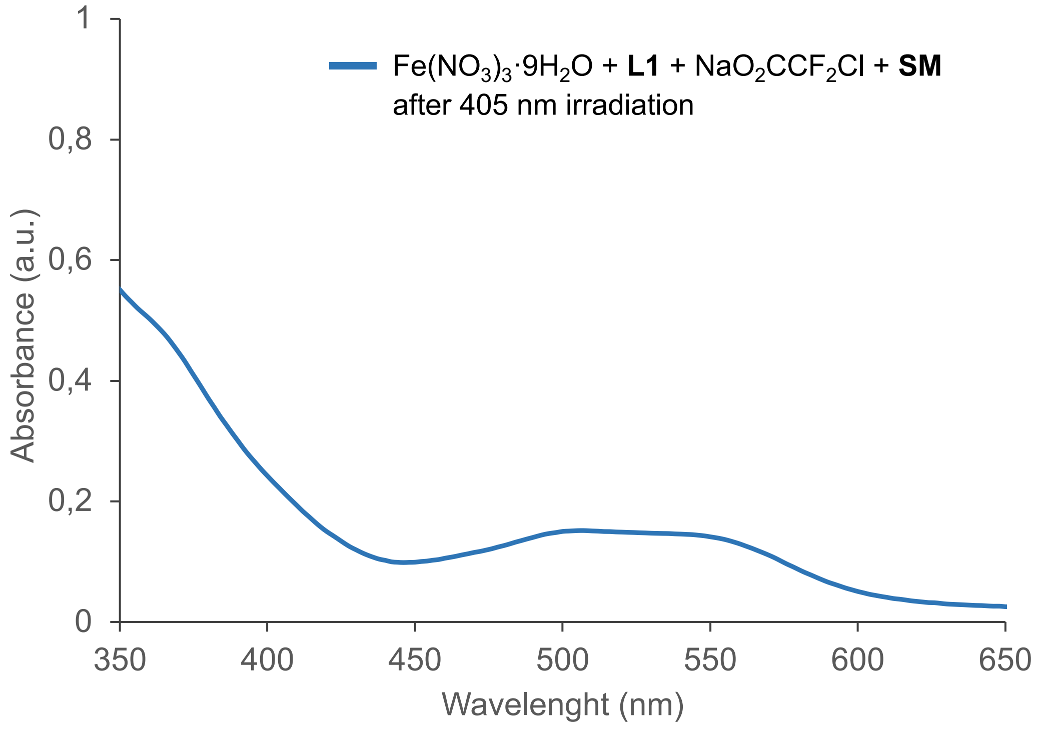

Fig. S15. UV-Vis monitorization of the photodecarboxylation process. Conditions: $Fe(NO_3)_3 \cdot 9H_2O$ (12.1 mg, 0.03 mmol, 1 equiv.), 4,4'-dimethoxy-2,2'-bipyridine (**L1**) (6.5 mg, 0.03 mmol, 10 mol%), NaO_2CCF_2Cl (274.4 mg, 1.5 mmol, 60 equiv.) and 4-tert-butylanisole (**SM**, 52.5 µL, 0.30 mmol, 10 equiv.) in 3 mL MeCN, irradiated with 405 nm LEDs for 24 hours. The solution was diluted before recording UV-Vis spectrum.

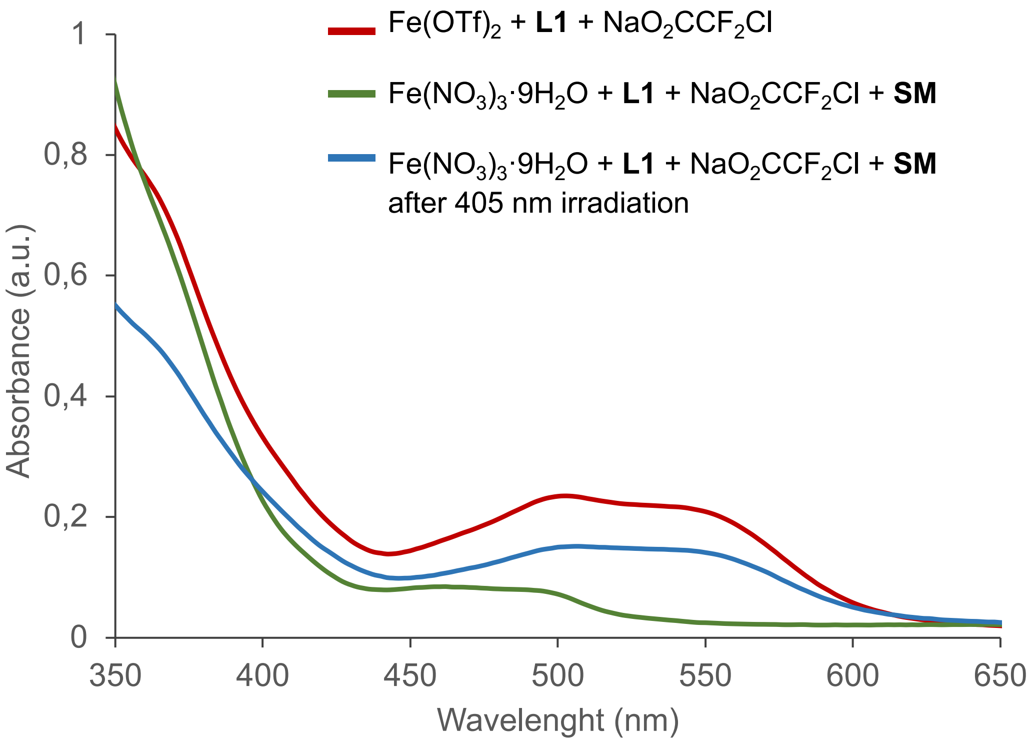

Fig. S16. Overlay of the photodecarboxylation reaction with UV-Vis spectra of Fe(II) and Fe(III) species. Conditions: $Fe(NO_3)_3·9H_2O$ (12.1 mg, 0.03 mmol, 1 equiv.), 4,4'-dimethoxy-2,2'-bipyridine (**L1**) (6.5 mg, 0.03 mmol, 10 mol%), NaO_2CCF_2Cl (274.4 mg, 1.5 mmol, 60 equiv.) and 4-tert-butylanisole (**SM**, 52.5 µL, 0.30 mmol, 10 equiv.) in 3 mL MeCN, irradiated with 405 nm LEDs for 24 hours (blue trace). The solution was diluted before recording UV-Vis spectrum. Red trace corresponds to the Fe(II) species, for comparison. Green trace corresponds to the Fe(III) species, for comparison.

REFERENCES

(1) McAtee, R. C.; Beatty, J. W.; McAtee, C. C.; Stephenson, C. R. J. Radical Chlorodifluoromethylation: Providing a Motif for (Hetero)Arene Diversification. *Org. Lett.* **2018**, *20* (12), 3491–3495. https://doi.org/10.1021/acs.orglett.8b01249.

(2) Meng, D.; Li, L.; Brown, A.; Desrosiers, J.-N.; Duan, S.; Hayward, C. M.; He, Z.; Hu, J.; Makowski, T.; Maloney, M.; Monfette, S.; Perfect, H.; Piper, J. L.; Zhou, M.; Widlicka, D. W. A Radical Chlorodifluoromethylation Protocol for Late-Stage Difluoromethylation and Its Application to an Oncology Candidate. *Cell Reports Physical Science* **2021**, *2* (4), 100394. https://doi.org/10.1016/j.xcrp.2021.100394.

(3) Yin, D.; Su, D.; Jin, J. Photoredox Catalytic Trifluoromethylation and Perfluoroalkylation of Arenes Using Trifluoroacetic and Related Carboxylic Acids. *Cell Reports Physical Science* **2020**, *1* (8), 100141. https://doi.org/10.1016/j.xcrp.2020.100141.

(4) Campbell, B. M.; Gordon, J. B.; Raguram, E. R.; Reynolds, K. G.; Sullivan, M. G.; Nocera, D. G. Ligand-to-Metal Charge Transfer of Ag(II) CF_2 X Carboxylates: Quantum Yield and Electrophotocatalytic Arene Fluoroalkylation Tuned by X. *Angew Chem Int Ed* **2025**, *64* (37), e202511642. https://doi.org/10.1002/anie.202511642.

(5) Bock, L.; Schultheiß, S. K.; Maschauer, S.; Lasch, R.; Gradl, S.; Prante, O.; Zard, S. Z.; Heinrich, M. R. Synthesis of 2-(Chlorodifluoromethyl)Indoles for Nucleophilic Halogen Exchange with [^{18}F]Fluoride. *Eur J Org Chem* **2021**, *2021* (46), 6258–6262. https://doi.org/10.1002/ejoc.202100937.

(6) Zhang, T.; Zhang, Y.; Li, Z.; Wu, B.; Shen, Q. ·YlideFluor-CF2Cl: A Shelf-Stable, Versatile Electrophilic or Radical Chlorodifluoromethylating Reagent. *Org. Chem. Front.* **2024**, *11* (14), 3924–3928. https://doi.org/10.1039/D4QO00600C.

(7) Lin, D.; Krishnamurti, V.; Prakash, G. K. S. Visible Light-Mediated Metal-Free Chlorodifluoromethylation of Arenes and Heteroarenes by a Hypervalent Iodine EDA Complex. *Eur J Org Chem* **2022**, *2022* (35), e202200607. https://doi.org/10.1002/ejoc.202200607.

(8) Zhou, K.; Xiao, Y.; Huang, Z.; Zhao, Y. Photocatalyzed Aryl C-H Fluorocarbonylation with CF_2 Br_2. *Angew Chem Int Ed* **2025**, *64* (2), e202414933. https://doi.org/10.1002/anie.202414933.

(9) Zhang, Y.; Zhu, J.; Shen, Q. Bromodifluoromethyl Sulfonium Ylide: An Easily Available Electrophilic Bromodifluoromethylating Reagent for Bromodifluoromethylation of Styrenes and Heteroarenes by Visible-Light-Promoted Photoredox. *Journal of Fluorine Chemistry* **2022**, *261–262*, 110021. https://doi.org/10.1016/j.jfluchem.2022.110021.

(10) Kawamura, S.; Henderson, C. J.; Aoki, Y.; Sekine, D.; Kobayashi, S.; Sodeoka, M. Reactivity and Properties of Bis(Chlorodifluoroacetyl) Peroxide Generated *in Situ* from Chlorodifluoroacetic Anhydride for Chlorodifluoromethylation Reactions. *Chem. Commun.* **2018**, *54* (80), 11276–11279. https://doi.org/10.1039/C8CC05905E.

NMR Spectral Data

^{1}H-NMR (400 MHz, $CDCl_3$) of 1a

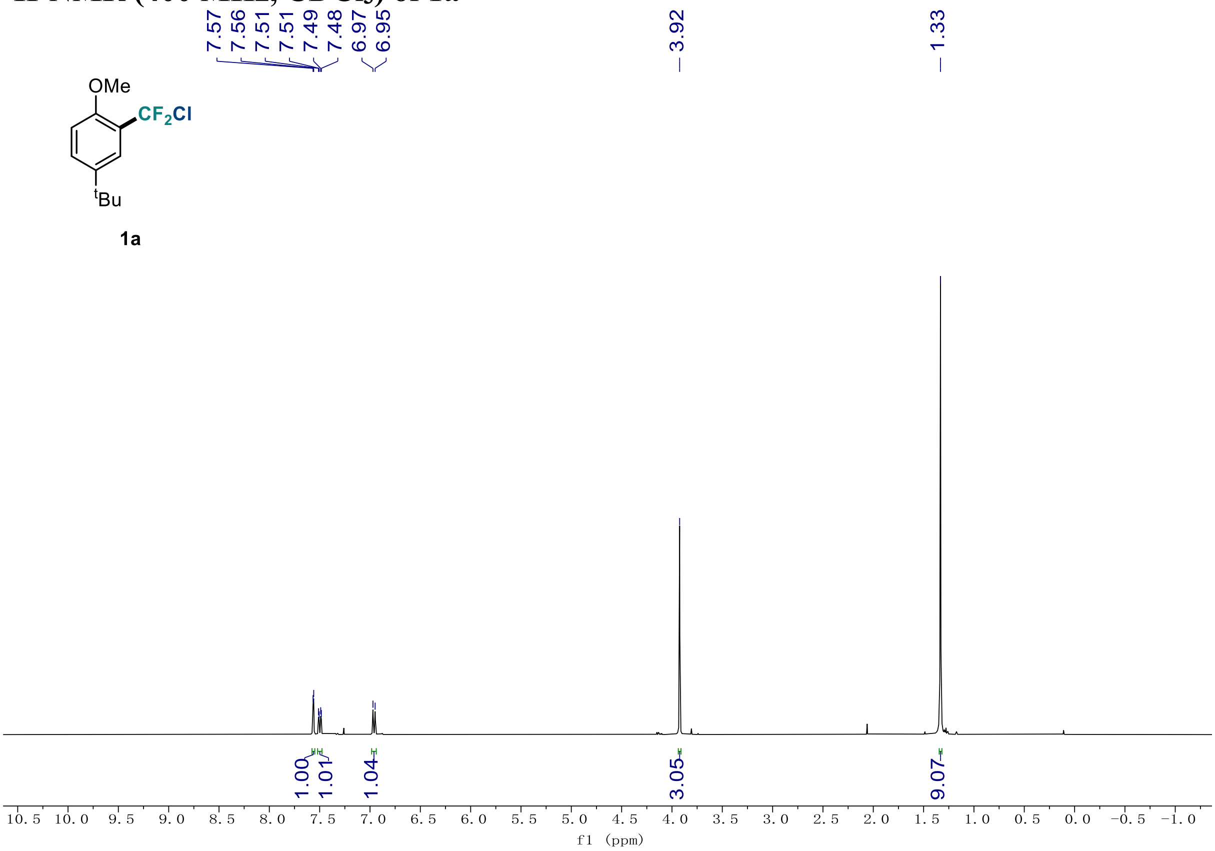

^{13}C-NMR (101 MHz, $CDCl_3$) of 1a

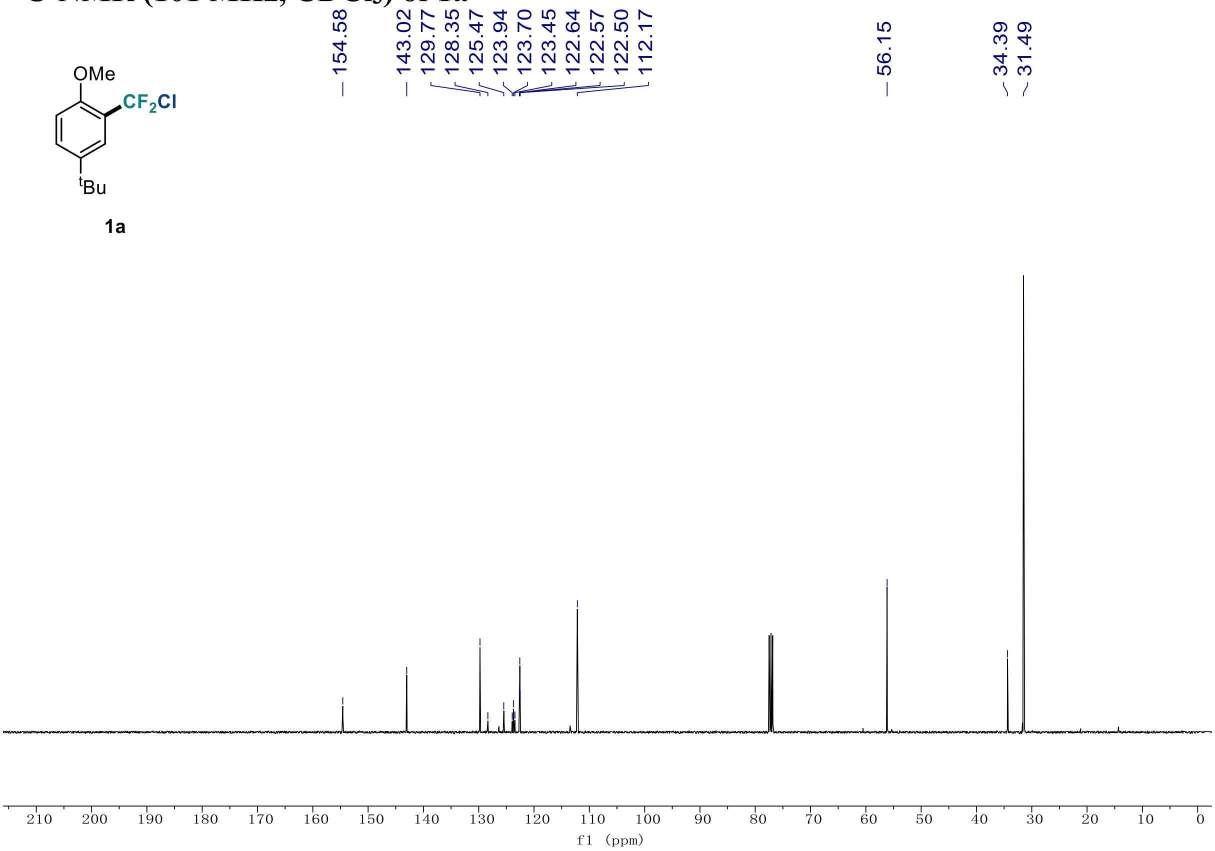

^{19}F-NMR (376 MHz, $CDCl_3$) of 1a

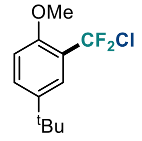

1a

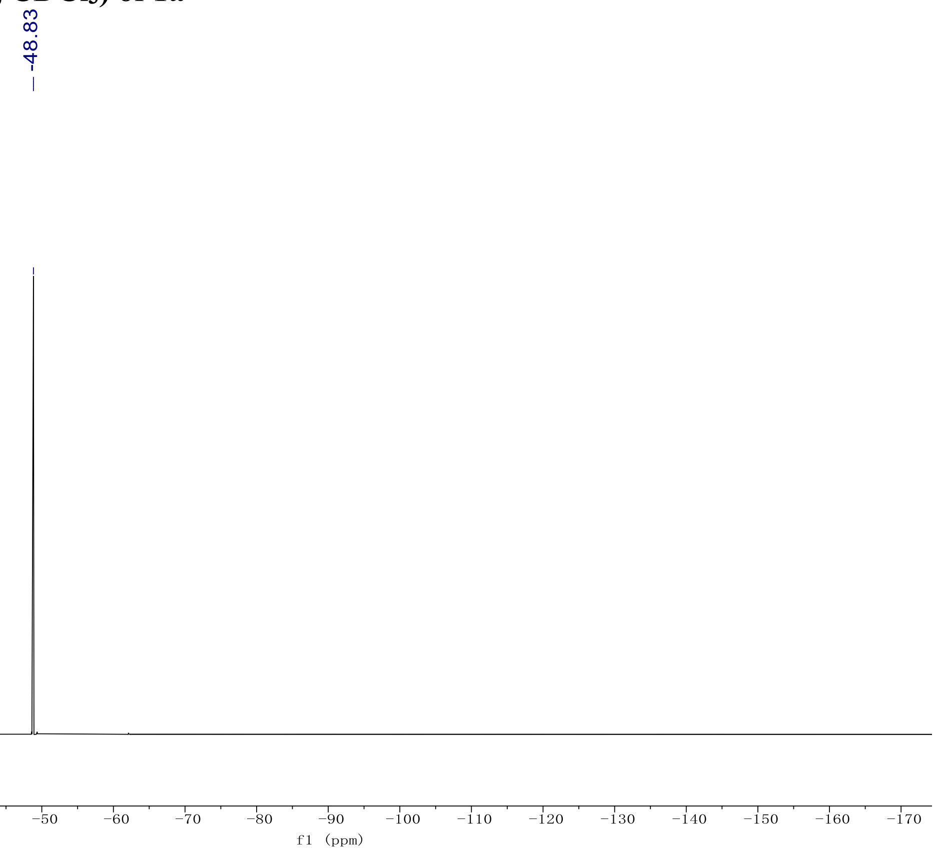

^{1}H-NMR (400 MHz, $CDCl_3$) of 3a

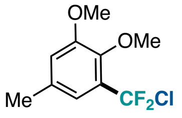

3a

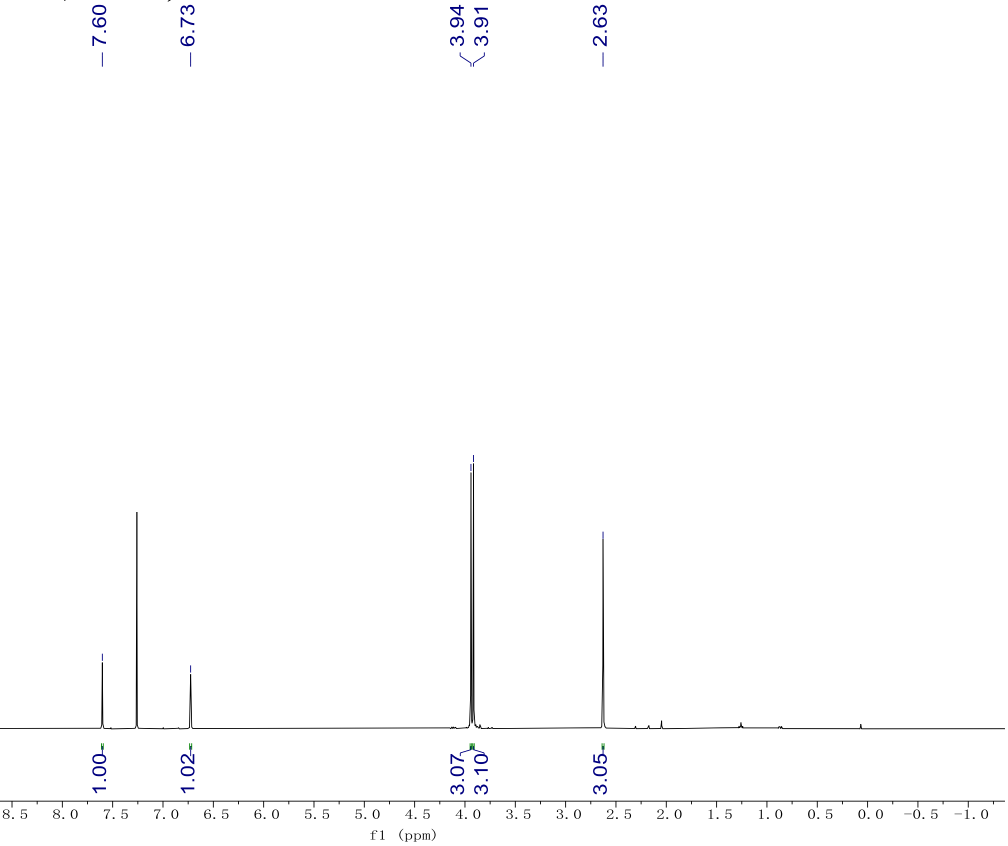

^{19}F-NMR (376 MHz, $CDCl_3$) of 3a

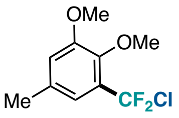

3a

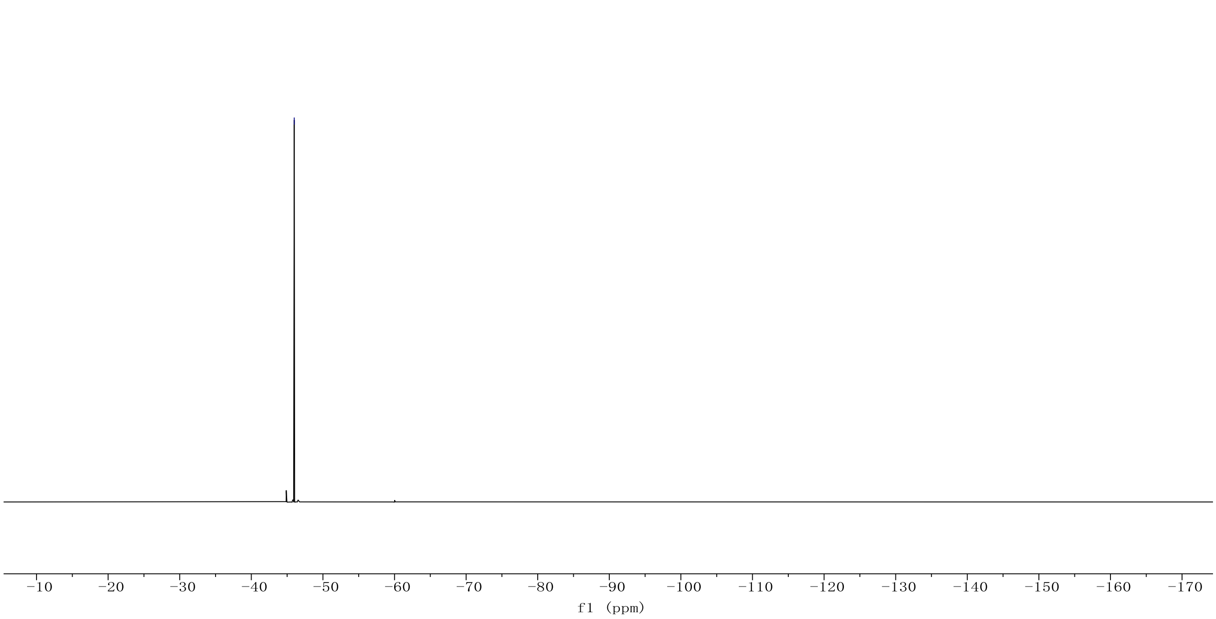

^{1}H-NMR (400 MHz, $CDCl_3$) of 5a

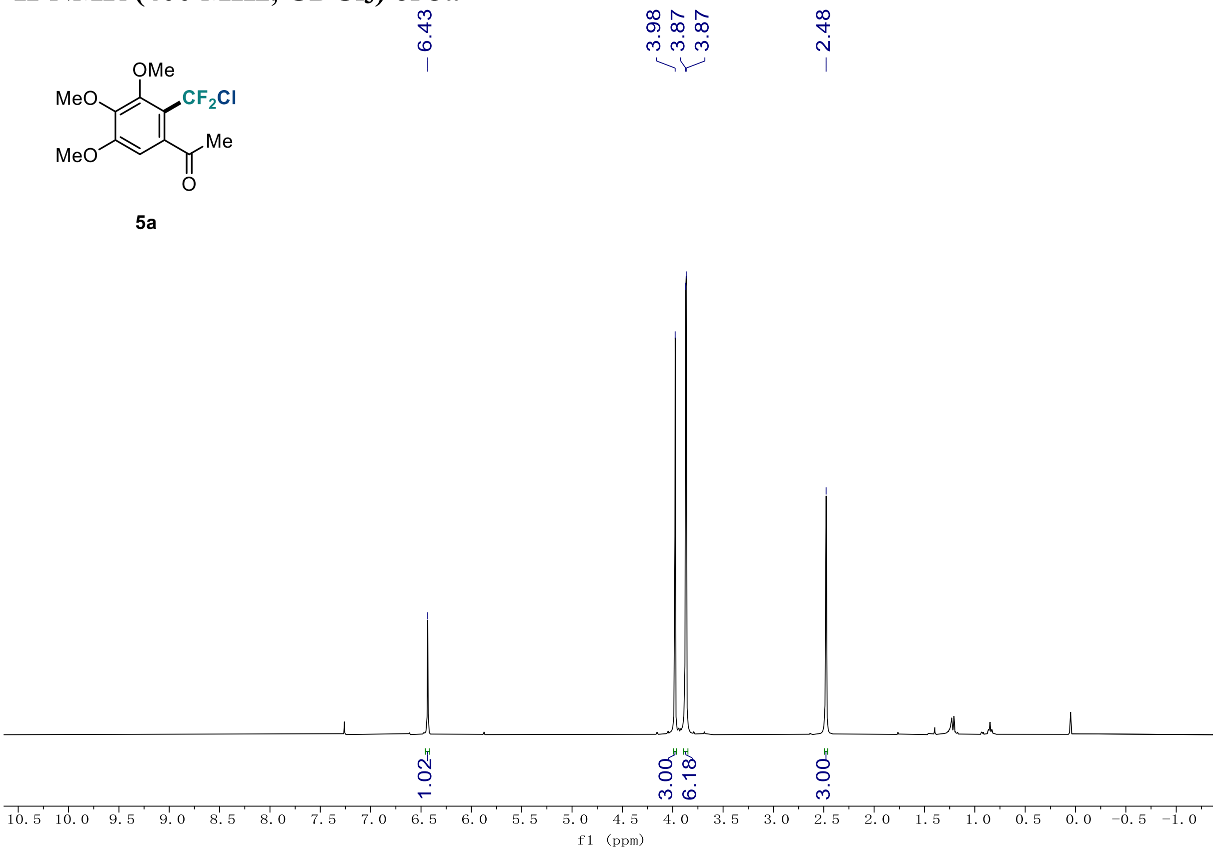

^{13}C-NMR (101 MHz, $CDCl_3$) of 5a

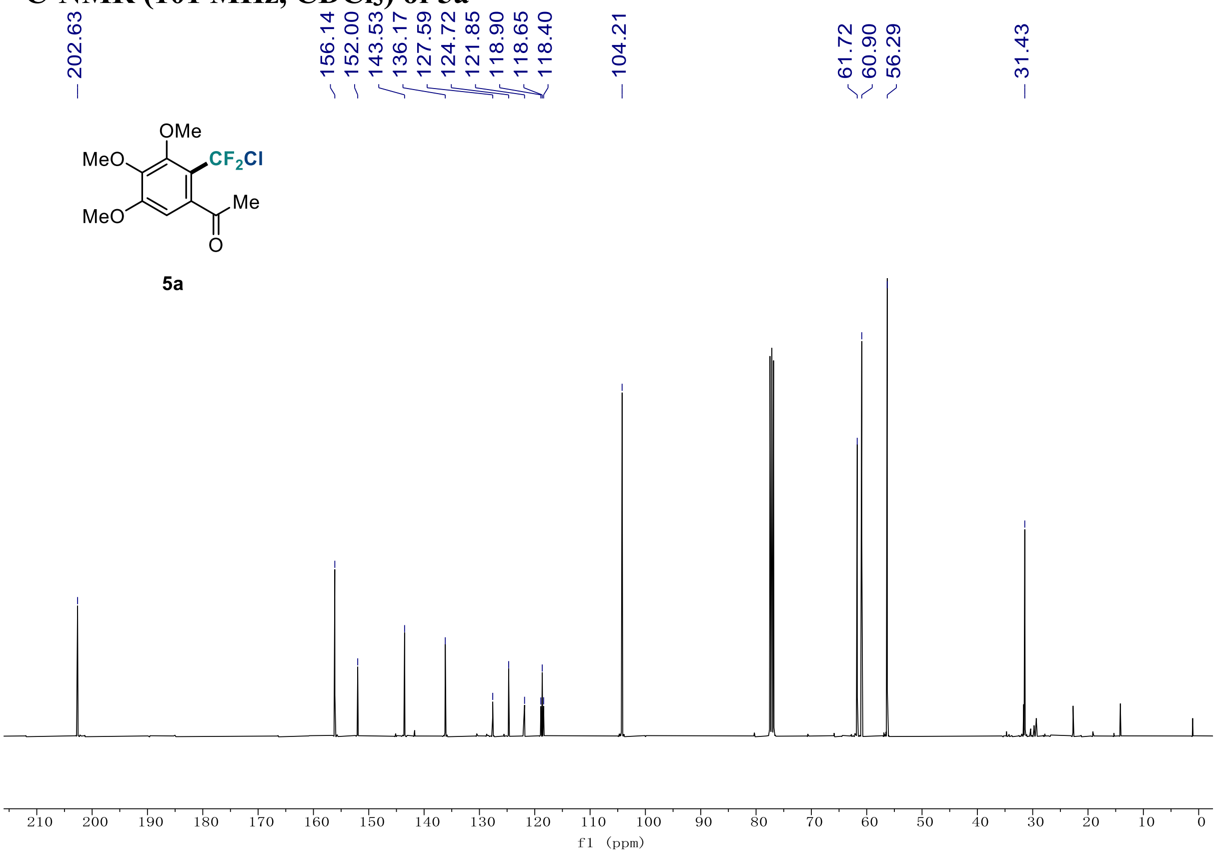

^{19}F-NMR (376 MHz, $CDCl_3$) of 5a

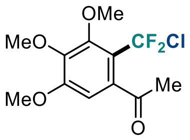

5a

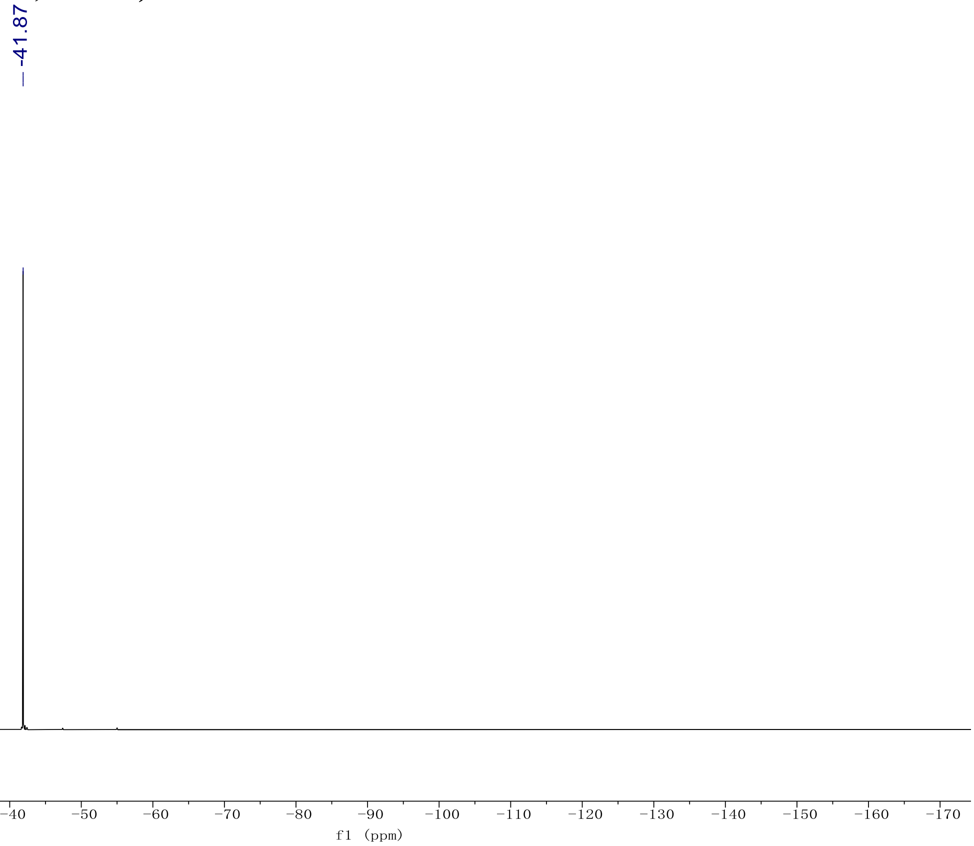

^{1}H-NMR (400 MHz, $CDCl_3$) of 7a

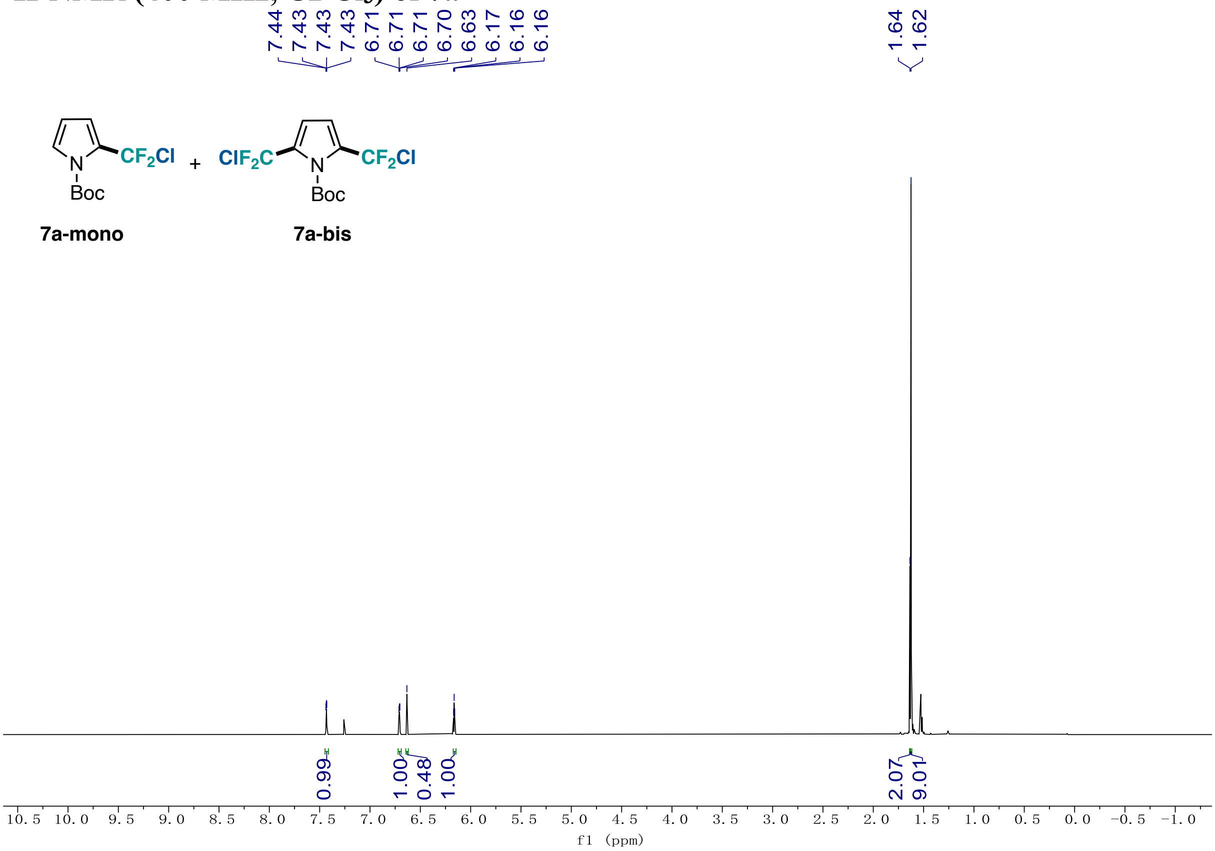

^{13}C-NMR (101 MHz, $CDCl_3$) of 7a

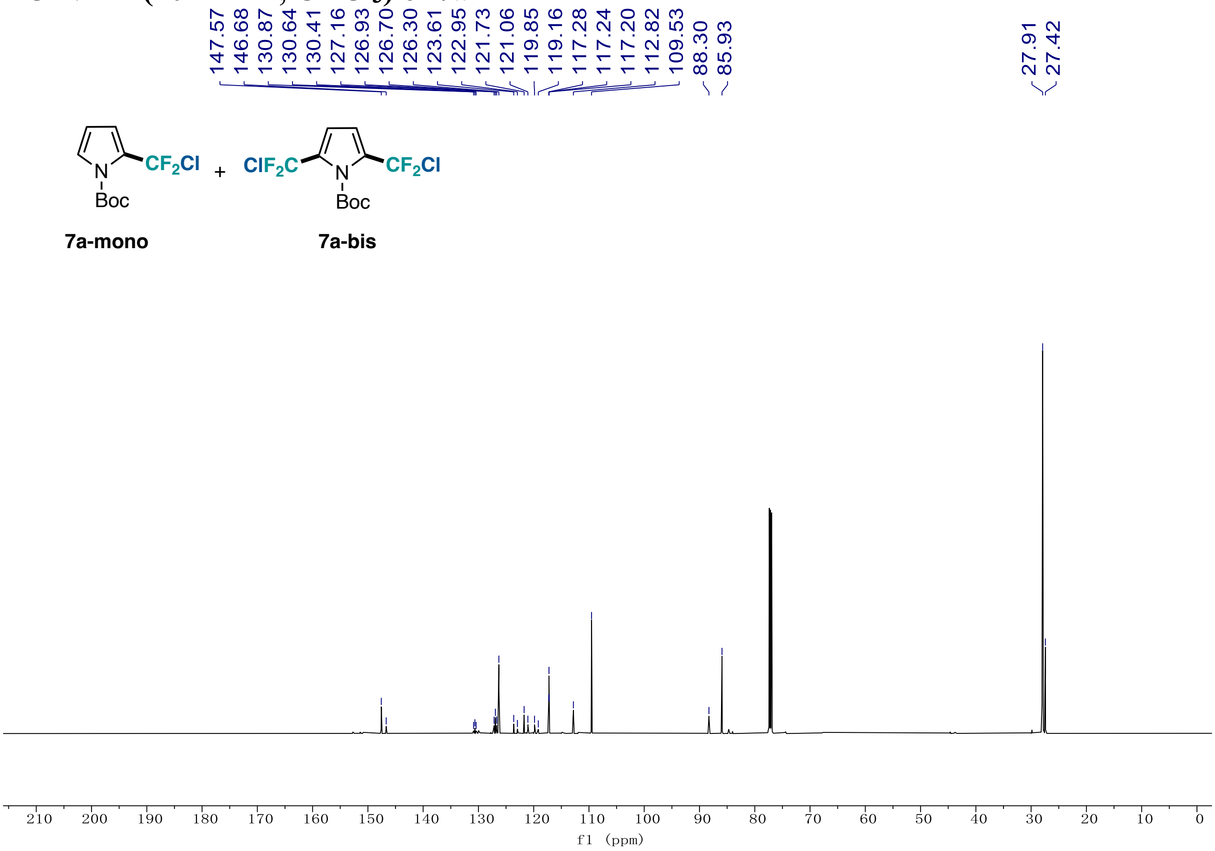

^{19}F-NMR (376 MHz, CDCl3) of 7a

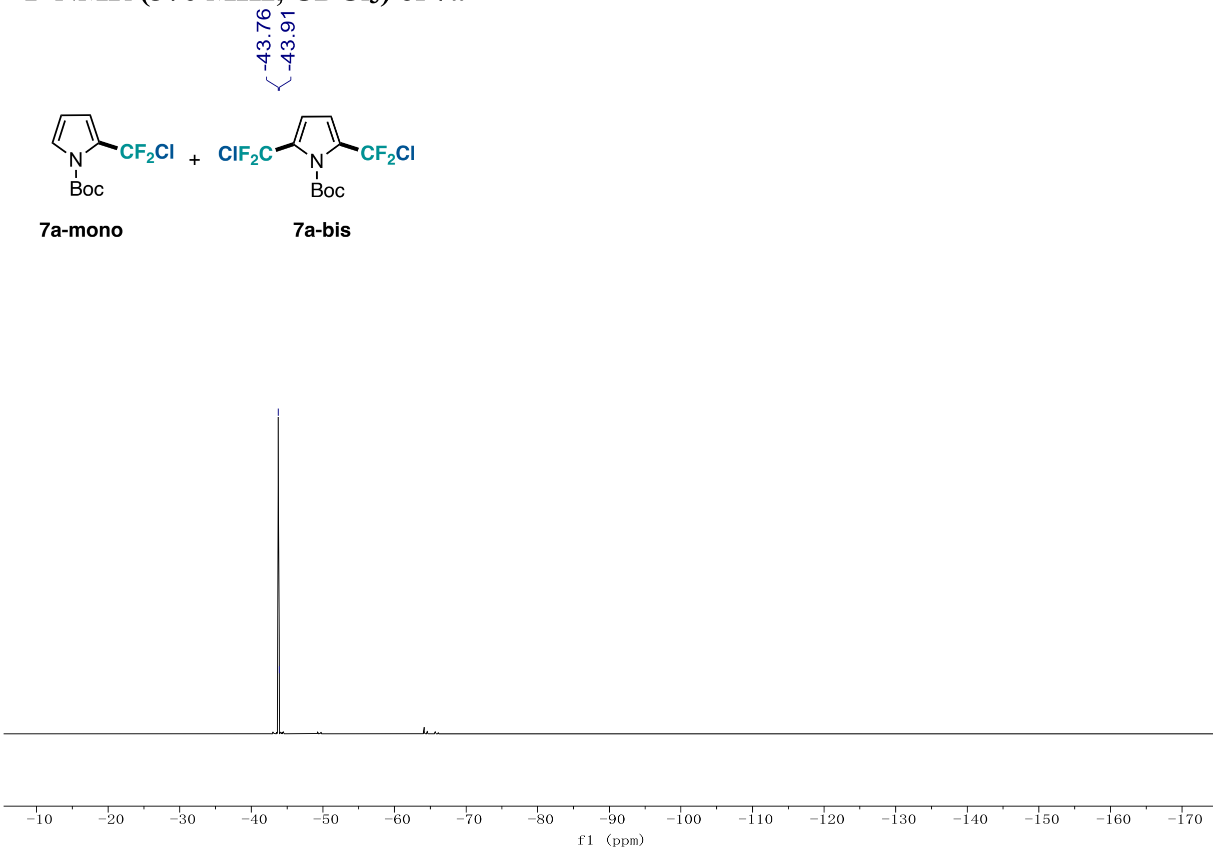

^{1}H-NMR (400 MHz, $CDCl_3$) of 8a

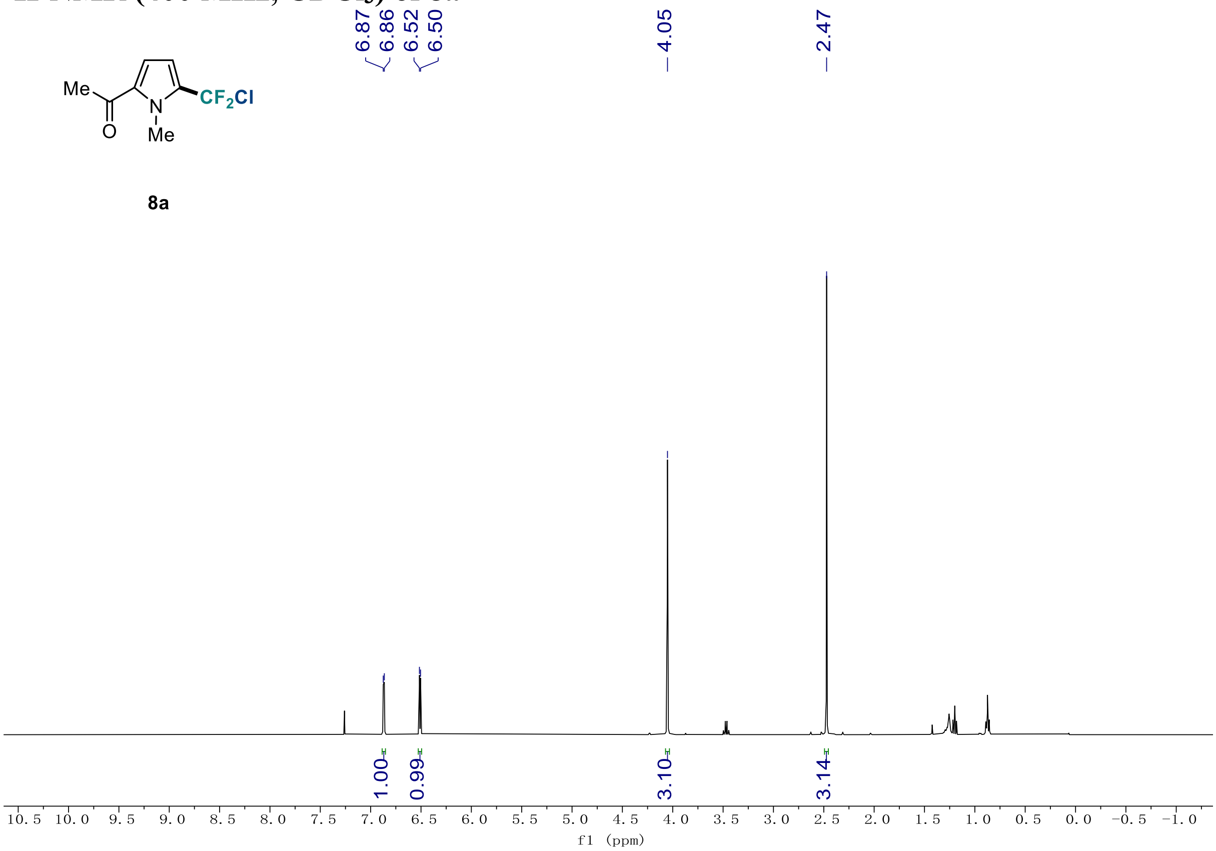

^{13}C-NMR (101 MHz, $CDCl_3$) of 8a

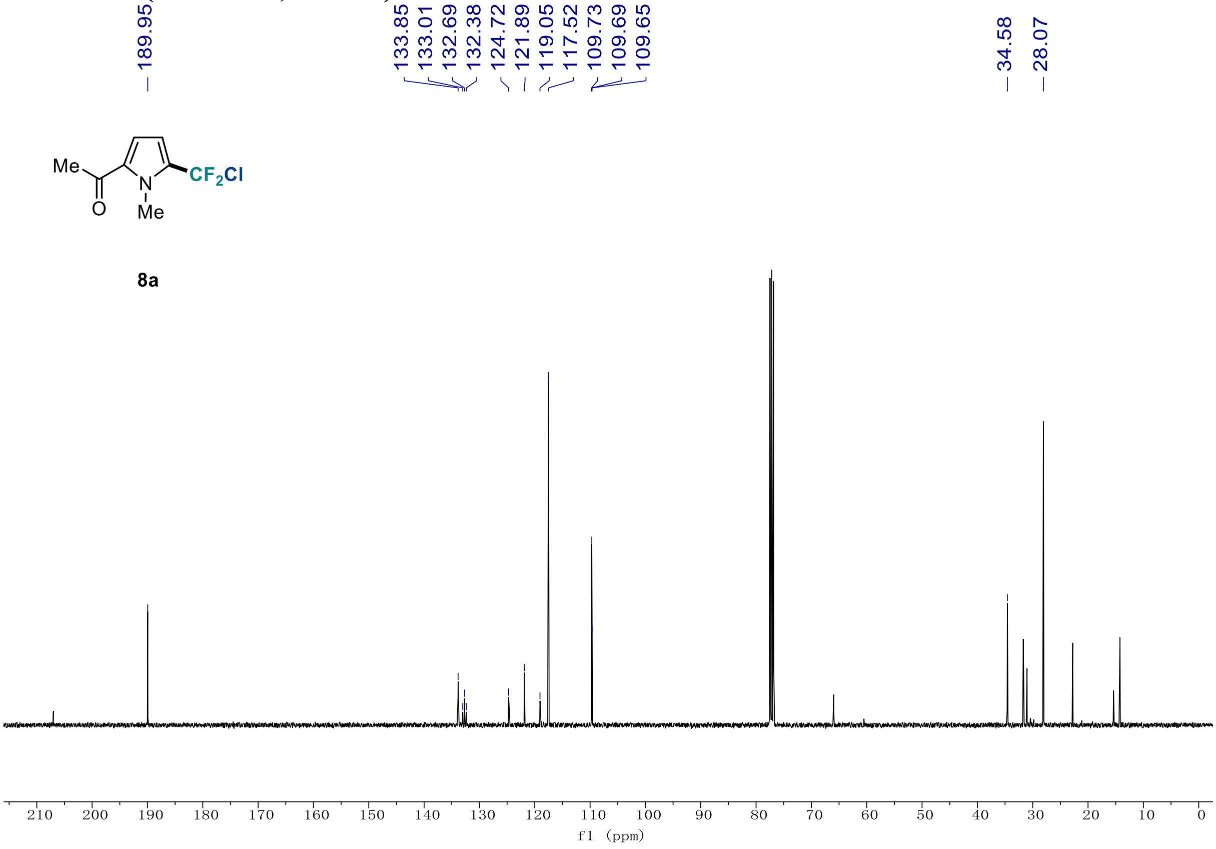

^{19}F-NMR (376 MHz, $CDCl_3$) of 8a

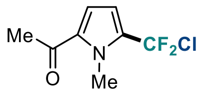

8a

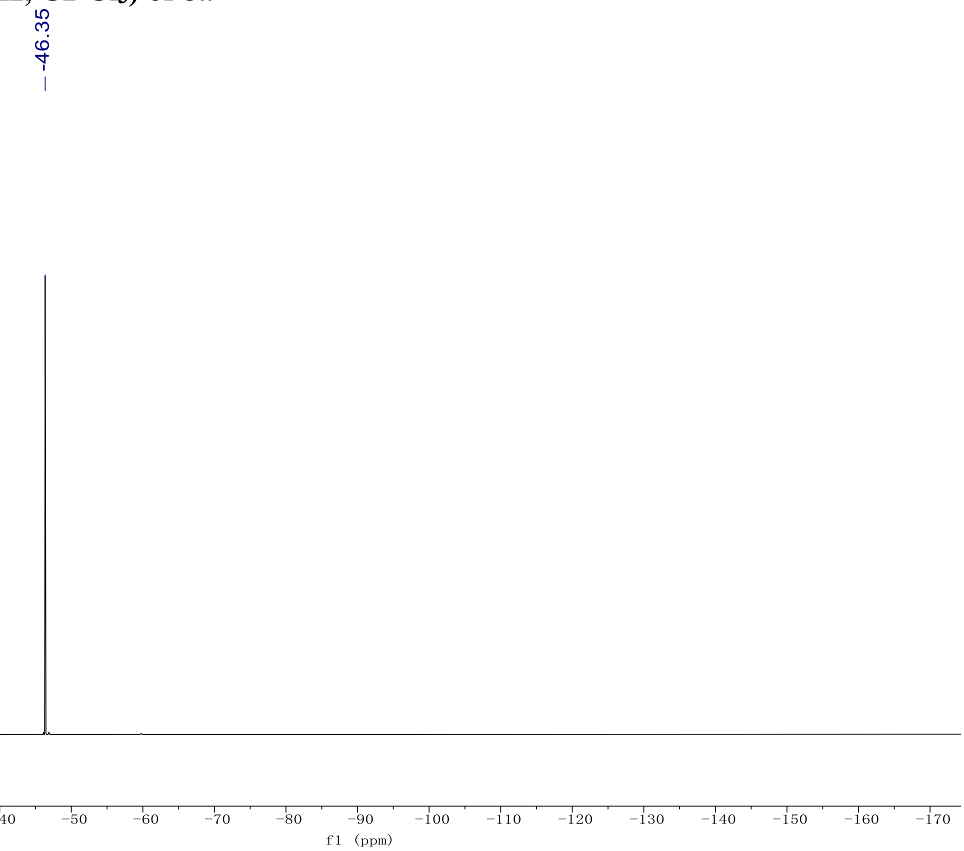

^{1}H-NMR (400 MHz, $CDCl_3$) of 9a

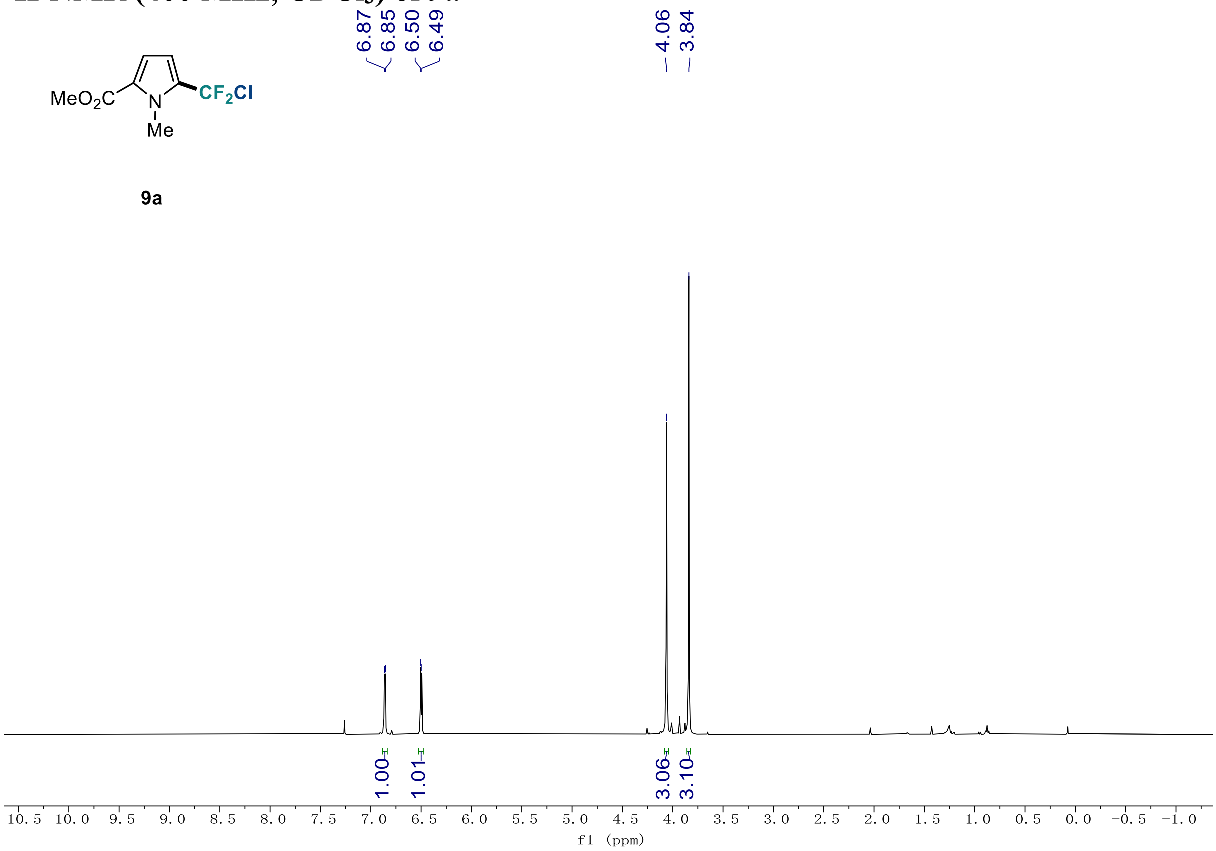

9a

^{13}C-NMR (101 MHz, $CDCl_3$) of 9a

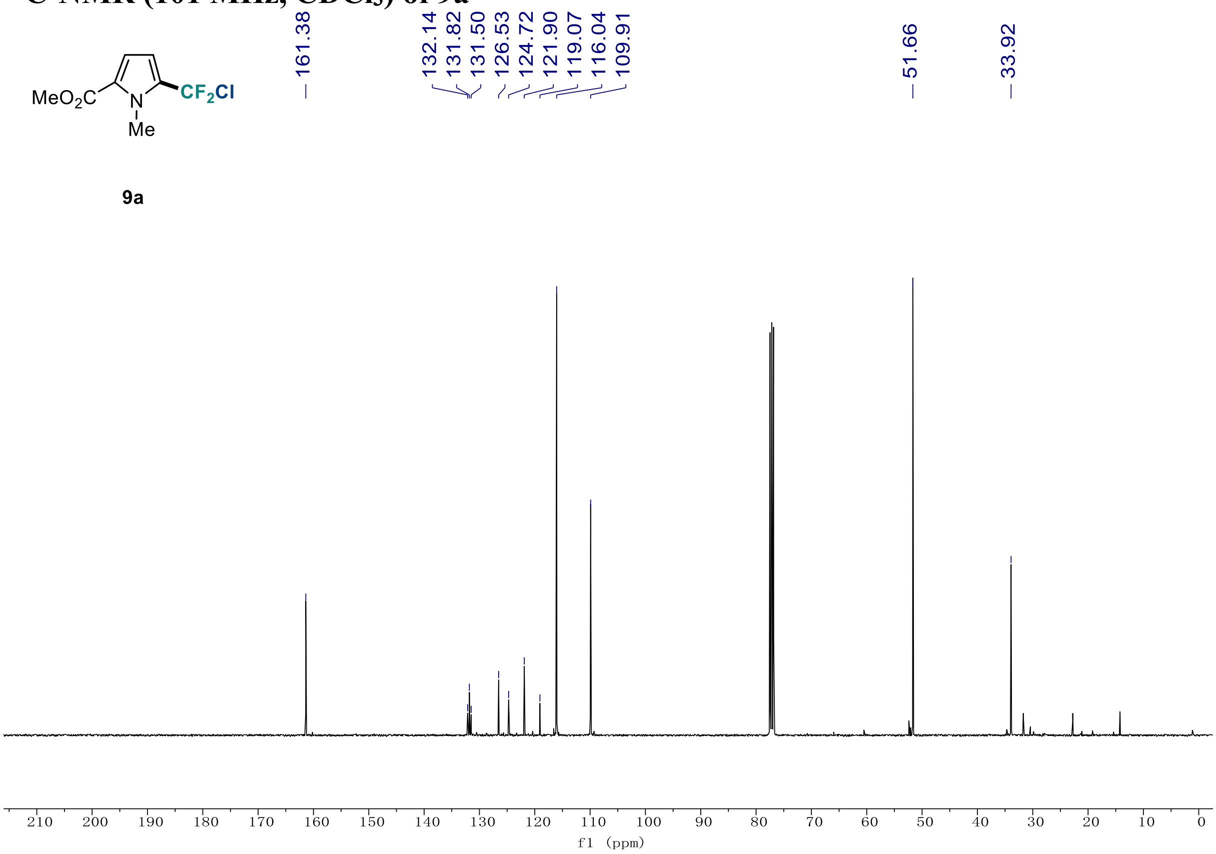

9a

^{19}F-NMR (376 MHz, $CDCl_3$) of 9a

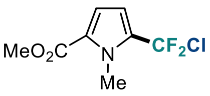

9a

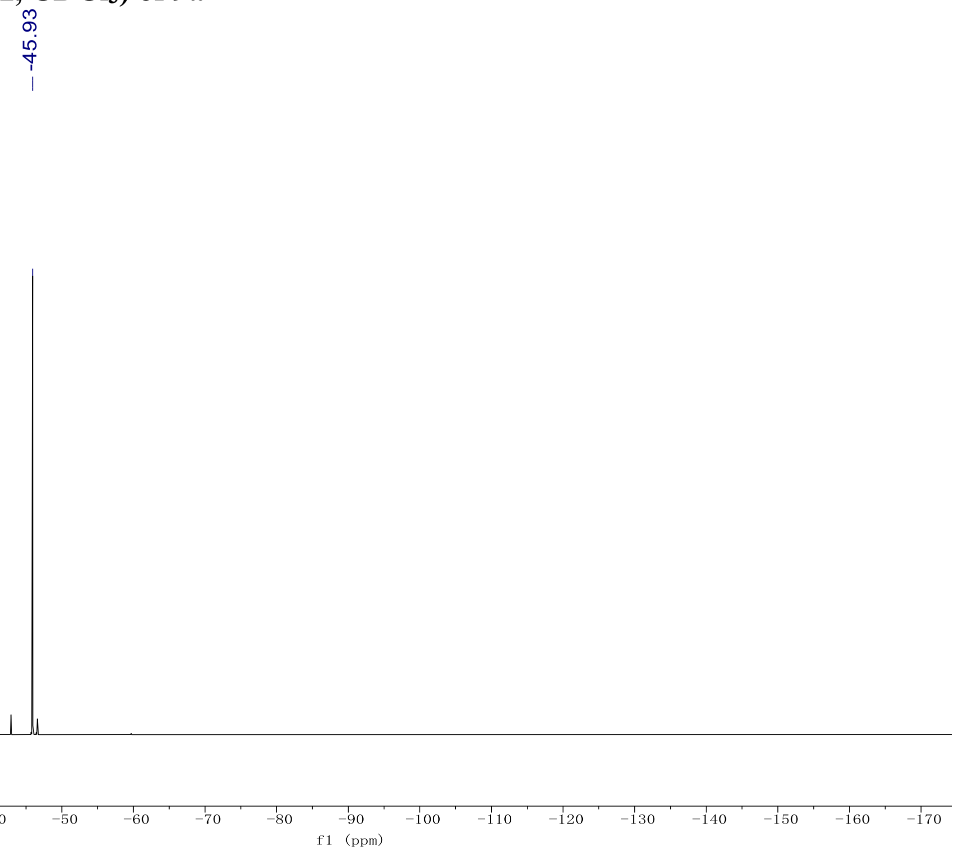

^{1}H-NMR (400 MHz, $CDCl_3$) of 10a

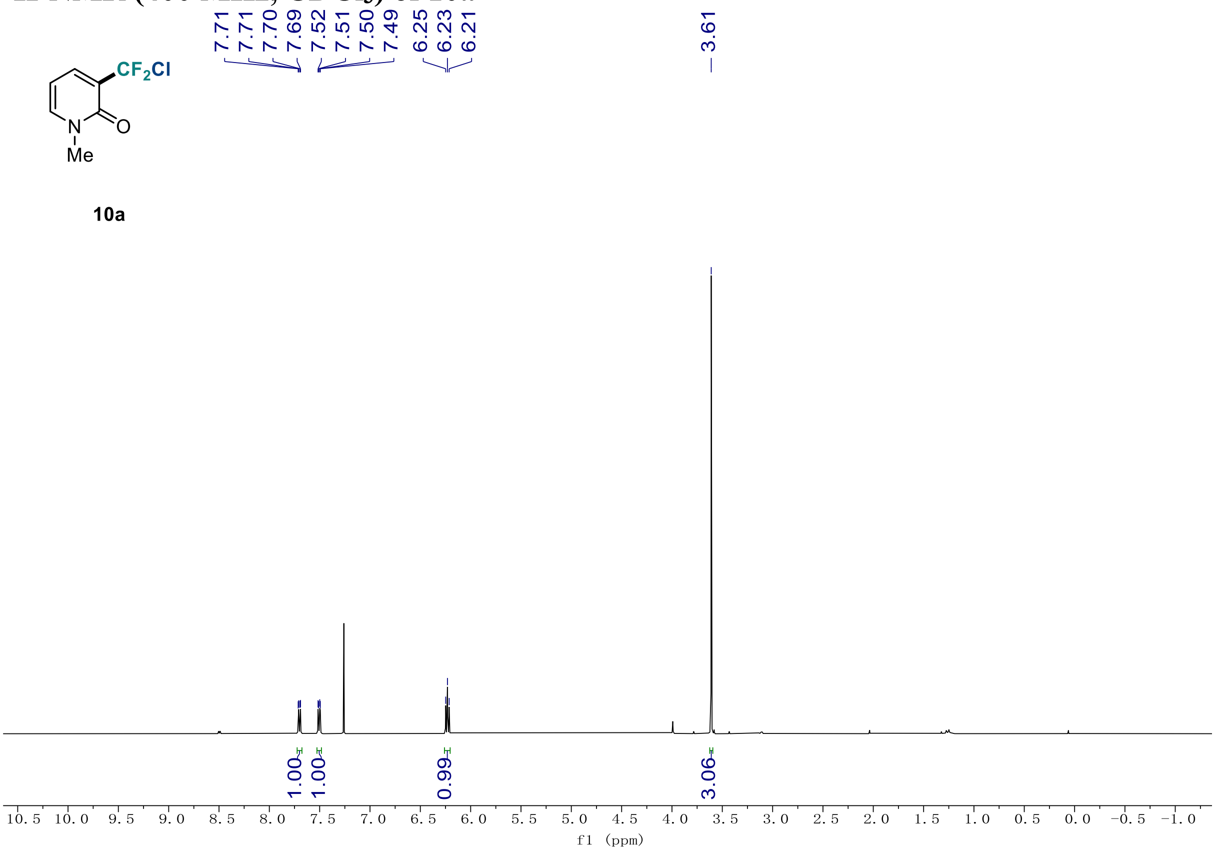

^{13}C-NMR (101 MHz, $CDCl_3$) of 10a

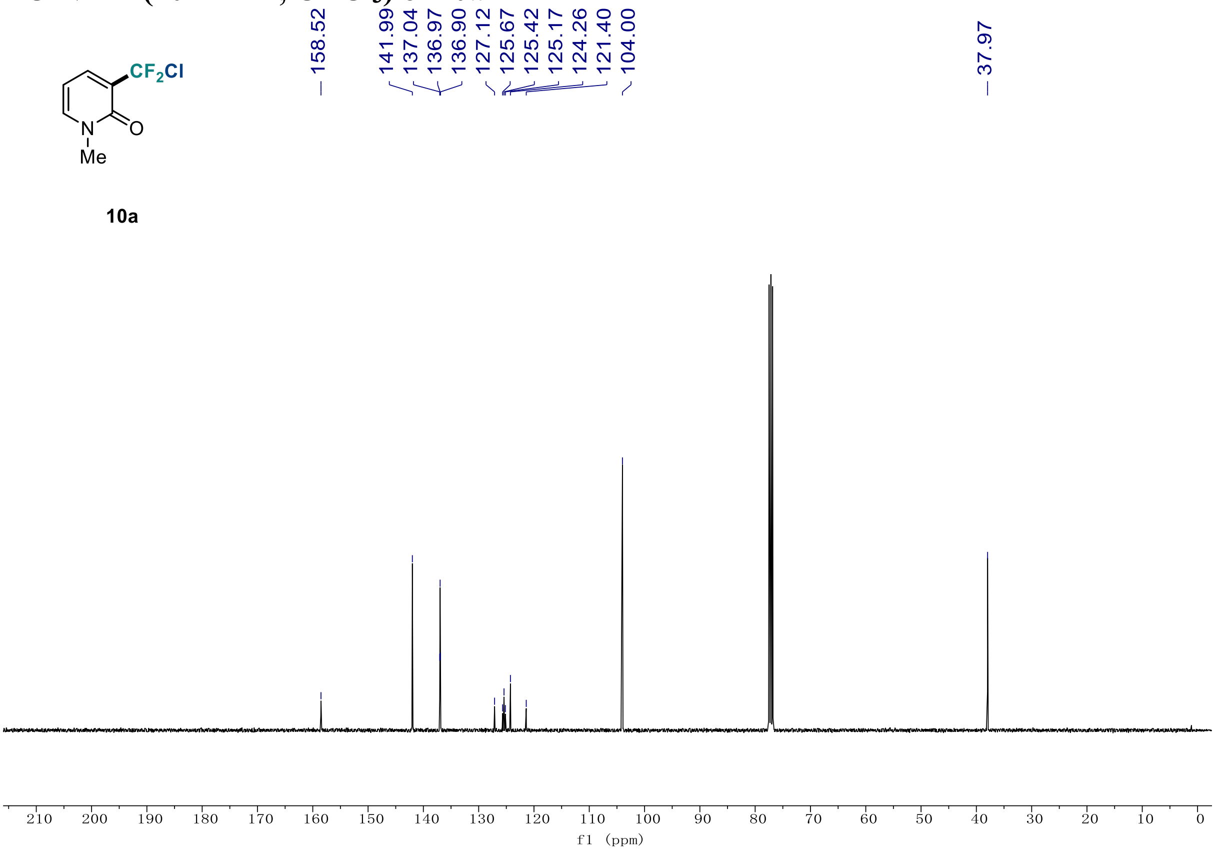

^{19}F-NMR (376 MHz, $CDCl_3$) of 10a

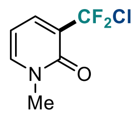

10a

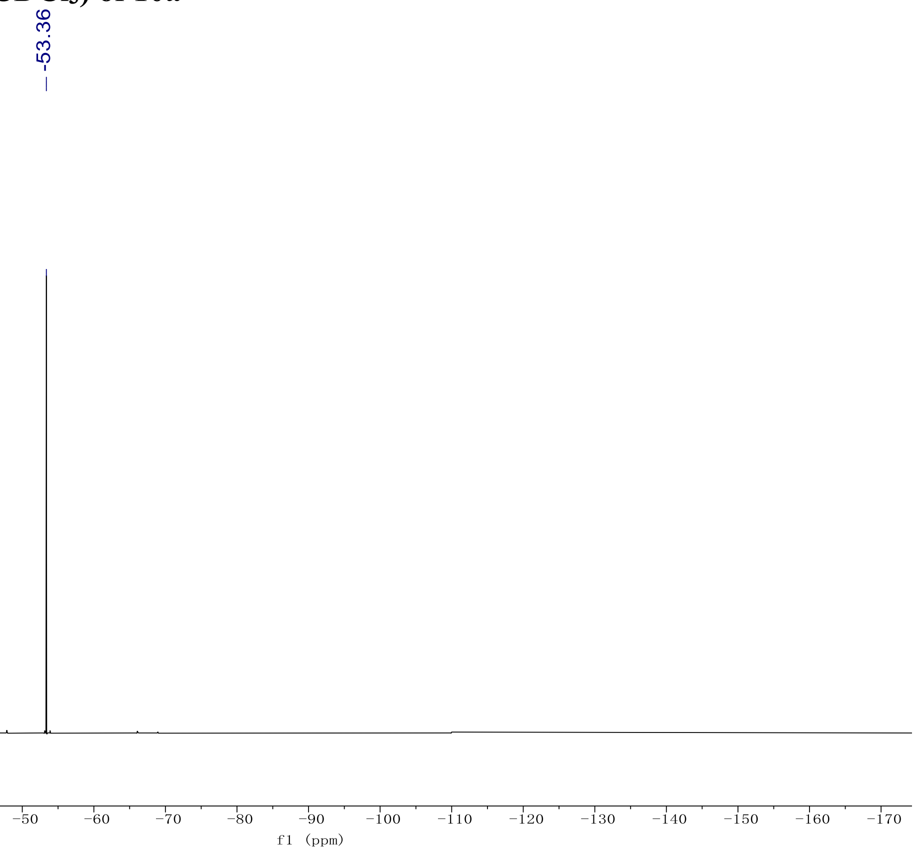

^{1}H-NMR (400 MHz, $CDCl_3$) of 11a

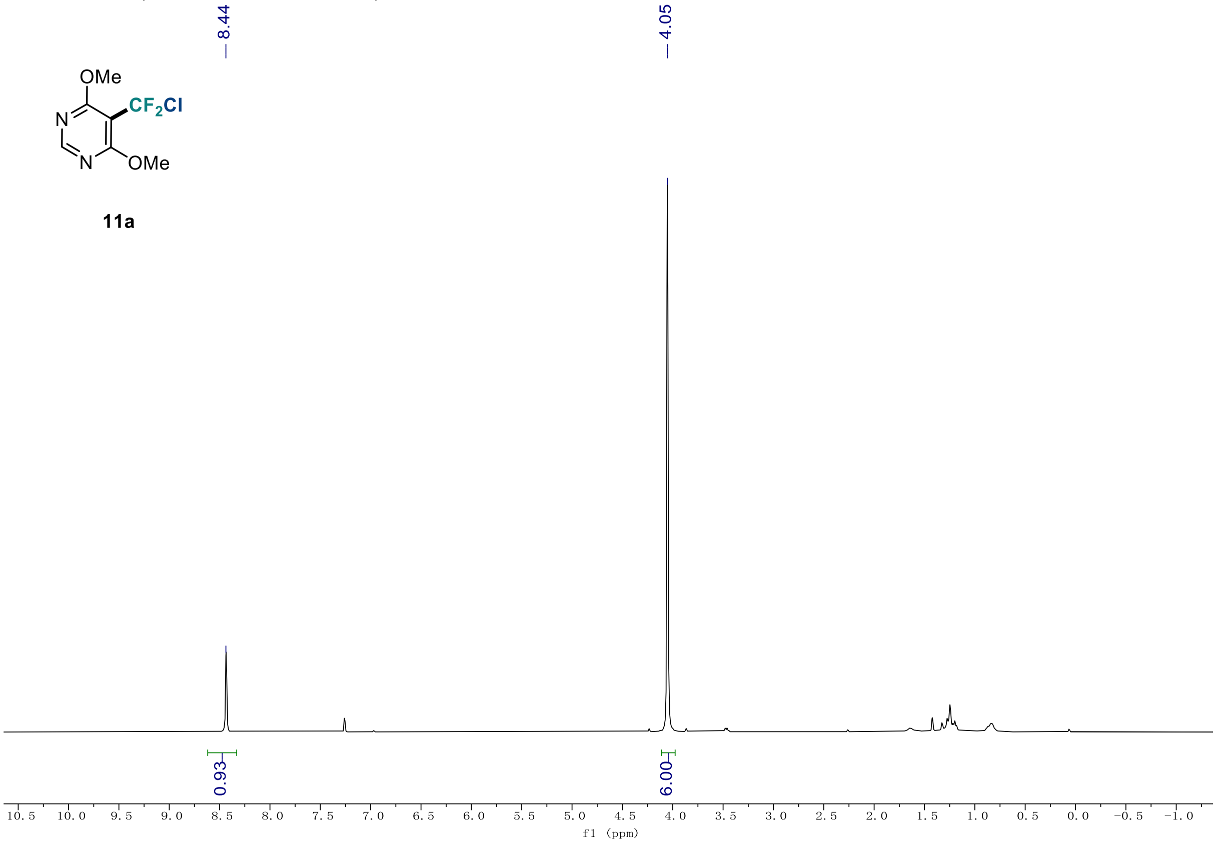

^{13}C-NMR (75 MHz, $CDCl_3$) of 11a

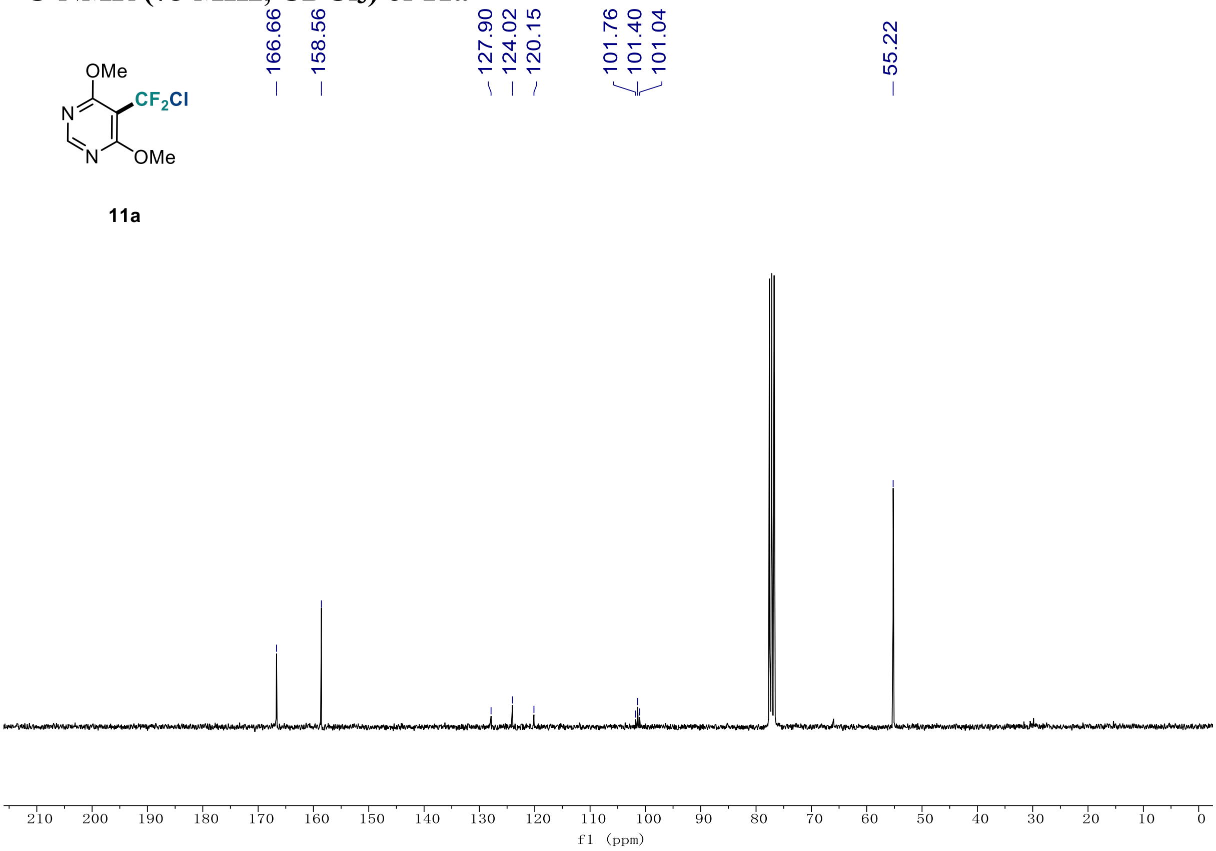

^{19}F-NMR (376 MHz, $CDCl_3$) of 11a

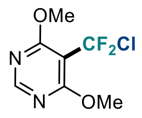

11a

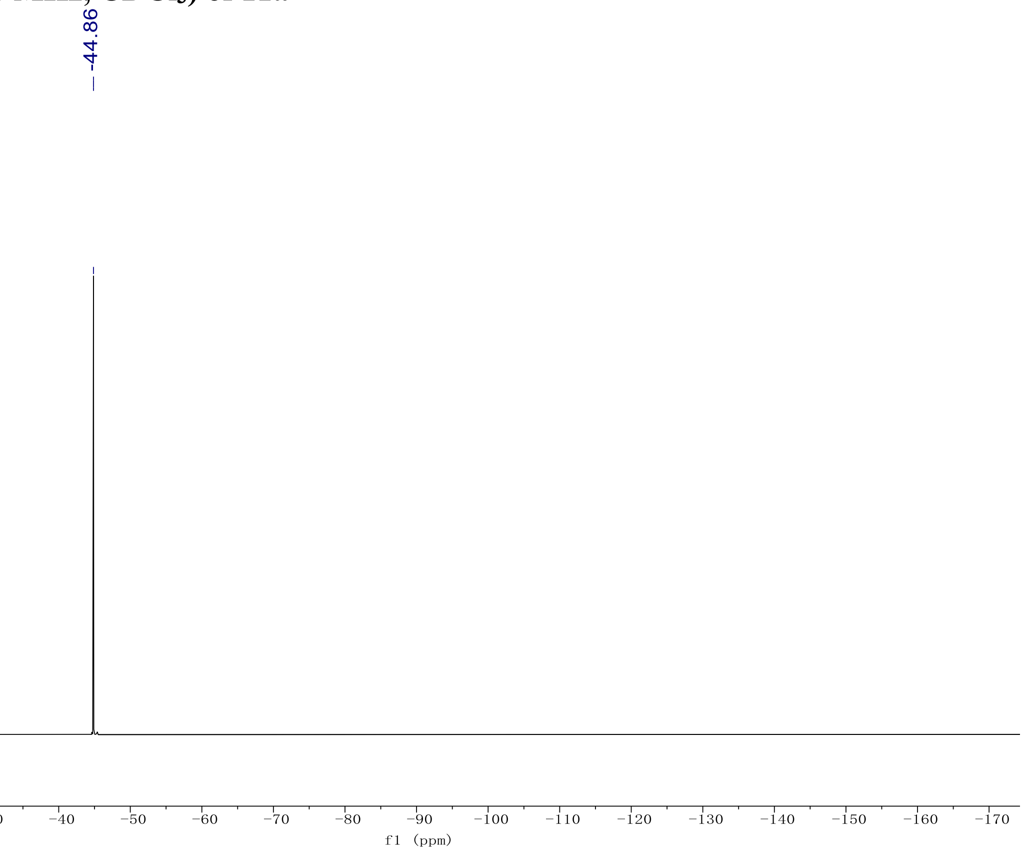

^{1}H-NMR (600 MHz, $CDCl_3$) of 12a

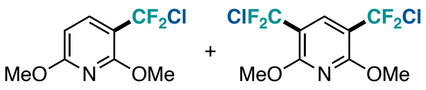

12a-mono **12a-bis**

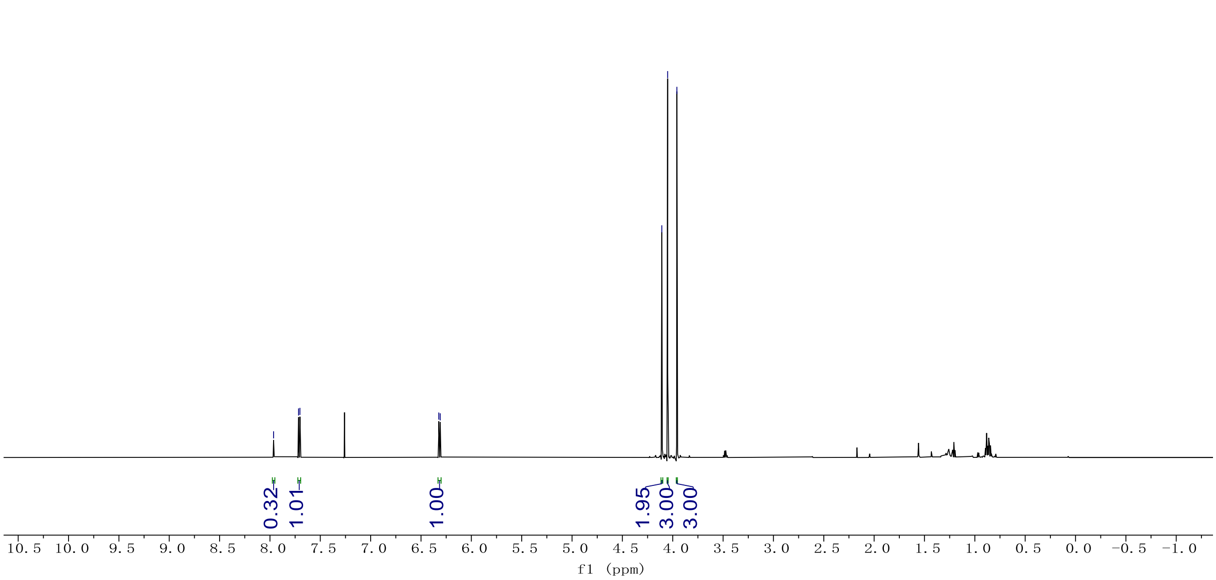

^{13}C-NMR (151 MHz, $CDCl_3$) of 12a

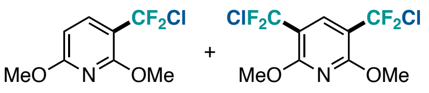

12a-mono **12a-bis**

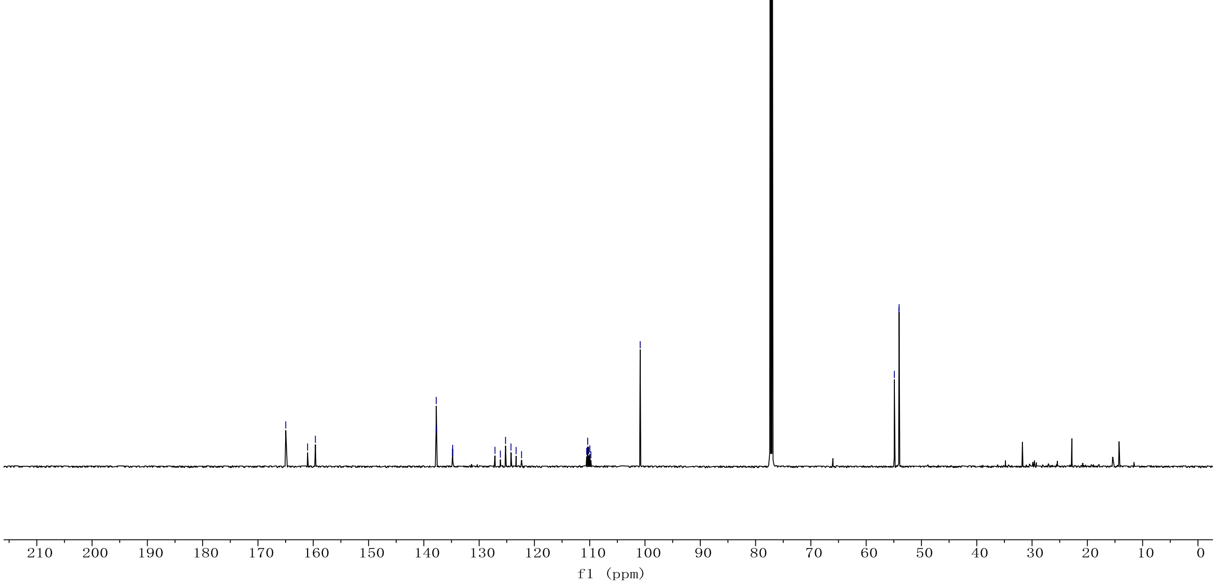

^{19}F-NMR (376 MHz, $CDCl_3$) of 12a

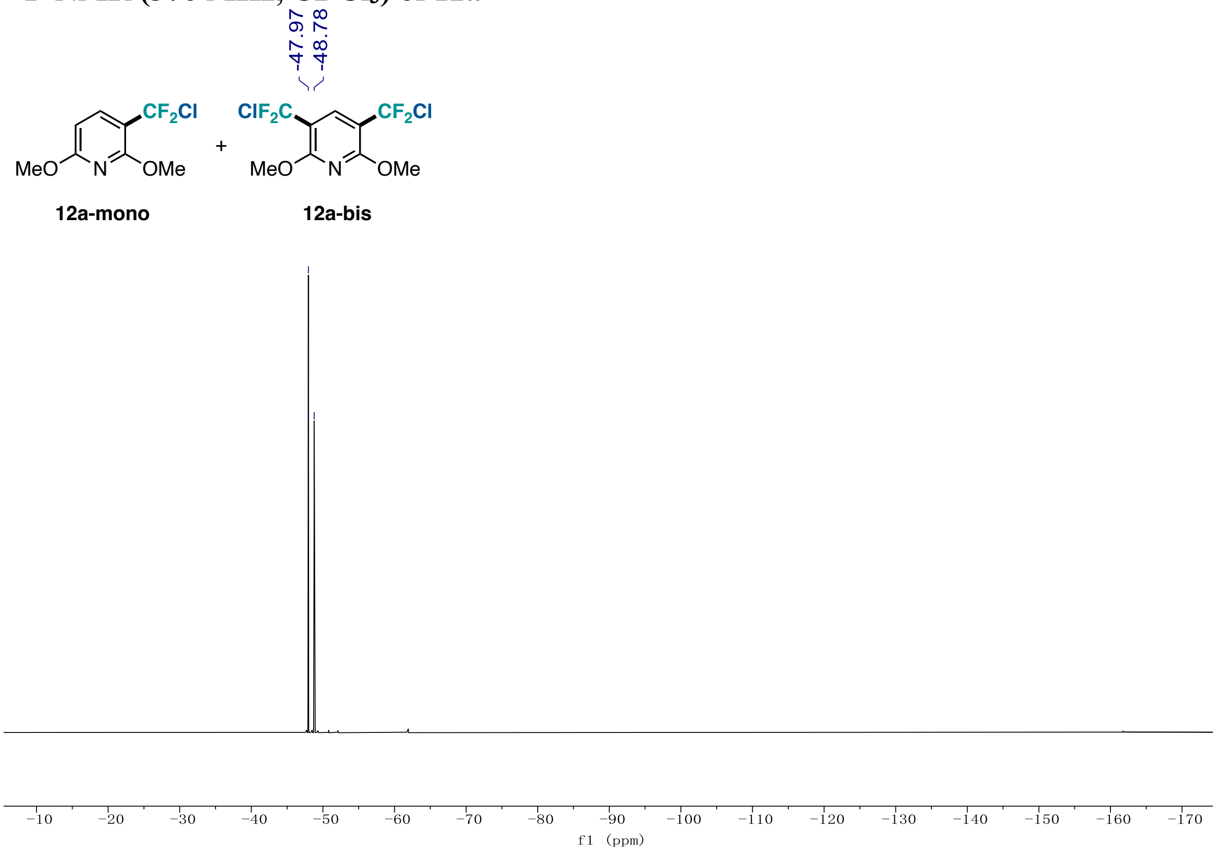

^{1}H-NMR (400 MHz, $CDCl_3$) of 13a

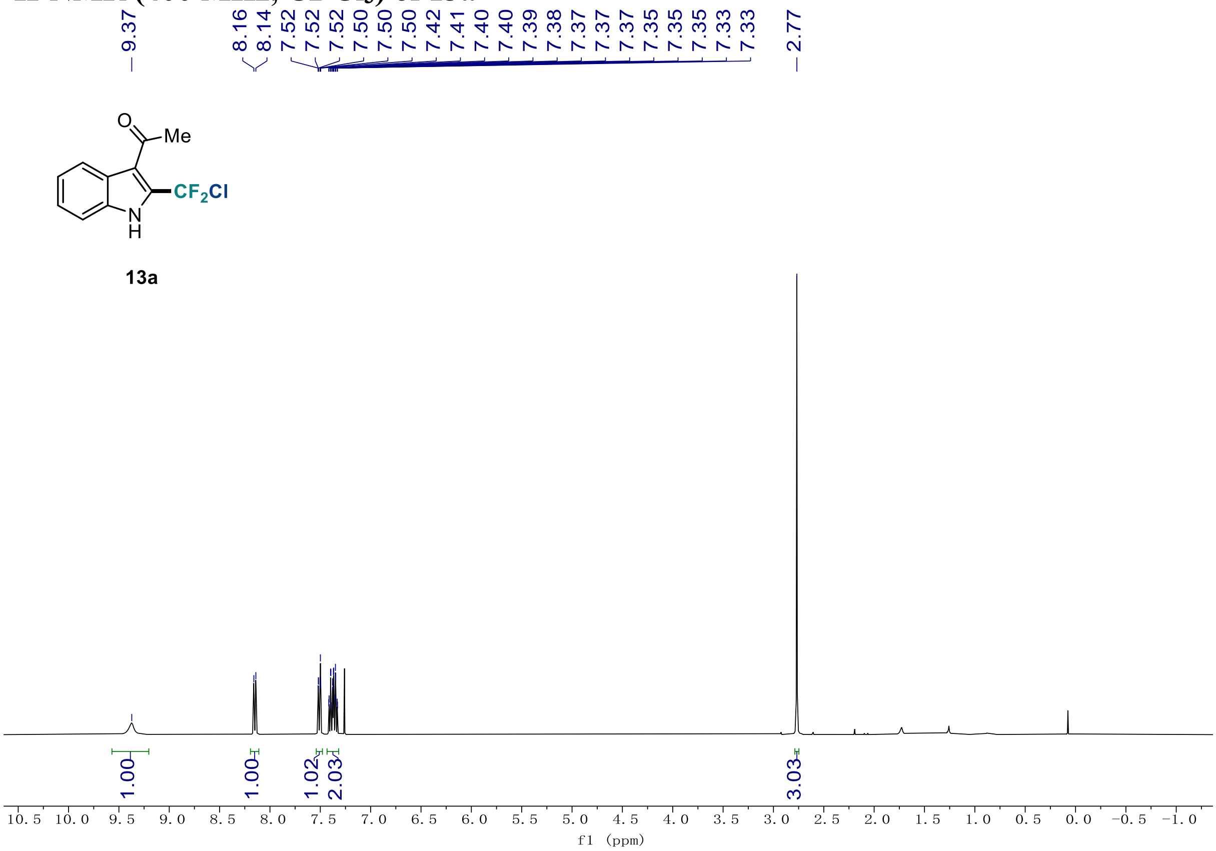

^{13}C-NMR (75 MHz, $CDCl_3$) of 13a

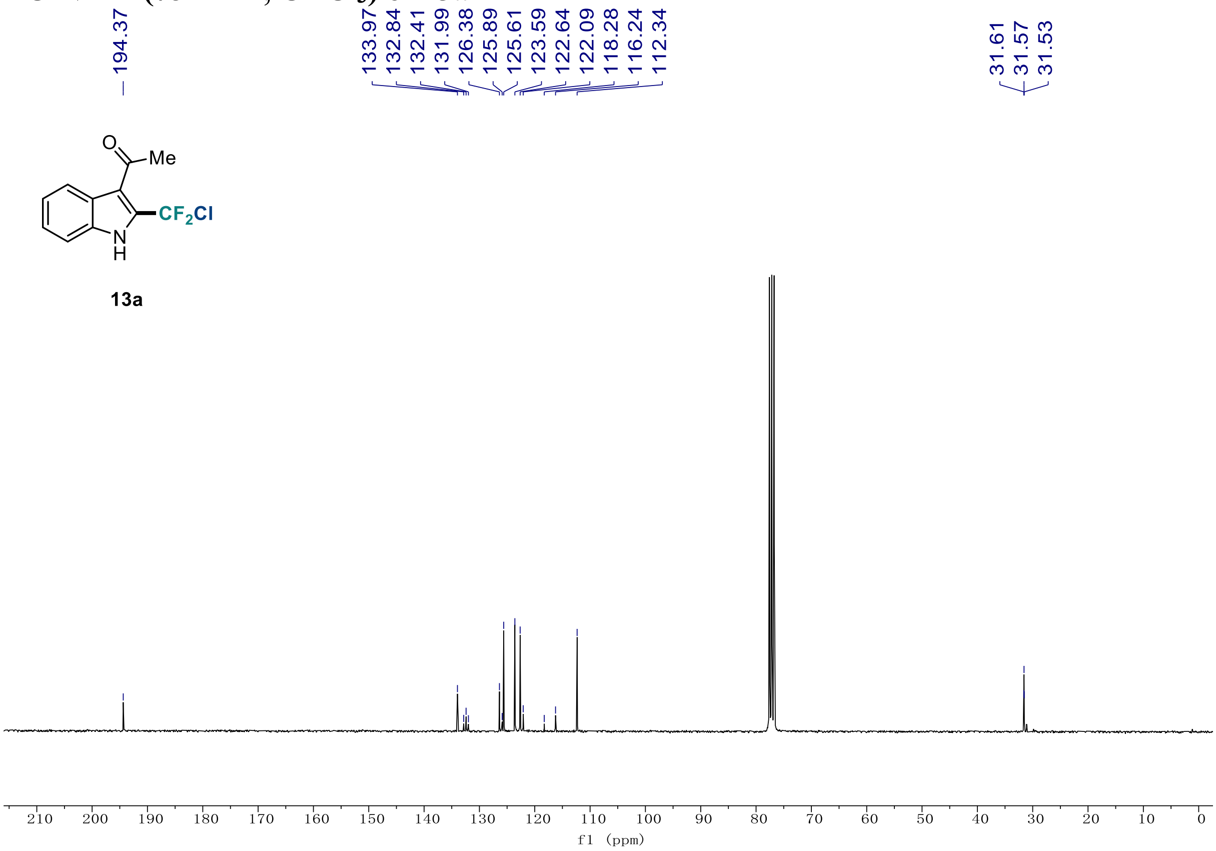

^{19}F-NMR (376 MHz, CDCl$_3$) of 13a

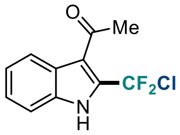

13a

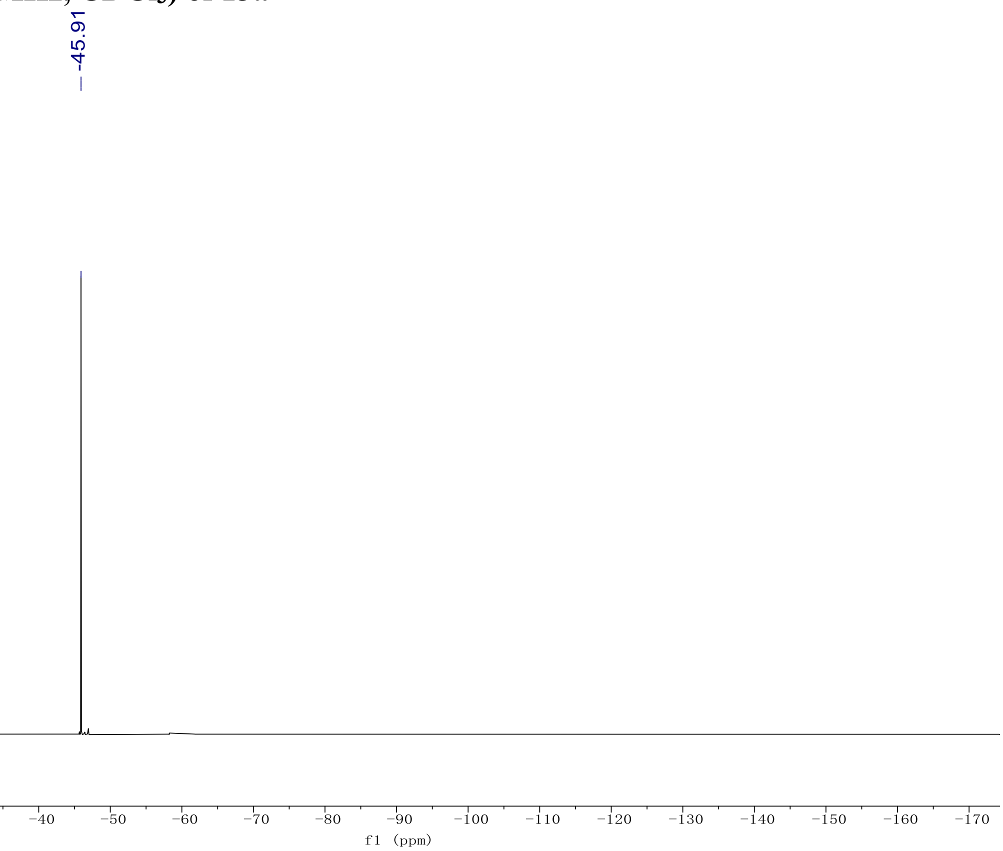

^{1}H-NMR (400 MHz, $CDCl_3$) of 14a-mono

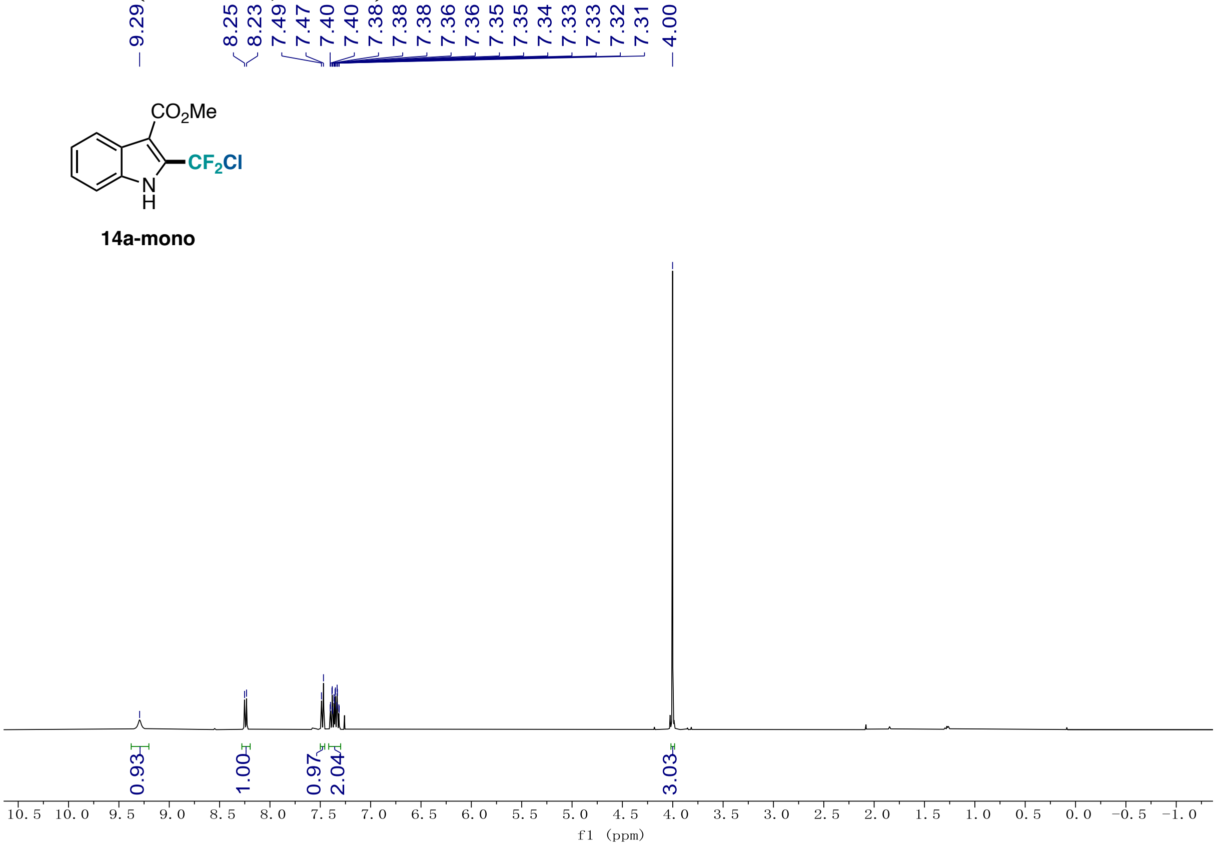

^{13}C-NMR (75 MHz, $CDCl_3$) of 14a-mono

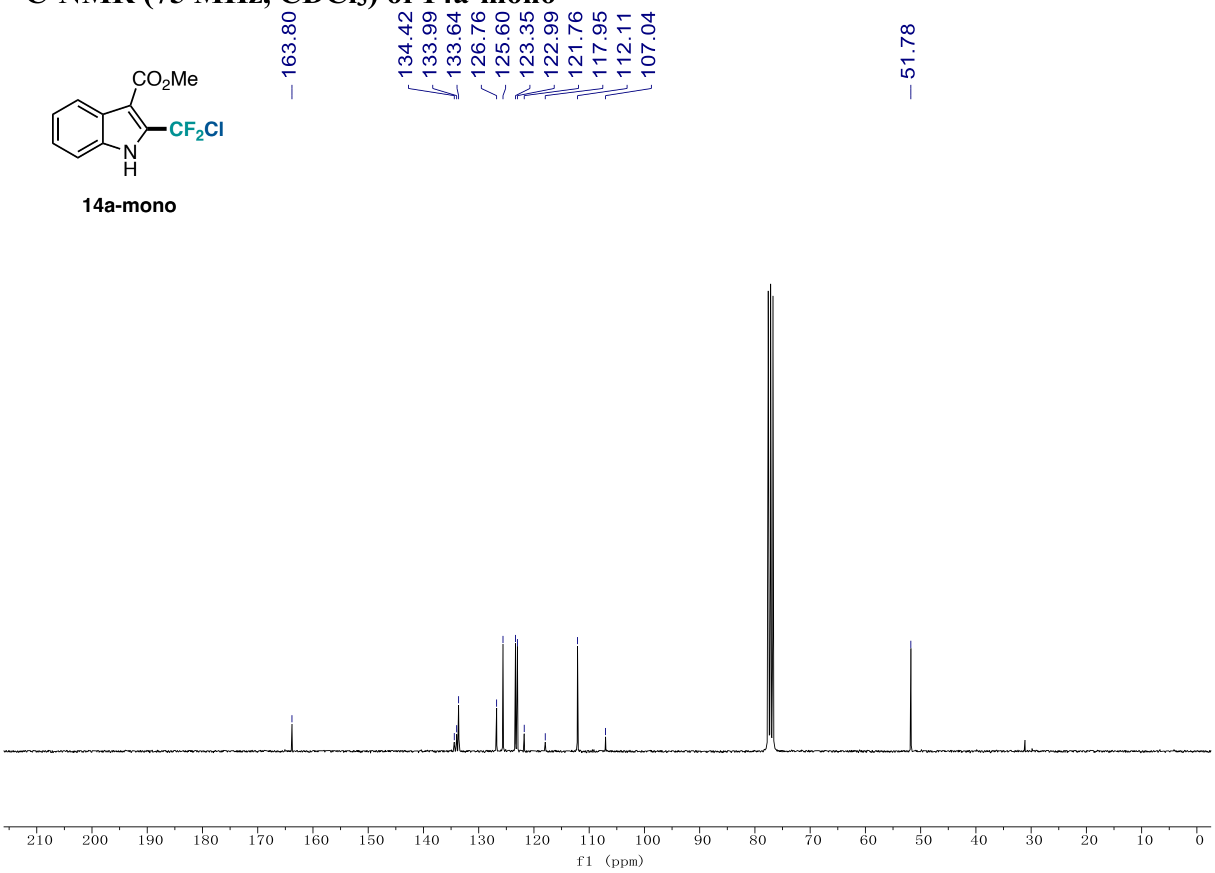

^{19}F-NMR (376 MHz, $CDCl_3$) of 14a-mono

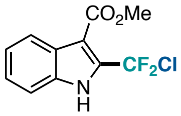

14a-mono

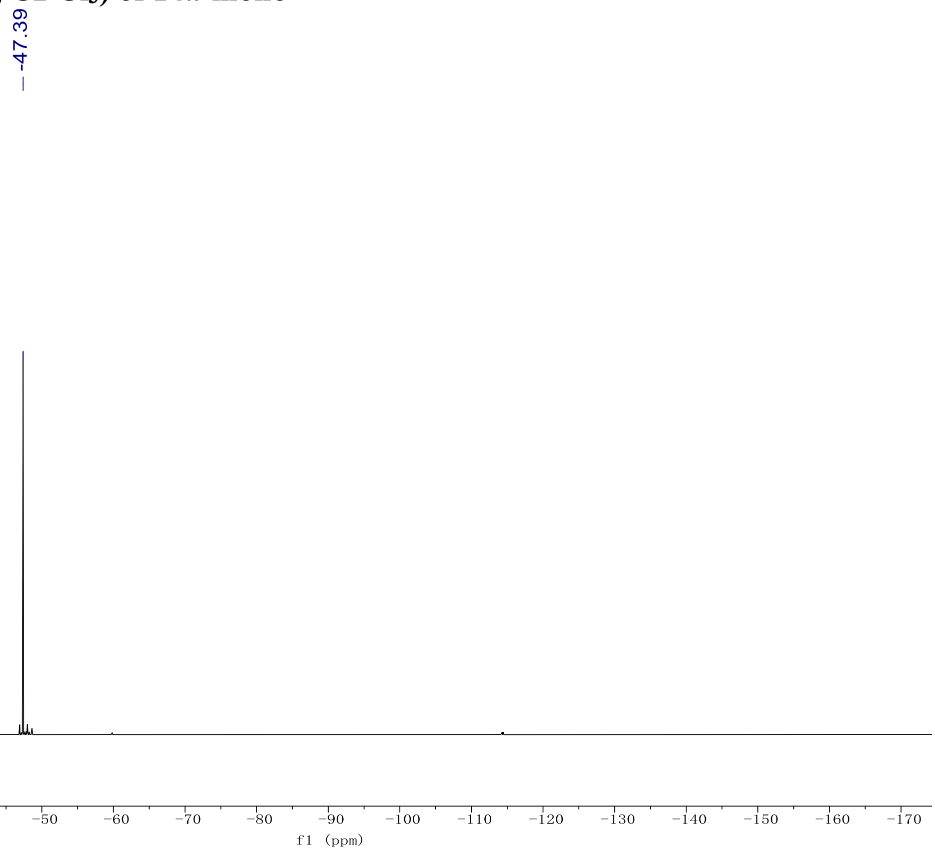

^{1}H-NMR (400 MHz, $CDCl_3$) of 14a-bis

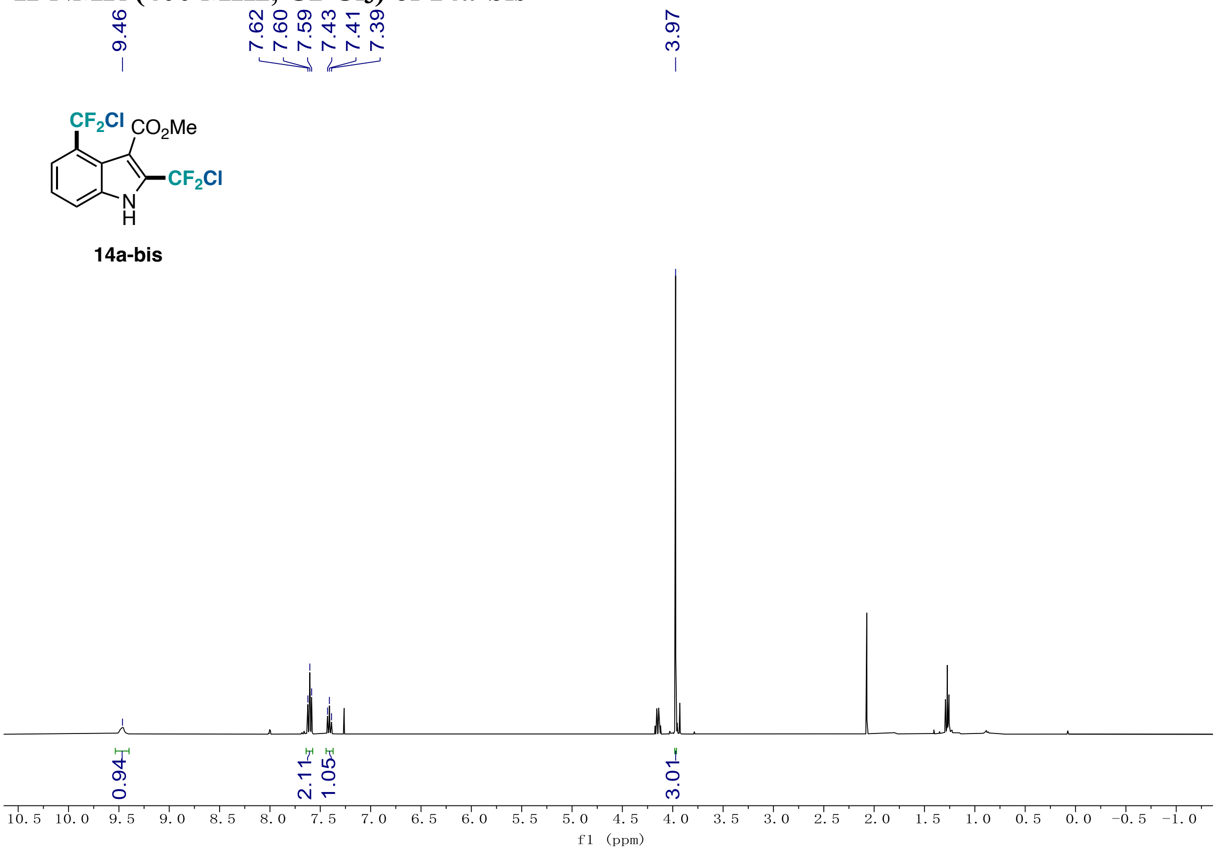

^{13}C-NMR (101 MHz, $CDCl_3$) of 14a-bis

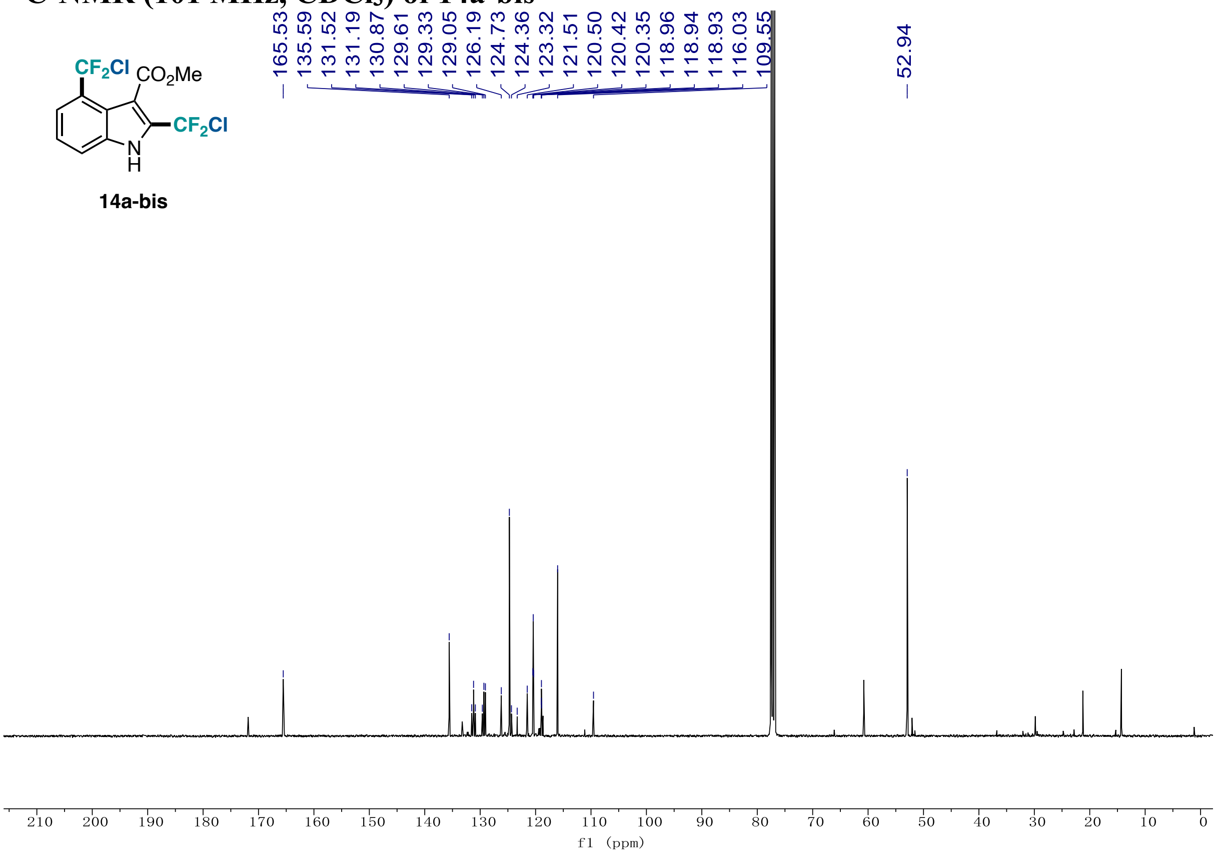

^{19}F-NMR (376 MHz, $CDCl_3$) of 14a-bis

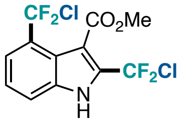

14a-bis

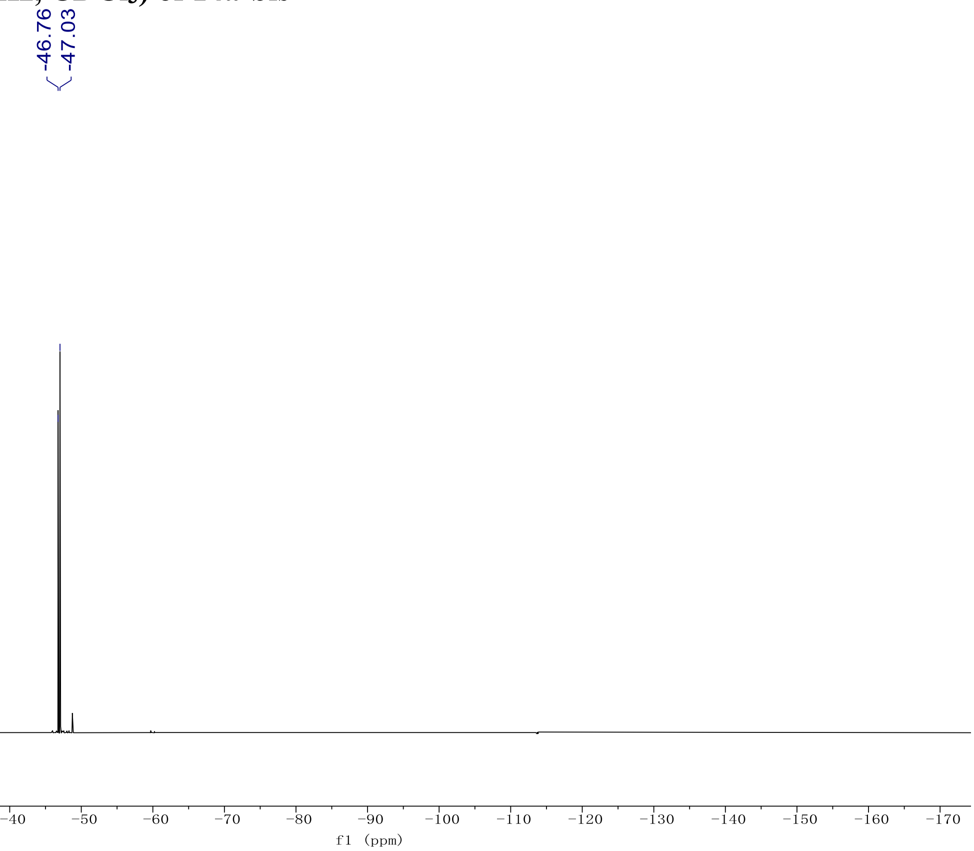

^{1}H-NMR (300 MHz, $CDCl_3$) of 15a-mono

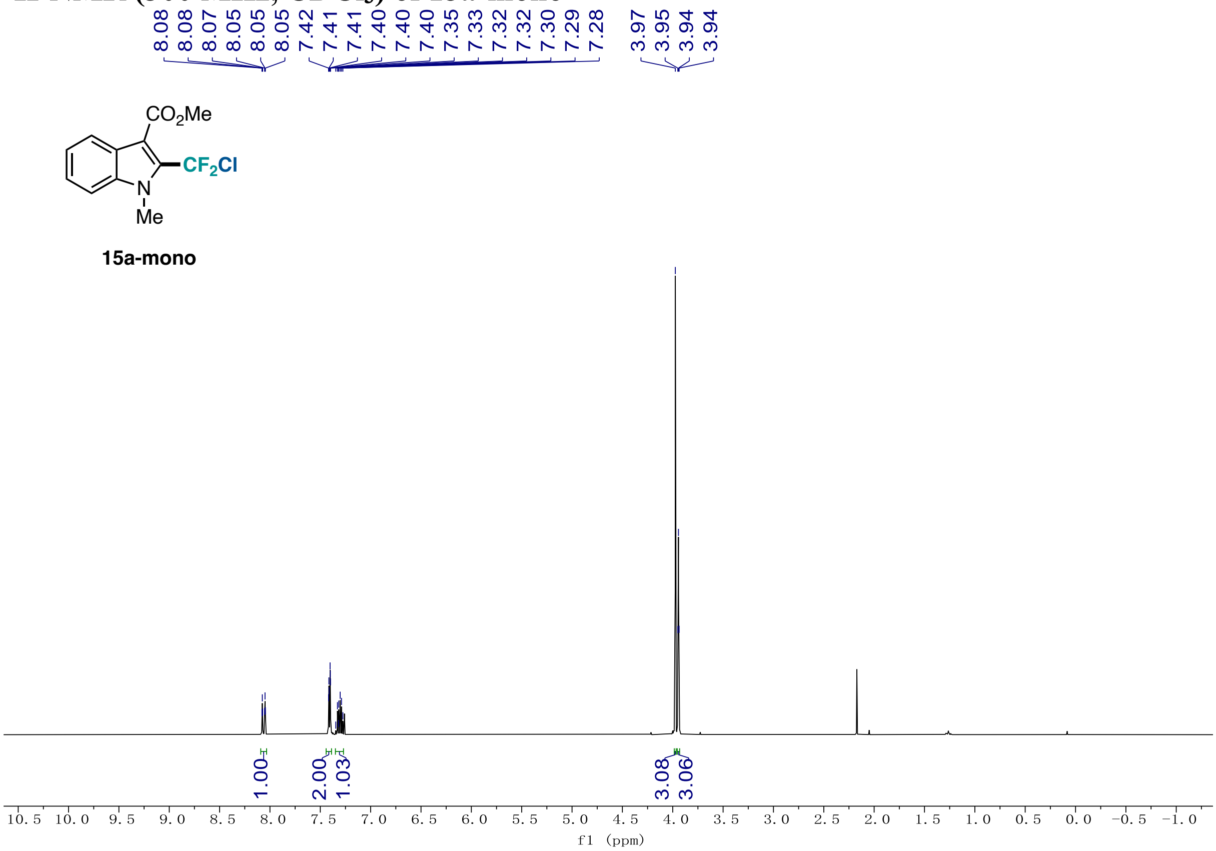

^{13}C-NMR (75 MHz, $CDCl_3$) of 15a-mono

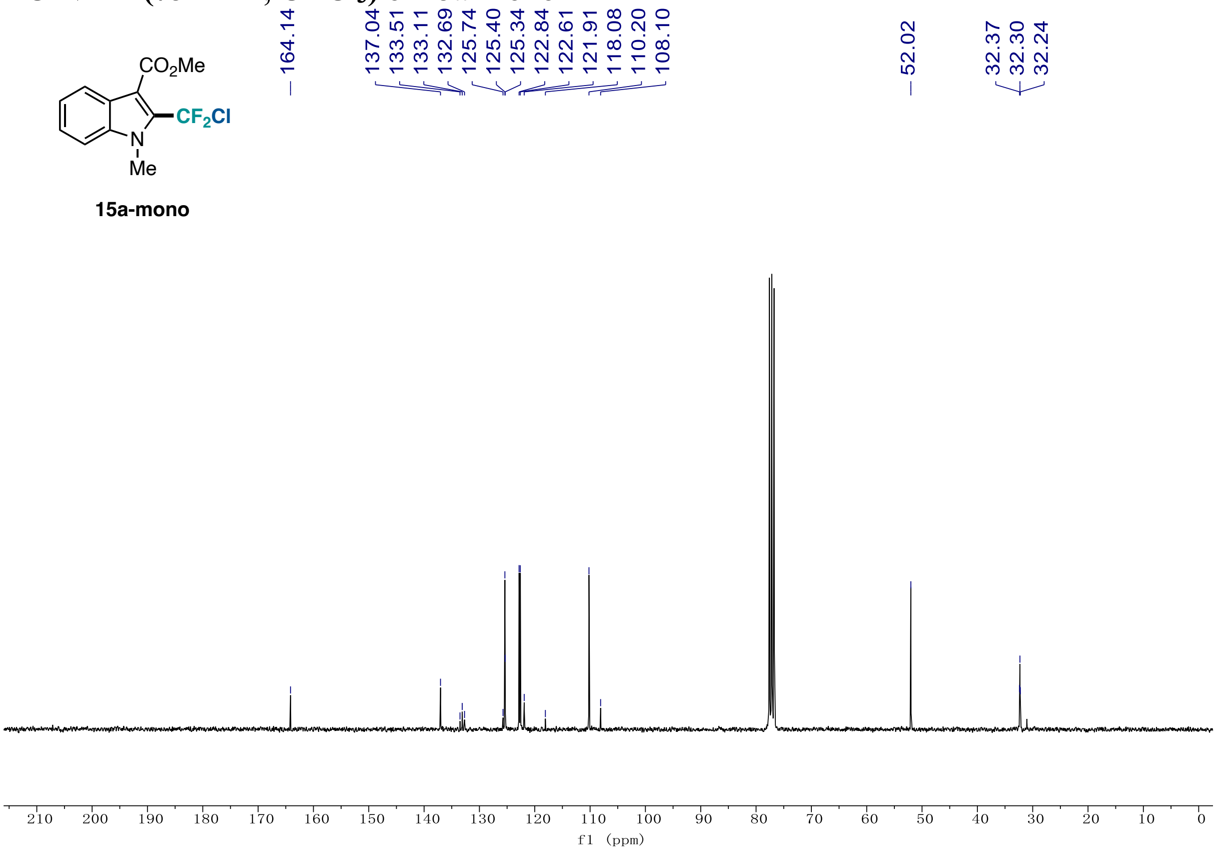

^{19}F-NMR (282 MHz, $CDCl_3$) of 15a-mono

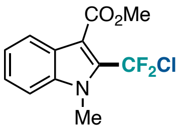

15a-mono

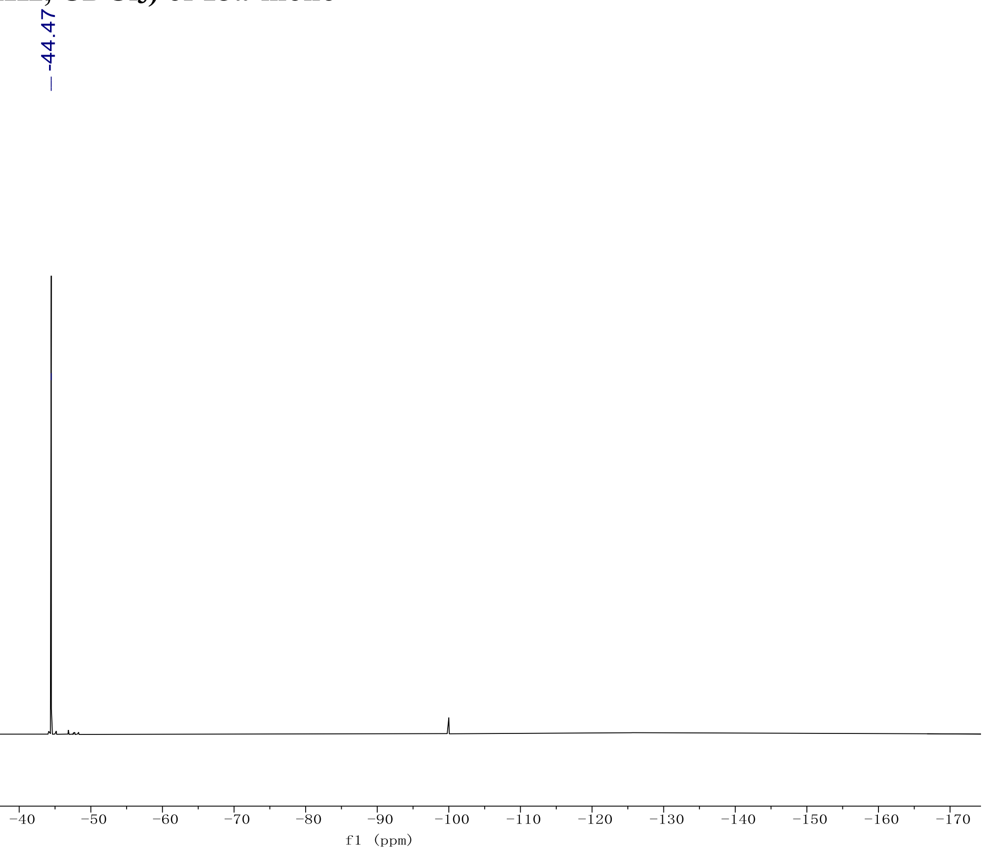

^{1}H-NMR (400 MHz, $CDCl_3$) of 15a-bis

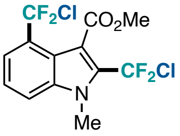

15a-bis

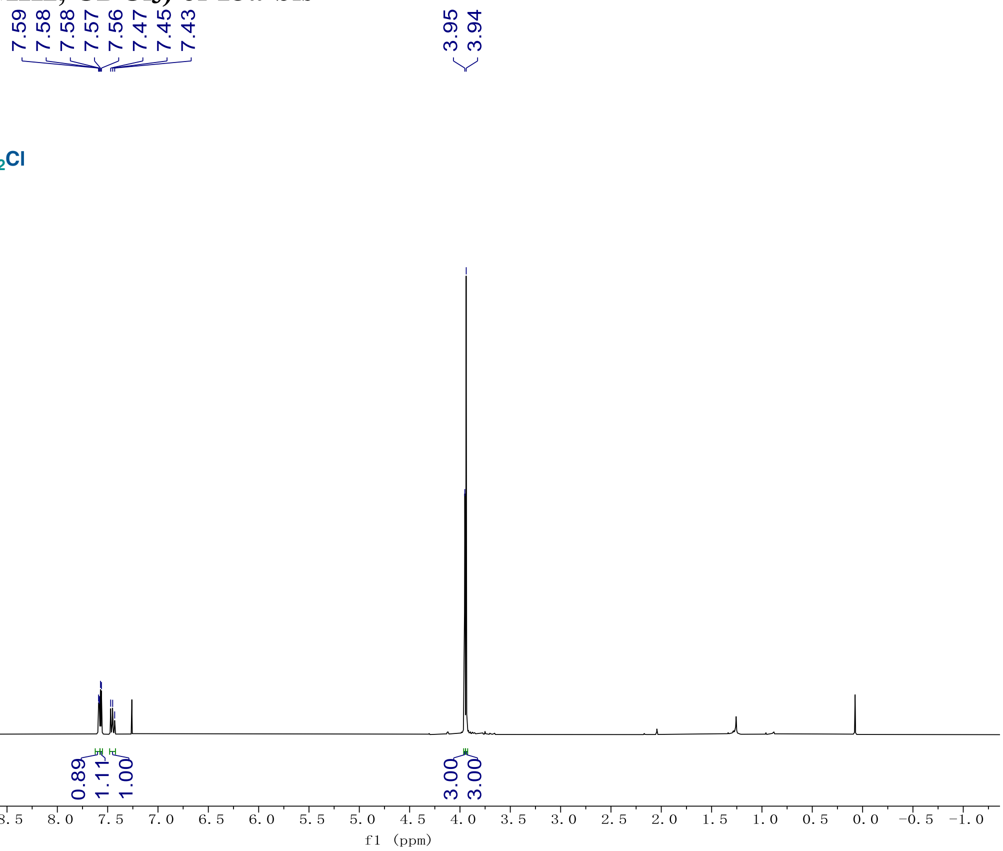

^{13}C-NMR (101 MHz, $CDCl_3$) of 15a-bis

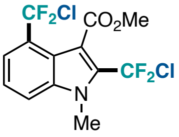

15a-bis

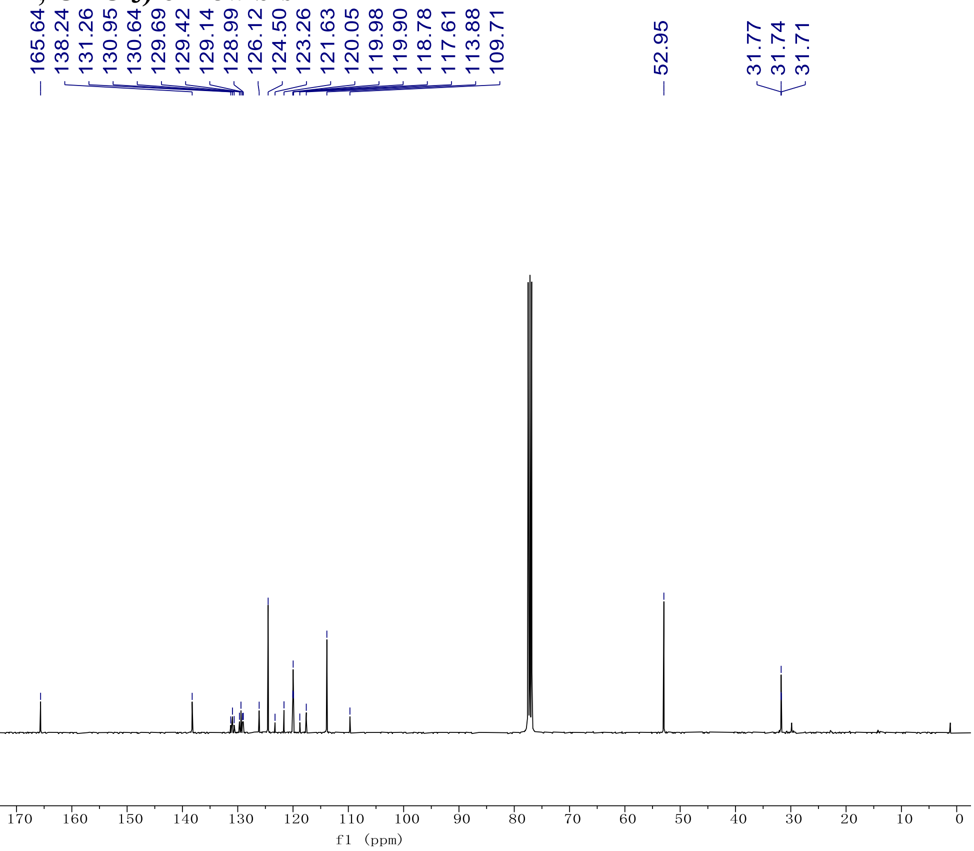

^{19}F-NMR (376 MHz, $CDCl_3$) of 15a-bis

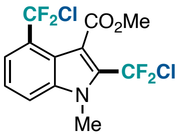

15a-bis

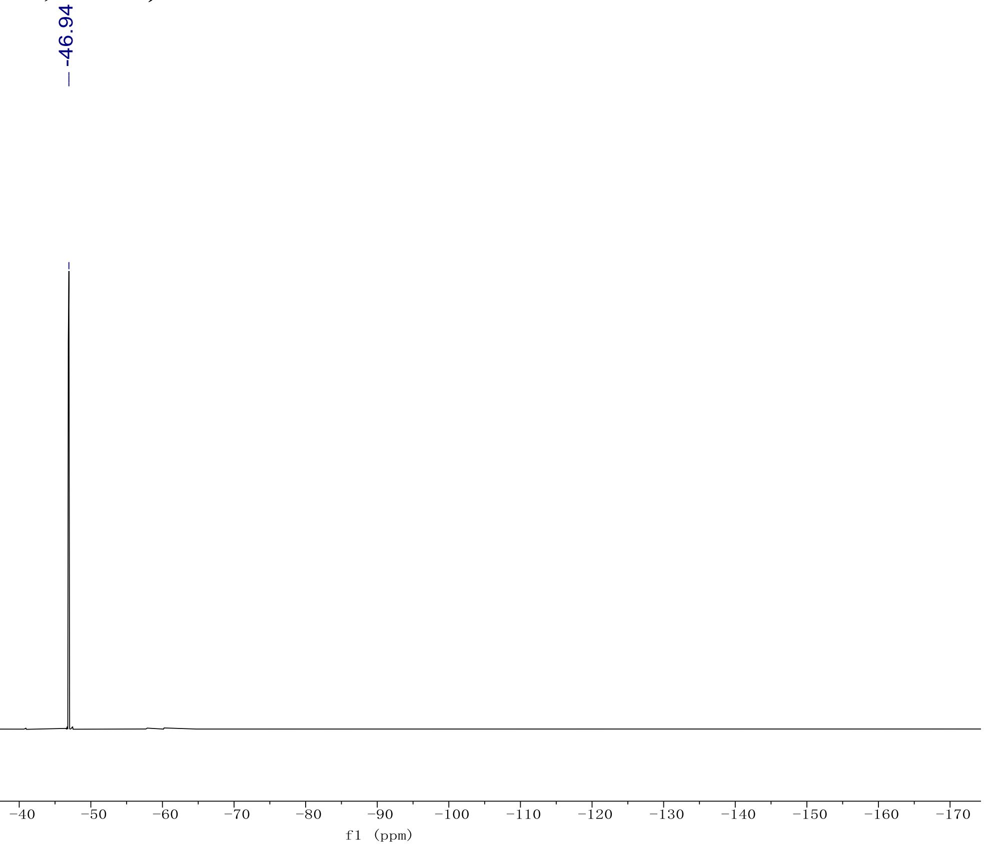

^{1}H-NMR (400 MHz, $CDCl_3$) of 16a

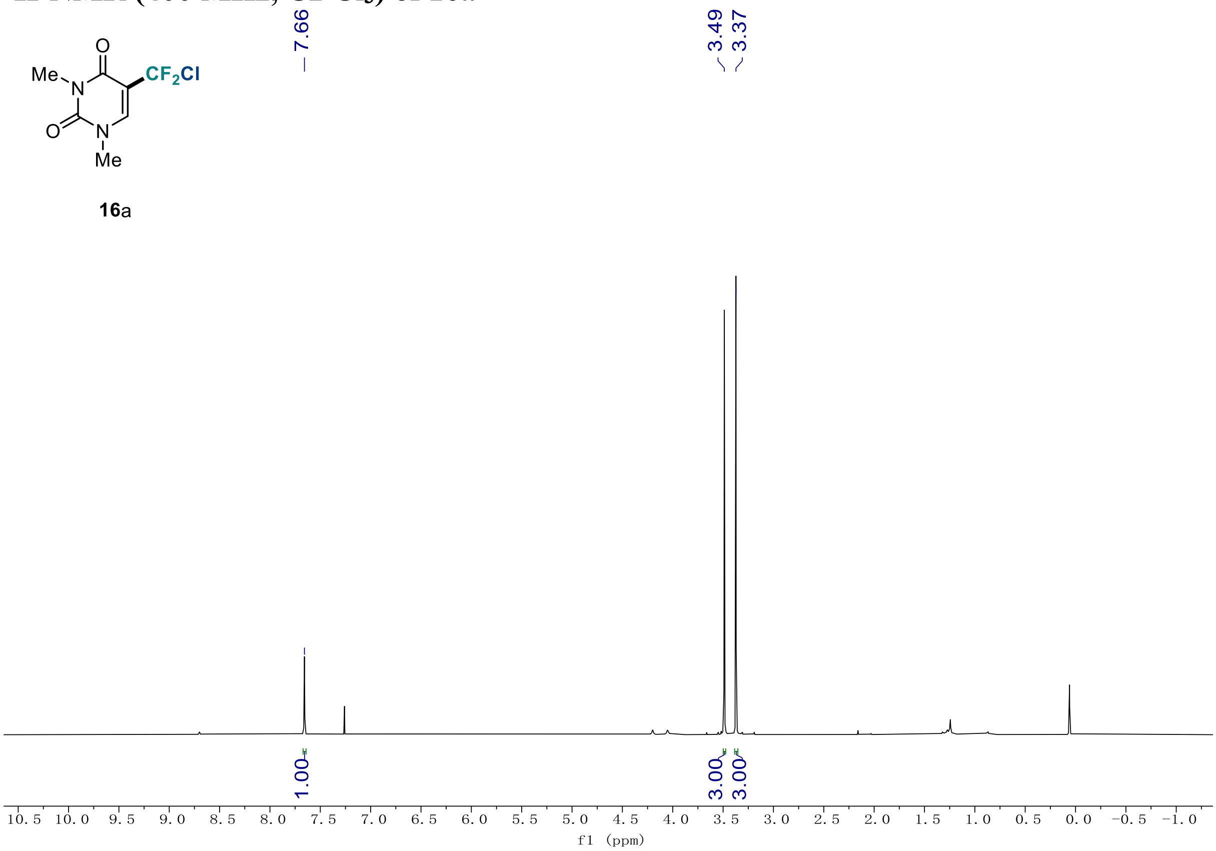

^{13}C-NMR (151 MHz, $CDCl_3$) of 16a

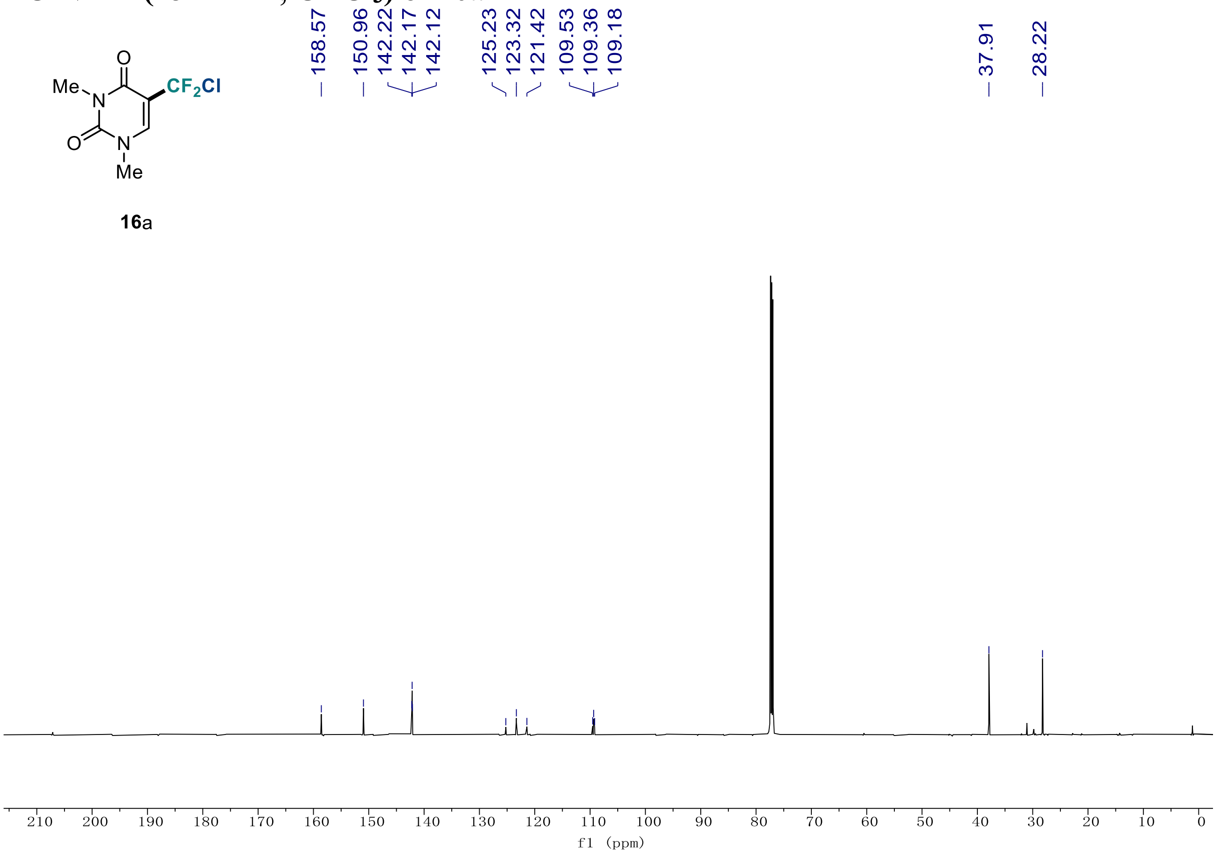

^{19}F-NMR (376 MHz, $CDCl_3$) of 16a

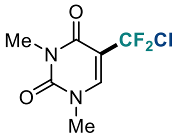

16a

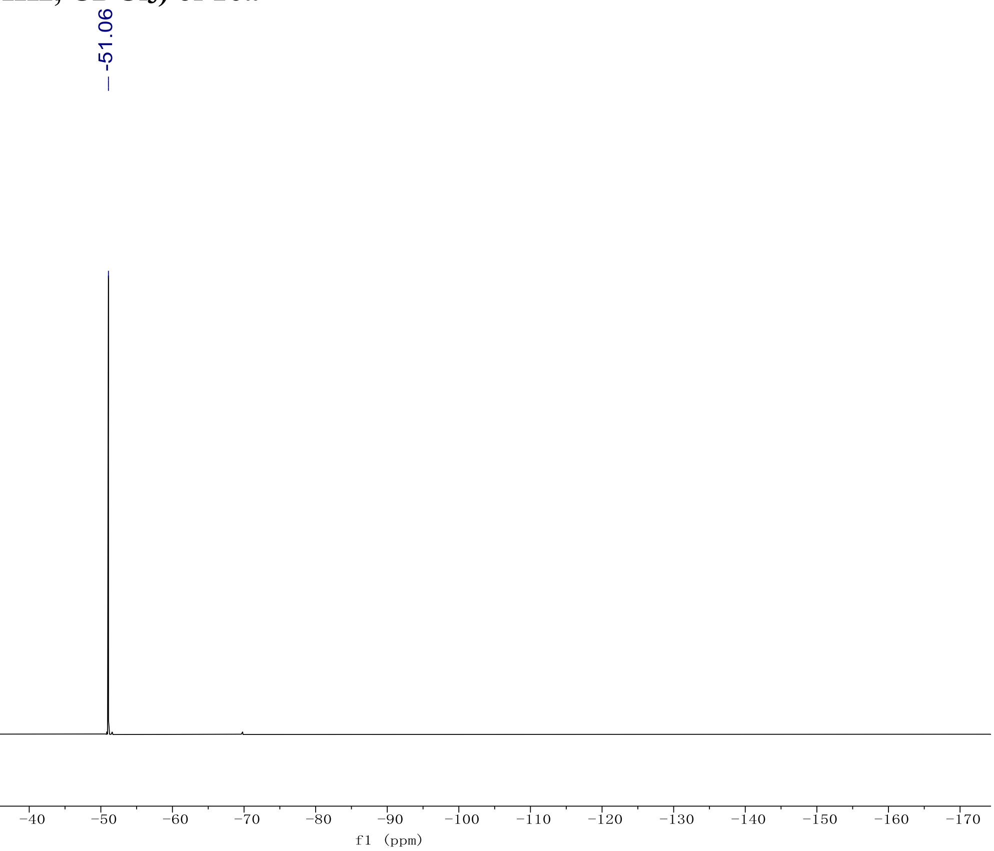

^{1}H-NMR (400 MHz, $CDCl_3$) of 17a

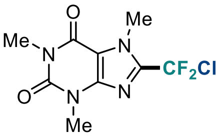

17a

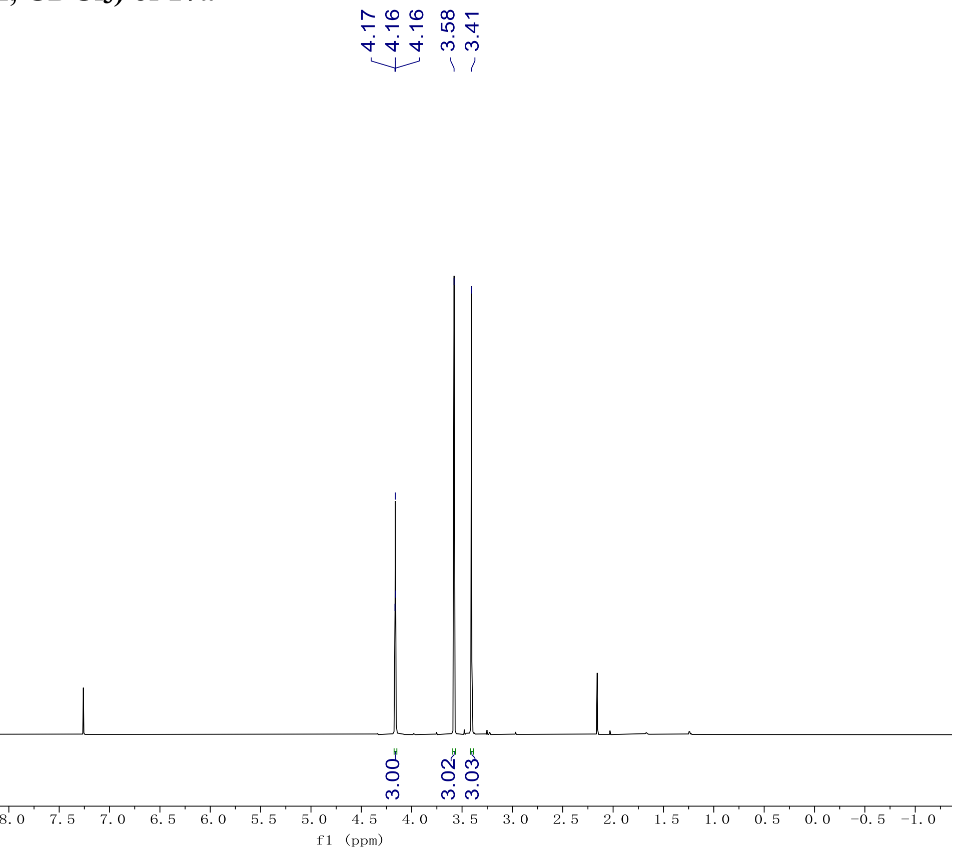

^{13}C-NMR (101 MHz, $CDCl_3$) of 17a

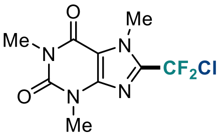

17a

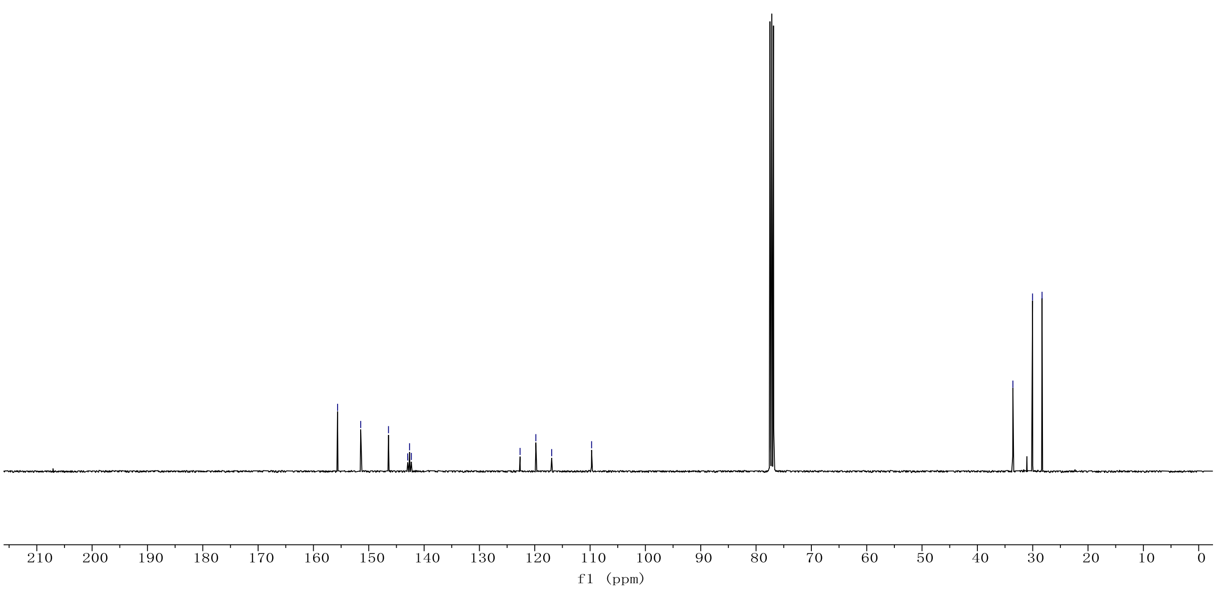

^{19}F-NMR (376 MHz, $CDCl_3$) of 17a

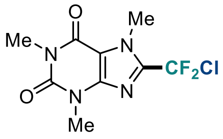

17a

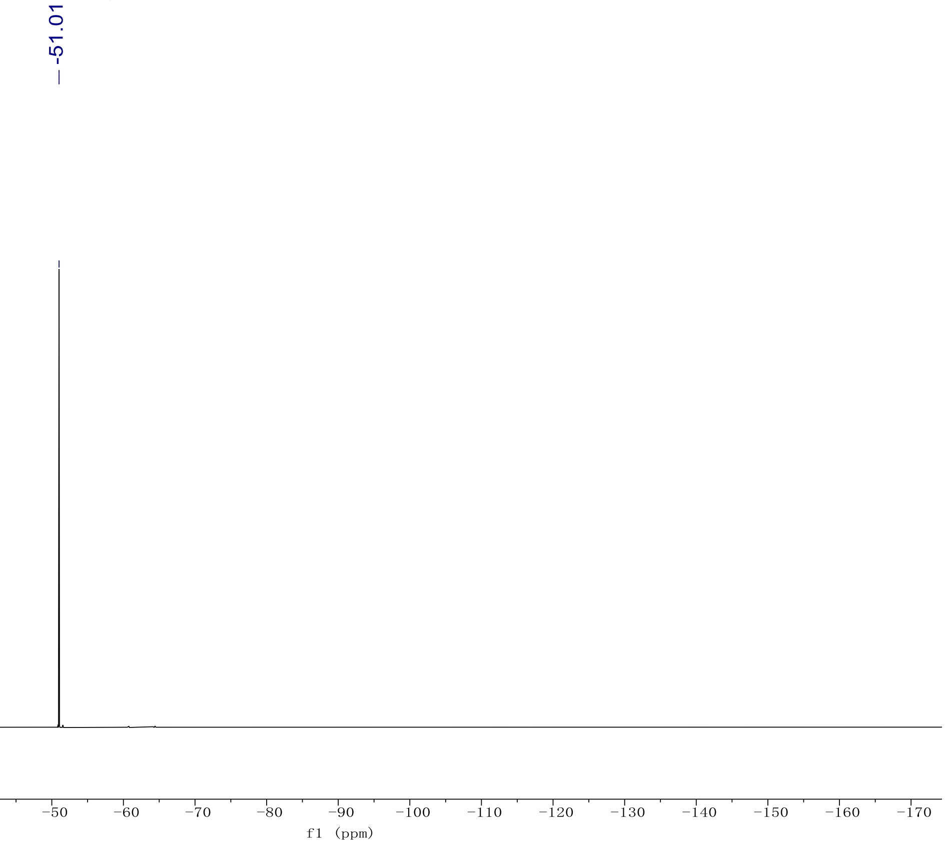

^{1}H-NMR (400 MHz, $CDCl_3$) of 18a

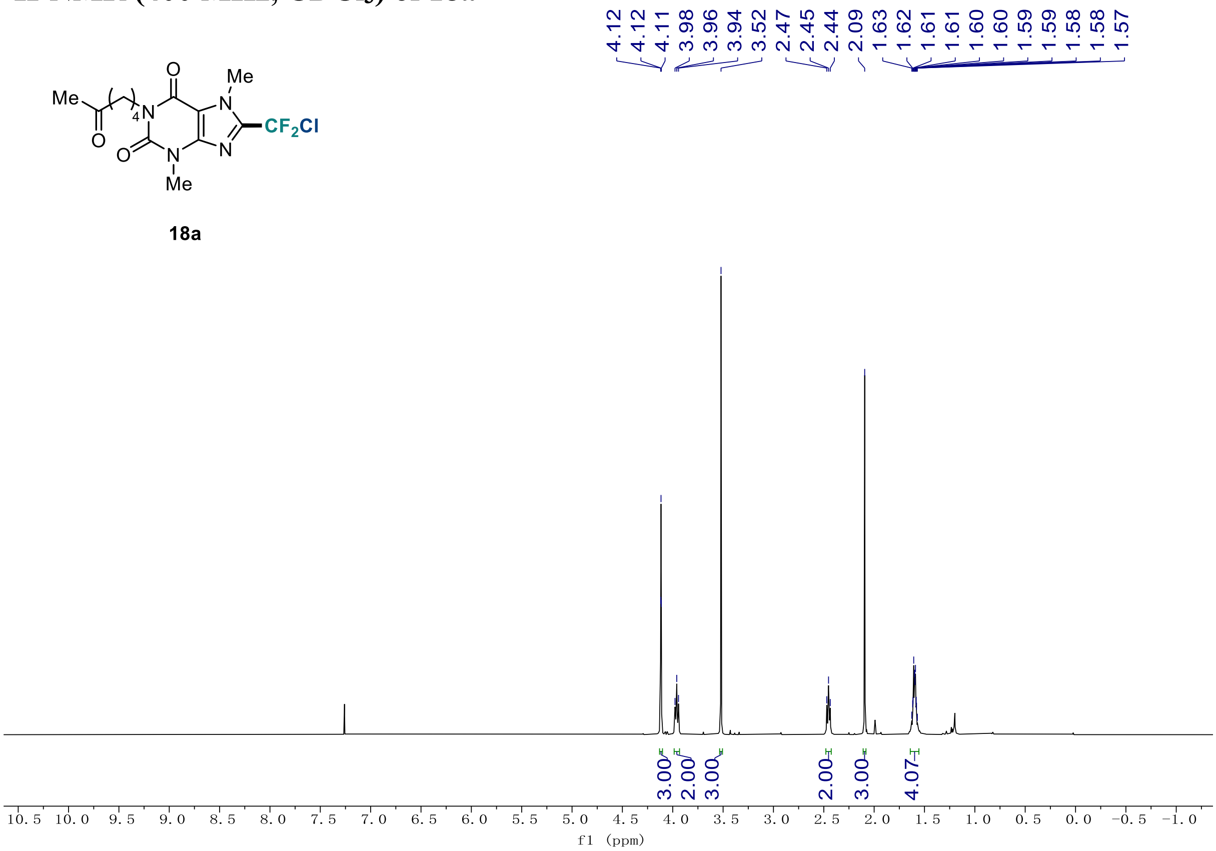

^{13}C-NMR (101 MHz, $CDCl_3$) of 18a

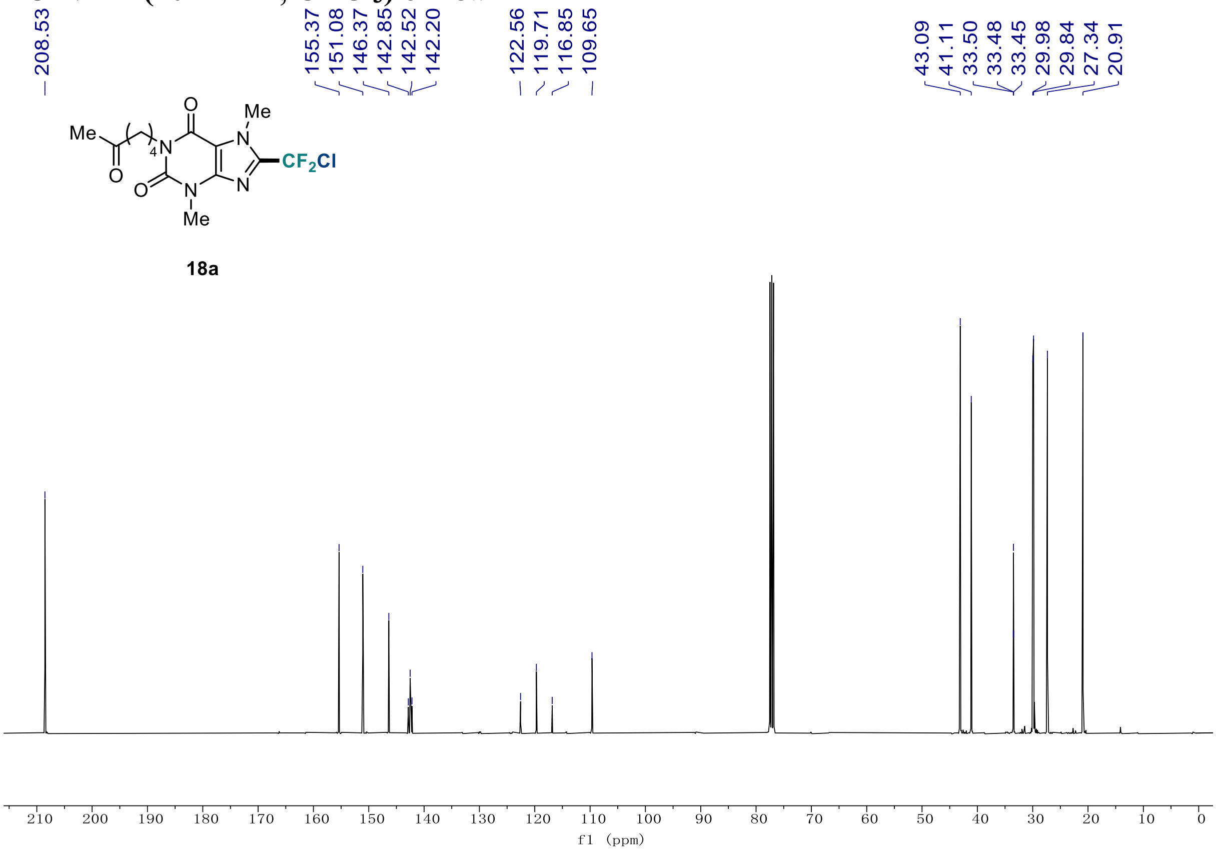

^{19}F-NMR (376 MHz, CDCl$_3$) of 18a

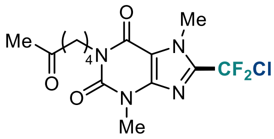

18a

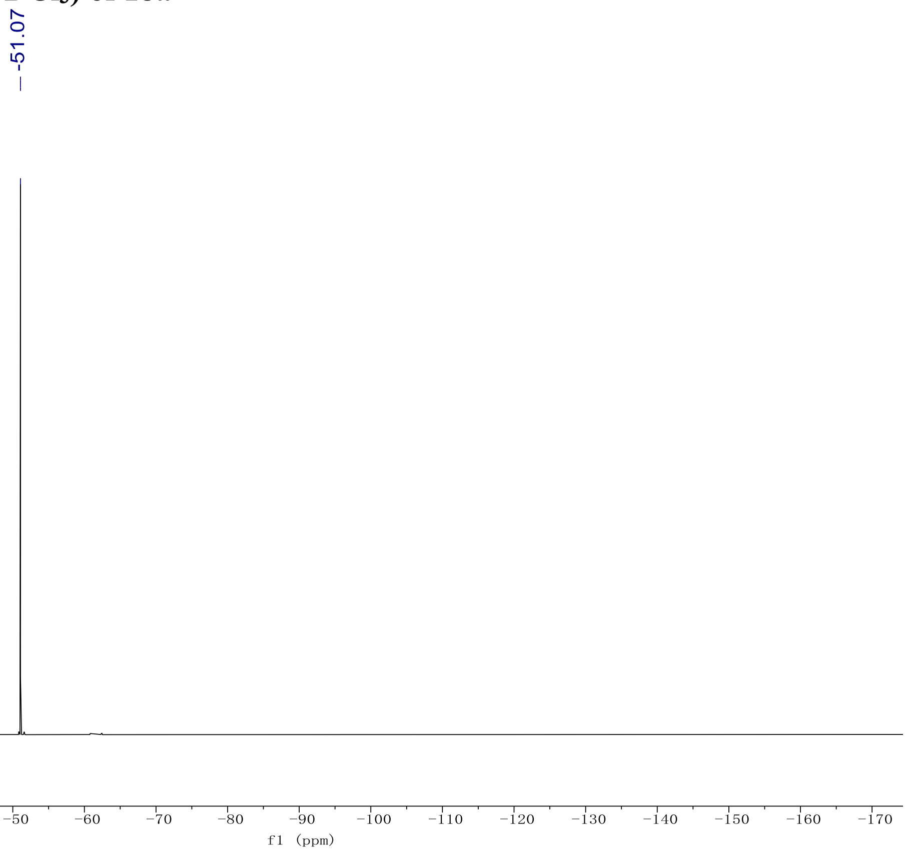

^{1}H-NMR (400 MHz, $CDCl_3$) of 19a

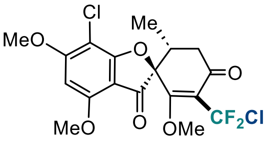

19a

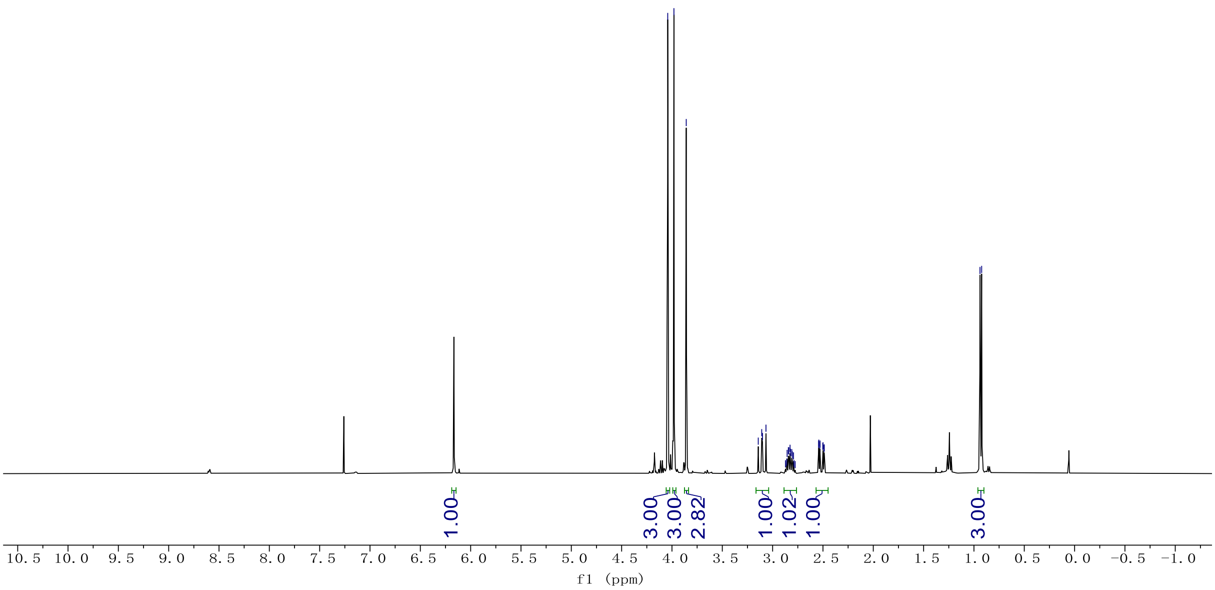

^{13}C-NMR (101 MHz, $CDCl_3$) of 19a

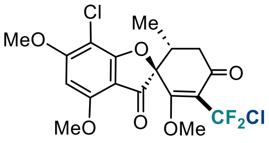

19a

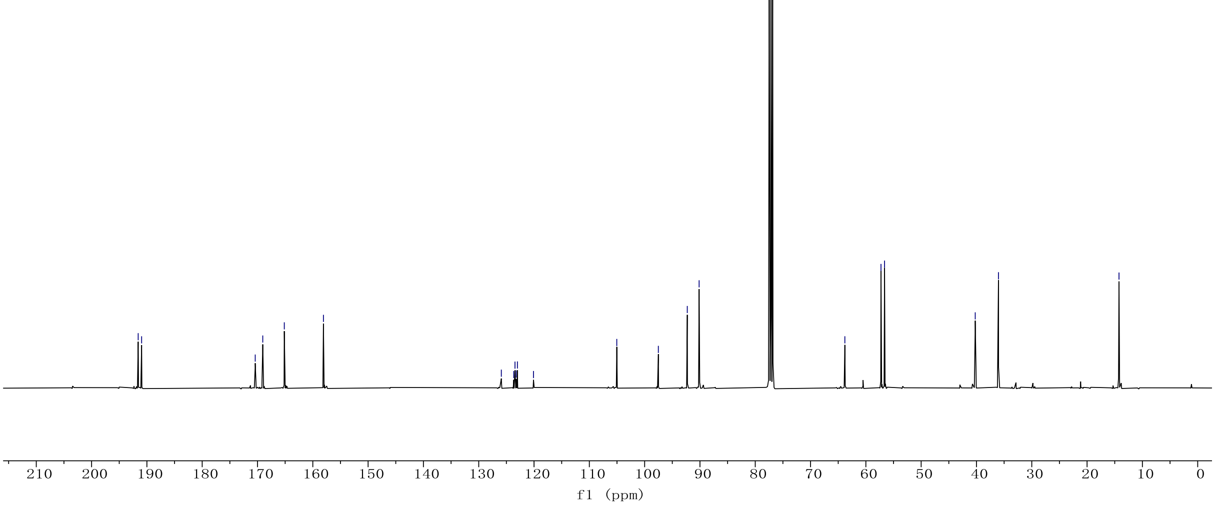

^{19}F-NMR (376 MHz, $CDCl_3$) of 19a

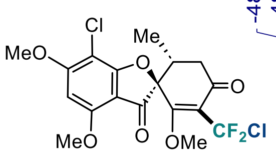

19a

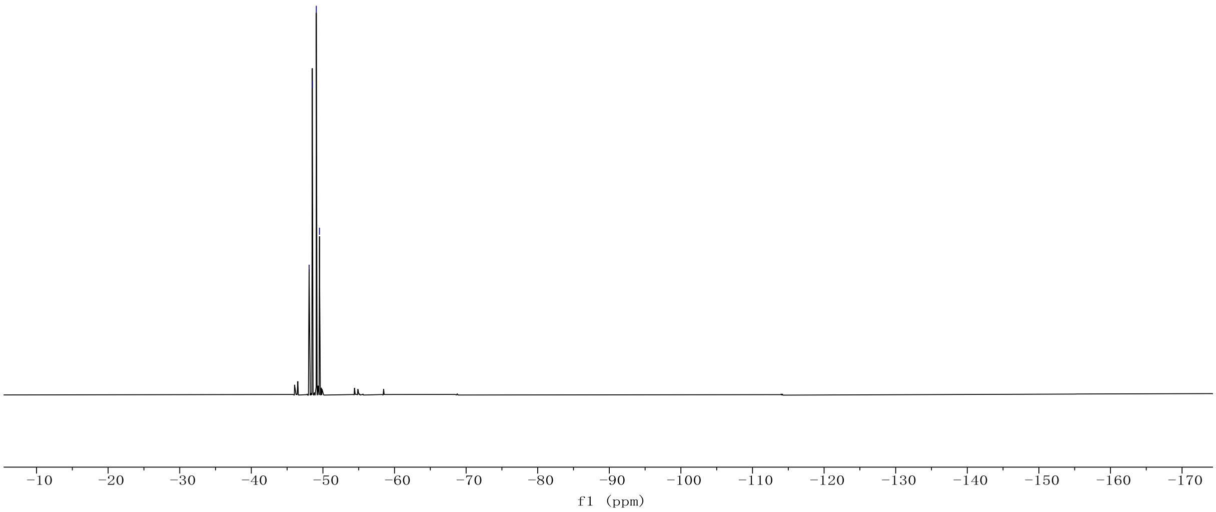

^{1}H-NMR (600 MHz, $CDCl_3$) of 20a

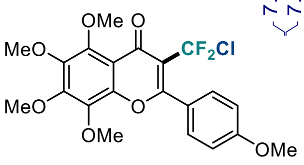

20a

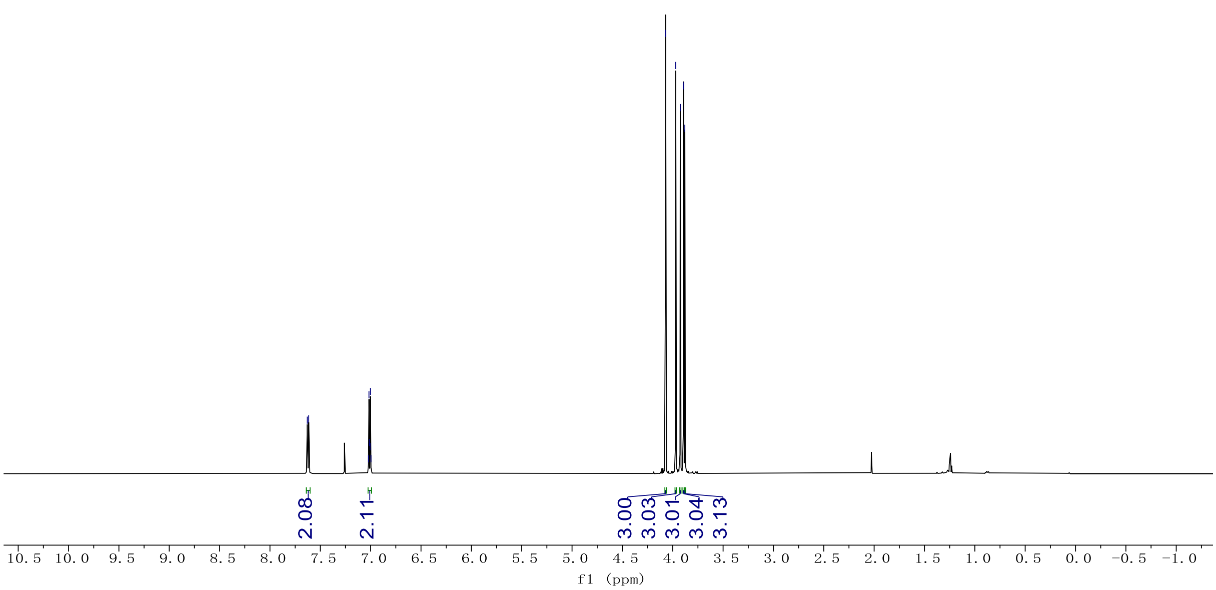

^{13}C-NMR (151 MHz, $CDCl_3$) of 20a

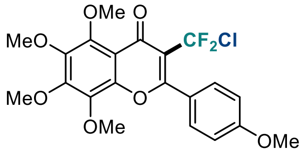

20a

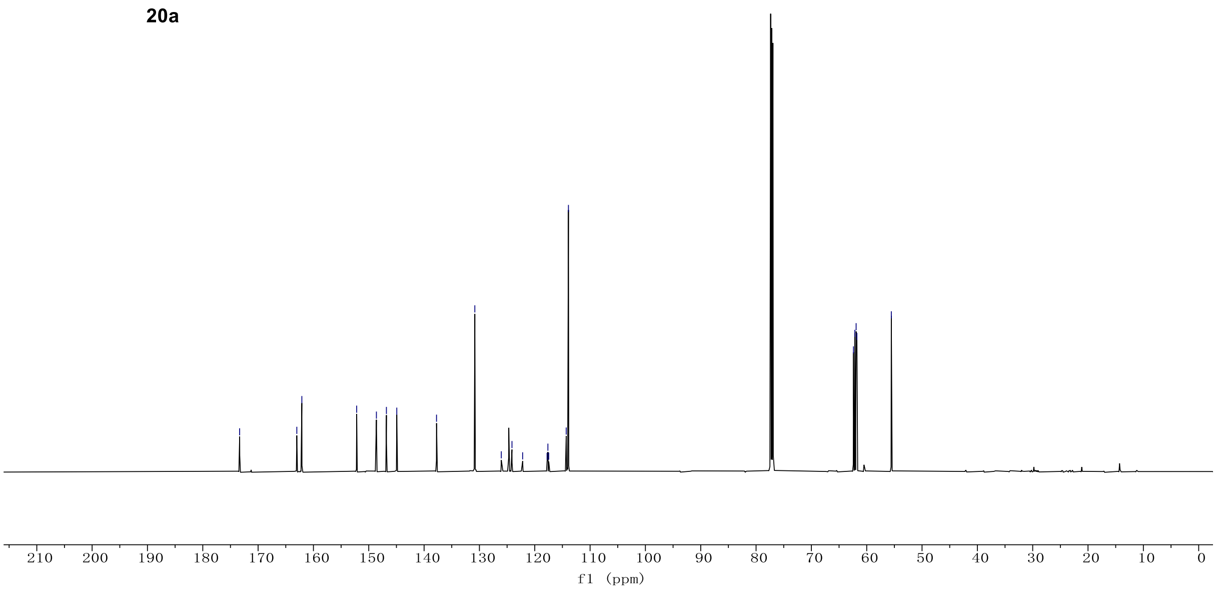

^{19}F-NMR (376 MHz, $CDCl_3$) of 20a

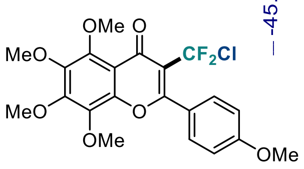

20a

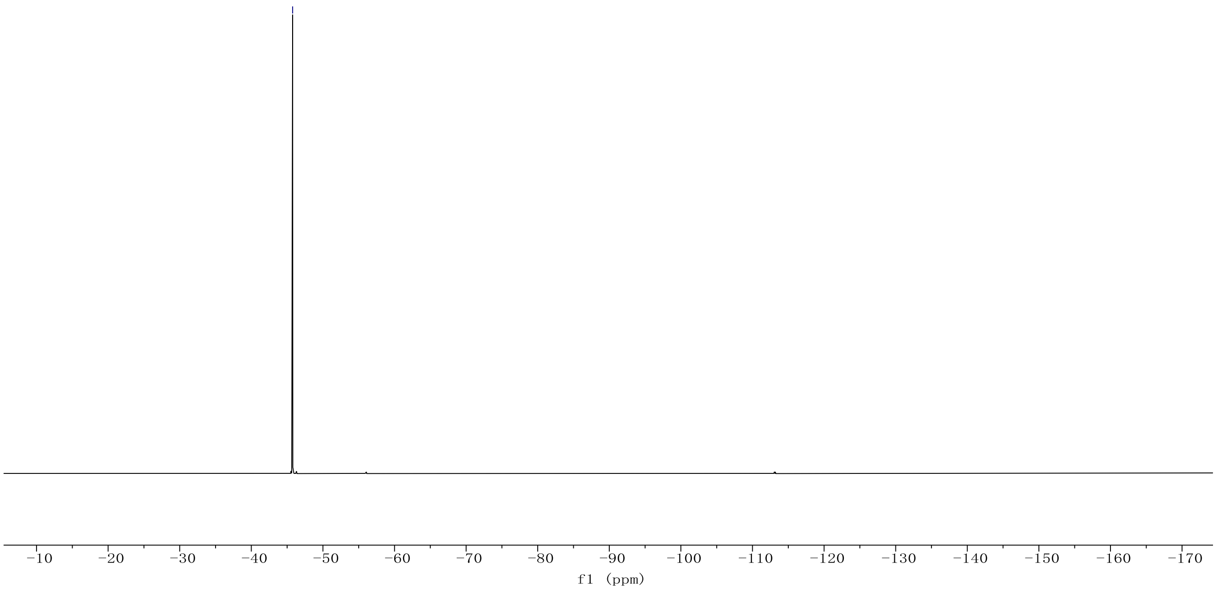

^{1}H-NMR (400 MHz, $CDCl_3$) of 1b

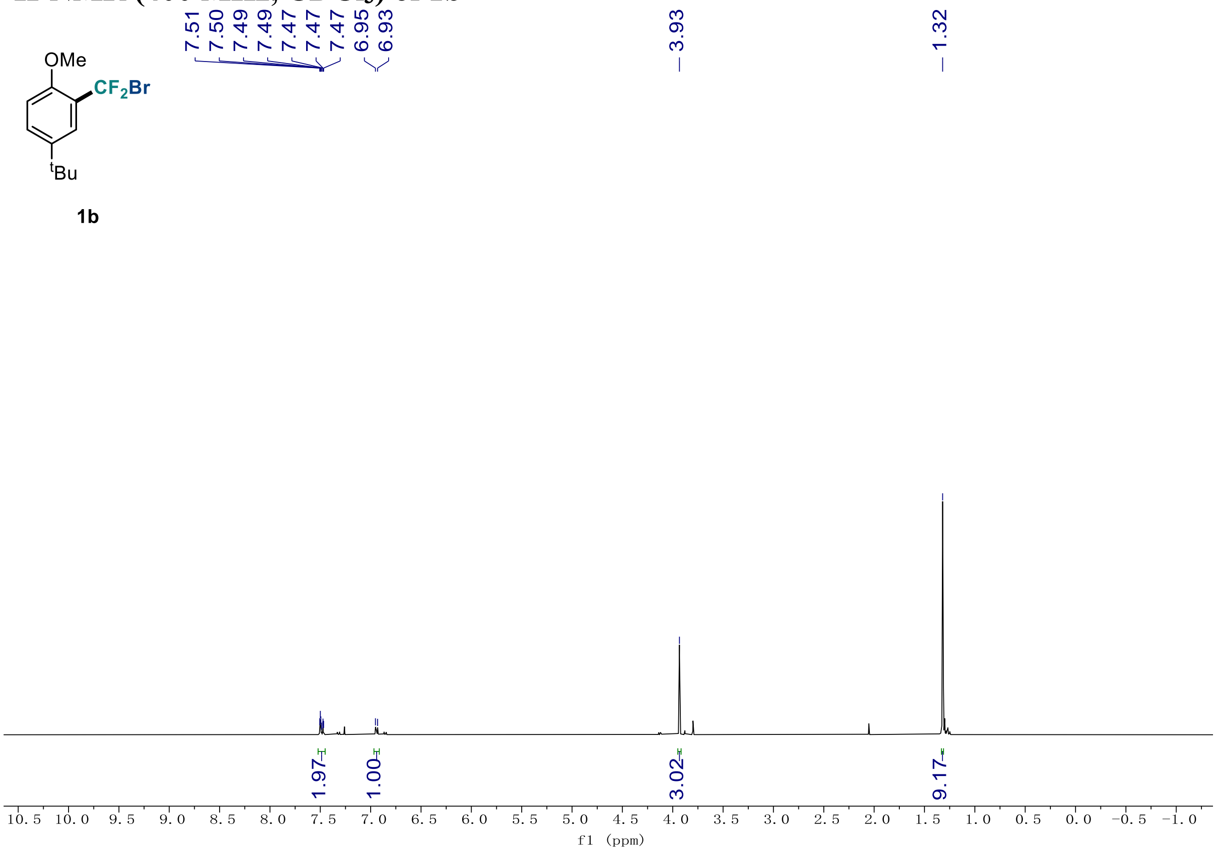

^{19}F-NMR (376 MHz, $CDCl_3$) of 1b

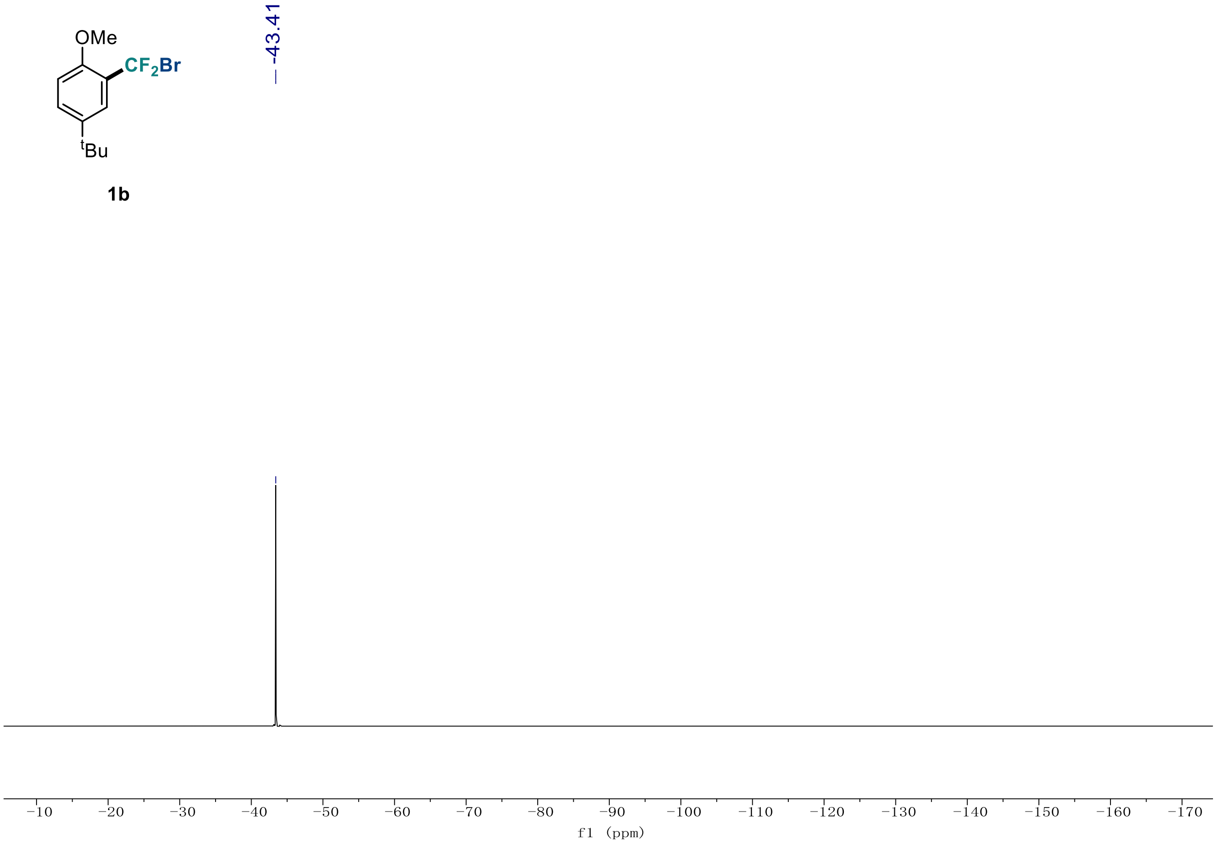

^{1}H-NMR (400 MHz, $CDCl_3$) of 17b

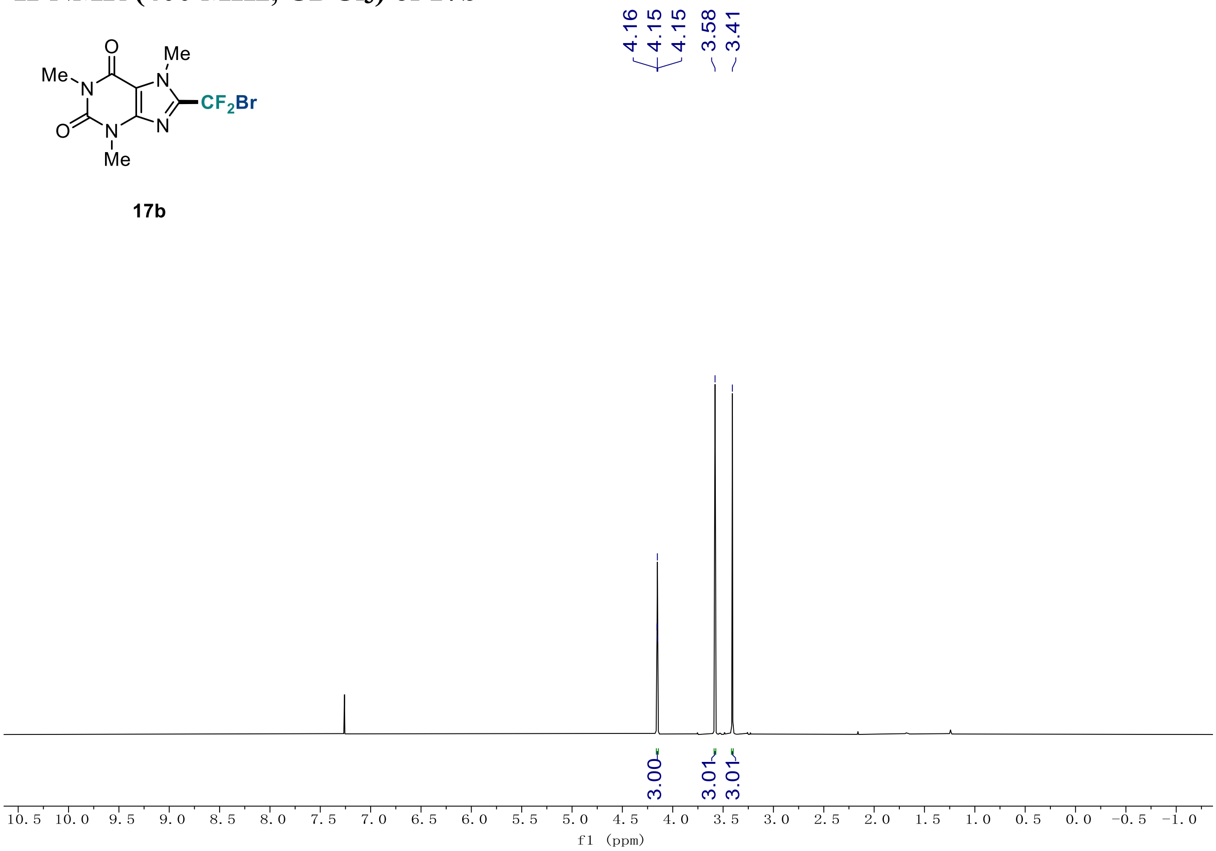

^{13}C-NMR (101 MHz, $CDCl_3$) of 17b

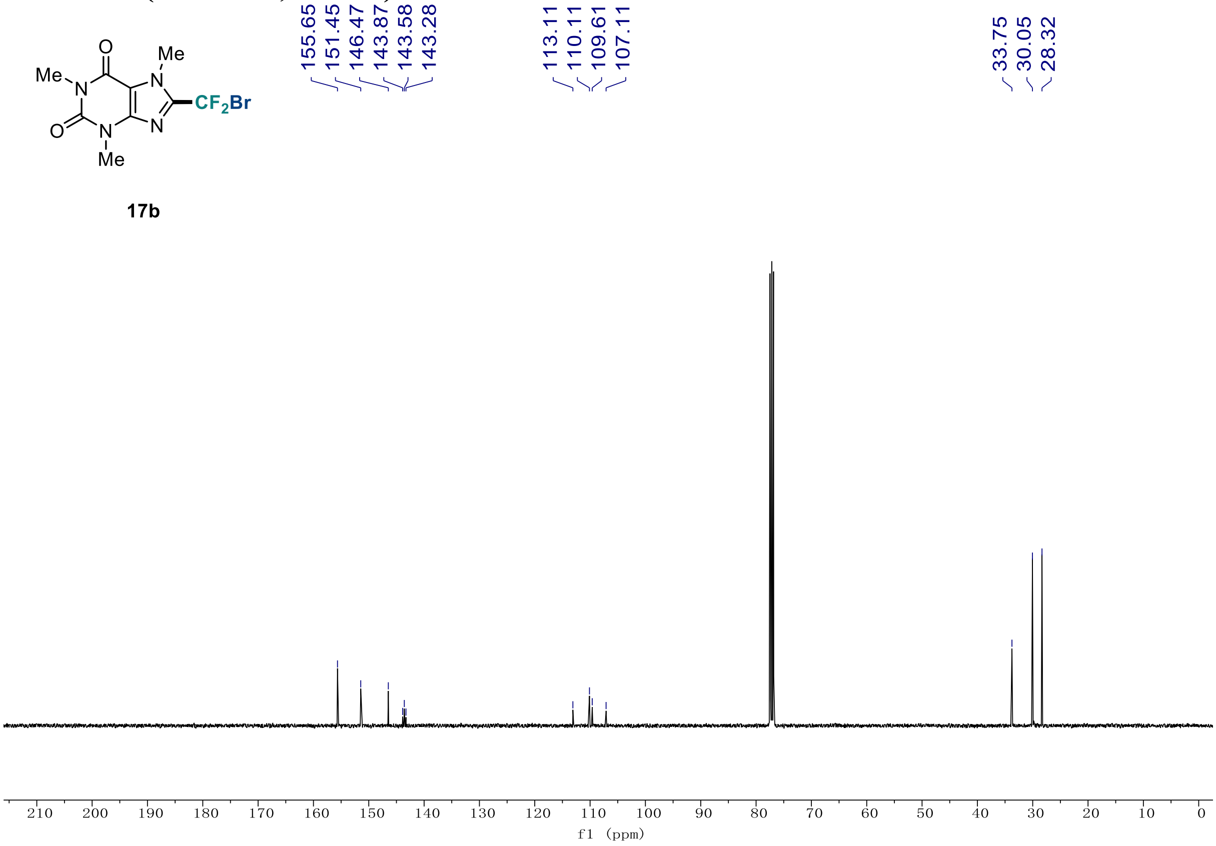

^{19}F-NMR (376 MHz, $CDCl_3$) of 17b

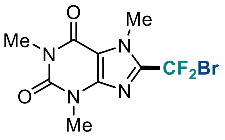

17b

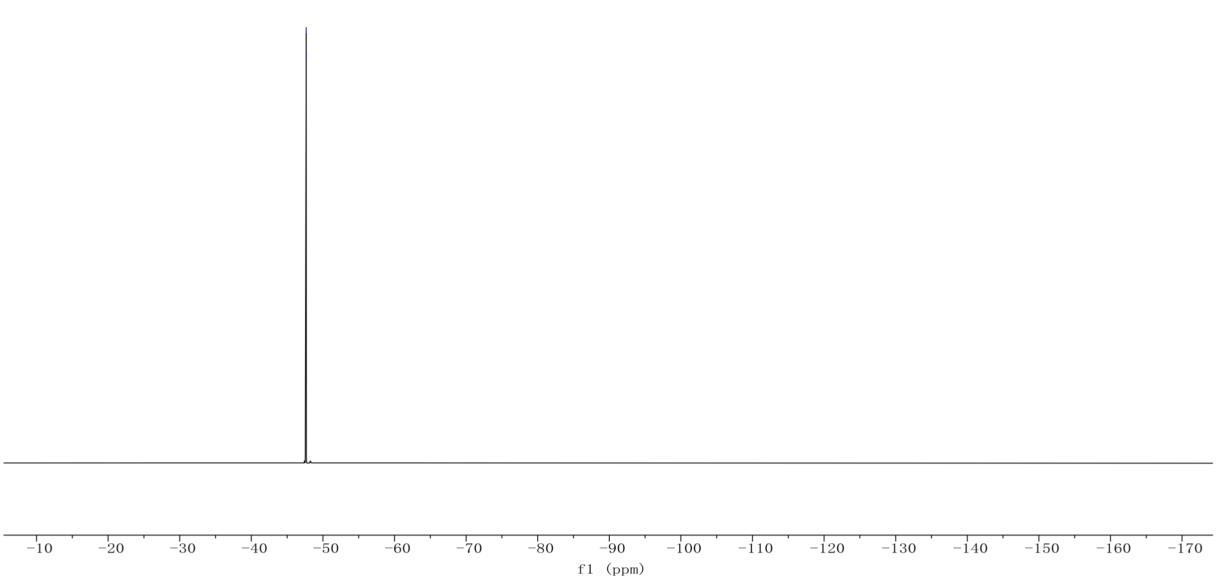

^{1}H-NMR (400 MHz, $CDCl_3$) of 18b

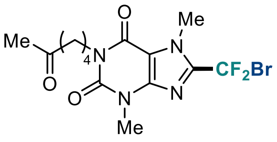

18b

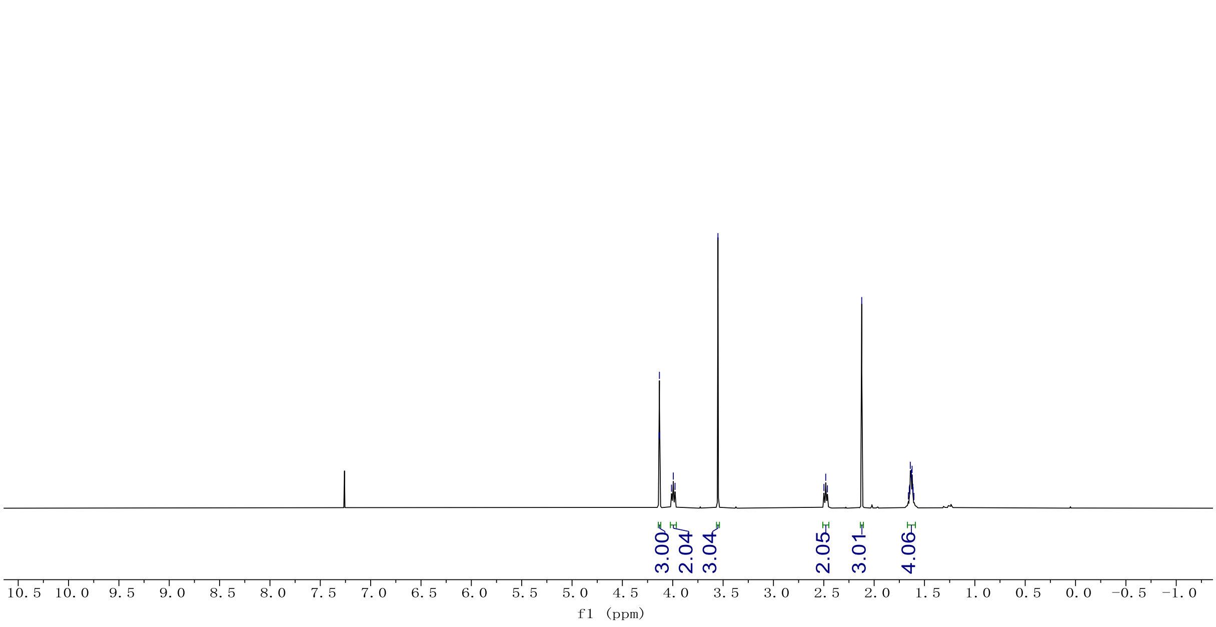

^{13}C-NMR (151 MHz, $CDCl_3$) of 18b

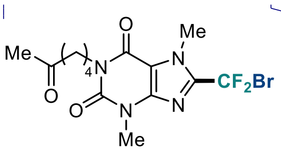

18b

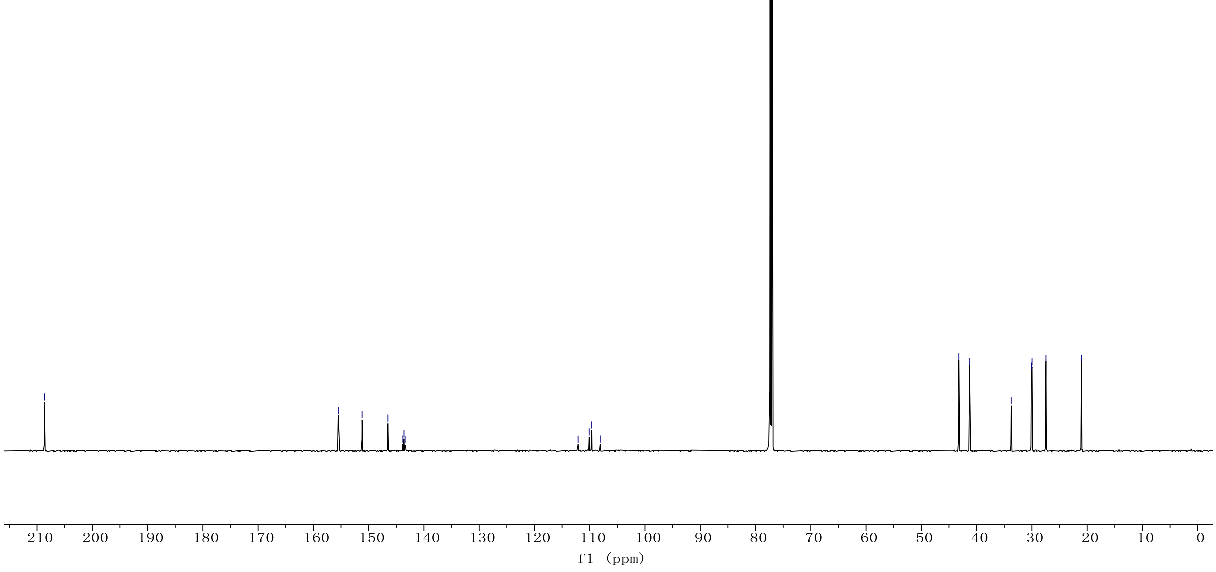

^{19}F-NMR (376 MHz, $CDCl_3$) of 18b

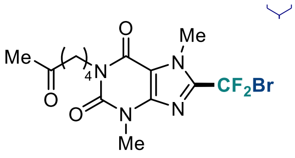

18b

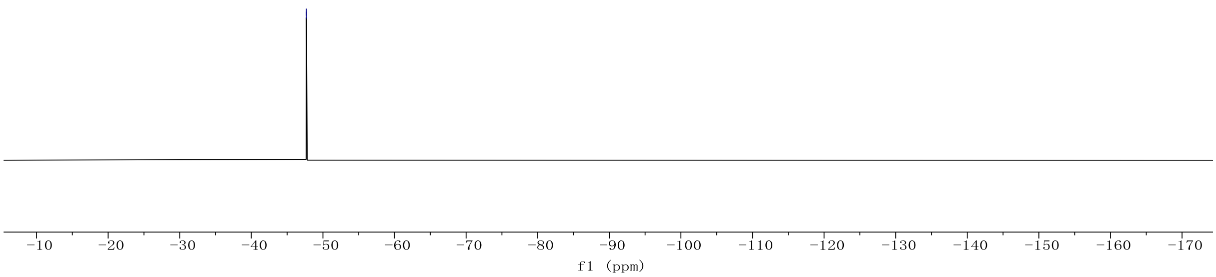

^{1}H-NMR (600 MHz, $CDCl_3$) of 21

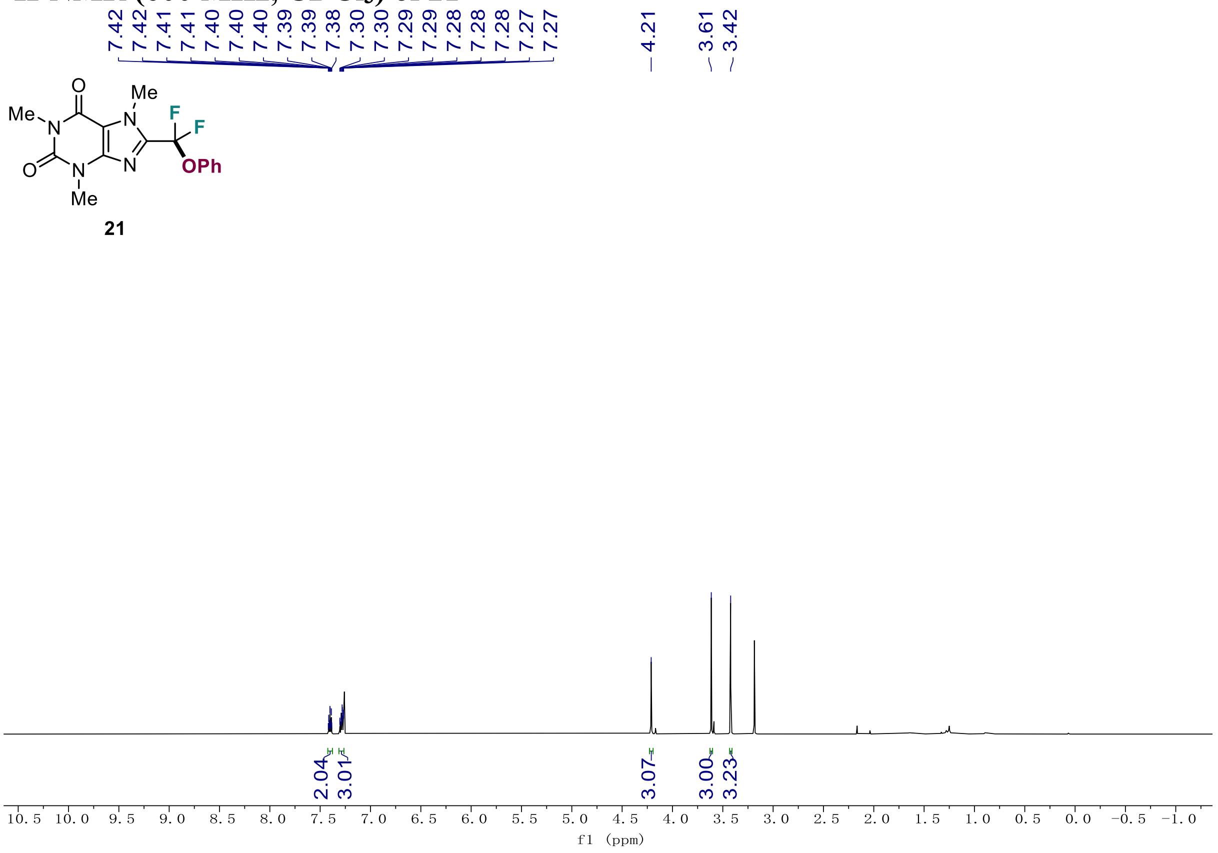

^{13}C-NMR (151 MHz, $CDCl_3$) of 21

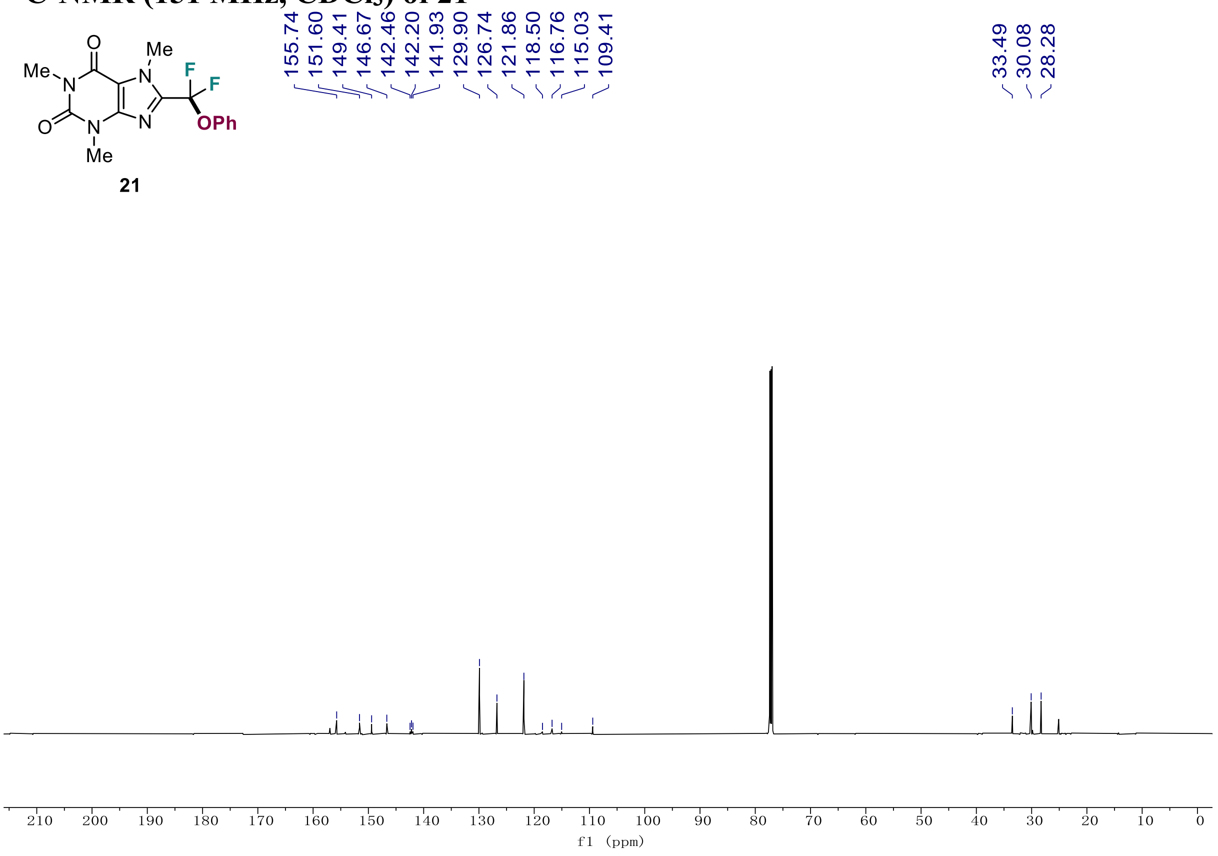

^{19}F-NMR (376 MHz, $CDCl_3$) of 21

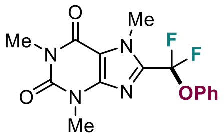

21

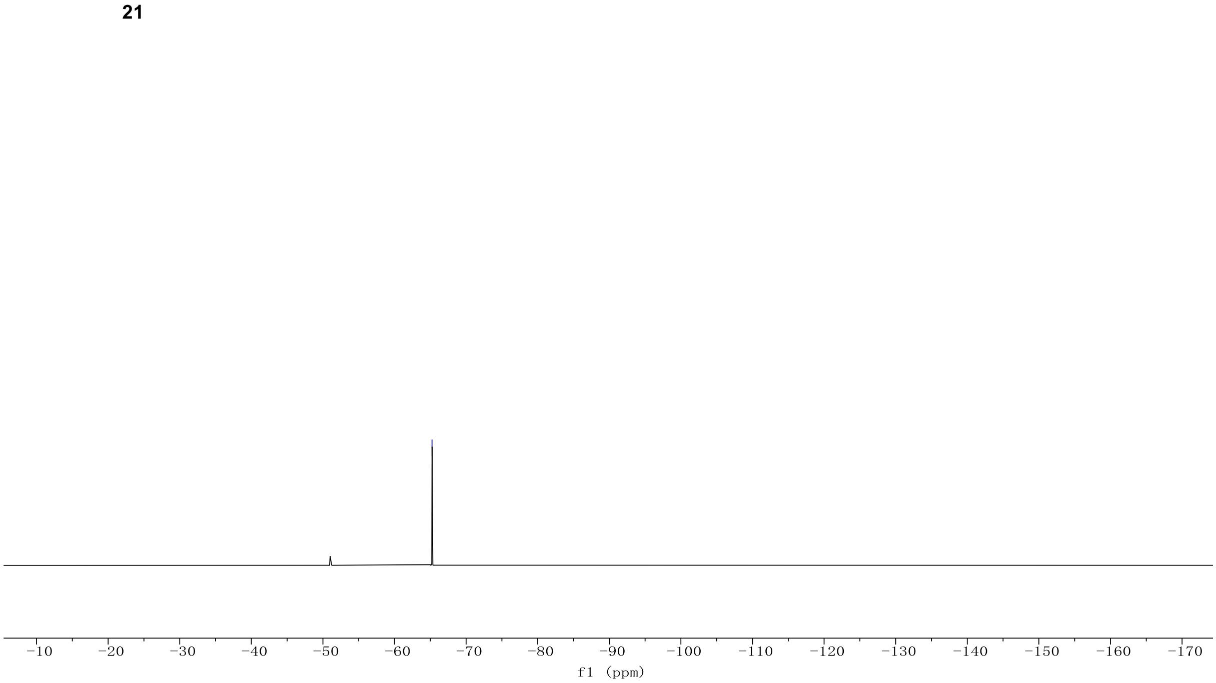

^{1}H-NMR (600 MHz, $CDCl_3$) of 22

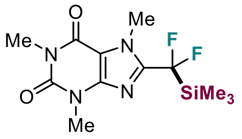

22

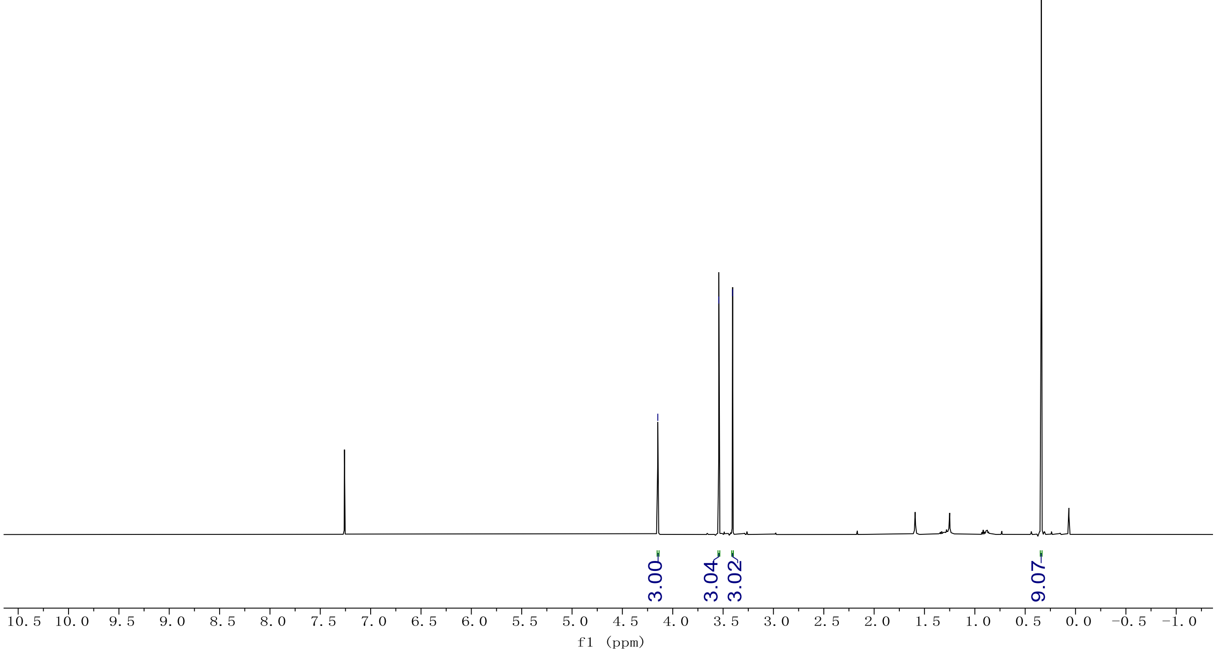

^{13}C-NMR (151 MHz, $CDCl_3$) of 22

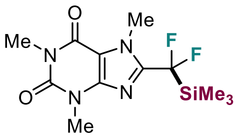

22

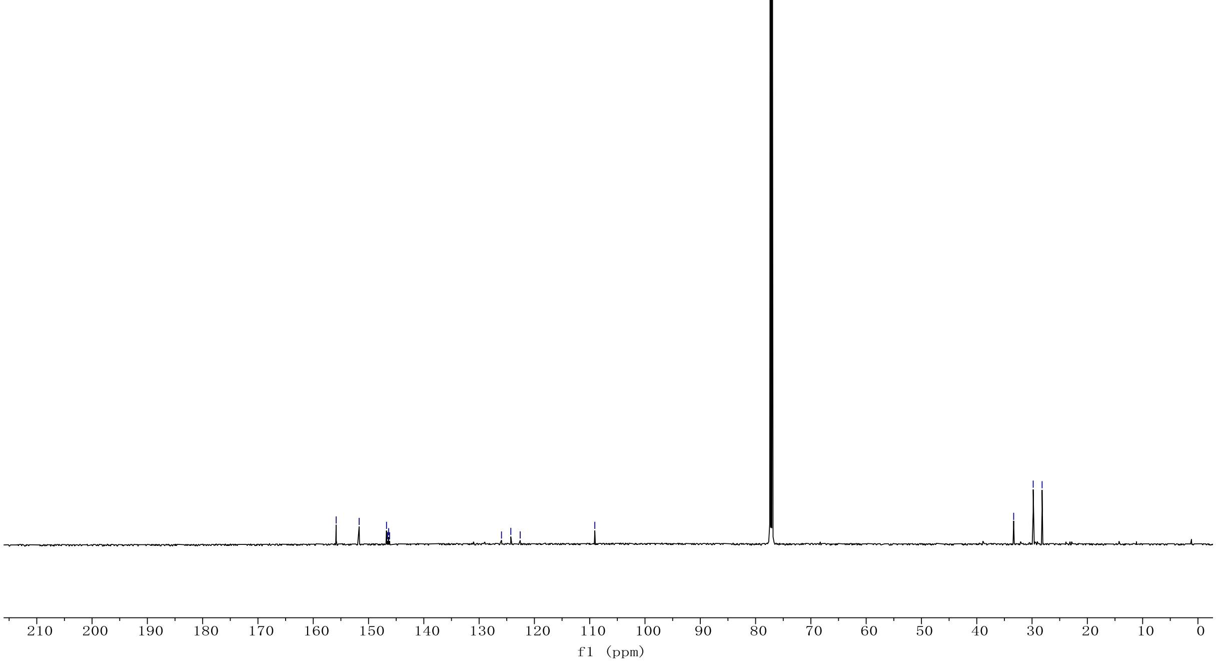

^{19}F-NMR (376 MHz, CDCl$_3$) of 22

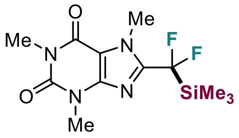

22

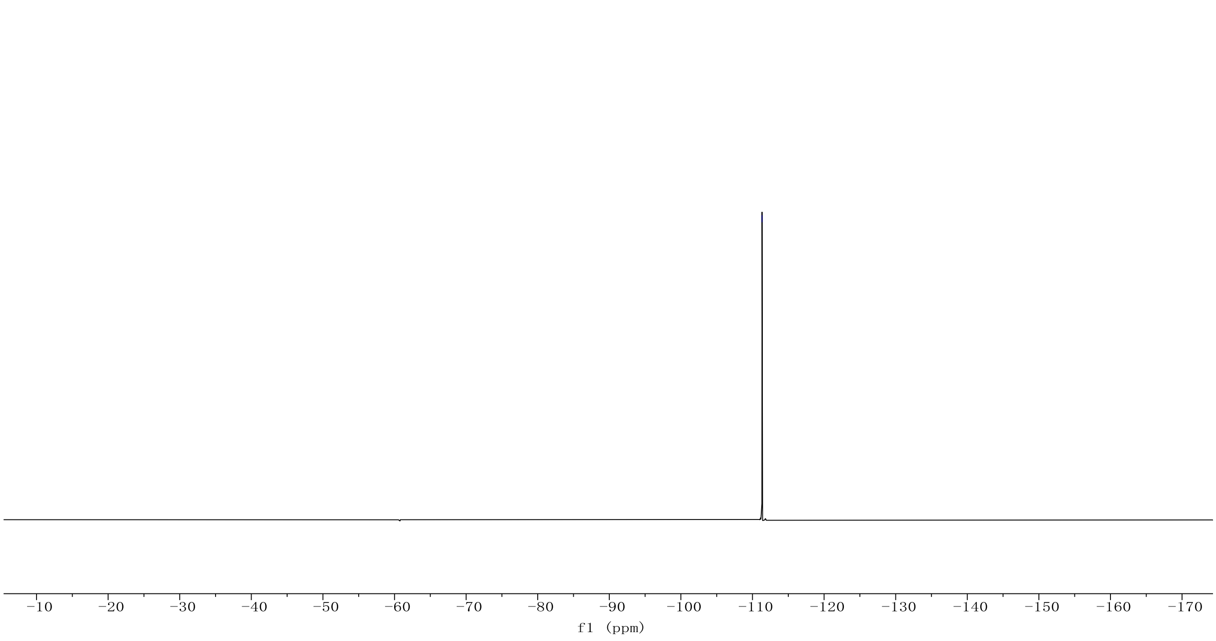

^{1}H-NMR (600 MHz, CDCl$_3$) of 23

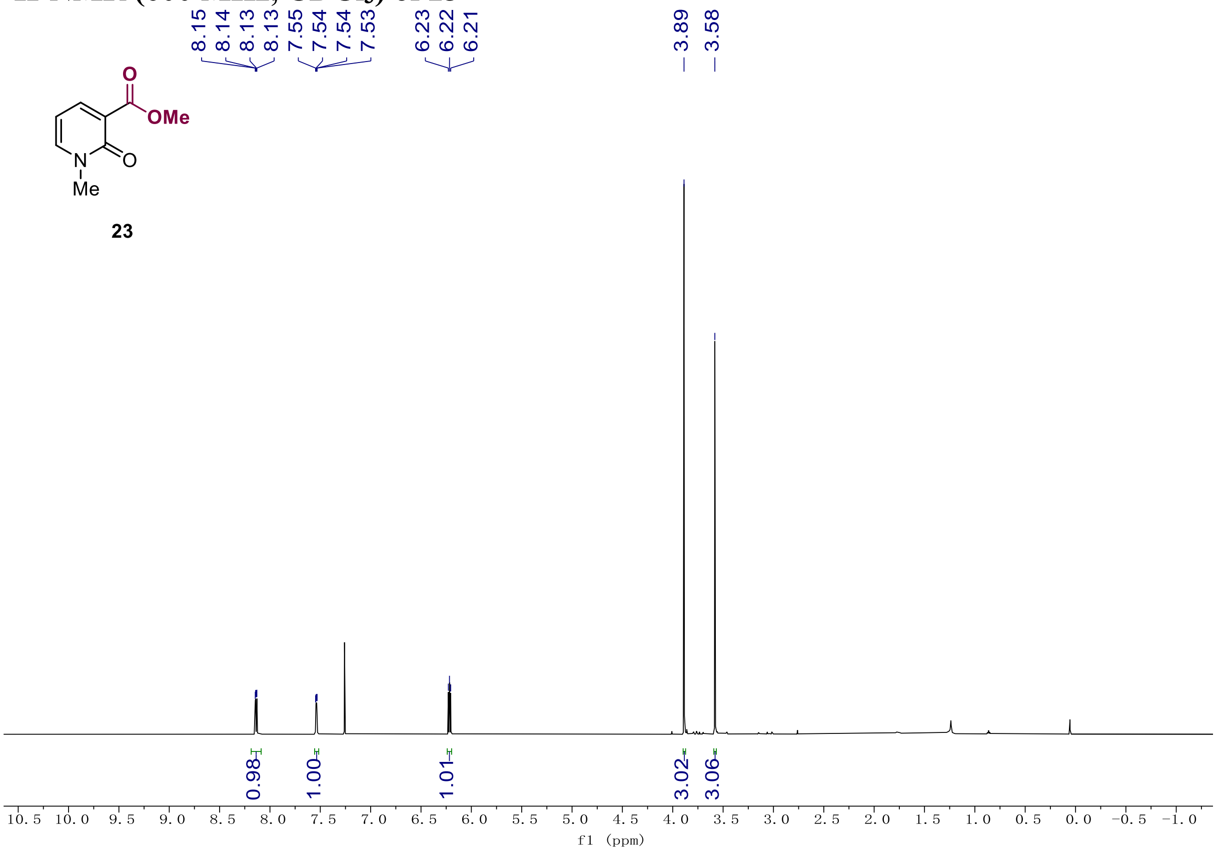

^{13}C-NMR (151 MHz, CDCl$_3$) of 23

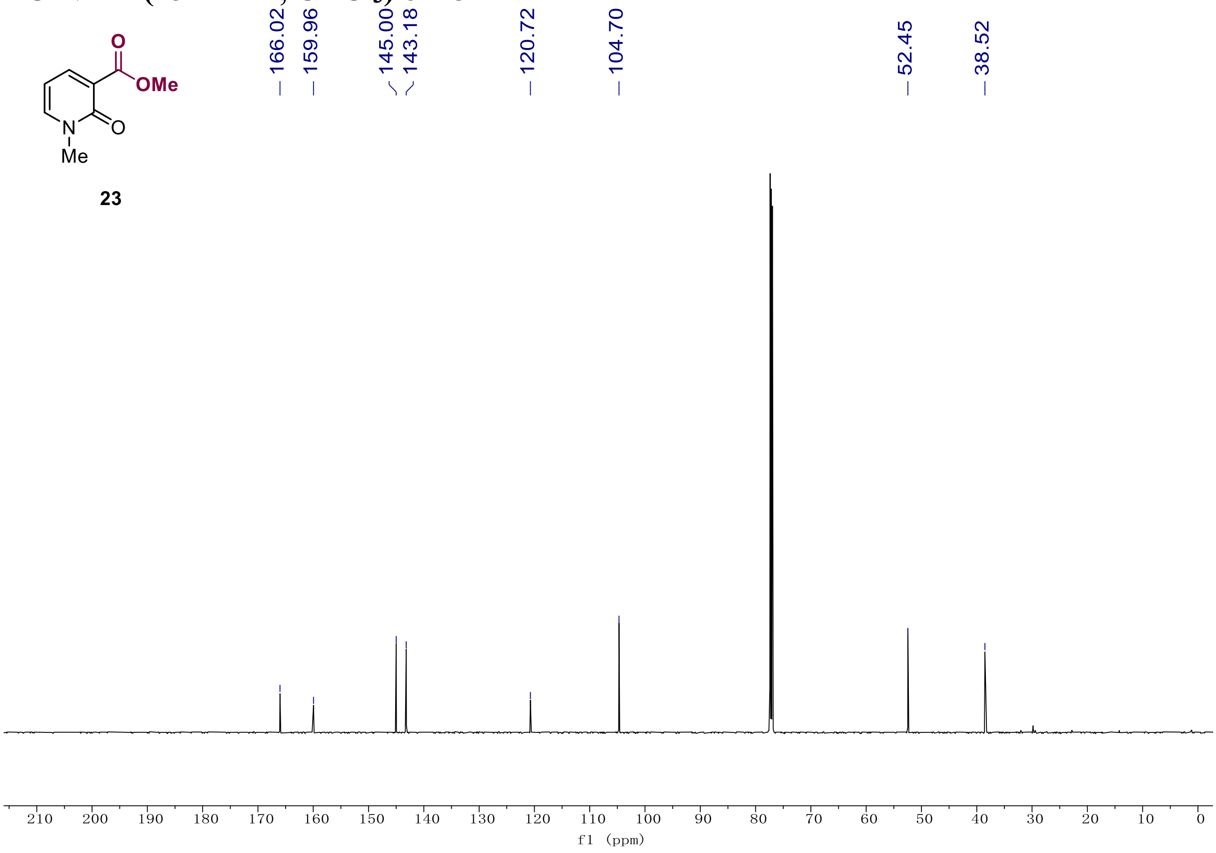

^{1}H-NMR (300 MHz, $CDCl_3$) of 24

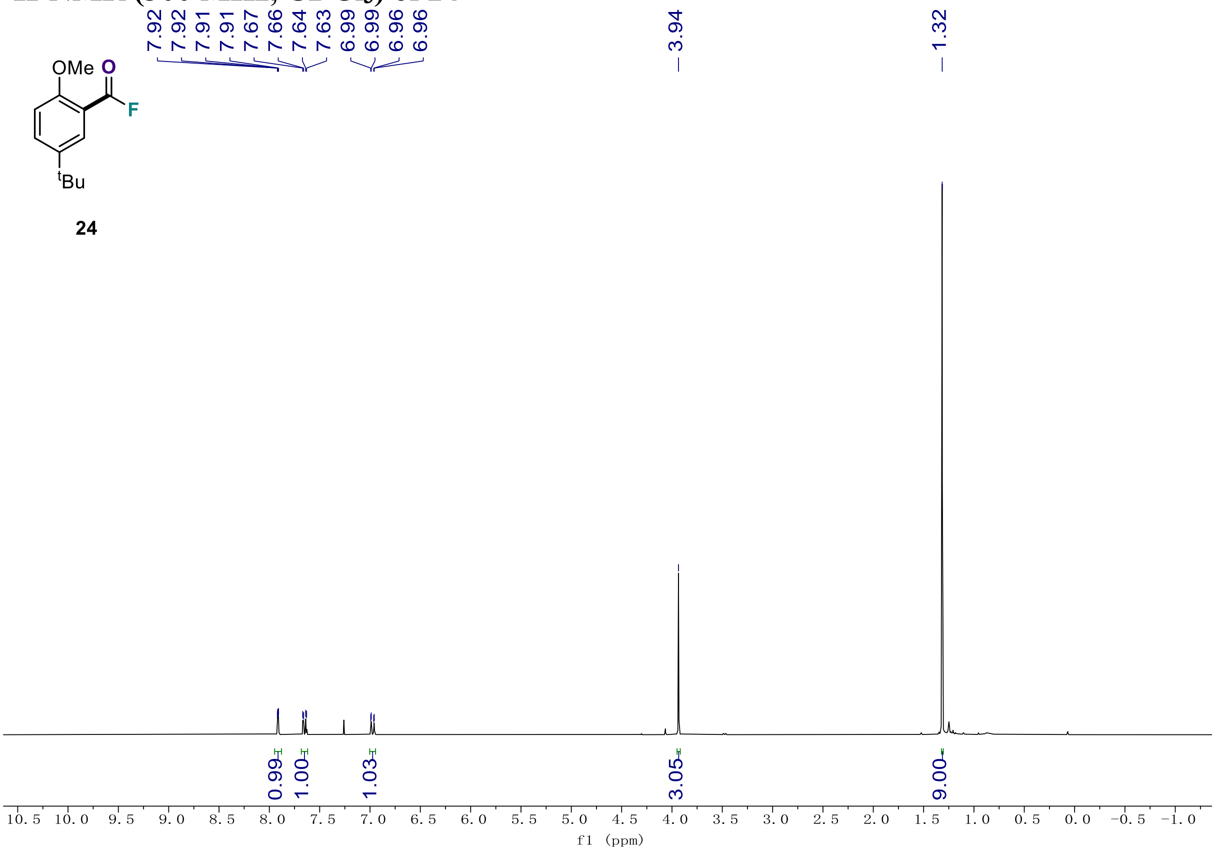

^{13}C-NMR (75 MHz, $CDCl_3$) of 24

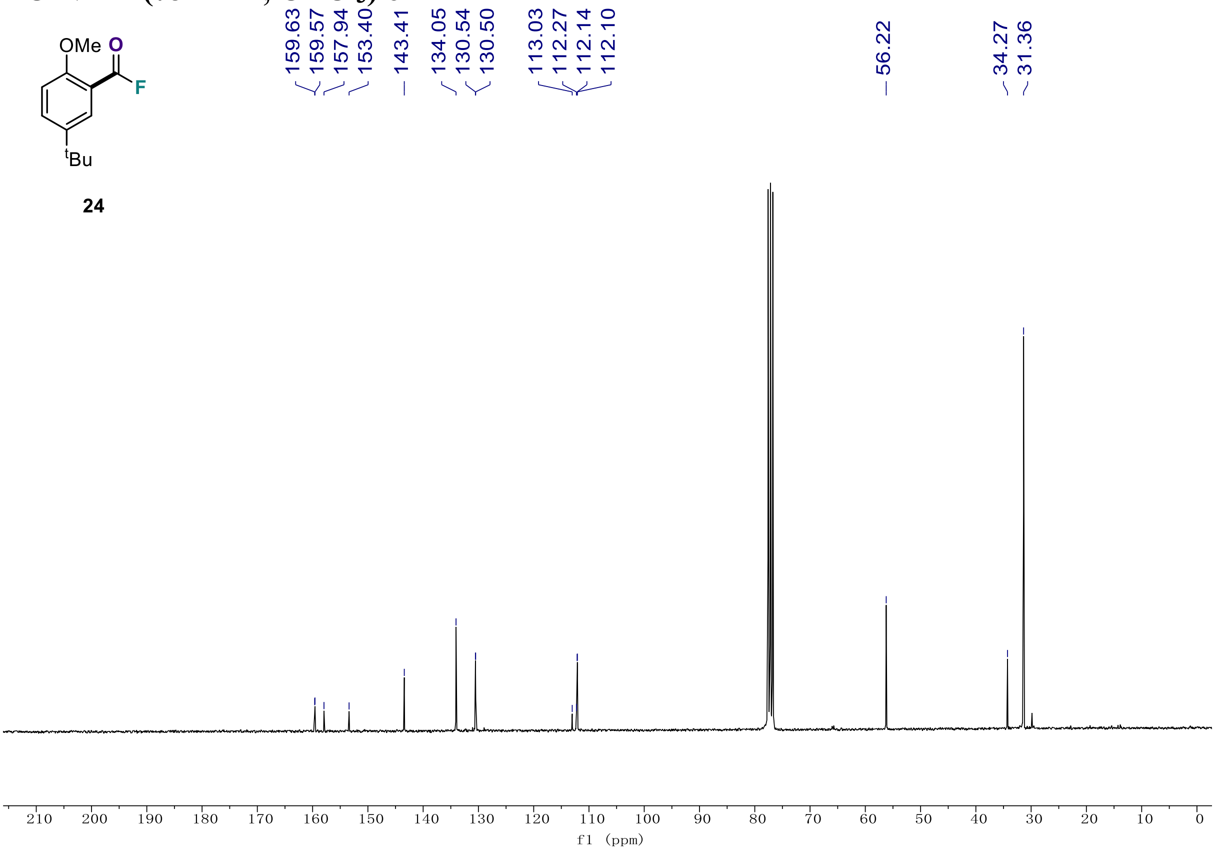

^{19}F-NMR (282 MHz, CDCl$_3$) of 24

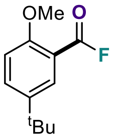

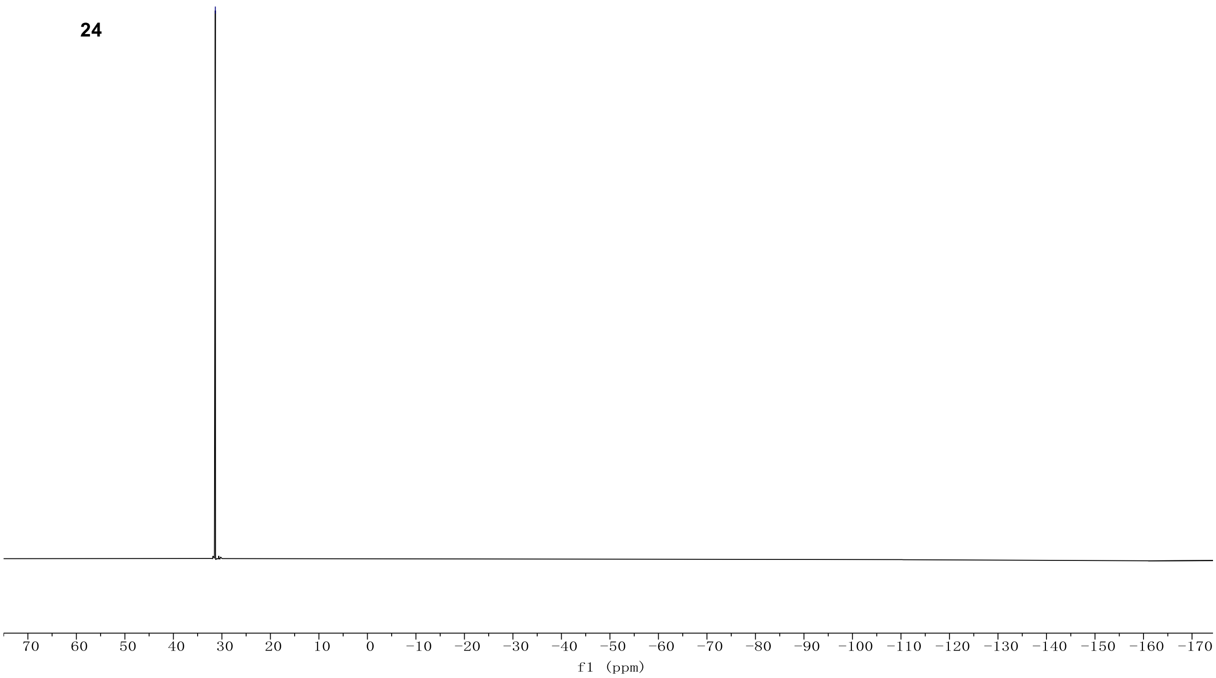

^{1}H-NMR (400 MHz, $CDCl_3$) of 25

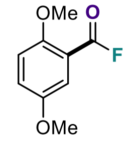

25

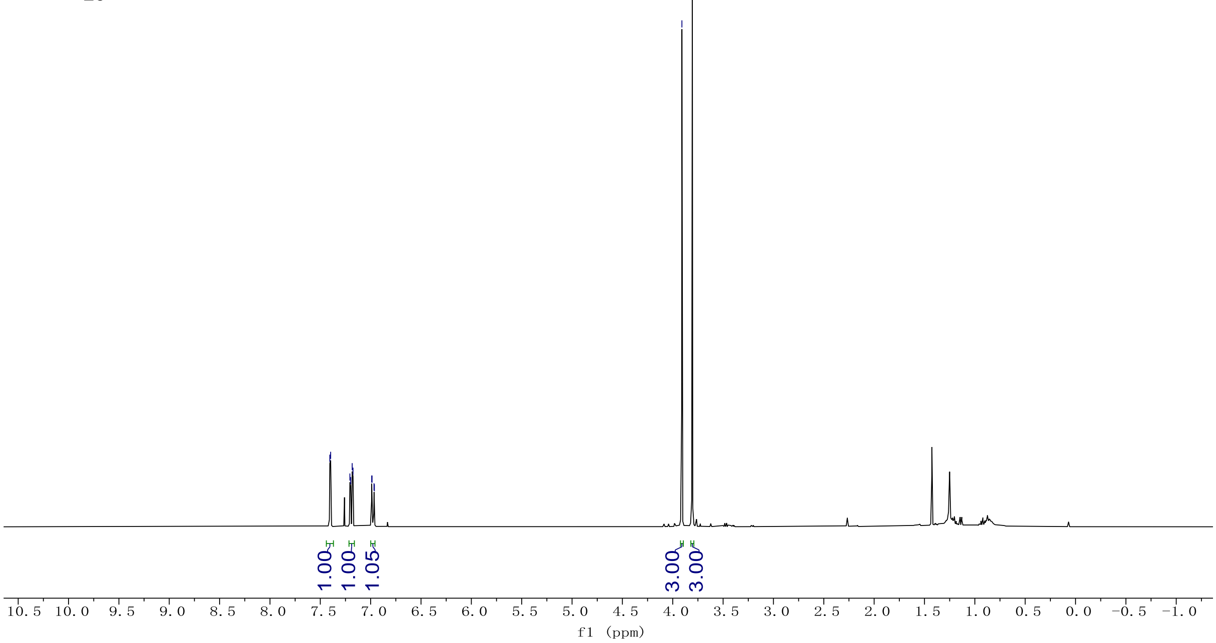

^{13}C-NMR (101 MHz, $CDCl_3$) of 25

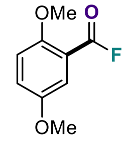

25

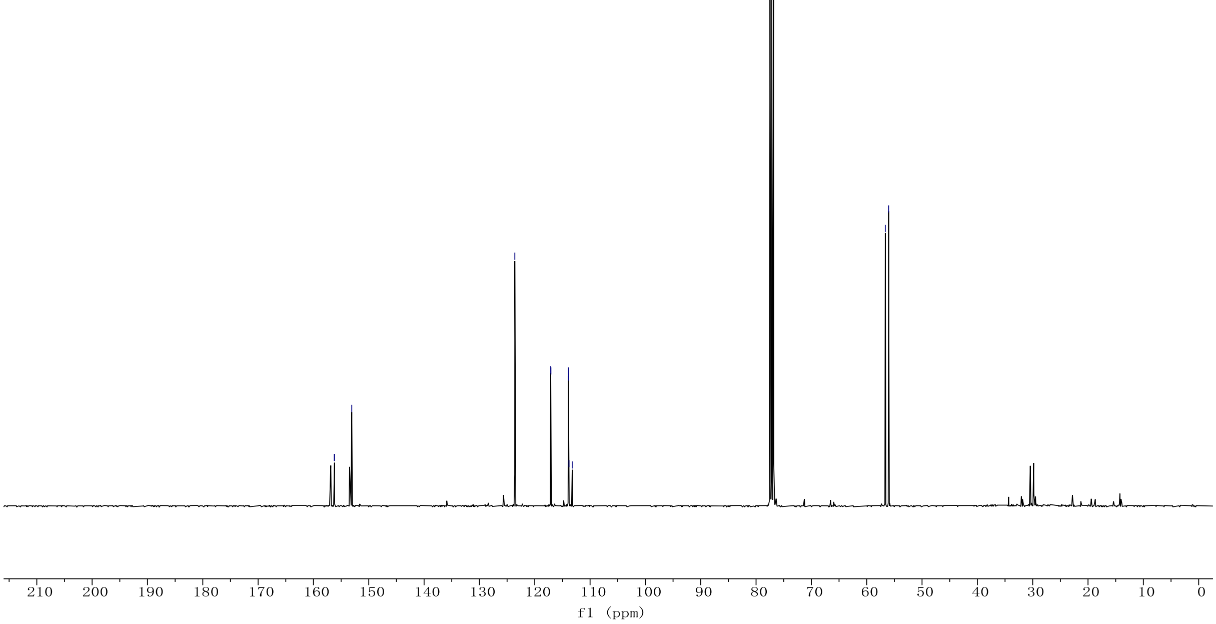

^{19}F-NMR (376 MHz, $CDCl_3$) of 25

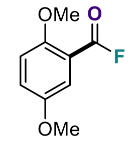

25

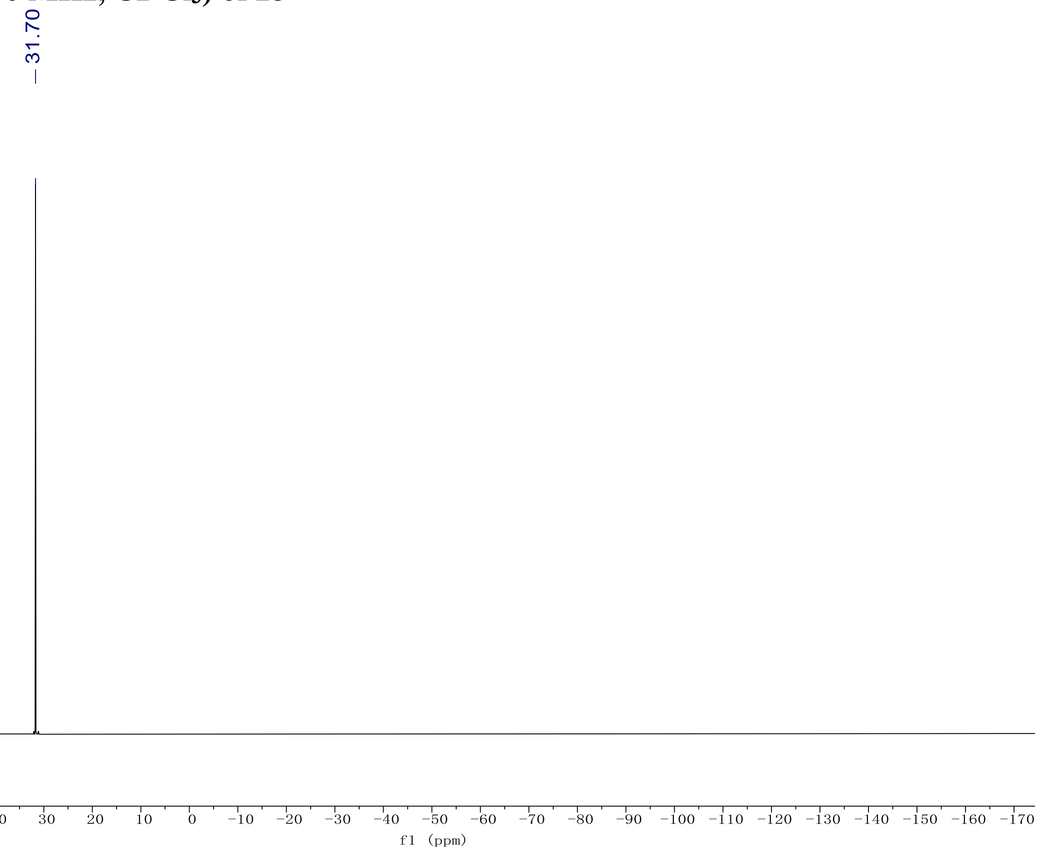

^{1}H-NMR (400 MHz, $CDCl_3$) of 26

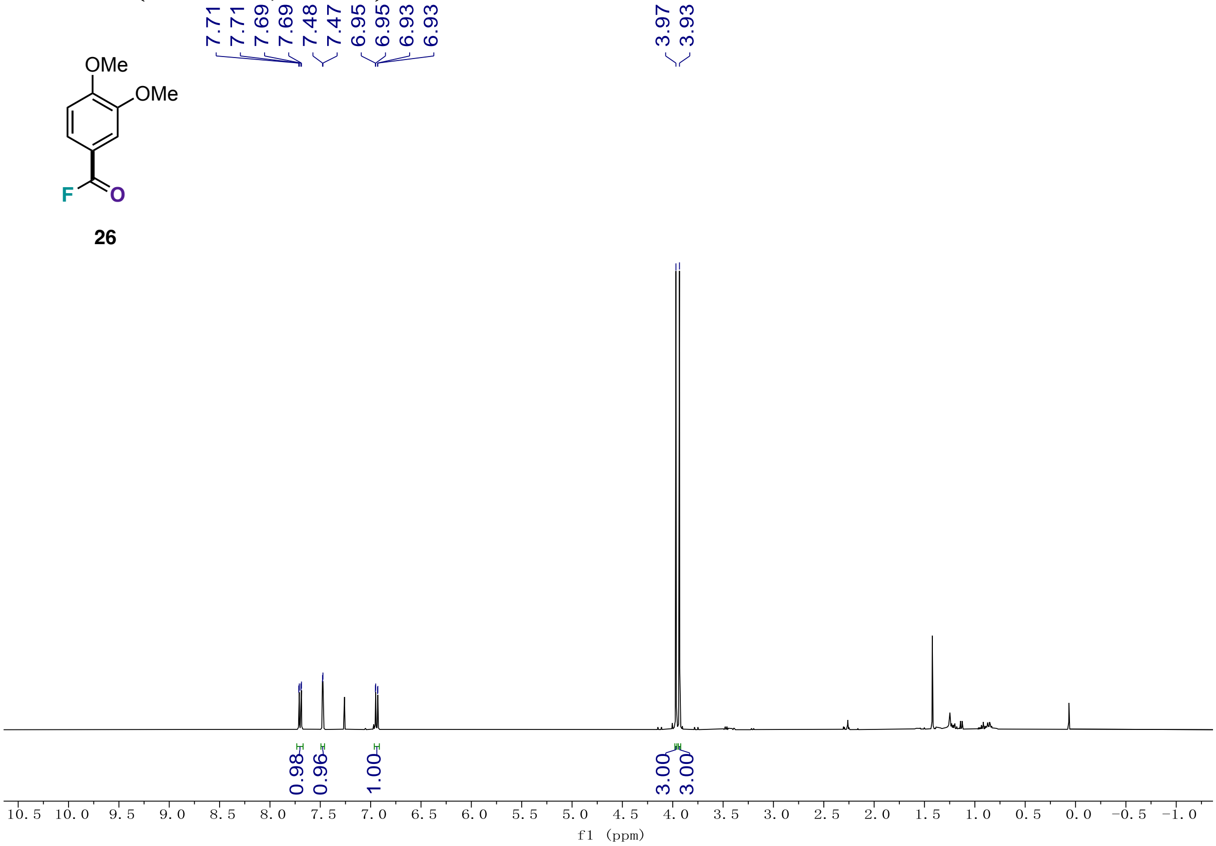

^{13}C-NMR (101 MHz, $CDCl_3$) of 26

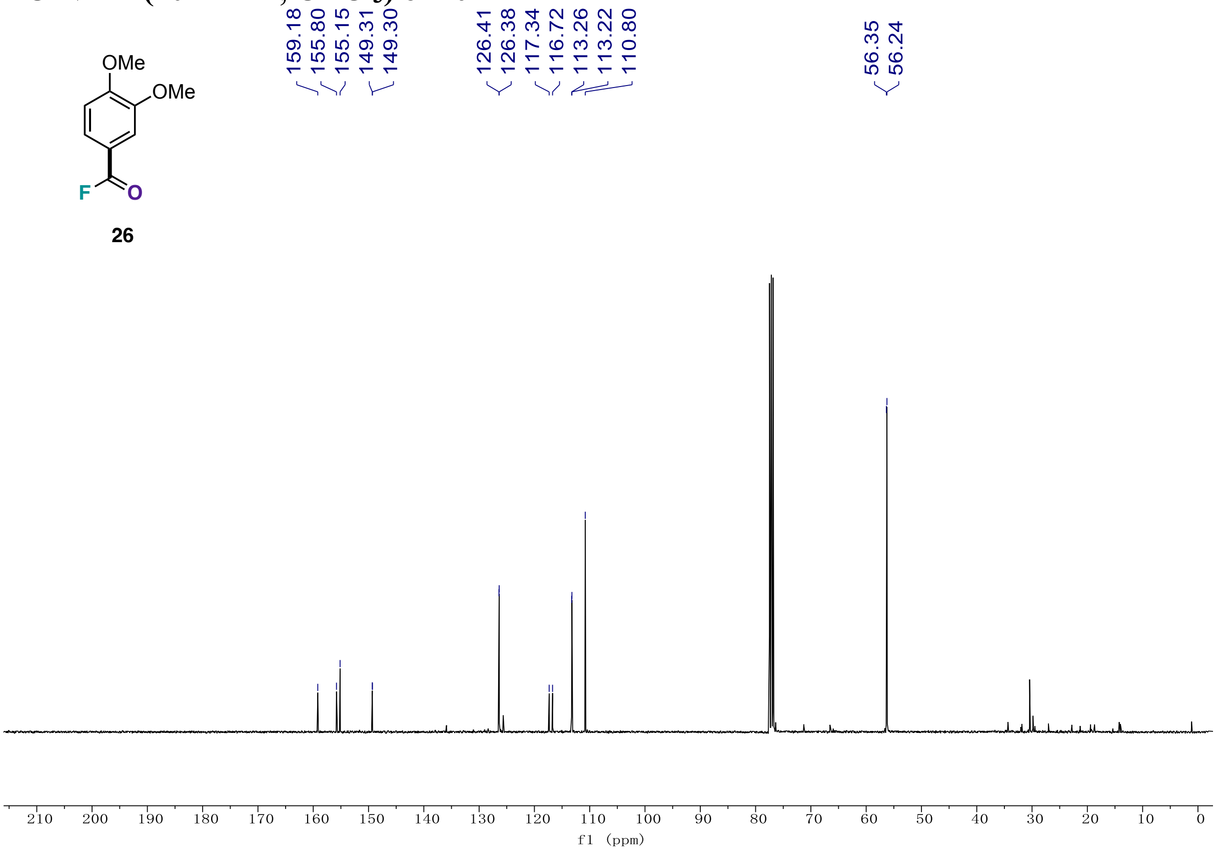

^{19}F-NMR (376 MHz, CDCl$_3$) of 26

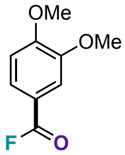

26

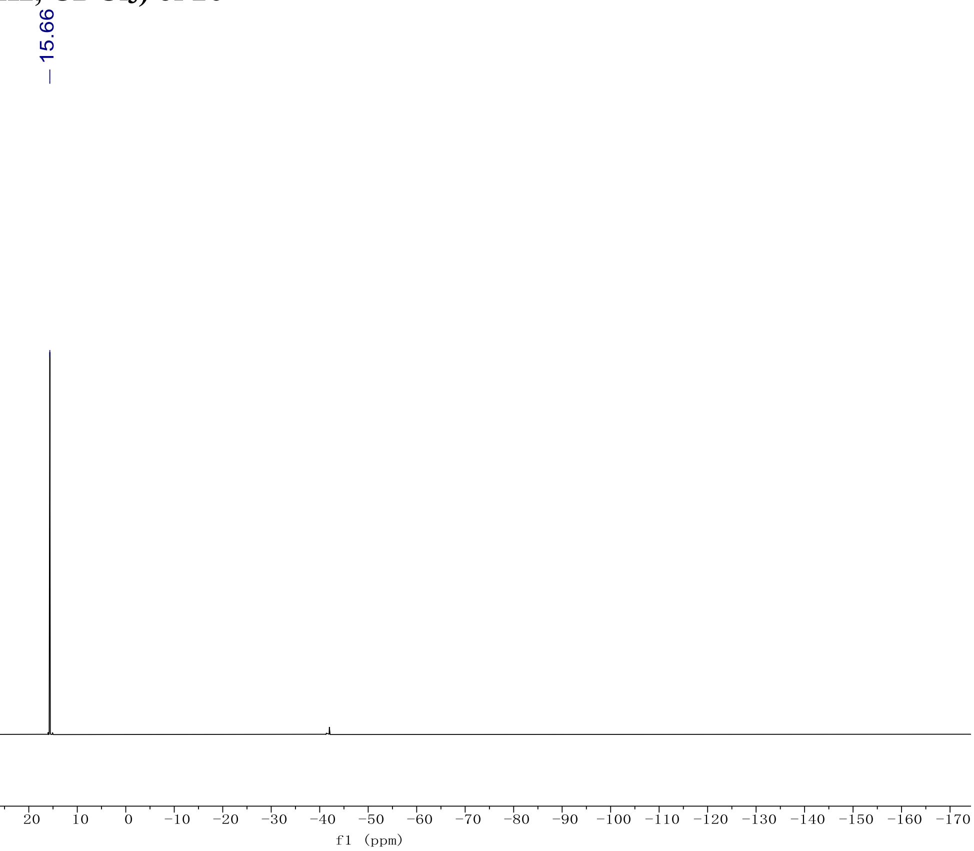

^{1}H-NMR (400 MHz, CDCl$_3$) of 27

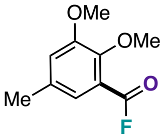

27

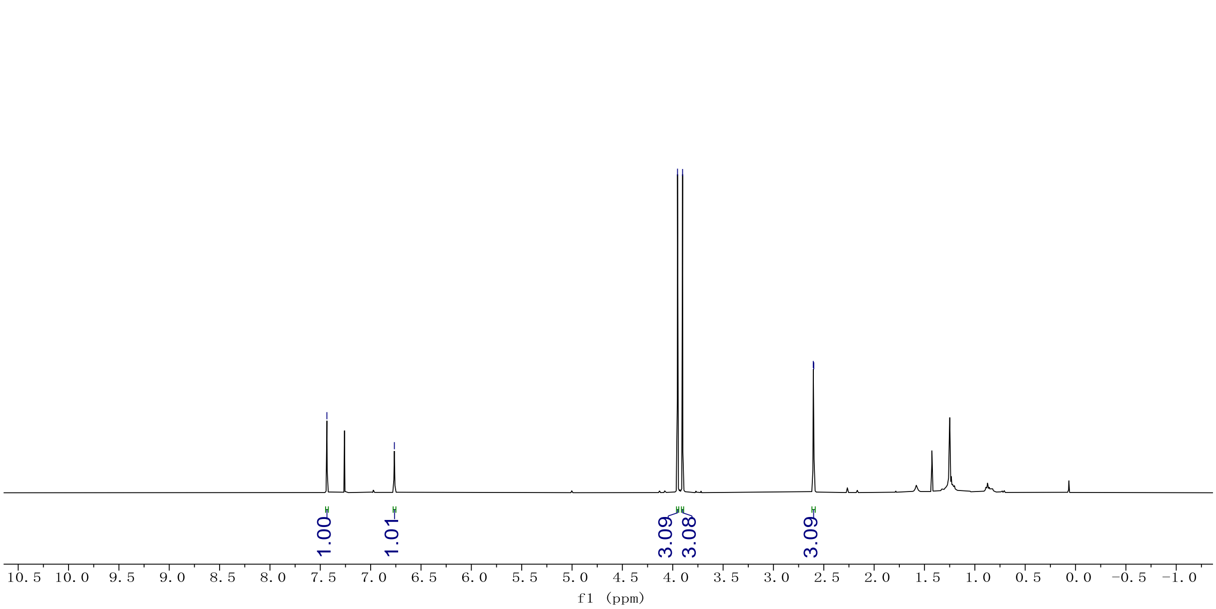

^{13}C-NMR (101 MHz, CDCl$_3$) of 27

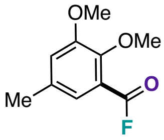

27

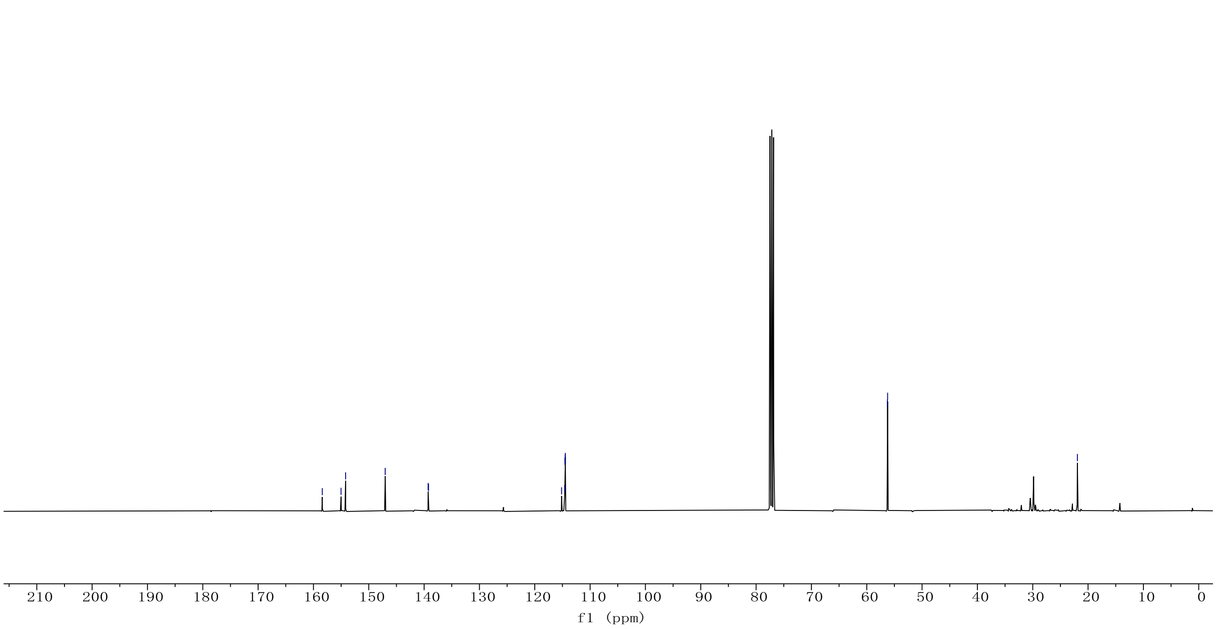

^{19}F-NMR (376 MHz, $CDCl_3$) of 27

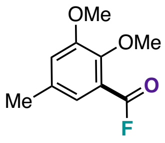

27

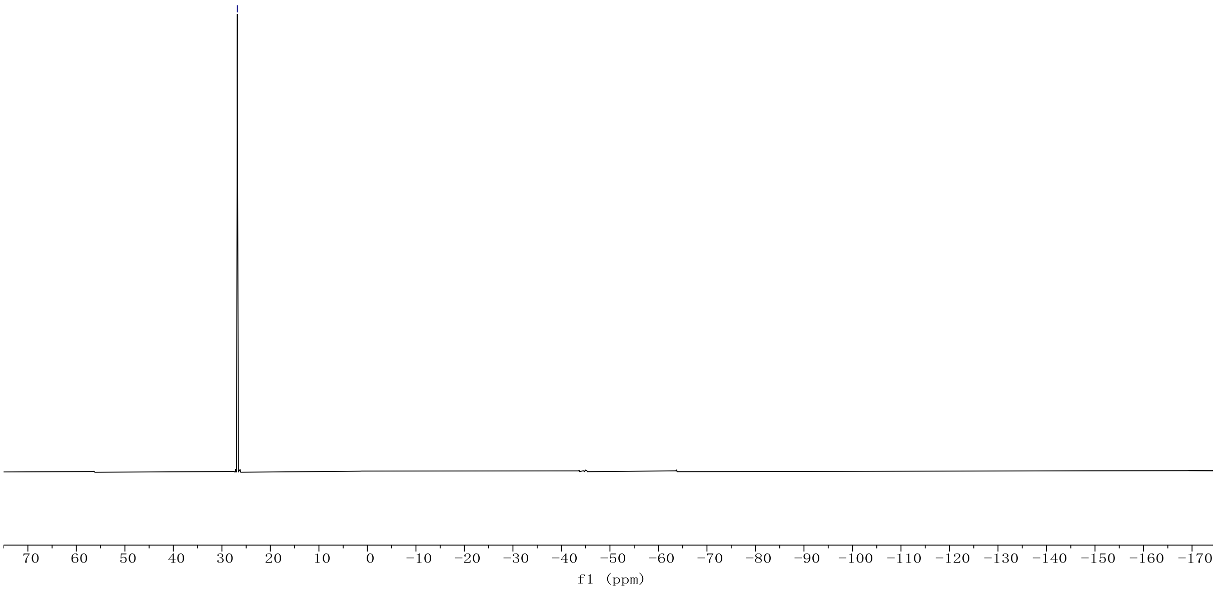

^{1}H-NMR (400 MHz, $CDCl_3$) of 28

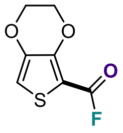

28

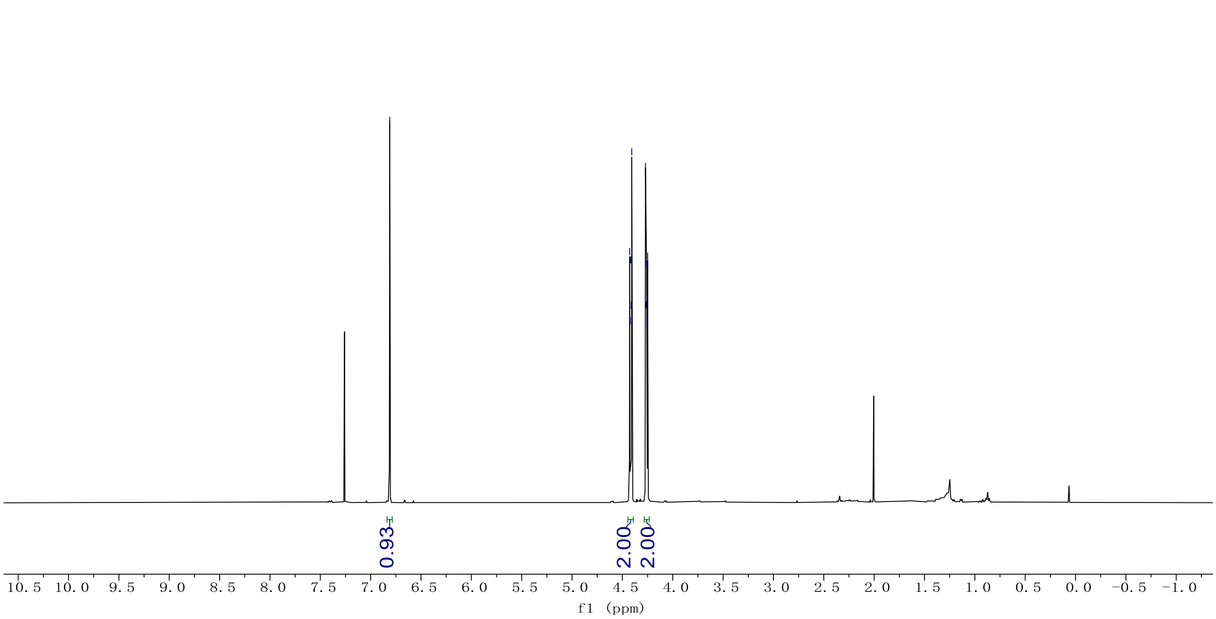

^{13}C-NMR (101 MHz, $CDCl_3$) of 28

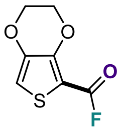

28

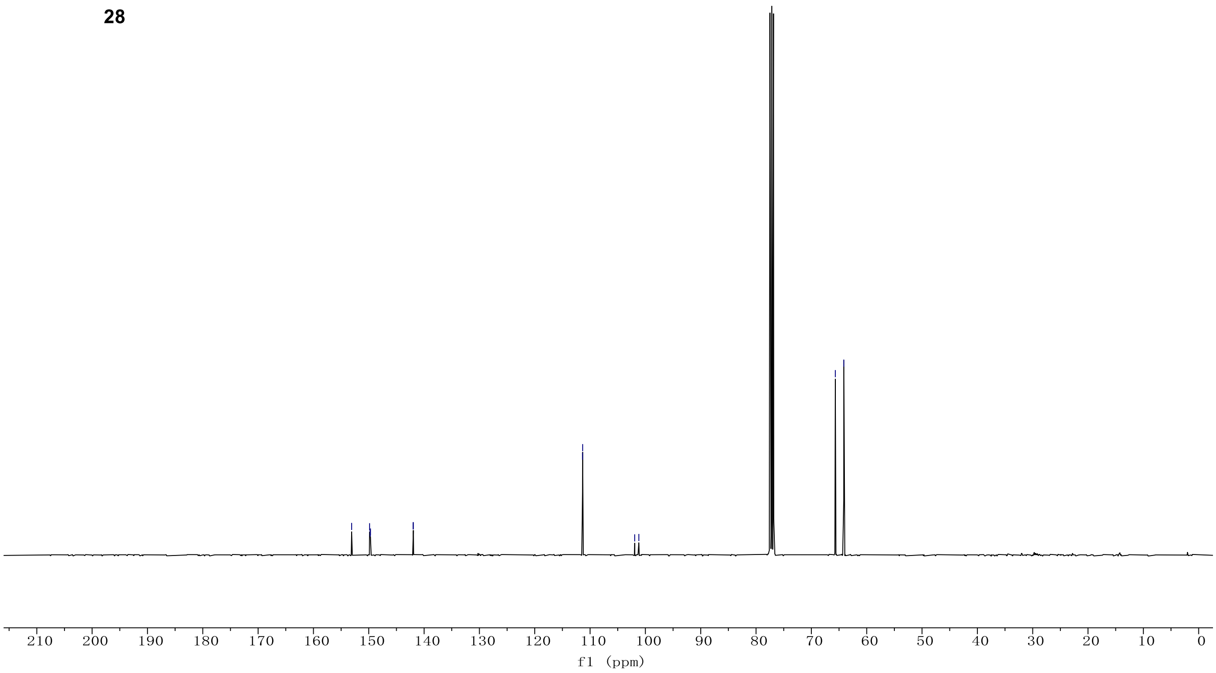

^{19}F-NMR (376 MHz, $CDCl_3$) of 28

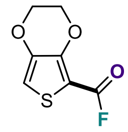

28

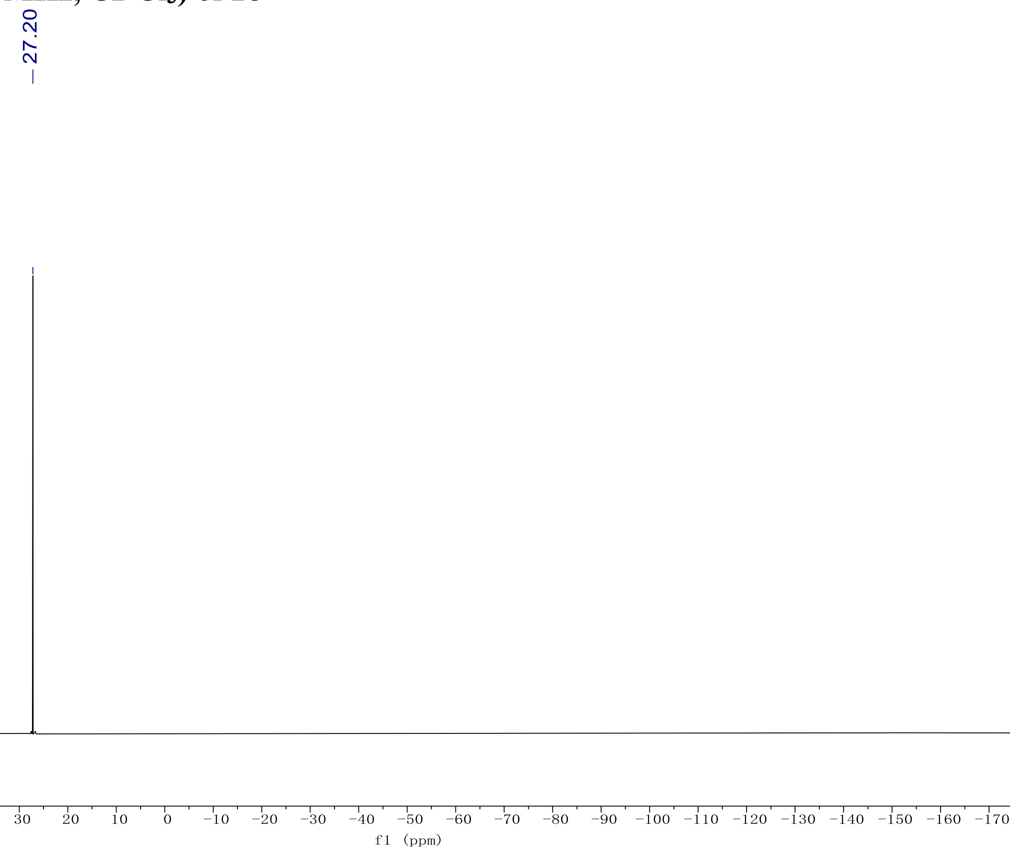

^{1}H-NMR (400 MHz, CDCl$_3$) of 29

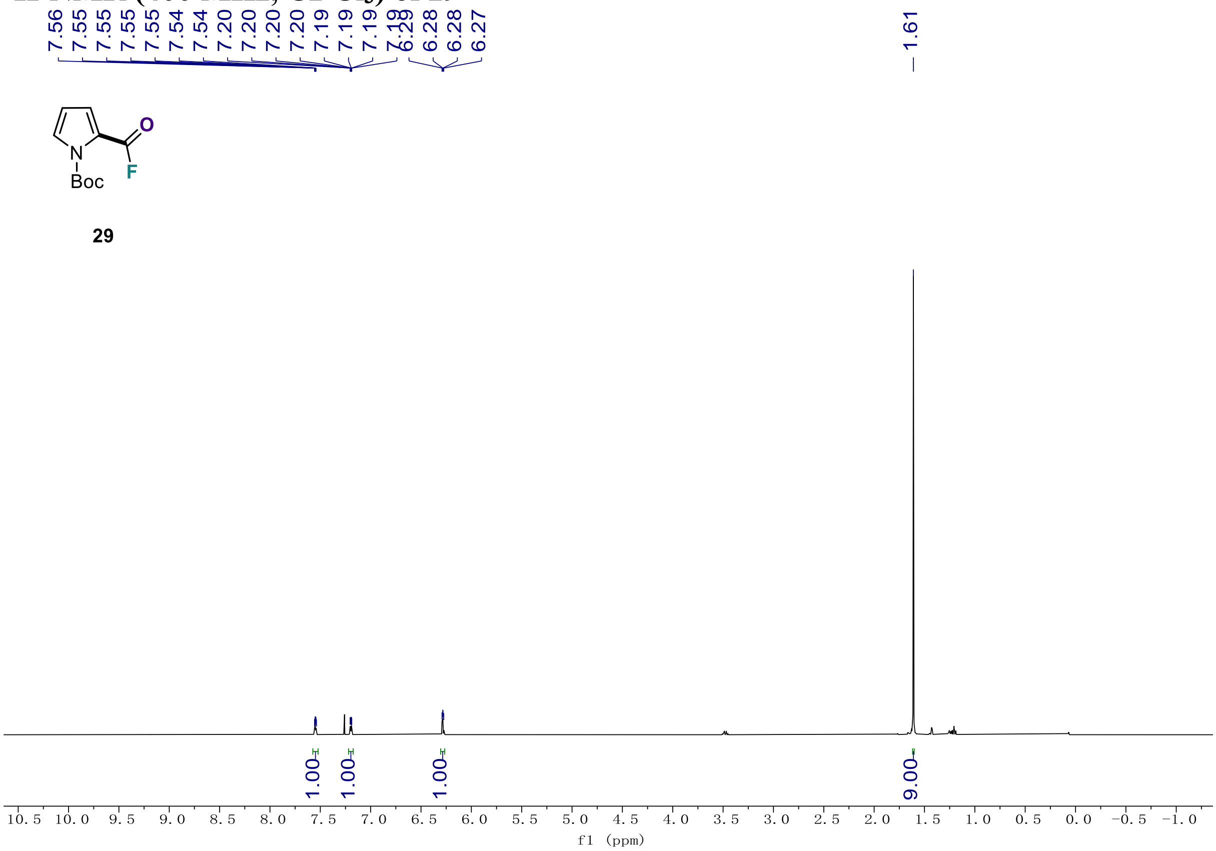

^{13}C-NMR (101 MHz, CDCl$_3$) of 29

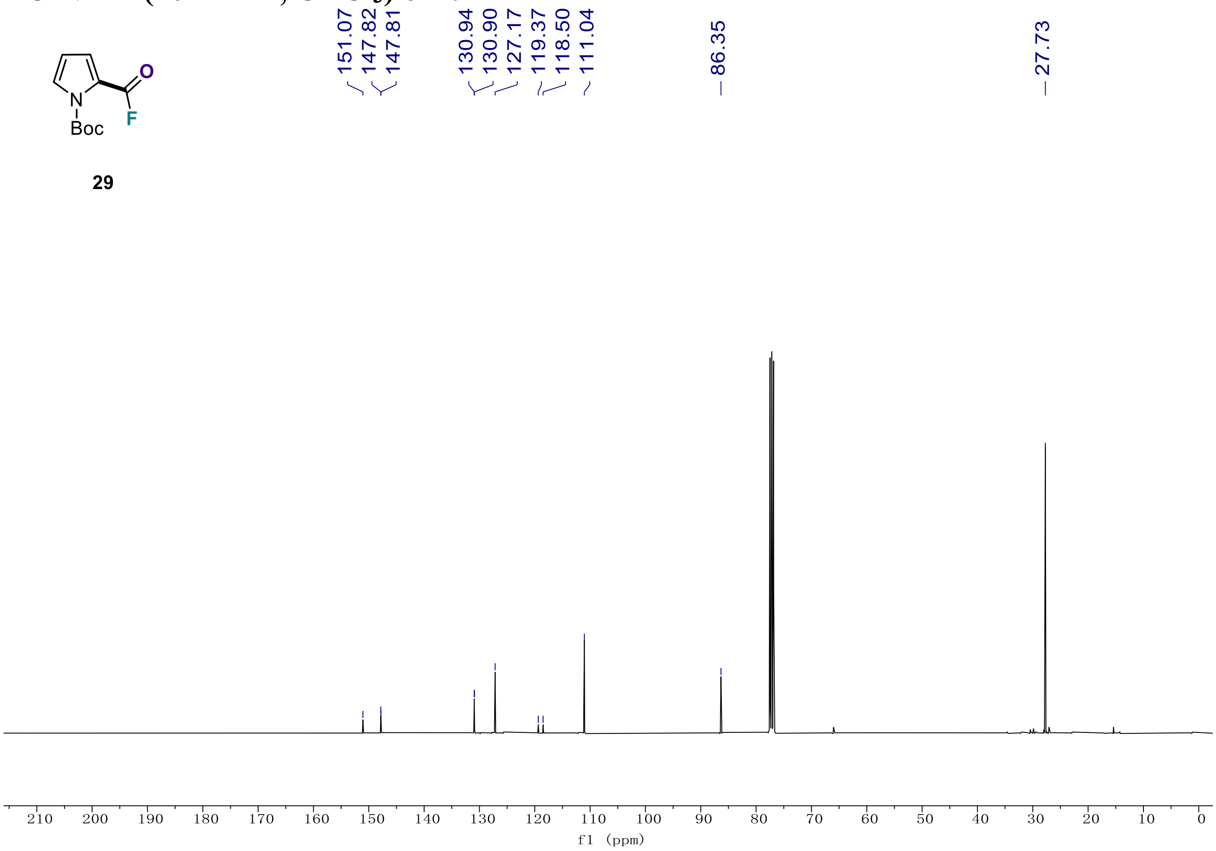

^{19}F-NMR (376 MHz, $CDCl_3$) of 29

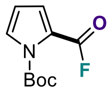

29

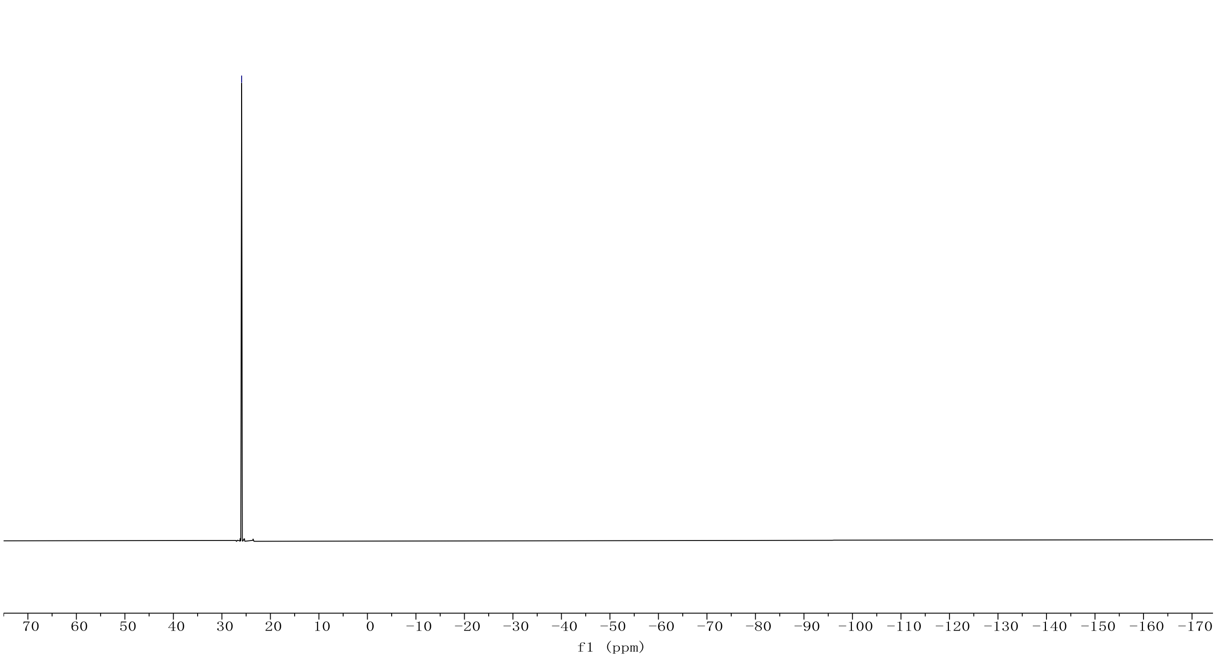

^{1}H-NMR (400 MHz, CDCl$_3$) of 30

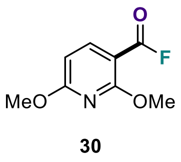

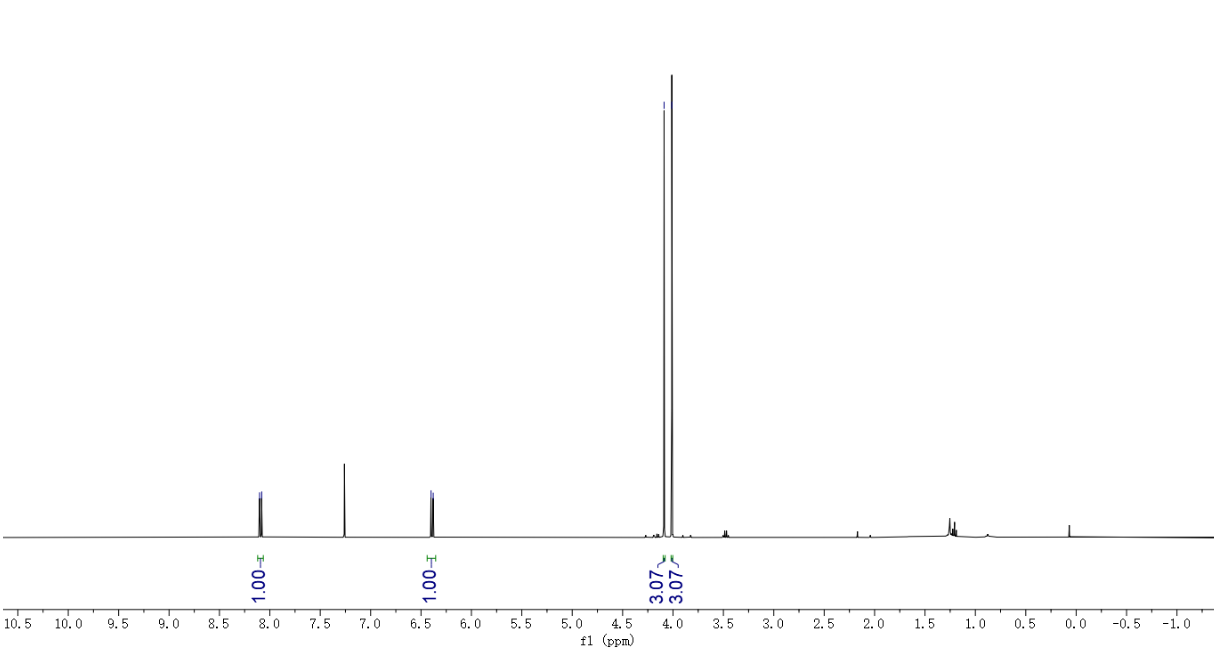

^{13}C-NMR (151 MHz, CDCl$_3$) of 30

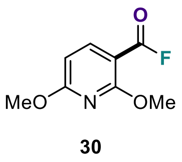

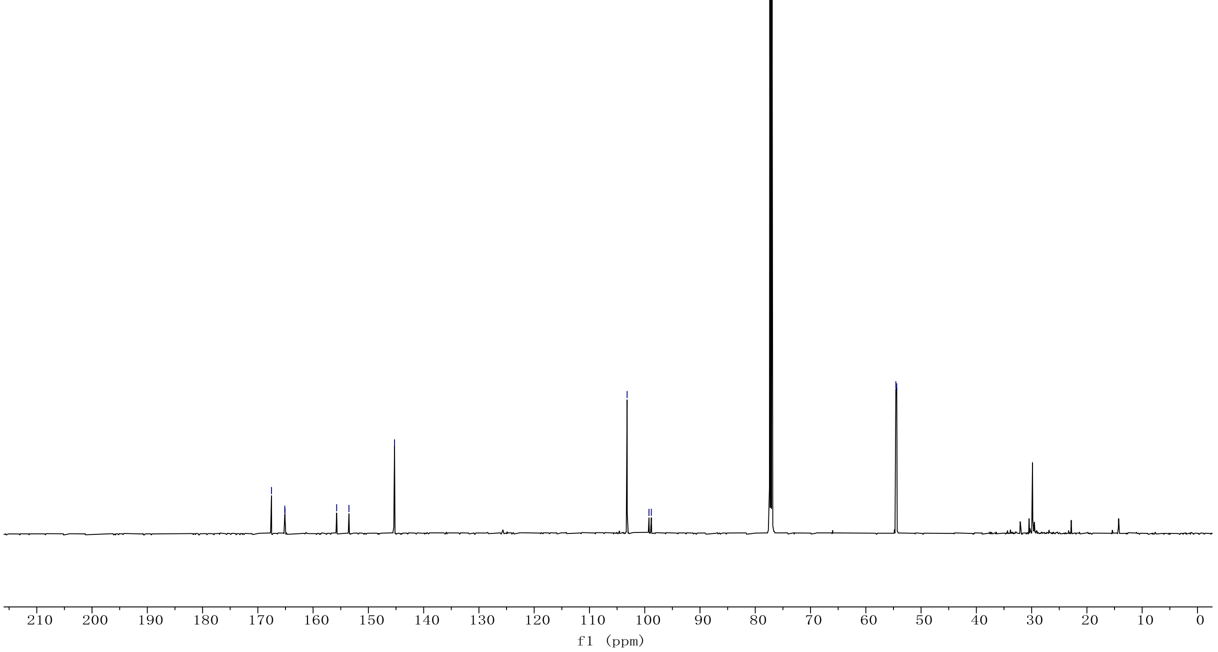

^{19}F-NMR (376 MHz, $CDCl_3$) of 30

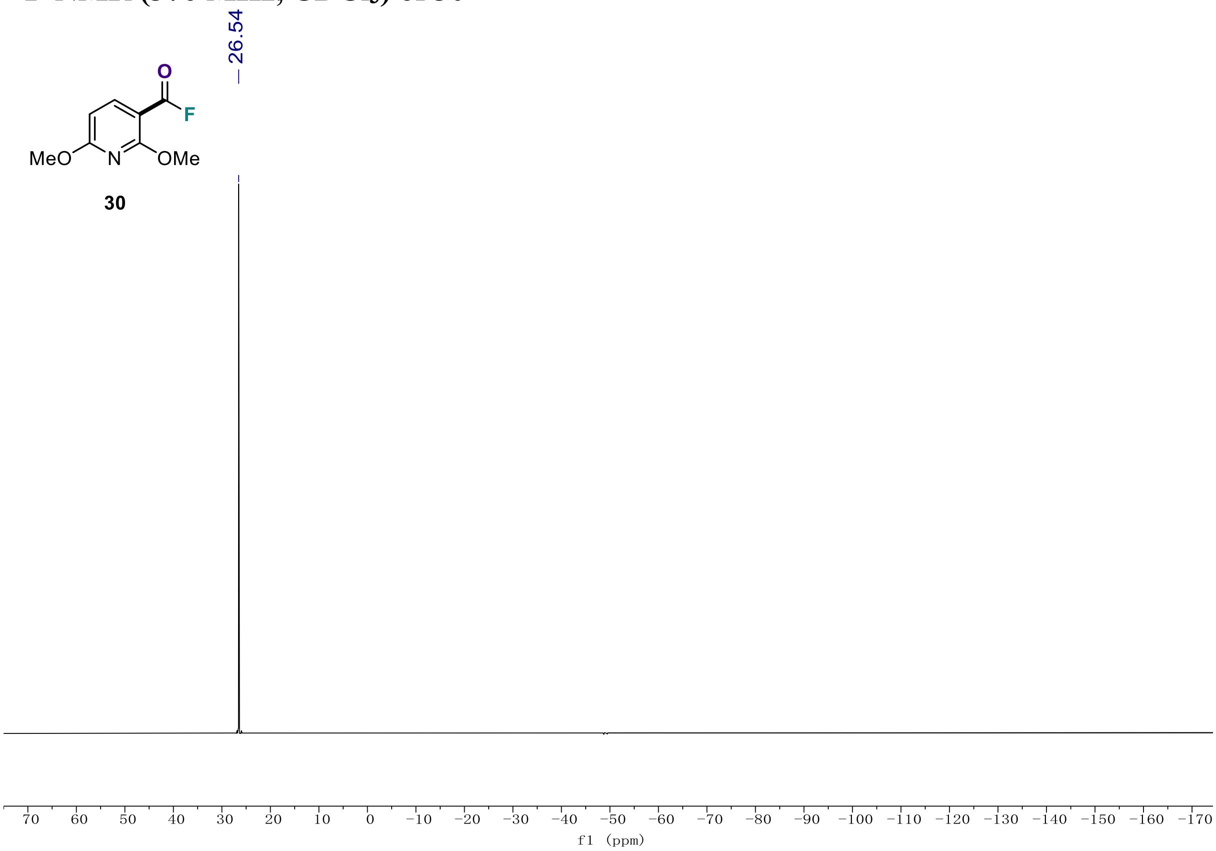

^{1}H-NMR (400 MHz, $CDCl_3$) of 31

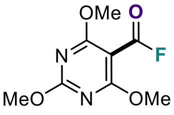

31

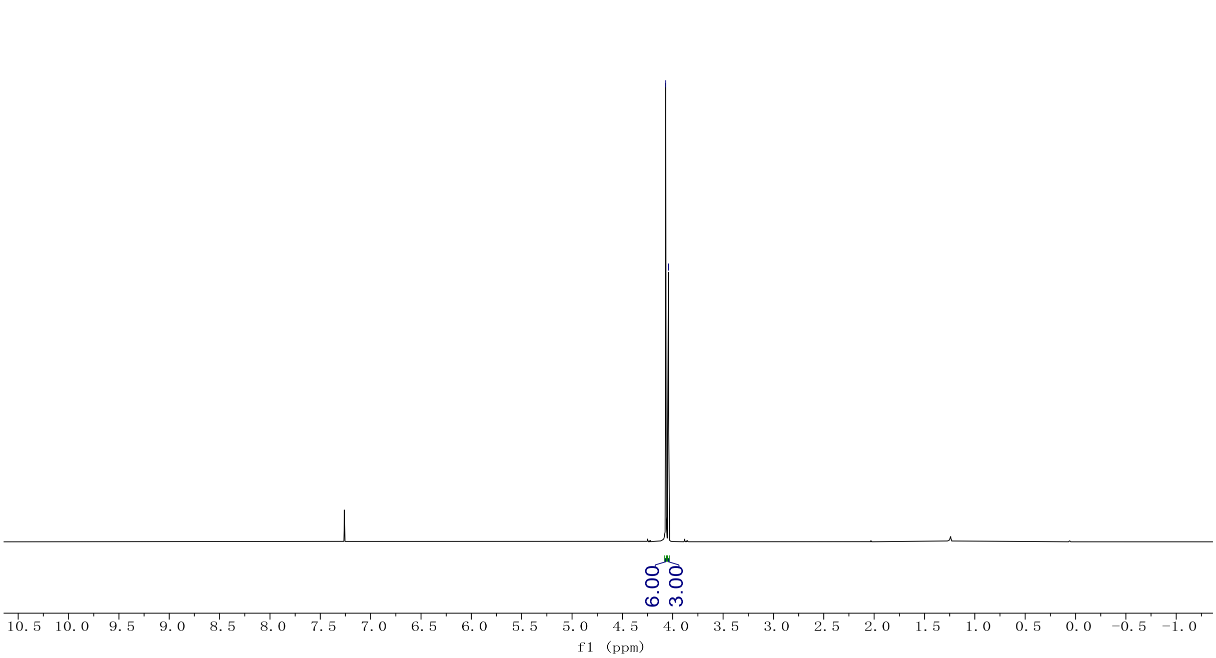

^{13}C-NMR (101 MHz, $CDCl_3$) of 31

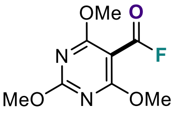

31

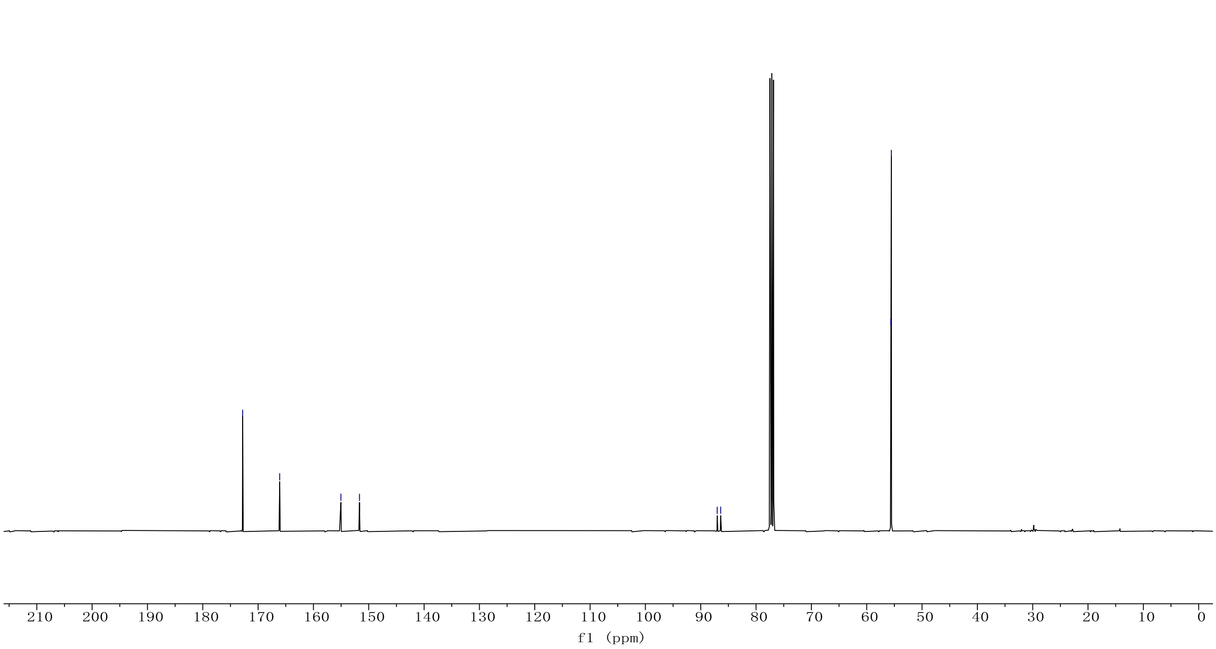

^{19}F-NMR (376 MHz, $CDCl_3$) of 31

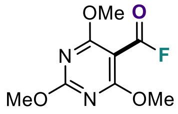

31

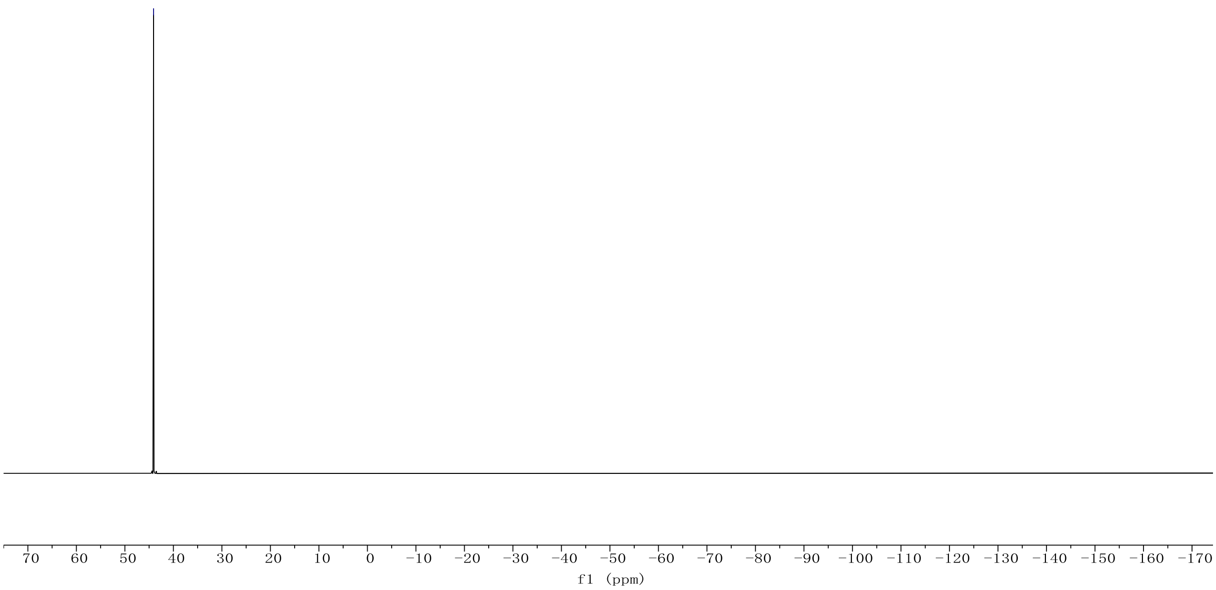

^{1}H-NMR (400 MHz, $CDCl_3$) of 32

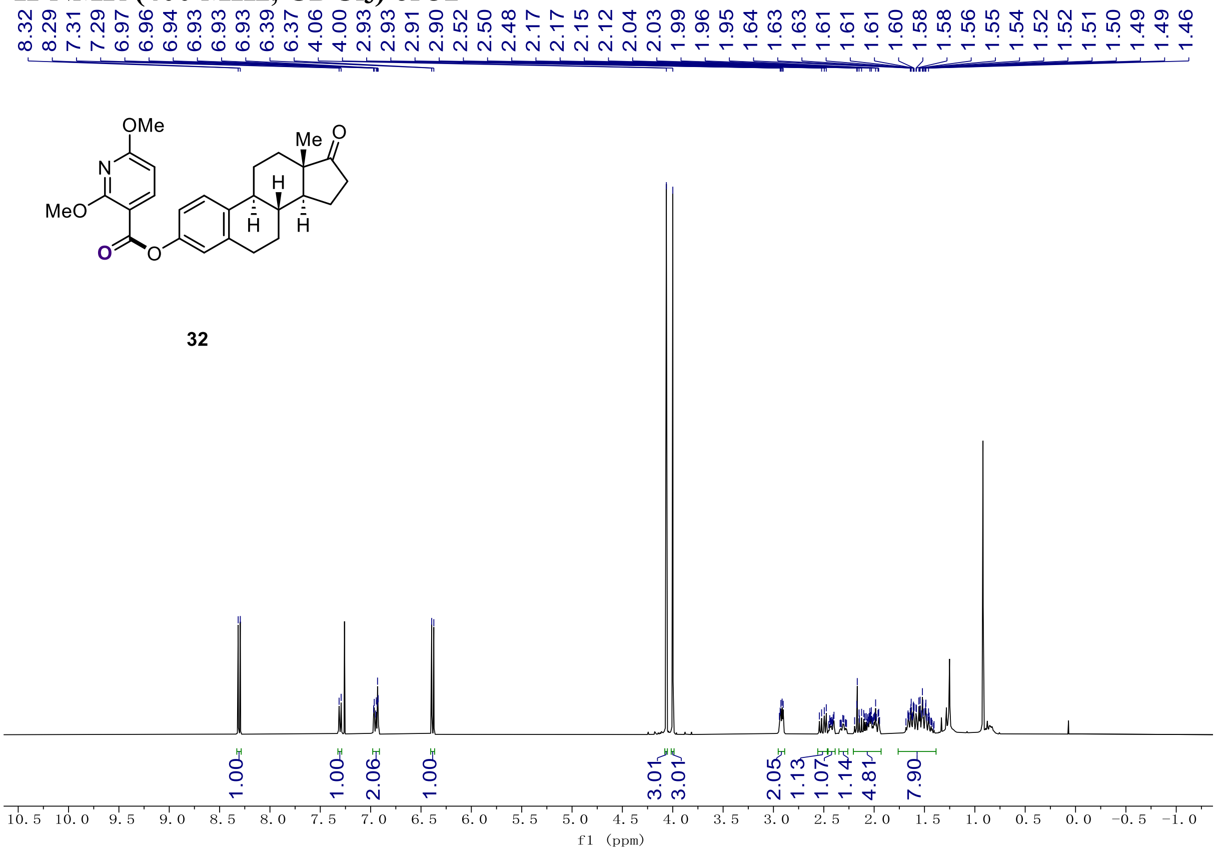

^{13}C-NMR (101 MHz, $CDCl_3$) of 32

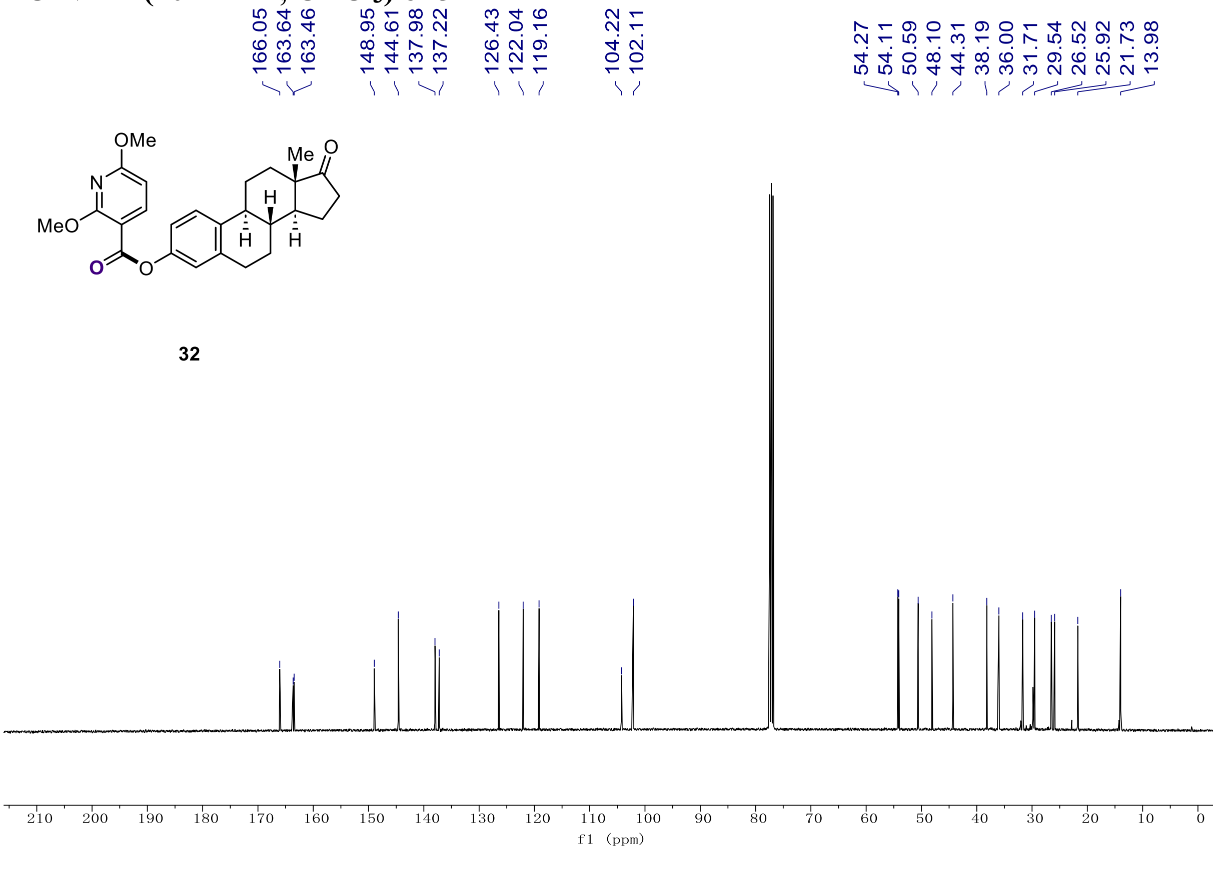

^{1}H-NMR (400 MHz, $CDCl_3$) of 33

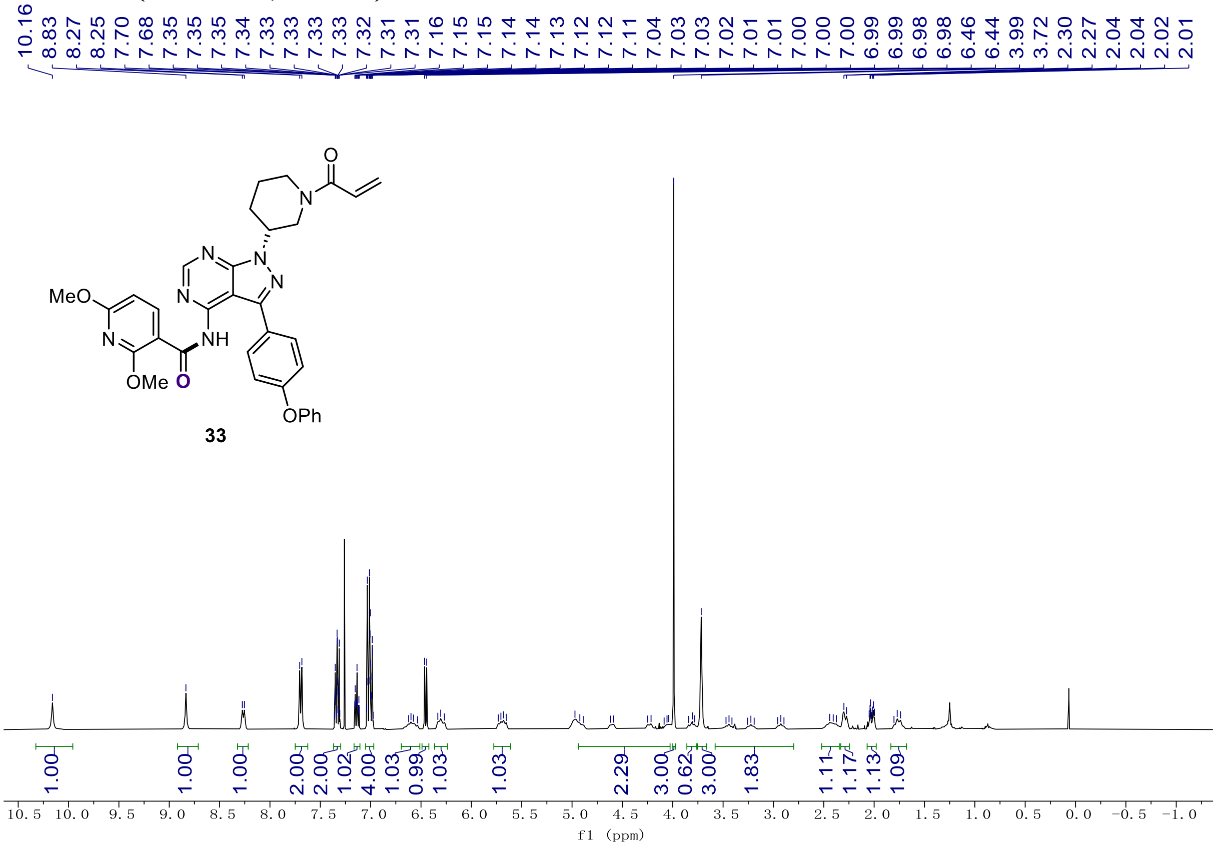

^{13}C-NMR (101 MHz, $CDCl_3$) of 33

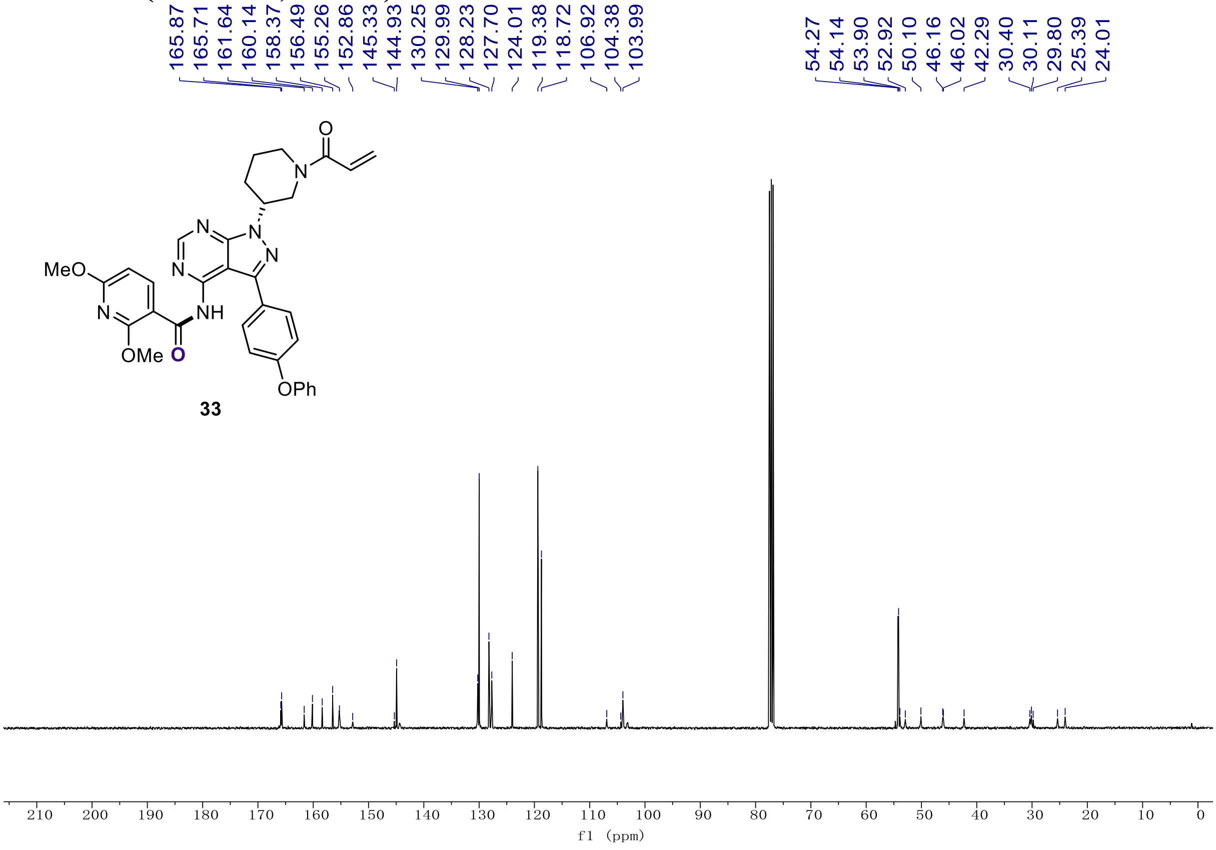

Supplement: Supplementary file 1 [file ol6c01214_si_001.pdf]
